# Learning curve for the laparoscopy-guided kidney biopsy procedure in small corpses of dogs and pigs

**DOI:** 10.1371/journal.pone.0257653

**Published:** 2021-09-27

**Authors:** Suellen Rodrigues Maia, Pamela Almerinda Mendes, Felipe Farias Pereira da Câmara Barros, Ilan Munhoz Ayer, Salvador Boccaletti Ramos, Alessandra Marieeli Vacari, Tiago Machado Carneiro Lucera, Vanessa Yurika Murakami, Leonardo Lamarca de Carvalho, Pedro Negri Bernardino, Fernanda Nastri Gouvêa, Sofia Borin-Crivellenti, Leandro Zuccolotto Crivellenti

**Affiliations:** 1 Department of Veterinary Clinics, School of Veterinary Medicine and Animal Science, São Paulo State University (UNESP), Botucatu, São Paulo, Brazil; 2 Veterinary Medicine Graduate Student, University of Franca (UNIFRAN), Franca, São Paulo, Brazil; 3 Department of Veterinary Medicine and Surgery, Federal Rural University of Rio de Janeiro (UFRRJ), Seropédica, Rio de Janeiro, Brazil; 4 Department of Animal Science, UNA—Academic Center, Pouso Alegre, Minas Gerais, Brazil; 5 Health Promotion Graduate Program, University of Franca (UNIFRAN), Franca, São Paulo, Brazil; 6 Animal Science Graduate Program/Veterinary Teaching Hospital of University of Franca (UNIFRAN), Franca, São Paulo, Brazil; 7 Global Study Program, University of California Davis (UC DAVIS), Davis, California, United States of America; 8 Graduate Program in Veterinary Science (PPGCV) / College of Veterinary Medicine (FAMEV), Federal University of Uberlândia (UFU), Uberlândia, Minas Gerais, Brazil; Faculty of Veterinary Medicine - University of Lisbon, PORTUGAL

## Abstract

The use of renal biopsy through laparoscopy is increasingly present both in human and veterinary medicine. However, both techniques require skill and training to make the operator capable to do it. The learning curve allows the quantitative and qualitative assessment of the number of attempts and minimum time for the surgical procedure. The objective included establish the learning curve for laparoscopy-guided kidney biopsy procedures in dog and pig corpses. Six dogs and six pigs corpses weighing less than 10 kg were used for this study. All corpses underwent kidney biopsy performed through laparoscopy. Twenty-four operators, two per animal, performed 20 renal biopsies each (10 for each kidney), with 480 collection-procedures in total. Duration and difficulty of the procedure and the biopsy sample quality were evaluated and statistical analysis was performed using a mixed regression model with a random effect of individuals and multivariate analysis of data. There were 91.5% of the samples that were adequate for evaluation. There was no significant difference in the number of glomeruli or cortex percentage considering the attempts in either species, demonstrating the operator’s ability since first collection. Swine samples showed higher amounts of renal cortex than canine samples. The procedure duration was shorter as more attempts were performed in dogs and pigs. From the fourth repetition, the professional reached a plateau for the variable related to ‘collection’, and from the second, the professional presented uniform duration for ‘sample storage’. Operators of the swine model acquired more agility than the dog ones. The variable ‘difficulty’ decreased as more repetitions were performed, reaching a plateau in the sixth attempt. Seven renal biopsies laparoscopy-guided are required for an operator to be considered ‘capable’ to perform the procedure in the referred species included. The learning curve for image-guided kidney biopsy procedures improves the implementation of this technique and benefits patients that undergo this procedure.

## Introduction

Various nephropathies occur frequently in veterinary medicine, and although a great part of them are detected through clinical-laboratory and imaging examinations [1] there are cases mainly glomerular diseases and acute nephropathies where a kidney biopsy can be indicated for the best definition of diagnosis, treatment method, and prognosis [2]. Video-surgeries have become a modality widely used for diagnostic reasons given the difficulty of acquiring a final diagnosis in multiple diseases and the high risk of performing a laparotomy for investigative biopsy sample collection, mainly because this technique is minimally invasive [3–5]. Among the organs biopsied for diagnostic purposes, multiple reports can be found in the literature, both for human and veterinary medicine [1–3, 6, 7].

Ultrasound-guided kidney biopsies are considered the standard method for humans and animals [2, 8], but laparoscopy can be recommended in specific cases, as seen for obese or pediatric human patients, cases of high blood pressure or hemostasis disorders, and for small-sized dogs in veterinary medicine [1, 8, 9]. Various researchers highlight the safety of the laparoscopic procedure [3, 4, 10–12] supported by studies where it was shown to be better than the ultrasound-guided biopsy [7]. However, the inadequate performance of this technique and the lack of skills of the operator enhance the risks for complication due to the kidney biopsy procedure, among other factors [1, 7, 13]. Considering that previous experiences executing a certain technique as one of the determinants of the success rate of this specific procedure for various modalities, including laparoscopy, the use of a learning curve can guarantee that a professional is suitable to perform the defined procedure [14, 15]. Due to the increasing necessity of kidney biopsies, together with the arrival of laparoscopy-guided diagnostic procedures and the required skills to use it, establishing a learning curve for the laparoscopy-guided kidney biopsy procedure can be considered fundamental to help with the training of capable professionals to use this technique, bringing benefits to the patients that will undergo kidney biopsy.

This study aims to establish a learning curve for laparoscopy-guided kidney biopsy procedures in dogs and pigs using the duration of the procedure, quality of the samples, and difficulty level of multiple and subsequent attempts as the indicator variables for perfecting the procedure. The number of attempts necessary for a professional to be considered capable of performing this technique can be determined using this curve, focusing on how many tries were required until the plateau.

## Material and methods

### Animals

The following study was approved by the Veterinary Ethics Committee of UNIFRAN–Universidade de Franca (protocol n° 6439100517).

In total, six dog corpses (3 males and 3 females) (patients who died at the Veterinary Teaching Hospital of the University of Franca—after consent of their tutors) and six pig corpses (females) (research animals donated by the University of Franca) were used. Animals presenting clinical history or examination before death compatible with renal diseases (e.g., azotemia), the ones that deceased more than 48 hours before the procedures (began autolysis), or animals presenting more than 10 kg (this technique is indicated in smaller animals) were excluded from the study.

### Personnel performing the procedures (operators)

Twenty-four veterinarians with surgical knowledge were selected that were without previous experience in laparoscopic procedures or percutaneous kidney biopsies. The operators were randomly divided into pairs per animal, and each one collected 20 fragments, 10 from left and right kidney (also randomly assigned to each pair). The samples were displayed to the operators after the collection in order for them to evaluate the gross characteristics of the biopsy and possibly encourage improvement of subsequent collections.

### Laparoscopy

Animals were placed in dorsal recumbence and fur from the epigastric region to the pubis was trimmed. The pre-procedure preparation of the animal followed the surgical standards required for this procedure [16]. A one-centimeter incision into the cutaneous and muscular layers was made using the scalpel as the entry point of the laparoscope. A 4 mm trocar with an insufflation valve (Endoscopy Surgery, Rio de Janeiro, Brazil) was inserted in the right or left flank, depending on which kidney would be biopsied and the abdomen was inflated with CO2 up to 8 mmHg of intra-abdominal pressure, with an inflation rate of 1 liter per minute.

### Kidney biopsy

Percutaneous kidney biopsies were performed with a semi-automatic Tru-cut^®^ (US BIOPSY, Franklin, USA) needle, 16 G (1.2 mm × 13 cm), guided by laparoscopy. For the biopsy procedure, the animal was laterally recumbent right or left, depending on the kidney accessed. A small cutaneous incision close to the kidney location was made using a scalpel to facilitate the needle puncture.

**Fig 1:**
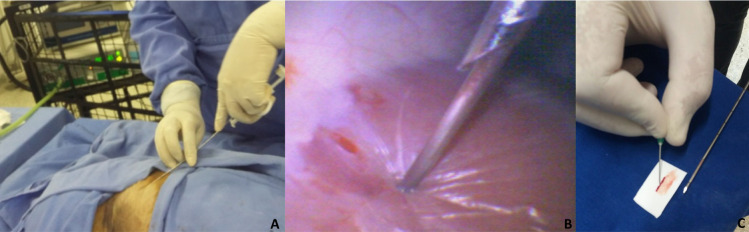
Percutaneous kidney biopsies guided by laparoscopy. (A) Percutaneous targeting of the Tru-cut^®^ needle based on the video image. (B) Insertion of the Tru-cut^®^ needle into the kidney to collect the fragment (video image). (C) Deposition of the renal fragment on designated cardboard cut for storage.

Every operator collected, sequentially, 10 fragments of each kidney from one animal, totaling 20 fragments per operator or 40 per animal since operators were in pairs and each pair worked with one animal. Animals and kidney sides were randomly assigned to operators. Sample collection was guided by laparoscopy to place the Tru-cut^®^ needle in the cortical region of the kidney, which performed the biopsy cut (Fig 1A and 1B). Fragments were retrieved from the inner part of the needle, placed in designated cardboard cuts (Fig 1C), and stored in vials with 10% formalin as described in the literature [17].

### Kidney histological examination

After 24 hours in formalin, samples were processed sequentially with ethanol and xylol and included in a paraffin block from which 3 μm cuts were acquired using the microtome and further stained in Hematoxylin and Eosin on a light microscopy slide. All slides were evaluated using a Universal Zeiss microscope (10X magnification), and the cortical percentage, as well as the number of glomeruli, were counted.

### Operator evaluation

Besides the fragment evaluation, other criteria of the operators were considered. Quantitative criteria included the duration of the collection (time spent from needle introduction to sample retrieval), the duration of the storing process (time spent to place the fragment on cardboard), the total amount of samples obtained, and the mean time taken from one biopsy to another. Qualitative criteria included the difficulty of the procedure according to the operator (3 = difficult; 2 = moderate; 1 = mild) and difficulty as each sample was collected.

### Statistical analysis

Descriptive statistics of the ‘difficulty of the procedure’ were presented with the median value and standard error and statistical calculation was made using post-test Bonferroni analysis. The species effect (dog vs. pig), the cortical percentage in the fragment, the number of glomeruli, and the duration of collection, as well as the storing process, were analyzed through ANOVA (PROC MIXED). Each variable was evaluated separately (fixed independent variables, animal species and attempts; random variables and treatment repetitions), and an appropriate variance structure for each variable was used. When significant interaction happened among the main effects (animal species and attempts), additional analysis of the variance was performed for each treatment.

The data were analyzed to determine whether they met the assumptions required for ANOVA analysis, i.e., normality and homoscedasticity (homogeneous variance), which were verified using Cramer-von Mises and Bartlett tests, respectively. When significant differences when observed among treatments, the means were compared using Tukey’s test with 5% significance (P < 0.05). Statistical analyses were performed using SAS Studio^®^ [18] and Graph Pad Prisma^®^ [19].

## Results

### Animals

From the twelve animal corpses included in this study, 6 were dogs (3 males– 50% and 3 females– 50%) and 6 were pigs (all females—100%). Dog corpses were composed of two Teckel (33.3%), Schnauzer (16.6%), Shih Tzu (16.6%) and mongrel (16.6%). Dog’s mean weight was 8.25±0.6 kg and mean age was 80±26 months. The selected pigs were Large White breed, 2 months years old and mean weight was 9±0.6 kg (Table 1).

**Table 1:** Characteristics of the animals selected in the study.

	Animals	
	Dogs (n = 6)	Pigs (n = 6)
**Weight (kg)**	8.25±0.6	9±0.6
**Age (months)**	80±26	2
**Sex**		
female	3 (50%)	100%
male	3 (50%)	
**Breed**	Teckel (33.3%),	Large White (100%)
	Schnauzer (16.6%),	
	Shih Tzu (16.6%)	
	Mixed Breed (16.6%)	

### Sample quality

**Fig 2:**
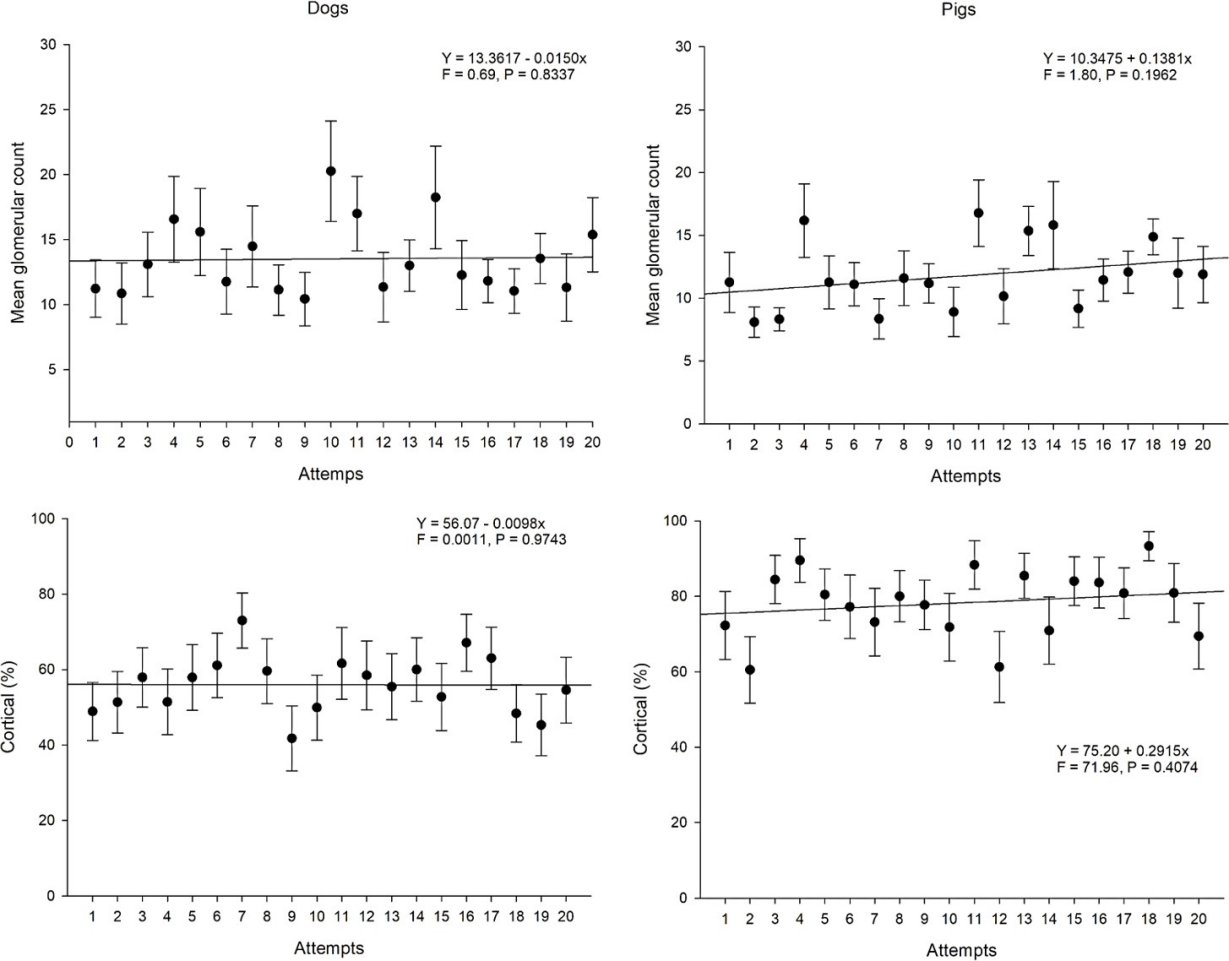
Mean glomerular count and cortical percentage of the kidney fragments collected by the operators throughout the study in the different species (canine and swine). (A) Mean glomerular count throughout the study attempts collected from dogs. (B) Mean glomerular count throughout the study attempts collected from pigs. (C) Mean kidney cortical percentage throughout the study attempts collected from dogs. (D) Mean kidney cortical percentage throughout the study attempts collected from pigs. The Y, F, and P values are indicated in each evaluation and show the presence (or not) of statistical differences of the variables considered for this figure among the attempts and between species.

**Fig 3:**
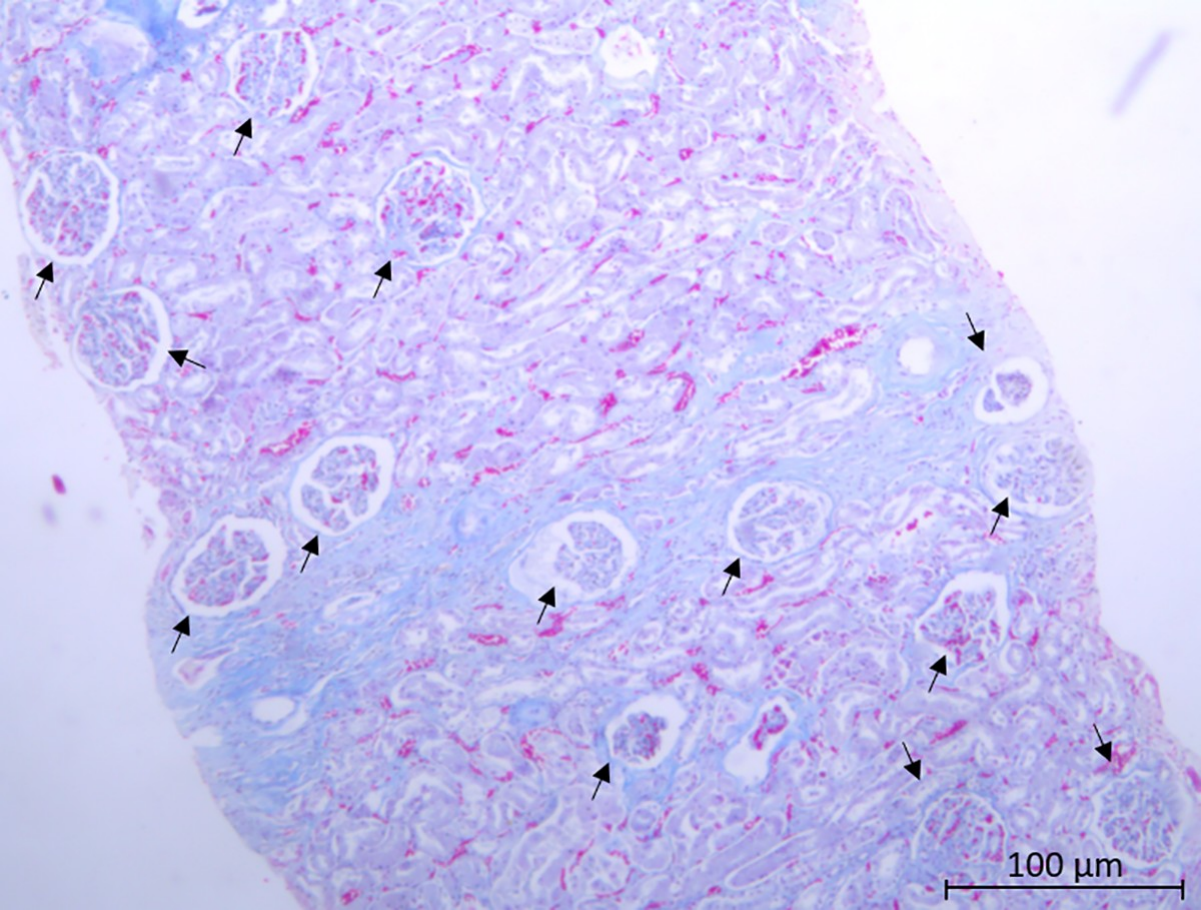
Pig kidney section stained in Hematoxylin and Eosin (HE). Glomerular amount higher than 10 (arrow) in a microscopic field of a cortical region of the kidney (10X magnification).

The mixed regression model showed that the glomerular evaluation and the percentage of renal cortex did not differ among the biopsy attempts in either species (Fig 2). The results showed the mean number of glomeruli to be adequate for a kidney biopsy since the first attempt (Fig 3). Even though there was no significant difference, it was possible to observe a tendency of increasing glomeruli count as the collections took place, as well as an increase in the cortical percentage of the fragment in the swine model. Moreover, when comparing the samples between species, the ones from pigs presented a higher mean cortical percentage of the fragments compared to the dog samples. No large blood vessels were detected in the samples.

### Operators evaluation

**Fig 4:**
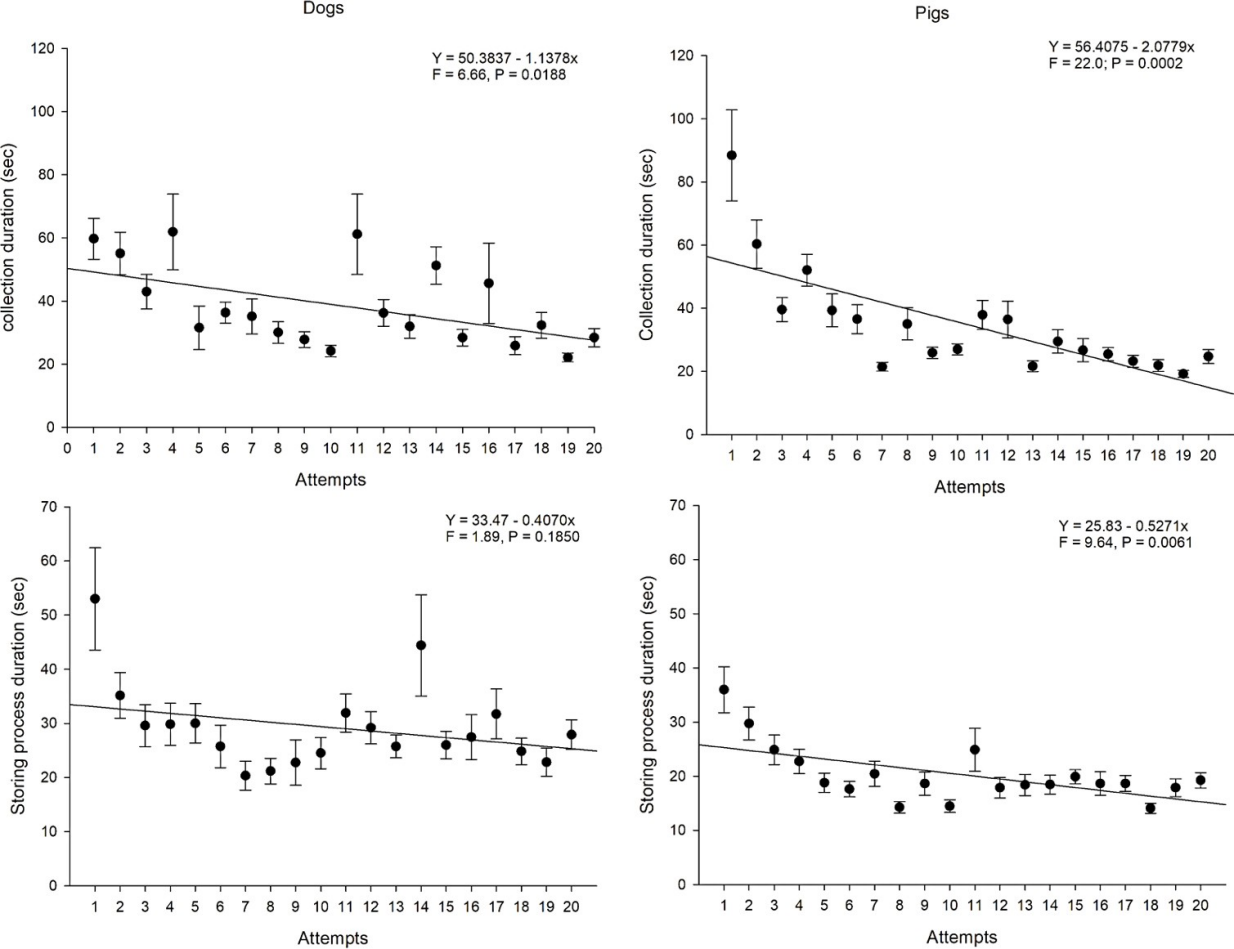
Mean times (seconds) spent in the laparoscopically guided kidney biopsy (duration of collection and storing process) throughout the attempts of the operators in both species (canine and swine). (A) Mean collection duration throughout the study from dogs; B) Mean collection duration throughout the study from pigs. (C) Mean storing process duration throughout the study from dogs. (D) Mean storing process duration throughout the study from pigs. The Y, F, and P values are indicated in each evaluation and show the presence (or not) of statistical differences of the variables considered for this figure among the attempts and between species.

**Fig 5:**
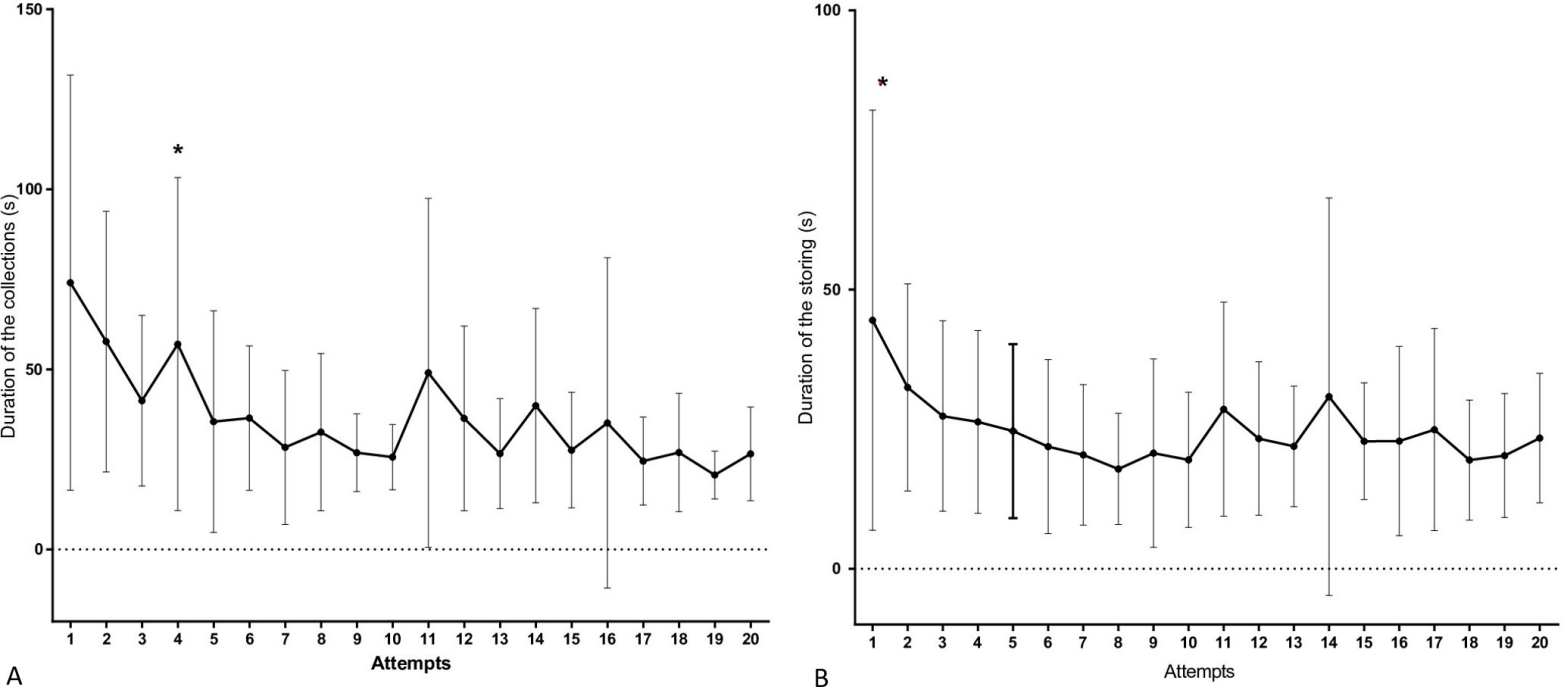
Multivariate analysis of the data. (A) Analysis acquired for the duration of the collections in both species (canine and swine) throughout the attempts in this study. (B) Analysis acquired for the duration of the storing process in both species (canine and swine) throughout the attempts in this study. (*) Moment when subsequent evaluations of this variable are stabilized—plateau (for the duration of collection and storing processes).

The duration of the procedure (both collection and storing process) throughout collection attempts showed statistical differences in both animal models. A clear reduction in the time to perform the procedure was detected as sequential collections took place. A statistical difference was also observed between species, which initially showed the collection procedure to last longer in pigs, but as the sequential collections progressed, the operators working with the pigs showed to be faster than the ones working with the dog models. Nevertheless, operators working with the swine model were faster in placing the samples on the cardboard (storing process) in almost all procedure sequences (Fig 4). It was possible to observe a difference in the collection duration from the fourth attempt on, where the operator started to reach the plateau of the learning curve. Regarding the duration of the storing process, multivariate analysis demonstrated that one attempt is enough for the operator to perform this part of the procedure at similar times for the subsequent attempts (Fig 5).

### Difficulties

**Fig 6:**
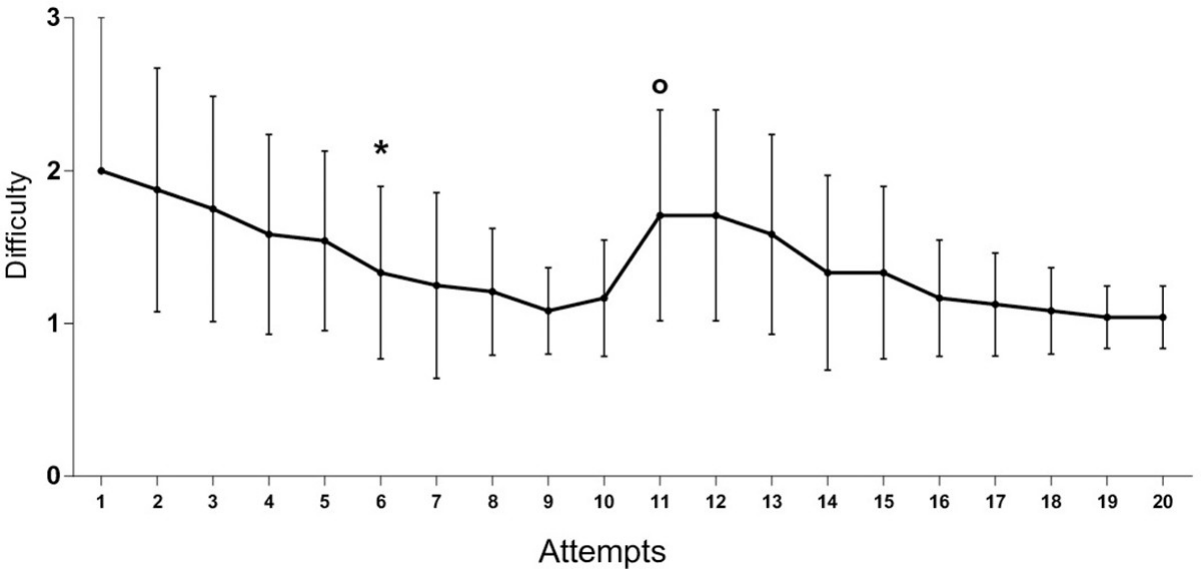
Learning curve for the variable ‘difficulty’. Median and standard error of the difficulty scores (1 = mild; 2 = moderate; 3 = difficult) reported by the 24 operators regarding the laparoscopic-guided kidney biopsy procedure throughout the attempts. (*) Moment when subsequent evaluations of variable ‘difficulty’ were stabilized—plateau; (°) Moment of residual return of the variable ‘difficulty’.

The direction and angulation of the needle observed through the video images were some of the main initial difficulties indicated by the operators. This was supported by the fact that two samples were lost due to the angle of the needle during the procedure in the pig model, which was a consequence of the anatomical proximity of the kidneys to the rib bones, given that two different operators hit this bone with the needle. Similarly, the retrieval of the fragment from the inner part of the needle and its placement onto the cardboard were largely indicated as initially difficult some operators lost the sample or cut the tissue section during this process. Excluding all lost samples, 439/480 fragments (91.5%) were in good enough condition to be evaluated. The overall difficulties of the procedure in each attempt of the collection, according to the operators, are shown in the learning curve pictured in Fig 6. The first collections presented meaningful difficulty and a large oscillation in the opinion of the operators, showing a large standard error. As sequential procedures took place, the standard error became smaller, and at the sixth attempt, the difficulty reported was smaller, and the operators reached the plateau of the learning curve. However, once the side of the collection changed (in the 11th collection of each operator) another almost independent learning curve was produced for this variable (difficulty of the procedure), even though the starting difficulty for the second side was smaller than for the starting side.

## Discussion

Through this study, we showed that good quality tissue samples from kidneys could be obtained using laparoscopically guided techniques since the professional’s first attempt at performing the procedure. However, an improvement in the collection is achieved as the operator repeats it more times faster and less difficult for the operator reaching a plateau in this improvement at the seventh time of collection. The main reason for one to consider the necessity of knowing how and developing enough skills to perform this procedure demonstrated by a learning curve is the requirement of a histopathological diagnosis for several conditions affecting the kidneys, together with the increasing demand for interpretation of these samples originated from small animals [6, 20, 21]. The pig models were added in this study due to their being the model commonly used in human medicine studies [22–25], indicating this study could be beneficial not only for small animal veterinarian practitioners but also for physicians, and with greater importance.

Our results showed that in laparoscopy-guided kidney biopsy procedures performed in dog and pig corpses, the sample quality measured using the cortical percentage of the fragment and the count of glomeruli is considered satisfactory for further histological analysis, independent of the professional experience. This conclusion can be taken using the thresholds established in previous papers, where the cortical percentage of the fragment should be larger than the medullar percentage and the required glomerular count is 5 or 10 glomeruli per fragment [26, 27]. This can be a direct result from seeing the kidney through video during the procedure, which provides the professional with the ability to locate exactly the place where the biopsy needle should puncture the organ, and thus gives a higher control over the process [7, 10, 28].

Contrastingly, the duration of the procedure decreased as the operators made more attempts until it reached a plateau, agreeing with finding from other studies that also focused on establishing learning curves and showed that the practitioner’s increase of experience leads to a decreased procedure time [29–31]. Time shortening in anesthetic and surgical procedures’ duration are beneficial and lower the chances of post-surgery complications [32], specially in patients with nephropathies.

As demonstrated in this study, the duration of the procedure decreased as more attempts were performed by the operators, both in dogs and pigs, and the operators working with pigs acquired the samples faster than the ones working with dogs. This could be a result of the anatomical differences between those species, besides the different location and morphology of this organ in those animal species. Pigs have parallel kidneys arranged close to the abdominal wall, together with an adipose tissue capsule, granting more stability to those when compared to dogs [33]. This particularity might have helped the operators of pigs since the needle was already near the organ once it penetrated the abdominal wall, requiring less manipulation and repositioning of the instrument. No difference was observed between the right and left kidney according to the variables considered in this study. This diverging result when compared to other studies that reported the right kidney to be easier to collect a sample than the left1 could indicate that this observation is more related to the professional/operator performing the technique and not the procedure itself.

According to the literature, when a laparoscopic-guided method is performed by a professional without experience, higher stress and level of difficulty can be detected in its execution, directly leading to complications from the procedure [34]. As showed by our results and also other researches, directing an instrument (e.g., needle) towards the targeted tissue using the help of video-images in a monitor is one of the initial difficulties for professionals working with this technique since the movements are inverted in the image and help of touch is almost null [30, 35, 36]. This factor can also be the explanation for why there is an increase in difficulty to perform this procedure once the contralateral kidney needs to be assessed, even if former collections were performed on the opposite side, since the side change directly affects the display of internal organs showed in the video images and how the operator will assess those images. This shows the importance of training the collection on both sides to guarantee greater skills and safety for the animal during an *in vivo* procedure.

It is important to highlight that the present study included variables pertaining to the procedure itself and the operators, but not the patient’s (the corpses, in our case). However, some of those variables (e.g., blood loss, time of hospitalization, trans- and post-procedure complications, and mortality) could be assessed by the samples’ consistency, size, and the number of arterioles present. Therefore, we were able to predict this procedure to be safe, even in the first biopsies, mainly because no large blood vessels or lesions in vital locations were observed during and after the procedures, which is an observation supported by other studies [37]. Despite this, training on cadavers cannot capture all the possible impacts of an *in vivo* procedure, such as the actual duration of the procedure and intraoperative complications, especially considering the laparoscopic procedure and pneumoperitoneum, which represents a limitation. However, our results show that training on cadavers is an important preliminary step before the procedure is performed on living beings.

In conclusion, we showed that veterinary professionals are capable of collecting good quality samples from kidneys using laparoscopic-guided techniques since their first attempt; however, for a faster procedure with lower difficulties for the operator, at least seven kidney biopsies would be required according to the learning curve assembled in this study.

## Supporting information

S1 Raw data (PDF)

S2 Raw data (PDF)

S3 Raw data (PDF)

## References

[1] Vaden SL. Renal biopsy: methods and interpretation. Vet Clin North Am Small Anim Pract. 2004;34(4):887–908. doi: 10.1016/j.cvsm.2004.03.010 15223207

[2] Vaden SL. Renal biopsy of dogs and cats. Clin Tech Small Anim Pract. 2005;20(1):11–22. doi: 10.1053/j.ctsap.2004.12.003 15822526

[3] Gimenez LF, Micali S, Chen RN, Moore RG, Kavoussi LR, Scheel PJ Jr. Laparoscopic renal biopsy. Kidney Int. 1998;54(2):525–29. doi: 10.1046/j.1523-1755.1998.00006.x 9690219

[4] Andreollo NA, Coelho Neto JDS, Lopes LR, Brandalise NA, Leonardi LS. A laparoscopia no diagnóstico das doenças intra-abdominais. Análise de 168 casos. Rev Assoc Med Bras. 1999;45(1):34–8. doi: 10.1590/s0104-42301999000100008 10436592

[5] Velhote MCP, Tannuriii U, Andrade WC, Filho JGM, Apezzato MLP, Tannur ACA. Videosurgery in infancy and childhood: state of the art. Experience with 1408 procedures in the Instituto da Criança “Pedro de Alcântara”. Rev Col Bras Cir. 2012;39(5):425–35. doi: 10.1590/s0100-69912012000500016 23174797

[6] Grauer GF. Canine glomerulonephritis: new thoughts on proteinuria and treatment. J Small Anim Pract. 2005;46(10):469–78. doi: 10.1111/j.1748-5827.2005.tb00275.x 16245660

[7] Rawlings CA, Diamond H, Howerth EW, Neuwirth L, Canalis C. Diagnostic quality of percutaneous kidney biopsy specimens obtained with laparoscopy versus ultrasound guidance in dogs. J Am Vet Med Assoc. 2003;223(3):317–21. doi: 10.2460/javma.2003.223.317 12906225

[8] Whittier WL. Complications of the percutaneous kidney biopsy. advances in chronic kidney disease. Adv Chronic Kidney Dis. 2012;19(3):179–87. doi: 10.1053/j.ackd.2012.04.003 22578678

[9] Jesus CM, Yamamoto H, Kawano PR, Rodrigo O, Fugita OE. Retroperitoneoscopic renal biopsy in children. International Braz J Urol. 2007;33(4):536–43. doi: 10.1590/s1677-55382007000400013 17767760

[10] Nowicki M, Rychlik A, Nieradka R, Kander M, Depta A, Chrzastowska M. Usefulness of laparoscopy guided renal biopsy in dogs. Pol J Vet Sci. 2010;13(2):363–71. . 20731194

[11] Repetto L, Oderda M, Soria F, Pisano F, Besso L, Pasquale G, et al. Retroperitoneal laparoscopic kidney biopsy: technical tips for a minimally invasive approach. J Endourol. 2011;25(10):1639–42. doi: 10.1089/end.2011.0065 21942797

[12] Park J, Lee J, Lee HB, Jeong SM. Laparoscopic kidney biopsy in dogs: Comparison of cup forceps and core needle biopsy. Vet Surg. 2017;46(2):226–32. doi: 10.1111/vsu.12598 27990651

[13] Lees GE, Cianciolo RE, Clubb FJ Jr. Renal biopsy and pathologic evaluation of glomerular disease. Top Companion Anim Med. 2011;26(3):143–53. doi: 10.1053/j.tcam.2011.04.006 21782145

[14] Santos EG, Netto GPB: Learning curve and iatrogenic injuries in laparoscopic cholecystectomies. Rev Col Bras Cir. 2010;37(3):184–89. doi: 10.1590/s0100-69912010000300005 21079890

[15] Mán E, Németh T, Géczi T, Simonka Z, Lázár G. Learning curve after rapid introduction of laparoscopic appendectomy: Are there any risks in surgical resident participation? World J Emerg Surg. 2016;11(1): 1–8. doi: 10.1186/s13017-016-0074-5 27148395 PMC4855767

[16] Fossum TW, Cho J, Dewey CW, Hayashi K, Huntingford JL, MacPhail CM, et al. Small Animal Surgery. 5th ed, Philadelphia, PA: Elsevier; 2019.

[17] Osborne CA, Bartges JW, Polzin DJ, Lulich JP, Johnston GR, Cox V. Percutaneous needle biopsy of the kidney. Indications, applications, technique, and complications. Vet Clin North Am Small Anim Pract. 1996;26(6):1461–504. doi: 10.1016/s0195-5616(96)50137-3 8911028

[18] SAS Institute. SAS/IML® 14.1 User`s Guide. Cary, NC: SAS Institute Inc.sm. 2015.

[19] GraphPad Prism version 7.00 for Windows, GraphPad Software.

[20] Vaden SL. Glomerular disease. Top Companion Anim Med. 2011;26(3):128–34. doi: 10.1053/j.tcam.2011.04.003 21782143

[21] Cianciolo RE, Brown CA, Mohr FC, Spangler WL, Aresu L, Van der Lugt JJ, et al. Pathologic evaluation of canine renal biopsies: Methods for identifying features that differentiate immune-mediated glomerulonephritides from other categories of glomerular diseases. J Vet Intern Med. 2013;27 Suppl 1:S10–S18. doi: 10.1111/jvim.12226 24635375

[22] Sampaio FJ, Pereira-Sampaio MA, Favorito LA. The pig kidney as an endourologic model: anatomic contribution. J Endourol. 1998;12(1):45–50. doi: 10.1089/end.1998.12.45 9531151

[23] Pereira-Sampaio MA, Favorito LA, Sampaio FJ. Pig kidney: Anatomical relationships between the intrarenal arteries and the kidney collecting system. Applied study for urological research and surgical training. J Urol. 2004;172(5 Pt 1):2077–81. doi: 10.1097/01.ju.0000138085.19352.b5 15540793

[24] Strohmaier WL, Giese A. Improved ex vivo training model for percutaneous renal surgery. Urol Res. 2009;37(2):107–10. doi: 10.1007/s00240-009-0180-x 19277625

[25] Giraud S, Favreau F, Chatauret N, Thuillier R, Maiga S, Hauet T. Contribution of large pig for renal ischemia-reperfusion and transplantation studies: the preclinical model. J Biomed Biotechnol. 2011;2011:1–14. doi: 10.1155/2011/532127 21403881 PMC3051176

[26] Crivellenti LZ, Cianciolo R, Wittum T, Lees GE, Adin CA. Associations of patient characteristics, disease stage, and biopsy technique with the diagnostic quality of core needle renal biopsy specimens from dogs with suspected kidney disease. J Am Vet Med Assoc. 2018;252(1):67–74. doi: 10.2460/javma.252.1.67 29244598

[27] Manashirova M, Pressler BM, Gelb HR, et al. Pilot evaluation of a vacuum-assisted biopsy instrument for percutaneous renal biopsy in dogs. J Am Anim Hosp Assoc. 2011;47(6):391–98. doi: 10.5326/JAAHA-MS-5637 22058345

[28] Nowicki M, Depta A: Biopsja nerek u psów i kotów. Medycyna Wet. 2001;57(2): 97–101.

[29] Shetye KR, Kavoussi LR, Ramakumar S, Fugita OE, Jarrett TW. Laparoscopic renal biopsy: A 9-year experience. BJU Int. 2003;91(9):817–20. doi: 10.1046/j.1464-410x.2003.04243.x 12780840

[30] Fransson BA. Advances in laparoscopic skills training and management. Vet Clin North Am Small Anim Pract. 2016;46(1):1–12. doi: 10.1016/j.cvsm.2015.08.002 26396055

[31] Guo J, Zeng Z, Cao R, Hu J. Intraoperative serious complications of laparoscopic urological surgeries: A single institute experience of 4,380 procedures. Int Braz J Urol. 2019;45(4):739–46. doi: 10.1590/S1677-5538.IBJU.2018.0601 31063283 PMC6837612

[32] Cheng H, Clymer JW, Po-Han Chen B, et al. Prolonged operative duration is associated with complications: A systematic review and meta-analysis. J Surg Res. 2018; 229:134–44. doi: 10.1016/j.jss.2018.03.022 29936980

[33] Dyce K, Sack W, Wensing CJG. Textbook of Veterinary Anatomy, 4th ed, St Louis, MO: Saunders Elsevier, 2010.

[34] Berguer R, Smith WD, Chung YH. Performing laparoscopic surgery is significantly more stressful for the surgeon than open surgery. Surg Endosc. 2001;15(10):1204–07. doi: 10.1007/s004640080030 11727101

[35] Jeong J, Ko J, Lim H, Kweon OK, Kim WH. Retroperitoneal laparoscopy in dogs: access technique, working space, and surgical anatomy. Vet Surg. 2016;45(S1):O102–O110. doi: 10.1111/vsu.12571 27731512 PMC5129584

[36] Satava RM. Emerging technologies for surgery in the 21st century. Arch Surg. 1999;134(11):1197–202. doi: 10.1001/archsurg.134.11.1197 10555633

[37] Khan N, Abboudi H, Khan MS, Dasgupta P, Ahmed K. Measuring the surgical ’learning curve’: Methods, variables and competency. BJU Int. 2014;113(3):504–8. doi: 10.1111/bju.12197 23819461

