# Supplementary material for: Learning curve for the laparoscopy-guided kidney biopsy procedure in small corpses of dogs and pigs
Source: PLoS One. 2021 Sep 27;16(9):e0257653. doi: 10.1371/journal.pone.0257653 (PMC8475998; doi:10.1371/journal.pone.0257653)
Supplement: S1 Raw data — (PDF) [file pone.0257653.s001.pdf]

Obs	ANI	TEN	RP	Y
1	c	1	1	70
2	c	2	1	95
3	c	3	1	95
4	c	4	1	90
5	c	5	1	95
6	c	6	1	95
7	c	7	1	95
8	c	8	1	95
9	c	9	1	45
10	c	10	1	95
11	c	11	1	95
12	c	12	1	95
13	c	13	1	95
14	c	14	1	95
15	c	15	1	95
16	c	16	1	95
17	c	17	1	95
18	c	18	1	85
19	c	19	1	0
20	c	20	1	95
21	c	1	2	60
22	c	2	2	90
23	c	3	2	40
24	c	4	2	60
25	c	5	2	95
26	c	6	2	80
27	c	7	2	95
28	c	8	2	95
29	c	9	2	95
30	c	10	2	95
31	c	11	2	95
32	c	12	2	85
33	c	13	2	95
34	c	14	2	95
35	c	15	2	90
36	c	16	2	85
37	c	17	2	70
38	c	18	2	95
39	c	19	2	50
40	c	20	2	95
41	c	1	3	0

Obs	ANI	TEN	RP	Y
**42**	c	2	3	95
**43**	c	3	3	0
**44**	c	4	3	95
**45**	c	5	3	95
**46**	c	6	3	0
**47**	c	7	3	95
**48**	c	8	3	0
**49**	c	9	3	0
**50**	c	10	3	50
**51**	c	11	3	100
**52**	c	12	3	100
**53**	c	13	3	5
**54**	c	14	3	30
**55**	c	15	3	95
**56**	c	16	3	40
**57**	c	17	3	95
**58**	c	18	3	90
**59**	c	19	3	30
**60**	c	20	3	20
**61**	c	1	4	45
**62**	c	2	4	60
**63**	c	3	4	60
**64**	c	4	4	0
**65**	c	5	4	10
**66**	c	6	4	0
**67**	c	7	4	45
**68**	c	8	4	95
**69**	c	9	4	0
**70**	c	10	4	40
**71**	c	11	4	95
**72**	c	12	4	90
**73**	c	13	4	0
**74**	c	14	4	95
**75**	c	15	4	15
**76**	c	16	4	100
**77**	c	17	4	60
**78**	c	18	4	50
**79**	c	19	4	50
**80**	c	20	4	10
**81**	c	1	5	78
**82**	c	2	5	95

Obs	ANI	TEN	RP	Y
83	c	3	5	95
84	c	4	5	97
85	c	5	5	95
86	c	6	5	78
87	c	7	5	78
88	c	8	5	78
89	c	9	5	78
90	c	10	5	95
91	c	11	5	98
92	c	12	5	78
93	c	13	5	78
94	c	14	5	78
95	c	15	5	78
96	c	16	5	78
97	c	17	5	78
98	c	18	5	78
99	c	19	5	78
100	c	20	5	78
101	c	1	6	78
102	c	2	6	78
103	c	3	6	78
104	c	4	6	78
105	c	5	6	0
106	c	6	6	78
107	c	7	6	78
108	c	8	6	95
109	c	9	6	78
110	c	10	6	0
111	c	11	6	95
112	c	12	6	95
113	c	13	6	95
114	c	14	6	12
115	c	15	6	12
116	c	16	6	0
117	c	17	6	0
118	c	18	6	2
119	c	19	6	95
120	c	20	6	95
121	c	1	7	12
122	c	2	7	0
123	c	3	7	0

Obs	ANI	TEN	RP	Y
**124**	c	4	7	0
**125**	c	5	7	0
**126**	c	6	7	95
**127**	c	7	7	95
**128**	c	8	7	12
**129**	c	9	7	0
**130**	c	10	7	12
**131**	c	11	7	0
**132**	c	12	7	0
**133**	c	13	7	0
**134**	c	14	7	95
**135**	c	15	7	0
**136**	c	16	7	95
**137**	c	17	7	95
**138**	c	18	7	12
**139**	c	19	7	0
**140**	c	20	7	12
**141**	c	1	8	0
**142**	c	2	8	0
**143**	c	3	8	4
**144**	c	4	8	12
**145**	c	5	8	95
**146**	c	6	8	12
**147**	c	7	8	0
**148**	c	8	8	0
**149**	c	9	8	10
**150**	c	10	8	10
**151**	c	11	8	0
**152**	c	12	8	0
**153**	c	13	8	0
**154**	c	14	8	0
**155**	c	15	8	95
**156**	c	16	8	95
**157**	c	17	8	0
**158**	c	18	8	0
**159**	c	19	8	95
**160**	c	20	8	95
**161**	c	1	9	95
**162**	c	2	9	12
**163**	c	3	9	95
**164**	c	4	9	95

Obs	ANI	TEN	RP	Y
**165**	c	5	9	12
**166**	c	6	9	95
**167**	c	7	9	95
**168**	c	8	9	95
**169**	c	9	9	95
**170**	c	10	9	2
**171**	c	11	9	0
**172**	c	12	9	0
**173**	c	13	9	92
**174**	c	14	9	0
**175**	c	15	9	0
**176**	c	16	9	0
**177**	c	17	9	0
**178**	c	18	9	0
**179**	c	19	9	0
**180**	c	20	9	0
**181**	c	4	10	0
**182**	c	5	10	100
**183**	c	6	10	0
**184**	c	7	10	0
**185**	c	8	10	50
**186**	c	9	10	0
**187**	c	10	10	0
**188**	c	11	10	.
**189**	c	13	10	.
**190**	c	15	10	.
**191**	c	17	10	.
**192**	c	19	10	.
**193**	c	1	11	0
**194**	c	2	11	0
**195**	c	3	11	100
**196**	c	4	11	0
**197**	c	6	11	100
**198**	c	7	11	100
**199**	c	8	11	0
**200**	c	9	11	0
**201**	c	10	11	100
**202**	c	11	11	100
**203**	c	12	11	100
**204**	c	13	11	100
**205**	c	14	11	100

Obs	ANI	TEN	RP	Y
206	c	15	11	100
207	c	16	11	50
208	c	17	11	100
209	c	18	11	80
210	c	19	11	100
211	c	20	11	100
212	c	1	12	100
213	c	2	12	40
214	c	3	12	70
215	c	4	12	90
216	c	5	12	40
217	c	6	12	100
218	c	7	12	100
219	c	8	12	100
220	c	9	12	100
221	c	10	12	100
222	c	11	12	0
223	c	12	12	0
224	c	13	12	50
225	c	15	12	0
226	c	16	12	100
227	c	17	12	100
228	c	18	12	40
229	c	19	12	0
230	c	20	12	0
231	s	1	13	0
232	s	2	13	100
233	s	3	13	100
234	s	5	13	100
235	s	6	13	100
236	s	7	13	0
237	s	10	13	95
238	s	12	13	95
239	s	13	13	95
240	s	14	13	95
241	s	15	13	100
242	s	16	13	95
243	s	17	13	100
244	s	18	13	100
245	s	19	13	95
246	s	20	13	100

Obs	ANI	TEN	RP	Y
**247**	s	1	14	100
**248**	s	3	14	100
**249**	s	4	14	100
**250**	s	5	14	100
**251**	s	8	14	100
**252**	s	9	14	100
**253**	s	10	14	0
**254**	s	11	14	100
**255**	s	12	14	100
**256**	s	13	14	60
**257**	s	14	14	100
**258**	s	15	14	100
**259**	s	16	14	95
**260**	s	17	14	50
**261**	s	18	14	100
**262**	s	19	14	100
**263**	s	20	14	0
**264**	s	1	15	95
**265**	s	2	15	0
**266**	s	3	15	100
**267**	s	4	15	100
**268**	s	6	15	100
**269**	s	7	15	100
**270**	s	8	15	100
**271**	s	9	15	100
**272**	s	10	15	100
**273**	s	11	15	100
**274**	s	12	15	0
**275**	s	13	15	0
**276**	s	14	15	100
**277**	s	16	15	100
**278**	s	17	15	100
**279**	s	18	15	100
**280**	s	19	15	0
**281**	s	20	15	30
**282**	s	1	16	100
**283**	s	2	16	100
**284**	s	3	16	70
**285**	s	4	16	100
**286**	s	5	16	30
**287**	s	6	16	100

Obs	ANI	TEN	RP	Y
**288**	s	7	16	100
**289**	s	8	16	100
**290**	s	9	16	50
**291**	s	10	16	100
**292**	s	12	16	0
**293**	s	14	16	0
**294**	s	15	16	50
**295**	s	16	16	100
**296**	s	17	16	100
**297**	s	18	16	100
**298**	s	19	16	100
**299**	s	1	17	0
**300**	s	2	17	0
**301**	s	3	17	100
**302**	s	4	17	100
**303**	s	5	17	60
**304**	s	6	17	0
**305**	s	7	17	100
**306**	s	9	17	100
**307**	s	10	17	100
**308**	s	11	17	100
**309**	s	12	17	40
**310**	s	13	17	95
**311**	s	14	17	100
**312**	s	15	17	0
**313**	s	16	17	100
**314**	s	17	17	95
**315**	s	18	17	40
**316**	s	19	17	100
**317**	s	20	17	95
**318**	s	1	18	100
**319**	s	2	18	0
**320**	s	3	18	0
**321**	s	4	18	0
**322**	s	5	18	100
**323**	s	6	18	100
**324**	s	7	18	0
**325**	s	8	18	0
**326**	s	9	18	100
**327**	s	10	18	100
**328**	s	11	18	0

Obs	ANI	TEN	RP	Y
**329**	s	12	18	0
**330**	s	13	18	100
**331**	s	14	18	0
**332**	s	15	18	100
**333**	s	16	18	0
**334**	s	17	18	0
**335**	s	18	18	100
**336**	s	19	18	0
**337**	s	1	19	100
**338**	s	2	19	100
**339**	s	4	19	100
**340**	s	5	19	100
**341**	s	6	19	0
**342**	s	7	19	100
**343**	s	8	19	100
**344**	s	9	19	100
**345**	s	11	19	100
**346**	s	12	19	100
**347**	s	13	19	100
**348**	s	16	19	100
**349**	s	17	19	100
**350**	s	19	19	100
**351**	s	1	20	100
**352**	s	2	20	50
**353**	s	3	20	90
**354**	s	4	20	95
**355**	s	5	20	100
**356**	s	7	20	100
**357**	s	8	20	50
**358**	s	9	20	60
**359**	s	10	20	95
**360**	s	12	20	0
**361**	s	13	20	95
**362**	s	14	20	95
**363**	s	15	20	95
**364**	s	16	20	100
**365**	s	17	20	95
**366**	s	19	20	100
**367**	s	20	20	100
**368**	s	1	21	100
**369**	s	3	21	100

Obs	ANI	TEN	RP	Y
370	s	4	21	100
371	s	5	21	0
372	s	7	21	100
373	s	8	21	100
374	s	9	21	100
375	s	10	21	100
376	s	11	21	100
377	s	12	21	100
378	s	13	21	95
379	s	14	21	100
380	s	15	21	100
381	s	16	21	30
382	s	17	21	30
383	s	19	21	100
384	s	20	21	100
385	s	1	22	100
386	s	2	22	60
387	s	3	22	100
388	s	4	22	100
389	s	5	22	100
390	s	6	22	95
391	s	7	22	5
392	s	8	22	100
393	s	9	22	0
394	s	10	22	0
395	s	11	22	95
396	s	12	22	100
397	s	13	22	100
398	s	14	22	0
399	s	15	22	95
400	s	17	22	100
401	s	18	22	100
402	s	19	22	95
403	s	20	22	0
404	s	1	23	0
405	s	2	23	100
406	s	4	23	95
407	s	5	23	100
408	s	6	23	100
409	s	7	23	100
410	s	8	23	50

Obs	ANI	TEN	RP	Y
**411**	s	9	23	95
**412**	s	10	23	100
**413**	s	11	23	100
**414**	s	12	23	100
**415**	s	13	23	100
**416**	s	14	23	90
**417**	s	15	23	100
**418**	s	16	23	100
**419**	s	17	23	100
**420**	s	18	23	100
**421**	s	20	23	100
**422**	s	2	24	95
**423**	s	4	24	95
**424**	s	5	24	95
**425**	s	6	24	100
**426**	s	7	24	100
**427**	s	8	24	100
**428**	s	9	24	50
**429**	s	10	24	0
**430**	s	11	24	100
**431**	s	12	24	100
**432**	s	13	24	100
**433**	s	14	24	100
**434**	s	15	24	100
**435**	s	16	24	100
**436**	s	17	24	100
**437**	s	18	24	100
**438**	s	19	24	100
**439**	s	20	24	100

Obs	ANI	TEN	RP	Y
1	c	1	1	70
2	c	2	1	95
3	c	3	1	95
4	c	4	1	90
5	c	5	1	95
6	c	6	1	95
7	c	7	1	95
8	c	8	1	95
9	c	9	1	45
10	c	10	1	95
11	c	11	1	95
12	c	12	1	95
13	c	13	1	95
14	c	14	1	95
15	c	15	1	95
16	c	16	1	95
17	c	17	1	95
18	c	18	1	85
19	c	19	1	0
20	c	20	1	95
21	c	1	2	60
22	c	2	2	90
23	c	3	2	40
24	c	4	2	60
25	c	5	2	95
26	c	6	2	80
27	c	7	2	95
28	c	8	2	95
29	c	9	2	95
30	c	10	2	95
31	c	11	2	95
32	c	12	2	85
33	c	13	2	95
34	c	14	2	95
35	c	15	2	90
36	c	16	2	85
37	c	17	2	70
38	c	18	2	95
39	c	19	2	50
40	c	20	2	95
41	c	1	3	0

Obs	ANI	TEN	RP	Y
**42**	c	2	3	95
**43**	c	3	3	0
**44**	c	4	3	95
**45**	c	5	3	95
**46**	c	6	3	0
**47**	c	7	3	95
**48**	c	8	3	0
**49**	c	9	3	0
**50**	c	10	3	50
**51**	c	11	3	100
**52**	c	12	3	100
**53**	c	13	3	5
**54**	c	14	3	30
**55**	c	15	3	95
**56**	c	16	3	40
**57**	c	17	3	95
**58**	c	18	3	90
**59**	c	19	3	30
**60**	c	20	3	20
**61**	c	1	4	45
**62**	c	2	4	60
**63**	c	3	4	60
**64**	c	4	4	0
**65**	c	5	4	10
**66**	c	6	4	0
**67**	c	7	4	45
**68**	c	8	4	95
**69**	c	9	4	0
**70**	c	10	4	40
**71**	c	11	4	95
**72**	c	12	4	90
**73**	c	13	4	0
**74**	c	14	4	95
**75**	c	15	4	15
**76**	c	16	4	100
**77**	c	17	4	60
**78**	c	18	4	50
**79**	c	19	4	50
**80**	c	20	4	10
**81**	c	1	5	78
**82**	c	2	5	95

Obs	ANI	TEN	RP	Y
83	c	3	5	95
84	c	4	5	97
85	c	5	5	95
86	c	6	5	78
87	c	7	5	78
88	c	8	5	78
89	c	9	5	78
90	c	10	5	95
91	c	11	5	98
92	c	12	5	78
93	c	13	5	78
94	c	14	5	78
95	c	15	5	78
96	c	16	5	78
97	c	17	5	78
98	c	18	5	78
99	c	19	5	78
100	c	20	5	78
101	c	1	6	78
102	c	2	6	78
103	c	3	6	78
104	c	4	6	78
105	c	5	6	0
106	c	6	6	78
107	c	7	6	78
108	c	8	6	95
109	c	9	6	78
110	c	10	6	0
111	c	11	6	95
112	c	12	6	95
113	c	13	6	95
114	c	14	6	12
115	c	15	6	12
116	c	16	6	0
117	c	17	6	0
118	c	18	6	2
119	c	19	6	95
120	c	20	6	95
121	c	1	7	12
122	c	2	7	0
123	c	3	7	0

Obs	ANI	TEN	RP	Y
**124**	c	4	7	0
**125**	c	5	7	0
**126**	c	6	7	95
**127**	c	7	7	95
**128**	c	8	7	12
**129**	c	9	7	0
**130**	c	10	7	12
**131**	c	11	7	0
**132**	c	12	7	0
**133**	c	13	7	0
**134**	c	14	7	95
**135**	c	15	7	0
**136**	c	16	7	95
**137**	c	17	7	95
**138**	c	18	7	12
**139**	c	19	7	0
**140**	c	20	7	12
**141**	c	1	8	0
**142**	c	2	8	0
**143**	c	3	8	4
**144**	c	4	8	12
**145**	c	5	8	95
**146**	c	6	8	12
**147**	c	7	8	0
**148**	c	8	8	0
**149**	c	9	8	10
**150**	c	10	8	10
**151**	c	11	8	0
**152**	c	12	8	0
**153**	c	13	8	0
**154**	c	14	8	0
**155**	c	15	8	95
**156**	c	16	8	95
**157**	c	17	8	0
**158**	c	18	8	0
**159**	c	19	8	95
**160**	c	20	8	95
**161**	c	1	9	95
**162**	c	2	9	12
**163**	c	3	9	95
**164**	c	4	9	95

Obs	ANI	TEN	RP	Y
165	c	5	9	12
166	c	6	9	95
167	c	7	9	95
168	c	8	9	95
169	c	9	9	95
170	c	10	9	2
171	c	11	9	0
172	c	12	9	0
173	c	13	9	92
174	c	14	9	0
175	c	15	9	0
176	c	16	9	0
177	c	17	9	0
178	c	18	9	0
179	c	19	9	0
180	c	20	9	0
181	c	4	10	0
182	c	5	10	100
183	c	6	10	0
184	c	7	10	0
185	c	8	10	50
186	c	9	10	0
187	c	10	10	0
188	c	11	10	.
189	c	13	10	.
190	c	15	10	.
191	c	17	10	.
192	c	19	10	.
193	c	1	11	0
194	c	2	11	0
195	c	3	11	100
196	c	4	11	0
197	c	6	11	100
198	c	7	11	100
199	c	8	11	0
200	c	9	11	0
201	c	10	11	100
202	c	11	11	100
203	c	12	11	100
204	c	13	11	100
205	c	14	11	100

Obs	ANI	TEN	RP	Y
206	c	15	11	100
207	c	16	11	50
208	c	17	11	100
209	c	18	11	80
210	c	19	11	100
211	c	20	11	100
212	c	1	12	100
213	c	2	12	40
214	c	3	12	70
215	c	4	12	90
216	c	5	12	40
217	c	6	12	100
218	c	7	12	100
219	c	8	12	100
220	c	9	12	100
221	c	10	12	100
222	c	11	12	0
223	c	12	12	0
224	c	13	12	50
225	c	15	12	0
226	c	16	12	100
227	c	17	12	100
228	c	18	12	40
229	c	19	12	0
230	c	20	12	0
231	s	1	13	0
232	s	2	13	100
233	s	3	13	100
234	s	5	13	100
235	s	6	13	100
236	s	7	13	0
237	s	10	13	95
238	s	12	13	95
239	s	13	13	95
240	s	14	13	95
241	s	15	13	100
242	s	16	13	95
243	s	17	13	100
244	s	18	13	100
245	s	19	13	95
246	s	20	13	100

Obs	ANI	TEN	RP	Y
**247**	s	1	14	100
**248**	s	3	14	100
**249**	s	4	14	100
**250**	s	5	14	100
**251**	s	8	14	100
**252**	s	9	14	100
**253**	s	10	14	0
**254**	s	11	14	100
**255**	s	12	14	100
**256**	s	13	14	60
**257**	s	14	14	100
**258**	s	15	14	100
**259**	s	16	14	95
**260**	s	17	14	50
**261**	s	18	14	100
**262**	s	19	14	100
**263**	s	20	14	0
**264**	s	1	15	95
**265**	s	2	15	0
**266**	s	3	15	100
**267**	s	4	15	100
**268**	s	6	15	100
**269**	s	7	15	100
**270**	s	8	15	100
**271**	s	9	15	100
**272**	s	10	15	100
**273**	s	11	15	100
**274**	s	12	15	0
**275**	s	13	15	0
**276**	s	14	15	100
**277**	s	16	15	100
**278**	s	17	15	100
**279**	s	18	15	100
**280**	s	19	15	0
**281**	s	20	15	30
**282**	s	1	16	100
**283**	s	2	16	100
**284**	s	3	16	70
**285**	s	4	16	100
**286**	s	5	16	30
**287**	s	6	16	100

Obs	ANI	TEN	RP	Y
288	s	7	16	100
289	s	8	16	100
290	s	9	16	50
291	s	10	16	100
292	s	12	16	0
293	s	14	16	0
294	s	15	16	50
295	s	16	16	100
296	s	17	16	100
297	s	18	16	100
298	s	19	16	100
299	s	1	17	0
300	s	2	17	0
301	s	3	17	100
302	s	4	17	100
303	s	5	17	60
304	s	6	17	0
305	s	7	17	100
306	s	9	17	100
307	s	10	17	100
308	s	11	17	100
309	s	12	17	40
310	s	13	17	95
311	s	14	17	100
312	s	15	17	0
313	s	16	17	100
314	s	17	17	95
315	s	18	17	40
316	s	19	17	100
317	s	20	17	95
318	s	1	18	100
319	s	2	18	0
320	s	3	18	0
321	s	4	18	0
322	s	5	18	100
323	s	6	18	100
324	s	7	18	0
325	s	8	18	0
326	s	9	18	100
327	s	10	18	100
328	s	11	18	0

Obs	ANI	TEN	RP	Y
**329**	s	12	18	0
**330**	s	13	18	100
**331**	s	14	18	0
**332**	s	15	18	100
**333**	s	16	18	0
**334**	s	17	18	0
**335**	s	18	18	100
**336**	s	19	18	0
**337**	s	1	19	100
**338**	s	2	19	100
**339**	s	4	19	100
**340**	s	5	19	100
**341**	s	6	19	0
**342**	s	7	19	100
**343**	s	8	19	100
**344**	s	9	19	100
**345**	s	11	19	100
**346**	s	12	19	100
**347**	s	13	19	100
**348**	s	16	19	100
**349**	s	17	19	100
**350**	s	19	19	100
**351**	s	1	20	100
**352**	s	2	20	50
**353**	s	3	20	90
**354**	s	4	20	95
**355**	s	5	20	100
**356**	s	7	20	100
**357**	s	8	20	50
**358**	s	9	20	60
**359**	s	10	20	95
**360**	s	12	20	0
**361**	s	13	20	95
**362**	s	14	20	95
**363**	s	15	20	95
**364**	s	16	20	100
**365**	s	17	20	95
**366**	s	19	20	100
**367**	s	20	20	100
**368**	s	1	21	100
**369**	s	3	21	100

Obs	ANI	TEN	RP	Y
**370**	s	4	21	100
**371**	s	5	21	0
**372**	s	7	21	100
**373**	s	8	21	100
**374**	s	9	21	100
**375**	s	10	21	100
**376**	s	11	21	100
**377**	s	12	21	100
**378**	s	13	21	95
**379**	s	14	21	100
**380**	s	15	21	100
**381**	s	16	21	30
**382**	s	17	21	30
**383**	s	19	21	100
**384**	s	20	21	100
**385**	s	1	22	100
**386**	s	2	22	60
**387**	s	3	22	100
**388**	s	4	22	100
**389**	s	5	22	100
**390**	s	6	22	95
**391**	s	7	22	5
**392**	s	8	22	100
**393**	s	9	22	0
**394**	s	10	22	0
**395**	s	11	22	95
**396**	s	12	22	100
**397**	s	13	22	100
**398**	s	14	22	0
**399**	s	15	22	95
**400**	s	17	22	100
**401**	s	18	22	100
**402**	s	19	22	95
**403**	s	20	22	0
**404**	s	1	23	0
**405**	s	2	23	100
**406**	s	4	23	95
**407**	s	5	23	100
**408**	s	6	23	100
**409**	s	7	23	100
**410**	s	8	23	50

Obs	ANI	TEN	RP	Y
**411**	s	9	23	95
**412**	s	10	23	100
**413**	s	11	23	100
**414**	s	12	23	100
**415**	s	13	23	100
**416**	s	14	23	90
**417**	s	15	23	100
**418**	s	16	23	100
**419**	s	17	23	100
**420**	s	18	23	100
**421**	s	20	23	100
**422**	s	2	24	95
**423**	s	4	24	95
**424**	s	5	24	95
**425**	s	6	24	100
**426**	s	7	24	100
**427**	s	8	24	100
**428**	s	9	24	50
**429**	s	10	24	0
**430**	s	11	24	100
**431**	s	12	24	100
**432**	s	13	24	100
**433**	s	14	24	100
**434**	s	15	24	100
**435**	s	16	24	100
**436**	s	17	24	100
**437**	s	18	24	100
**438**	s	19	24	100
**439**	s	20	24	100

Obs	ANI	TEN	RP	Y
1	c	1	1	70
2	c	2	1	95
3	c	3	1	95
4	c	4	1	90
5	c	5	1	95
6	c	6	1	95
7	c	7	1	95
8	c	8	1	95
9	c	9	1	45
10	c	10	1	95
11	c	11	1	95
12	c	12	1	95
13	c	13	1	95
14	c	14	1	95
15	c	15	1	95
16	c	16	1	95
17	c	17	1	95
18	c	18	1	85
19	c	19	1	0
20	c	20	1	95
21	c	1	2	60
22	c	2	2	90
23	c	3	2	40
24	c	4	2	60
25	c	5	2	95
26	c	6	2	80
27	c	7	2	95
28	c	8	2	95
29	c	9	2	95
30	c	10	2	95
31	c	11	2	95
32	c	12	2	85
33	c	13	2	95
34	c	14	2	95
35	c	15	2	90
36	c	16	2	85
37	c	17	2	70
38	c	18	2	95
39	c	19	2	50
40	c	20	2	95
41	c	1	3	0

Obs	ANI	TEN	RP	Y
**42**	c	2	3	95
**43**	c	3	3	0
**44**	c	4	3	95
**45**	c	5	3	95
**46**	c	6	3	0
**47**	c	7	3	95
**48**	c	8	3	0
**49**	c	9	3	0
**50**	c	10	3	50
**51**	c	11	3	100
**52**	c	12	3	100
**53**	c	13	3	5
**54**	c	14	3	30
**55**	c	15	3	95
**56**	c	16	3	40
**57**	c	17	3	95
**58**	c	18	3	90
**59**	c	19	3	30
**60**	c	20	3	20
**61**	c	1	4	45
**62**	c	2	4	60
**63**	c	3	4	60
**64**	c	4	4	0
**65**	c	5	4	10
**66**	c	6	4	0
**67**	c	7	4	45
**68**	c	8	4	95
**69**	c	9	4	0
**70**	c	10	4	40
**71**	c	11	4	95
**72**	c	12	4	90
**73**	c	13	4	0
**74**	c	14	4	95
**75**	c	15	4	15
**76**	c	16	4	100
**77**	c	17	4	60
**78**	c	18	4	50
**79**	c	19	4	50
**80**	c	20	4	10
**81**	c	1	5	78
**82**	c	2	5	95

Obs	ANI	TEN	RP	Y
83	c	3	5	95
84	c	4	5	97
85	c	5	5	95
86	c	6	5	78
87	c	7	5	78
88	c	8	5	78
89	c	9	5	78
90	c	10	5	95
91	c	11	5	98
92	c	12	5	78
93	c	13	5	78
94	c	14	5	78
95	c	15	5	78
96	c	16	5	78
97	c	17	5	78
98	c	18	5	78
99	c	19	5	78
100	c	20	5	78
101	c	1	6	78
102	c	2	6	78
103	c	3	6	78
104	c	4	6	78
105	c	5	6	0
106	c	6	6	78
107	c	7	6	78
108	c	8	6	95
109	c	9	6	78
110	c	10	6	0
111	c	11	6	95
112	c	12	6	95
113	c	13	6	95
114	c	14	6	12
115	c	15	6	12
116	c	16	6	0
117	c	17	6	0
118	c	18	6	2
119	c	19	6	95
120	c	20	6	95
121	c	1	7	12
122	c	2	7	0
123	c	3	7	0

Obs	ANI	TEN	RP	Y
**124**	c	4	7	0
**125**	c	5	7	0
**126**	c	6	7	95
**127**	c	7	7	95
**128**	c	8	7	12
**129**	c	9	7	0
**130**	c	10	7	12
**131**	c	11	7	0
**132**	c	12	7	0
**133**	c	13	7	0
**134**	c	14	7	95
**135**	c	15	7	0
**136**	c	16	7	95
**137**	c	17	7	95
**138**	c	18	7	12
**139**	c	19	7	0
**140**	c	20	7	12
**141**	c	1	8	0
**142**	c	2	8	0
**143**	c	3	8	4
**144**	c	4	8	12
**145**	c	5	8	95
**146**	c	6	8	12
**147**	c	7	8	0
**148**	c	8	8	0
**149**	c	9	8	10
**150**	c	10	8	10
**151**	c	11	8	0
**152**	c	12	8	0
**153**	c	13	8	0
**154**	c	14	8	0
**155**	c	15	8	95
**156**	c	16	8	95
**157**	c	17	8	0
**158**	c	18	8	0
**159**	c	19	8	95
**160**	c	20	8	95
**161**	c	1	9	95
**162**	c	2	9	12
**163**	c	3	9	95
**164**	c	4	9	95

Obs	ANI	TEN	RP	Y
**165**	c	5	9	12
**166**	c	6	9	95
**167**	c	7	9	95
**168**	c	8	9	95
**169**	c	9	9	95
**170**	c	10	9	2
**171**	c	11	9	0
**172**	c	12	9	0
**173**	c	13	9	92
**174**	c	14	9	0
**175**	c	15	9	0
**176**	c	16	9	0
**177**	c	17	9	0
**178**	c	18	9	0
**179**	c	19	9	0
**180**	c	20	9	0
**181**	c	4	10	0
**182**	c	5	10	100
**183**	c	6	10	0
**184**	c	7	10	0
**185**	c	8	10	50
**186**	c	9	10	0
**187**	c	10	10	0
**188**	c	11	10	.
**189**	c	13	10	.
**190**	c	15	10	.
**191**	c	17	10	.
**192**	c	19	10	.
**193**	c	1	11	0
**194**	c	2	11	0
**195**	c	3	11	100
**196**	c	4	11	0
**197**	c	6	11	100
**198**	c	7	11	100
**199**	c	8	11	0
**200**	c	9	11	0
**201**	c	10	11	100
**202**	c	11	11	100
**203**	c	12	11	100
**204**	c	13	11	100
**205**	c	14	11	100

Obs	ANI	TEN	RP	Y
**206**	c	15	11	100
**207**	c	16	11	50
**208**	c	17	11	100
**209**	c	18	11	80
**210**	c	19	11	100
**211**	c	20	11	100
**212**	c	1	12	100
**213**	c	2	12	40
**214**	c	3	12	70
**215**	c	4	12	90
**216**	c	5	12	40
**217**	c	6	12	100
**218**	c	7	12	100
**219**	c	8	12	100
**220**	c	9	12	100
**221**	c	10	12	100
**222**	c	11	12	0
**223**	c	12	12	0
**224**	c	13	12	50
**225**	c	15	12	0
**226**	c	16	12	100
**227**	c	17	12	100
**228**	c	18	12	40
**229**	c	19	12	0
**230**	c	20	12	0
**231**	s	1	13	0
**232**	s	2	13	100
**233**	s	3	13	100
**234**	s	5	13	100
**235**	s	6	13	100
**236**	s	7	13	0
**237**	s	10	13	95
**238**	s	12	13	95
**239**	s	13	13	95
**240**	s	14	13	95
**241**	s	15	13	100
**242**	s	16	13	95
**243**	s	17	13	100
**244**	s	18	13	100
**245**	s	19	13	95
**246**	s	20	13	100

Obs	ANI	TEN	RP	Y
247	s	1	14	100
248	s	3	14	100
249	s	4	14	100
250	s	5	14	100
251	s	8	14	100
252	s	9	14	100
253	s	10	14	0
254	s	11	14	100
255	s	12	14	100
256	s	13	14	60
257	s	14	14	100
258	s	15	14	100
259	s	16	14	95
260	s	17	14	50
261	s	18	14	100
262	s	19	14	100
263	s	20	14	0
264	s	1	15	95
265	s	2	15	0
266	s	3	15	100
267	s	4	15	100
268	s	6	15	100
269	s	7	15	100
270	s	8	15	100
271	s	9	15	100
272	s	10	15	100
273	s	11	15	100
274	s	12	15	0
275	s	13	15	0
276	s	14	15	100
277	s	16	15	100
278	s	17	15	100
279	s	18	15	100
280	s	19	15	0
281	s	20	15	30
282	s	1	16	100
283	s	2	16	100
284	s	3	16	70
285	s	4	16	100
286	s	5	16	30
287	s	6	16	100

Obs	ANI	TEN	RP	Y
**288**	s	7	16	100
**289**	s	8	16	100
**290**	s	9	16	50
**291**	s	10	16	100
**292**	s	12	16	0
**293**	s	14	16	0
**294**	s	15	16	50
**295**	s	16	16	100
**296**	s	17	16	100
**297**	s	18	16	100
**298**	s	19	16	100
**299**	s	1	17	0
**300**	s	2	17	0
**301**	s	3	17	100
**302**	s	4	17	100
**303**	s	5	17	60
**304**	s	6	17	0
**305**	s	7	17	100
**306**	s	9	17	100
**307**	s	10	17	100
**308**	s	11	17	100
**309**	s	12	17	40
**310**	s	13	17	95
**311**	s	14	17	100
**312**	s	15	17	0
**313**	s	16	17	100
**314**	s	17	17	95
**315**	s	18	17	40
**316**	s	19	17	100
**317**	s	20	17	95
**318**	s	1	18	100
**319**	s	2	18	0
**320**	s	3	18	0
**321**	s	4	18	0
**322**	s	5	18	100
**323**	s	6	18	100
**324**	s	7	18	0
**325**	s	8	18	0
**326**	s	9	18	100
**327**	s	10	18	100
**328**	s	11	18	0

Obs	ANI	TEN	RP	Y
329	s	12	18	0
330	s	13	18	100
331	s	14	18	0
332	s	15	18	100
333	s	16	18	0
334	s	17	18	0
335	s	18	18	100
336	s	19	18	0
337	s	1	19	100
338	s	2	19	100
339	s	4	19	100
340	s	5	19	100
341	s	6	19	0
342	s	7	19	100
343	s	8	19	100
344	s	9	19	100
345	s	11	19	100
346	s	12	19	100
347	s	13	19	100
348	s	16	19	100
349	s	17	19	100
350	s	19	19	100
351	s	1	20	100
352	s	2	20	50
353	s	3	20	90
354	s	4	20	95
355	s	5	20	100
356	s	7	20	100
357	s	8	20	50
358	s	9	20	60
359	s	10	20	95
360	s	12	20	0
361	s	13	20	95
362	s	14	20	95
363	s	15	20	95
364	s	16	20	100
365	s	17	20	95
366	s	19	20	100
367	s	20	20	100
368	s	1	21	100
369	s	3	21	100

Obs	ANI	TEN	RP	Y
370	s	4	21	100
371	s	5	21	0
372	s	7	21	100
373	s	8	21	100
374	s	9	21	100
375	s	10	21	100
376	s	11	21	100
377	s	12	21	100
378	s	13	21	95
379	s	14	21	100
380	s	15	21	100
381	s	16	21	30
382	s	17	21	30
383	s	19	21	100
384	s	20	21	100
385	s	1	22	100
386	s	2	22	60
387	s	3	22	100
388	s	4	22	100
389	s	5	22	100
390	s	6	22	95
391	s	7	22	5
392	s	8	22	100
393	s	9	22	0
394	s	10	22	0
395	s	11	22	95
396	s	12	22	100
397	s	13	22	100
398	s	14	22	0
399	s	15	22	95
400	s	17	22	100
401	s	18	22	100
402	s	19	22	95
403	s	20	22	0
404	s	1	23	0
405	s	2	23	100
406	s	4	23	95
407	s	5	23	100
408	s	6	23	100
409	s	7	23	100
410	s	8	23	50

Obs	ANI	TEN	RP	Y
**411**	s	9	23	95
**412**	s	10	23	100
**413**	s	11	23	100
**414**	s	12	23	100
**415**	s	13	23	100
**416**	s	14	23	90
**417**	s	15	23	100
**418**	s	16	23	100
**419**	s	17	23	100
**420**	s	18	23	100
**421**	s	20	23	100
**422**	s	2	24	95
**423**	s	4	24	95
**424**	s	5	24	95
**425**	s	6	24	100
**426**	s	7	24	100
**427**	s	8	24	100
**428**	s	9	24	50
**429**	s	10	24	0
**430**	s	11	24	100
**431**	s	12	24	100
**432**	s	13	24	100
**433**	s	14	24	100
**434**	s	15	24	100
**435**	s	16	24	100
**436**	s	17	24	100
**437**	s	18	24	100
**438**	s	19	24	100
**439**	s	20	24	100

Obs	ANI	TEN	RP	Y
1	c	1	1	70
2	c	2	1	95
3	c	3	1	95
4	c	4	1	90
5	c	5	1	95
6	c	6	1	95
7	c	7	1	95
8	c	8	1	95
9	c	9	1	45
10	c	10	1	95
11	c	11	1	95
12	c	12	1	95
13	c	13	1	95
14	c	14	1	95
15	c	15	1	95
16	c	16	1	95
17	c	17	1	95
18	c	18	1	85
19	c	19	1	0
20	c	20	1	95
21	c	1	2	60
22	c	2	2	90
23	c	3	2	40
24	c	4	2	60
25	c	5	2	95
26	c	6	2	80
27	c	7	2	95
28	c	8	2	95
29	c	9	2	95
30	c	10	2	95
31	c	11	2	95
32	c	12	2	85
33	c	13	2	95
34	c	14	2	95
35	c	15	2	90
36	c	16	2	85
37	c	17	2	70
38	c	18	2	95
39	c	19	2	50
40	c	20	2	95
41	c	1	3	0

Obs	ANI	TEN	RP	Y
**42**	c	2	3	95
**43**	c	3	3	0
**44**	c	4	3	95
**45**	c	5	3	95
**46**	c	6	3	0
**47**	c	7	3	95
**48**	c	8	3	0
**49**	c	9	3	0
**50**	c	10	3	50
**51**	c	11	3	100
**52**	c	12	3	100
**53**	c	13	3	5
**54**	c	14	3	30
**55**	c	15	3	95
**56**	c	16	3	40
**57**	c	17	3	95
**58**	c	18	3	90
**59**	c	19	3	30
**60**	c	20	3	20
**61**	c	1	4	45
**62**	c	2	4	60
**63**	c	3	4	60
**64**	c	4	4	0
**65**	c	5	4	10
**66**	c	6	4	0
**67**	c	7	4	45
**68**	c	8	4	95
**69**	c	9	4	0
**70**	c	10	4	40
**71**	c	11	4	95
**72**	c	12	4	90
**73**	c	13	4	0
**74**	c	14	4	95
**75**	c	15	4	15
**76**	c	16	4	100
**77**	c	17	4	60
**78**	c	18	4	50
**79**	c	19	4	50
**80**	c	20	4	10
**81**	c	1	5	78
**82**	c	2	5	95

Obs	ANI	TEN	RP	Y
83	c	3	5	95
84	c	4	5	97
85	c	5	5	95
86	c	6	5	78
87	c	7	5	78
88	c	8	5	78
89	c	9	5	78
90	c	10	5	95
91	c	11	5	98
92	c	12	5	78
93	c	13	5	78
94	c	14	5	78
95	c	15	5	78
96	c	16	5	78
97	c	17	5	78
98	c	18	5	78
99	c	19	5	78
100	c	20	5	78
101	c	1	6	78
102	c	2	6	78
103	c	3	6	78
104	c	4	6	78
105	c	5	6	0
106	c	6	6	78
107	c	7	6	78
108	c	8	6	95
109	c	9	6	78
110	c	10	6	0
111	c	11	6	95
112	c	12	6	95
113	c	13	6	95
114	c	14	6	12
115	c	15	6	12
116	c	16	6	0
117	c	17	6	0
118	c	18	6	2
119	c	19	6	95
120	c	20	6	95
121	c	1	7	12
122	c	2	7	0
123	c	3	7	0

Obs	ANI	TEN	RP	Y
**124**	c	4	7	0
**125**	c	5	7	0
**126**	c	6	7	95
**127**	c	7	7	95
**128**	c	8	7	12
**129**	c	9	7	0
**130**	c	10	7	12
**131**	c	11	7	0
**132**	c	12	7	0
**133**	c	13	7	0
**134**	c	14	7	95
**135**	c	15	7	0
**136**	c	16	7	95
**137**	c	17	7	95
**138**	c	18	7	12
**139**	c	19	7	0
**140**	c	20	7	12
**141**	c	1	8	0
**142**	c	2	8	0
**143**	c	3	8	4
**144**	c	4	8	12
**145**	c	5	8	95
**146**	c	6	8	12
**147**	c	7	8	0
**148**	c	8	8	0
**149**	c	9	8	10
**150**	c	10	8	10
**151**	c	11	8	0
**152**	c	12	8	0
**153**	c	13	8	0
**154**	c	14	8	0
**155**	c	15	8	95
**156**	c	16	8	95
**157**	c	17	8	0
**158**	c	18	8	0
**159**	c	19	8	95
**160**	c	20	8	95
**161**	c	1	9	95
**162**	c	2	9	12
**163**	c	3	9	95
**164**	c	4	9	95

Obs	ANI	TEN	RP	Y
**165**	c	5	9	12
**166**	c	6	9	95
**167**	c	7	9	95
**168**	c	8	9	95
**169**	c	9	9	95
**170**	c	10	9	2
**171**	c	11	9	0
**172**	c	12	9	0
**173**	c	13	9	92
**174**	c	14	9	0
**175**	c	15	9	0
**176**	c	16	9	0
**177**	c	17	9	0
**178**	c	18	9	0
**179**	c	19	9	0
**180**	c	20	9	0
**181**	c	4	10	0
**182**	c	5	10	100
**183**	c	6	10	0
**184**	c	7	10	0
**185**	c	8	10	50
**186**	c	9	10	0
**187**	c	10	10	0
**188**	c	11	10	.
**189**	c	13	10	.
**190**	c	15	10	.
**191**	c	17	10	.
**192**	c	19	10	.
**193**	c	1	11	0
**194**	c	2	11	0
**195**	c	3	11	100
**196**	c	4	11	0
**197**	c	6	11	100
**198**	c	7	11	100
**199**	c	8	11	0
**200**	c	9	11	0
**201**	c	10	11	100
**202**	c	11	11	100
**203**	c	12	11	100
**204**	c	13	11	100
**205**	c	14	11	100

Obs	ANI	TEN	RP	Y
**206**	c	15	11	100
**207**	c	16	11	50
**208**	c	17	11	100
**209**	c	18	11	80
**210**	c	19	11	100
**211**	c	20	11	100
**212**	c	1	12	100
**213**	c	2	12	40
**214**	c	3	12	70
**215**	c	4	12	90
**216**	c	5	12	40
**217**	c	6	12	100
**218**	c	7	12	100
**219**	c	8	12	100
**220**	c	9	12	100
**221**	c	10	12	100
**222**	c	11	12	0
**223**	c	12	12	0
**224**	c	13	12	50
**225**	c	15	12	0
**226**	c	16	12	100
**227**	c	17	12	100
**228**	c	18	12	40
**229**	c	19	12	0
**230**	c	20	12	0
**231**	s	1	13	0
**232**	s	2	13	100
**233**	s	3	13	100
**234**	s	5	13	100
**235**	s	6	13	100
**236**	s	7	13	0
**237**	s	10	13	95
**238**	s	12	13	95
**239**	s	13	13	95
**240**	s	14	13	95
**241**	s	15	13	100
**242**	s	16	13	95
**243**	s	17	13	100
**244**	s	18	13	100
**245**	s	19	13	95
**246**	s	20	13	100

Obs	ANI	TEN	RP	Y
247	s	1	14	100
248	s	3	14	100
249	s	4	14	100
250	s	5	14	100
251	s	8	14	100
252	s	9	14	100
253	s	10	14	0
254	s	11	14	100
255	s	12	14	100
256	s	13	14	60
257	s	14	14	100
258	s	15	14	100
259	s	16	14	95
260	s	17	14	50
261	s	18	14	100
262	s	19	14	100
263	s	20	14	0
264	s	1	15	95
265	s	2	15	0
266	s	3	15	100
267	s	4	15	100
268	s	6	15	100
269	s	7	15	100
270	s	8	15	100
271	s	9	15	100
272	s	10	15	100
273	s	11	15	100
274	s	12	15	0
275	s	13	15	0
276	s	14	15	100
277	s	16	15	100
278	s	17	15	100
279	s	18	15	100
280	s	19	15	0
281	s	20	15	30
282	s	1	16	100
283	s	2	16	100
284	s	3	16	70
285	s	4	16	100
286	s	5	16	30
287	s	6	16	100

Obs	ANI	TEN	RP	Y
288	s	7	16	100
289	s	8	16	100
290	s	9	16	50
291	s	10	16	100
292	s	12	16	0
293	s	14	16	0
294	s	15	16	50
295	s	16	16	100
296	s	17	16	100
297	s	18	16	100
298	s	19	16	100
299	s	1	17	0
300	s	2	17	0
301	s	3	17	100
302	s	4	17	100
303	s	5	17	60
304	s	6	17	0
305	s	7	17	100
306	s	9	17	100
307	s	10	17	100
308	s	11	17	100
309	s	12	17	40
310	s	13	17	95
311	s	14	17	100
312	s	15	17	0
313	s	16	17	100
314	s	17	17	95
315	s	18	17	40
316	s	19	17	100
317	s	20	17	95
318	s	1	18	100
319	s	2	18	0
320	s	3	18	0
321	s	4	18	0
322	s	5	18	100
323	s	6	18	100
324	s	7	18	0
325	s	8	18	0
326	s	9	18	100
327	s	10	18	100
328	s	11	18	0

Obs	ANI	TEN	RP	Y
**329**	s	12	18	0
**330**	s	13	18	100
**331**	s	14	18	0
**332**	s	15	18	100
**333**	s	16	18	0
**334**	s	17	18	0
**335**	s	18	18	100
**336**	s	19	18	0
**337**	s	1	19	100
**338**	s	2	19	100
**339**	s	4	19	100
**340**	s	5	19	100
**341**	s	6	19	0
**342**	s	7	19	100
**343**	s	8	19	100
**344**	s	9	19	100
**345**	s	11	19	100
**346**	s	12	19	100
**347**	s	13	19	100
**348**	s	16	19	100
**349**	s	17	19	100
**350**	s	19	19	100
**351**	s	1	20	100
**352**	s	2	20	50
**353**	s	3	20	90
**354**	s	4	20	95
**355**	s	5	20	100
**356**	s	7	20	100
**357**	s	8	20	50
**358**	s	9	20	60
**359**	s	10	20	95
**360**	s	12	20	0
**361**	s	13	20	95
**362**	s	14	20	95
**363**	s	15	20	95
**364**	s	16	20	100
**365**	s	17	20	95
**366**	s	19	20	100
**367**	s	20	20	100
**368**	s	1	21	100
**369**	s	3	21	100

Obs	ANI	TEN	RP	Y
370	s	4	21	100
371	s	5	21	0
372	s	7	21	100
373	s	8	21	100
374	s	9	21	100
375	s	10	21	100
376	s	11	21	100
377	s	12	21	100
378	s	13	21	95
379	s	14	21	100
380	s	15	21	100
381	s	16	21	30
382	s	17	21	30
383	s	19	21	100
384	s	20	21	100
385	s	1	22	100
386	s	2	22	60
387	s	3	22	100
388	s	4	22	100
389	s	5	22	100
390	s	6	22	95
391	s	7	22	5
392	s	8	22	100
393	s	9	22	0
394	s	10	22	0
395	s	11	22	95
396	s	12	22	100
397	s	13	22	100
398	s	14	22	0
399	s	15	22	95
400	s	17	22	100
401	s	18	22	100
402	s	19	22	95
403	s	20	22	0
404	s	1	23	0
405	s	2	23	100
406	s	4	23	95
407	s	5	23	100
408	s	6	23	100
409	s	7	23	100
410	s	8	23	50

Obs	ANI	TEN	RP	Y
**411**	s	9	23	95
**412**	s	10	23	100
**413**	s	11	23	100
**414**	s	12	23	100
**415**	s	13	23	100
**416**	s	14	23	90
**417**	s	15	23	100
**418**	s	16	23	100
**419**	s	17	23	100
**420**	s	18	23	100
**421**	s	20	23	100
**422**	s	2	24	95
**423**	s	4	24	95
**424**	s	5	24	95
**425**	s	6	24	100
**426**	s	7	24	100
**427**	s	8	24	100
**428**	s	9	24	50
**429**	s	10	24	0
**430**	s	11	24	100
**431**	s	12	24	100
**432**	s	13	24	100
**433**	s	14	24	100
**434**	s	15	24	100
**435**	s	16	24	100
**436**	s	17	24	100
**437**	s	18	24	100
**438**	s	19	24	100
**439**	s	20	24	100

Obs	ANI	TEN	RP	Y
**1**	c	1	1	70
**2**	c	2	1	95
**3**	c	3	1	95
**4**	c	4	1	90
**5**	c	5	1	95
**6**	c	6	1	95
**7**	c	7	1	95
**8**	c	8	1	95
**9**	c	9	1	45
**10**	c	10	1	95
**11**	c	11	1	95
**12**	c	12	1	95
**13**	c	13	1	95
**14**	c	14	1	95
**15**	c	15	1	95
**16**	c	16	1	95
**17**	c	17	1	95
**18**	c	18	1	85
**19**	c	19	1	0
**20**	c	20	1	95
**21**	c	1	2	60
**22**	c	2	2	90
**23**	c	3	2	40
**24**	c	4	2	60
**25**	c	5	2	95
**26**	c	6	2	80
**27**	c	7	2	95
**28**	c	8	2	95
**29**	c	9	2	95
**30**	c	10	2	95
**31**	c	11	2	95
**32**	c	12	2	85
**33**	c	13	2	95
**34**	c	14	2	95
**35**	c	15	2	90
**36**	c	16	2	85
**37**	c	17	2	70
**38**	c	18	2	95
**39**	c	19	2	50
**40**	c	20	2	95
**41**	c	1	3	0

Obs	ANI	TEN	RP	Y
**42**	c	2	3	95
**43**	c	3	3	0
**44**	c	4	3	95
**45**	c	5	3	95
**46**	c	6	3	0
**47**	c	7	3	95
**48**	c	8	3	0
**49**	c	9	3	0
**50**	c	10	3	50
**51**	c	11	3	100
**52**	c	12	3	100
**53**	c	13	3	5
**54**	c	14	3	30
**55**	c	15	3	95
**56**	c	16	3	40
**57**	c	17	3	95
**58**	c	18	3	90
**59**	c	19	3	30
**60**	c	20	3	20
**61**	c	1	4	45
**62**	c	2	4	60
**63**	c	3	4	60
**64**	c	4	4	0
**65**	c	5	4	10
**66**	c	6	4	0
**67**	c	7	4	45
**68**	c	8	4	95
**69**	c	9	4	0
**70**	c	10	4	40
**71**	c	11	4	95
**72**	c	12	4	90
**73**	c	13	4	0
**74**	c	14	4	95
**75**	c	15	4	15
**76**	c	16	4	100
**77**	c	17	4	60
**78**	c	18	4	50
**79**	c	19	4	50
**80**	c	20	4	10
**81**	c	1	5	78
**82**	c	2	5	95

Obs	ANI	TEN	RP	Y
**83**	c	3	5	95
**84**	c	4	5	97
**85**	c	5	5	95
**86**	c	6	5	78
**87**	c	7	5	78
**88**	c	8	5	78
**89**	c	9	5	78
**90**	c	10	5	95
**91**	c	11	5	98
**92**	c	12	5	78
**93**	c	13	5	78
**94**	c	14	5	78
**95**	c	15	5	78
**96**	c	16	5	78
**97**	c	17	5	78
**98**	c	18	5	78
**99**	c	19	5	78
**100**	c	20	5	78
**101**	c	1	6	78
**102**	c	2	6	78
**103**	c	3	6	78
**104**	c	4	6	78
**105**	c	5	6	0
**106**	c	6	6	78
**107**	c	7	6	78
**108**	c	8	6	95
**109**	c	9	6	78
**110**	c	10	6	0
**111**	c	11	6	95
**112**	c	12	6	95
**113**	c	13	6	95
**114**	c	14	6	12
**115**	c	15	6	12
**116**	c	16	6	0
**117**	c	17	6	0
**118**	c	18	6	2
**119**	c	19	6	95
**120**	c	20	6	95
**121**	c	1	7	12
**122**	c	2	7	0
**123**	c	3	7	0

Obs	ANI	TEN	RP	Y
**124**	c	4	7	0
**125**	c	5	7	0
**126**	c	6	7	95
**127**	c	7	7	95
**128**	c	8	7	12
**129**	c	9	7	0
**130**	c	10	7	12
**131**	c	11	7	0
**132**	c	12	7	0
**133**	c	13	7	0
**134**	c	14	7	95
**135**	c	15	7	0
**136**	c	16	7	95
**137**	c	17	7	95
**138**	c	18	7	12
**139**	c	19	7	0
**140**	c	20	7	12
**141**	c	1	8	0
**142**	c	2	8	0
**143**	c	3	8	4
**144**	c	4	8	12
**145**	c	5	8	95
**146**	c	6	8	12
**147**	c	7	8	0
**148**	c	8	8	0
**149**	c	9	8	10
**150**	c	10	8	10
**151**	c	11	8	0
**152**	c	12	8	0
**153**	c	13	8	0
**154**	c	14	8	0
**155**	c	15	8	95
**156**	c	16	8	95
**157**	c	17	8	0
**158**	c	18	8	0
**159**	c	19	8	95
**160**	c	20	8	95
**161**	c	1	9	95
**162**	c	2	9	12
**163**	c	3	9	95
**164**	c	4	9	95

Obs	ANI	TEN	RP	Y
**165**	c	5	9	12
**166**	c	6	9	95
**167**	c	7	9	95
**168**	c	8	9	95
**169**	c	9	9	95
**170**	c	10	9	2
**171**	c	11	9	0
**172**	c	12	9	0
**173**	c	13	9	92
**174**	c	14	9	0
**175**	c	15	9	0
**176**	c	16	9	0
**177**	c	17	9	0
**178**	c	18	9	0
**179**	c	19	9	0
**180**	c	20	9	0
**181**	c	4	10	0
**182**	c	5	10	100
**183**	c	6	10	0
**184**	c	7	10	0
**185**	c	8	10	50
**186**	c	9	10	0
**187**	c	10	10	0
**188**	c	11	10	.
**189**	c	13	10	.
**190**	c	15	10	.
**191**	c	17	10	.
**192**	c	19	10	.
**193**	c	1	11	0
**194**	c	2	11	0
**195**	c	3	11	100
**196**	c	4	11	0
**197**	c	6	11	100
**198**	c	7	11	100
**199**	c	8	11	0
**200**	c	9	11	0
**201**	c	10	11	100
**202**	c	11	11	100
**203**	c	12	11	100
**204**	c	13	11	100
**205**	c	14	11	100

Obs	ANI	TEN	RP	Y
**206**	c	15	11	100
**207**	c	16	11	50
**208**	c	17	11	100
**209**	c	18	11	80
**210**	c	19	11	100
**211**	c	20	11	100
**212**	c	1	12	100
**213**	c	2	12	40
**214**	c	3	12	70
**215**	c	4	12	90
**216**	c	5	12	40
**217**	c	6	12	100
**218**	c	7	12	100
**219**	c	8	12	100
**220**	c	9	12	100
**221**	c	10	12	100
**222**	c	11	12	0
**223**	c	12	12	0
**224**	c	13	12	50
**225**	c	15	12	0
**226**	c	16	12	100
**227**	c	17	12	100
**228**	c	18	12	40
**229**	c	19	12	0
**230**	c	20	12	0
**231**	s	1	13	0
**232**	s	2	13	100
**233**	s	3	13	100
**234**	s	5	13	100
**235**	s	6	13	100
**236**	s	7	13	0
**237**	s	10	13	95
**238**	s	12	13	95
**239**	s	13	13	95
**240**	s	14	13	95
**241**	s	15	13	100
**242**	s	16	13	95
**243**	s	17	13	100
**244**	s	18	13	100
**245**	s	19	13	95
**246**	s	20	13	100

Obs	ANI	TEN	RP	Y
**247**	s	1	14	100
**248**	s	3	14	100
**249**	s	4	14	100
**250**	s	5	14	100
**251**	s	8	14	100
**252**	s	9	14	100
**253**	s	10	14	0
**254**	s	11	14	100
**255**	s	12	14	100
**256**	s	13	14	60
**257**	s	14	14	100
**258**	s	15	14	100
**259**	s	16	14	95
**260**	s	17	14	50
**261**	s	18	14	100
**262**	s	19	14	100
**263**	s	20	14	0
**264**	s	1	15	95
**265**	s	2	15	0
**266**	s	3	15	100
**267**	s	4	15	100
**268**	s	6	15	100
**269**	s	7	15	100
**270**	s	8	15	100
**271**	s	9	15	100
**272**	s	10	15	100
**273**	s	11	15	100
**274**	s	12	15	0
**275**	s	13	15	0
**276**	s	14	15	100
**277**	s	16	15	100
**278**	s	17	15	100
**279**	s	18	15	100
**280**	s	19	15	0
**281**	s	20	15	30
**282**	s	1	16	100
**283**	s	2	16	100
**284**	s	3	16	70
**285**	s	4	16	100
**286**	s	5	16	30
**287**	s	6	16	100

Obs	ANI	TEN	RP	Y
**288**	s	7	16	100
**289**	s	8	16	100
**290**	s	9	16	50
**291**	s	10	16	100
**292**	s	12	16	0
**293**	s	14	16	0
**294**	s	15	16	50
**295**	s	16	16	100
**296**	s	17	16	100
**297**	s	18	16	100
**298**	s	19	16	100
**299**	s	1	17	0
**300**	s	2	17	0
**301**	s	3	17	100
**302**	s	4	17	100
**303**	s	5	17	60
**304**	s	6	17	0
**305**	s	7	17	100
**306**	s	9	17	100
**307**	s	10	17	100
**308**	s	11	17	100
**309**	s	12	17	40
**310**	s	13	17	95
**311**	s	14	17	100
**312**	s	15	17	0
**313**	s	16	17	100
**314**	s	17	17	95
**315**	s	18	17	40
**316**	s	19	17	100
**317**	s	20	17	95
**318**	s	1	18	100
**319**	s	2	18	0
**320**	s	3	18	0
**321**	s	4	18	0
**322**	s	5	18	100
**323**	s	6	18	100
**324**	s	7	18	0
**325**	s	8	18	0
**326**	s	9	18	100
**327**	s	10	18	100
**328**	s	11	18	0

Obs	ANI	TEN	RP	Y
**329**	s	12	18	0
**330**	s	13	18	100
**331**	s	14	18	0
**332**	s	15	18	100
**333**	s	16	18	0
**334**	s	17	18	0
**335**	s	18	18	100
**336**	s	19	18	0
**337**	s	1	19	100
**338**	s	2	19	100
**339**	s	4	19	100
**340**	s	5	19	100
**341**	s	6	19	0
**342**	s	7	19	100
**343**	s	8	19	100
**344**	s	9	19	100
**345**	s	11	19	100
**346**	s	12	19	100
**347**	s	13	19	100
**348**	s	16	19	100
**349**	s	17	19	100
**350**	s	19	19	100
**351**	s	1	20	100
**352**	s	2	20	50
**353**	s	3	20	90
**354**	s	4	20	95
**355**	s	5	20	100
**356**	s	7	20	100
**357**	s	8	20	50
**358**	s	9	20	60
**359**	s	10	20	95
**360**	s	12	20	0
**361**	s	13	20	95
**362**	s	14	20	95
**363**	s	15	20	95
**364**	s	16	20	100
**365**	s	17	20	95
**366**	s	19	20	100
**367**	s	20	20	100
**368**	s	1	21	100
**369**	s	3	21	100

Obs	ANI	TEN	RP	Y
**370**	s	4	21	100
**371**	s	5	21	0
**372**	s	7	21	100
**373**	s	8	21	100
**374**	s	9	21	100
**375**	s	10	21	100
**376**	s	11	21	100
**377**	s	12	21	100
**378**	s	13	21	95
**379**	s	14	21	100
**380**	s	15	21	100
**381**	s	16	21	30
**382**	s	17	21	30
**383**	s	19	21	100
**384**	s	20	21	100
**385**	s	1	22	100
**386**	s	2	22	60
**387**	s	3	22	100
**388**	s	4	22	100
**389**	s	5	22	100
**390**	s	6	22	95
**391**	s	7	22	5
**392**	s	8	22	100
**393**	s	9	22	0
**394**	s	10	22	0
**395**	s	11	22	95
**396**	s	12	22	100
**397**	s	13	22	100
**398**	s	14	22	0
**399**	s	15	22	95
**400**	s	17	22	100
**401**	s	18	22	100
**402**	s	19	22	95
**403**	s	20	22	0
**404**	s	1	23	0
**405**	s	2	23	100
**406**	s	4	23	95
**407**	s	5	23	100
**408**	s	6	23	100
**409**	s	7	23	100
**410**	s	8	23	50

Obs	ANI	TEN	RP	Y
411	s	9	23	95
412	s	10	23	100
413	s	11	23	100
414	s	12	23	100
415	s	13	23	100
416	s	14	23	90
417	s	15	23	100
418	s	16	23	100
419	s	17	23	100
420	s	18	23	100
421	s	20	23	100
422	s	2	24	95
423	s	4	24	95
424	s	5	24	95
425	s	6	24	100
426	s	7	24	100
427	s	8	24	100
428	s	9	24	50
429	s	10	24	0
430	s	11	24	100
431	s	12	24	100
432	s	13	24	100
433	s	14	24	100
434	s	15	24	100
435	s	16	24	100
436	s	17	24	100
437	s	18	24	100
438	s	19	24	100
439	s	20	24	100

The Mixed Procedure

Model Information	
Data Set	WORK.AU
Dependent Variable	Y
Covariance Structure	Ante-dependence
Subject Effect	RP(TEN)
Estimation Method	REML
Residual Variance Method	None
Fixed Effects SE Method	Model-Based
Degrees of Freedom Method	Between-Within

Class Level Information		
Class	Levels	Values
RP	24	1 2 3 4 5 6 7 8 9 10 11 12 13 14 15 16 17 18 19 20 21 22 23 24
TEN	20	1 10 11 12 13 14 15 16 17 18 19 2 20 3 4 5 6 7 8 9
ANI	2	c s

Dimensions	
Covariance Parameters	3
Columns in X	63
Columns in Z	0
Subjects	439
Max Obs per Subject	1

Number of Observations	
Number of Observations Read	439
Number of Observations Used	434
Number of Observations Not Used	5

Iteration History			
Iteration	Evaluations	-2 Res Log Like	Criterion
0	1	4142.84756616	
1	2	4140.75409301	0.00000000

Convergence criteria met but final Hessian is not positive definite.

Estimated R Matrix for RP(TEN) 1 1	
Row	Col1
1	1828.11

The Mixed Procedure

Estimated R Correlation Matrix for RP(TEN) 1 1	
**Row**	**Col1**
**1**	1.0000

Covariance Parameter Estimates		
**Cov Parm**	**Subject**	**Estimate**
**Var(1)**	RP(TEN)	1828.11
**Var(2)**	RP(TEN)	1480.89
**Rho(1)**	RP(TEN)	0

Fit Statistics	
**-2 Res Log Likelihood**	4140.8
**AIC (Smaller is Better)**	4146.8
**AICC (Smaller is Better)**	4146.8
**BIC (Smaller is Better)**	4159.0

Null Model Likelihood Ratio Test		
**DF**	**Chi-Square**	**Pr > ChiSq**
2	2.09	0.3511

Type 3 Tests of Fixed Effects				
**Effect**	**Num DF**	**Den DF**	**F Value**	**Pr > F**
**TEN**	19	394	0.43	0.9844
**ANI**	1	394	32.50	<.0001
**TEN*ANI**	19	394	0.46	0.9769

The Mixed Procedure

Model Information	
**Data Set**	WORK.AU
**Dependent Variable**	Y
**Covariance Structure**	Autoregressive
**Subject Effect**	RP(TEN)
**Estimation Method**	REML
**Residual Variance Method**	Profile
**Fixed Effects SE Method**	Model-Based
**Degrees of Freedom Method**	Between-Within

Class Level Information		
**Class**	**Levels**	**Values**
**RP**	24	1 2 3 4 5 6 7 8 9 10 11 12 13 14 15 16 17 18 19 20 21 22 23 24
**TEN**	20	1 10 11 12 13 14 15 16 17 18 19 2 20 3 4 5 6 7 8 9
**ANI**	2	c s

Dimensions	
**Covariance Parameters**	2
**Columns in X**	63
**Columns in Z**	0
**Subjects**	439
**Max Obs per Subject**	1

Number of Observations	
**Number of Observations Read**	439
**Number of Observations Used**	434
**Number of Observations Not Used**	5

Iteration History			
**Iteration**	**Evaluations**	**-2 Res Log Like**	**Criterion**
**0**	1	4142.84756616	
**1**	1	4142.84756616	0.00000000

Convergence criteria met but final Hessian is not positive definite.

Estimated R Matrix for RP(TEN) 1 1	
**Row**	**Col1**
**1**	1694.72

The Mixed Procedure

Estimated R Correlation Matrix for RP(TEN) 1 1	
**Row**	**Col1**
**1**	1.0000

Covariance Parameter Estimates		
**Cov Parm**	**Subject**	**Estimate**
**AR(1)**	RP(TEN)	0
**Residual**		1694.72

Fit Statistics	
**-2 Res Log Likelihood**	4142.8
**AIC (Smaller is Better)**	4146.8
**AICC (Smaller is Better)**	4146.9
**BIC (Smaller is Better)**	4155.0

Null Model Likelihood Ratio Test		
**DF**	**Chi-Square**	**Pr > ChiSq**
1	0.00	1.0000

Type 3 Tests of Fixed Effects				
**Effect**	**Num DF**	**Den DF**	**F Value**	**Pr > F**
**TEN**	19	394	0.42	0.9867
**ANI**	1	394	31.59	<.0001
**TEN*ANI**	19	394	0.45	0.9803

The Mixed Procedure

Model Information	
**Data Set**	WORK.AU
**Dependent Variable**	Y
**Covariance Structure**	Heterogeneous Autoregressive
**Subject Effect**	RP(TEN)
**Estimation Method**	REML
**Residual Variance Method**	None
**Fixed Effects SE Method**	Model-Based
**Degrees of Freedom Method**	Between-Within

Class Level Information		
**Class**	**Levels**	**Values**
**RP**	24	1 2 3 4 5 6 7 8 9 10 11 12 13 14 15 16 17 18 19 20 21 22 23 24
**TEN**	20	1 10 11 12 13 14 15 16 17 18 19 2 20 3 4 5 6 7 8 9
**ANI**	2	c s

Dimensions	
**Covariance Parameters**	3
**Columns in X**	63
**Columns in Z**	0
**Subjects**	439
**Max Obs per Subject**	1

Number of Observations	
**Number of Observations Read**	439
**Number of Observations Used**	434
**Number of Observations Not Used**	5

Iteration History			
**Iteration**	**Evaluations**	**-2 Res Log Like**	**Criterion**
**0**	1	4142.84756616	
**1**	2	4140.75409301	0.00000000

Convergence criteria met but final Hessian is not positive definite.

Estimated R Matrix for RP(TEN) 1 1	
**Row**	**Col1**
**1**	1828.11

The Mixed Procedure

Estimated R Correlation Matrix for RP(TEN) 1 1	
**Row**	**Col1**
**1**	1.0000

Covariance Parameter Estimates		
**Cov Parm**	**Subject**	**Estimate**
**Var(1)**	RP(TEN)	1828.11
**Var(2)**	RP(TEN)	1480.89
**ARH(1)**	RP(TEN)	0

Fit Statistics	
**-2 Res Log Likelihood**	4140.8
**AIC (Smaller is Better)**	4146.8
**AICC (Smaller is Better)**	4146.8
**BIC (Smaller is Better)**	4159.0

Null Model Likelihood Ratio Test		
**DF**	**Chi-Square**	**Pr > ChiSq**
2	2.09	0.3511

Type 3 Tests of Fixed Effects				
**Effect**	**Num DF**	**Den DF**	**F Value**	**Pr > F**
**TEN**	19	394	0.43	0.9844
**ANI**	1	394	32.50	<.0001
**TEN*ANI**	19	394	0.46	0.9769

The Mixed Procedure

Model Information	
**Data Set**	WORK.AU
**Dependent Variable**	Y
**Covariance Structure**	Autoregressive Moving Average
**Subject Effect**	RP(TEN)
**Estimation Method**	REML
**Residual Variance Method**	Profile
**Fixed Effects SE Method**	Model-Based
**Degrees of Freedom Method**	Between-Within

Class Level Information		
**Class**	**Levels**	**Values**
**RP**	24	1 2 3 4 5 6 7 8 9 10 11 12 13 14 15 16 17 18 19 20 21 22 23 24
**TEN**	20	1 10 11 12 13 14 15 16 17 18 19 2 20 3 4 5 6 7 8 9
**ANI**	2	c s

Dimensions	
**Covariance Parameters**	3
**Columns in X**	63
**Columns in Z**	0
**Subjects**	439
**Max Obs per Subject**	1

Number of Observations	
**Number of Observations Read**	439
**Number of Observations Used**	434
**Number of Observations Not Used**	5

Iteration History			
**Iteration**	**Evaluations**	**-2 Res Log Like**	**Criterion**
**0**	1	4142.84756616	
**1**	1	4142.84756616	0.00000000

Convergence criteria met but final Hessian is not positive definite.

Estimated R Matrix for RP(TEN) 1 1	
**Row**	**Col1**
**1**	1694.72

The Mixed Procedure

Estimated R Correlation Matrix for RP(TEN) 1 1	
Row	Col1
1	1.0000

Covariance Parameter Estimates		
Cov Parm	Subject	Estimate
Rho	RP(TEN)	0
Gamma	RP(TEN)	0
Residual		1694.72

Fit Statistics	
-2 Res Log Likelihood	4142.8
AIC (Smaller is Better)	4148.8
AICC (Smaller is Better)	4148.9
BIC (Smaller is Better)	4161.1

Null Model Likelihood Ratio Test		
DF	Chi-Square	Pr > ChiSq
2	0.00	1.0000

Type 3 Tests of Fixed Effects				
Effect	Num DF	Den DF	F Value	Pr > F
TEN	19	394	0.42	0.9867
ANI	1	394	31.59	<.0001
TEN*ANI	19	394	0.45	0.9803

The Mixed Procedure

Model Information	
Data Set	WORK.AU
Dependent Variable	Y
Covariance Structure	Compound Symmetry
Subject Effect	RP(TEN)
Estimation Method	REML
Residual Variance Method	Profile
Fixed Effects SE Method	Model-Based
Degrees of Freedom Method	Between-Within

Class Level Information		
Class	Levels	Values
RP	24	1 2 3 4 5 6 7 8 9 10 11 12 13 14 15 16 17 18 19 20 21 22 23 24
TEN	20	1 10 11 12 13 14 15 16 17 18 19 2 20 3 4 5 6 7 8 9
ANI	2	c s

Dimensions	
Covariance Parameters	2
Columns in X	63
Columns in Z	0
Subjects	439
Max Obs per Subject	1

Number of Observations	
Number of Observations Read	439
Number of Observations Used	434
Number of Observations Not Used	5

Iteration History			
Iteration	Evaluations	-2 Res Log Like	Criterion
0	1	4142.84756616	
1	1	4142.84756616	0.00000000

Convergence criteria met.

Estimated R Matrix for RP(TEN) 1 1	
Row	Col1
1	1694.72

The Mixed Procedure

Estimated R Correlation Matrix for RP(TEN) 1 1	
**Row**	**Col1**
**1**	1.0000

Covariance Parameter Estimates		
**Cov Parm**	**Subject**	**Estimate**
**CS**	RP(TEN)	1693.72
**Residual**		0.9994

Fit Statistics	
**-2 Res Log Likelihood**	4142.8
**AIC (Smaller is Better)**	4146.8
**AICC (Smaller is Better)**	4146.9
**BIC (Smaller is Better)**	4155.0

Null Model Likelihood Ratio Test		
**DF**	**Chi-Square**	**Pr > ChiSq**
1	0.00	1.0000

Type 3 Tests of Fixed Effects				
**Effect**	**Num DF**	**Den DF**	**F Value**	**Pr > F**
**TEN**	19	394	0.42	0.9867
**ANI**	1	394	31.59	<.0001
**TEN*ANI**	19	394	0.45	0.9803

The Mixed Procedure

Model Information	
**Data Set**	WORK.AU
**Dependent Variable**	Y
**Covariance Structure**	Heterogeneous Compound Symmetry
**Subject Effect**	RP(TEN)
**Estimation Method**	REML
**Residual Variance Method**	None
**Fixed Effects SE Method**	Model-Based
**Degrees of Freedom Method**	Between-Within

Class Level Information		
**Class**	**Levels**	**Values**
**RP**	24	1 2 3 4 5 6 7 8 9 10 11 12 13 14 15 16 17 18 19 20 21 22 23 24
**TEN**	20	1 10 11 12 13 14 15 16 17 18 19 2 20 3 4 5 6 7 8 9
**ANI**	2	c s

Dimensions	
**Covariance Parameters**	3
**Columns in X**	63
**Columns in Z**	0
**Subjects**	439
**Max Obs per Subject**	1

Number of Observations	
**Number of Observations Read**	439
**Number of Observations Used**	434
**Number of Observations Not Used**	5

Iteration History			
**Iteration**	**Evaluations**	**-2 Res Log Like**	**Criterion**
**0**	1	4142.84756616	
**1**	2	4140.75409301	0.00000000

Convergence criteria met but final Hessian is not positive definite.

Estimated R Matrix for RP(TEN) 1 1	
**Row**	**Col1**
**1**	1828.11

The Mixed Procedure

Estimated R Correlation Matrix for RP(TEN) 1 1	
Row	Col1
1	1.0000

Covariance Parameter Estimates		
Cov Parm	Subject	Estimate
Var(1)	RP(TEN)	1828.11
Var(2)	RP(TEN)	1480.89
CSH	RP(TEN)	0

Fit Statistics	
-2 Res Log Likelihood	4140.8
AIC (Smaller is Better)	4146.8
AICC (Smaller is Better)	4146.8
BIC (Smaller is Better)	4159.0

Null Model Likelihood Ratio Test		
DF	Chi-Square	Pr > ChiSq
2	2.09	0.3511

Type 3 Tests of Fixed Effects				
Effect	Num DF	Den DF	F Value	Pr > F
TEN	19	394	0.43	0.9844
ANI	1	394	32.50	<.0001
TEN*ANI	19	394	0.46	0.9769

The Mixed Procedure

Model Information	
**Data Set**	WORK.AU
**Dependent Variable**	Y
**Covariance Structure**	Factor Analytic
**Subject Effect**	RP(TEN)
**Estimation Method**	REML
**Residual Variance Method**	None
**Fixed Effects SE Method**	Model-Based
**Degrees of Freedom Method**	Between-Within

Class Level Information		
**Class**	**Levels**	**Values**
**RP**	24	1 2 3 4 5 6 7 8 9 10 11 12 13 14 15 16 17 18 19 20 21 22 23 24
**TEN**	20	1 10 11 12 13 14 15 16 17 18 19 2 20 3 4 5 6 7 8 9
**ANI**	2	c s

Dimensions	
**Covariance Parameters**	2
**Columns in X**	63
**Columns in Z**	0
**Subjects**	439
**Max Obs per Subject**	1

Number of Observations	
**Number of Observations Read**	439
**Number of Observations Used**	434
**Number of Observations Not Used**	5

The Mixed Procedure

Model Information	
**Data Set**	WORK.AU
**Dependent Variable**	Y
**Covariance Structure**	Factor Analytic
**Subject Effect**	RP(TEN)
**Estimation Method**	REML
**Residual Variance Method**	None
**Fixed Effects SE Method**	Model-Based
**Degrees of Freedom Method**	Between-Within

Class Level Information		
**Class**	**Levels**	**Values**
**RP**	24	1 2 3 4 5 6 7 8 9 10 11 12 13 14 15 16 17 18 19 20 21 22 23 24
**TEN**	20	1 10 11 12 13 14 15 16 17 18 19 2 20 3 4 5 6 7 8 9
**ANI**	2	c s

Dimensions	
**Covariance Parameters**	4
**Columns in X**	63
**Columns in Z**	0
**Subjects**	439
**Max Obs per Subject**	1

Number of Observations	
**Number of Observations Read**	439
**Number of Observations Used**	434
**Number of Observations Not Used**	5

Iteration History			
**Iteration**	**Evaluations**	**-2 Res Log Like**	**Criterion**
**0**	1	4142.84756616	
**1**	2	4214.14766660	0.07471466
**2**	3	4142.02200207	0.00025626
**3**	2	4140.91165658	0.00014281
**4**	1	4140.67744765	.
**5**	1	4140.67663343	.
**6**	1	4140.67663342	0.00000000

Convergence criteria met but final Hessian is not positive definite.

The Mixed Procedure

Estimated R Matrix for RP(TEN) 1 1	
**Row**	**Col1**
**1**	1864.58

Estimated R Correlation Matrix for RP(TEN) 1 1	
**Row**	**Col1**
**1**	1.0000

Covariance Parameter Estimates		
**Cov Parm**	**Subject**	**Estimate**
**FA(1)**	RP(TEN)	1655.18
**FA(2)**	RP(TEN)	2.345E-6
**FA(1,1)**	RP(TEN)	14.4708
**FA(2,1)**	RP(TEN)	38.8647

Fit Statistics	
**-2 Res Log Likelihood**	4140.7
**AIC (Smaller is Better)**	4148.7
**AICC (Smaller is Better)**	4148.8
**BIC (Smaller is Better)**	4165.0

Null Model Likelihood Ratio Test		
**DF**	**Chi-Square**	**Pr > ChiSq**
3	2.17	0.5377

Type 3 Tests of Fixed Effects				
**Effect**	**Num DF**	**Den DF**	**F Value**	**Pr > F**
**TEN**	19	394	0.42	0.9861
**ANI**	1	394	31.86	<.0001
**TEN*ANI**	19	394	0.45	0.9793

The Mixed Procedure

Model Information	
**Data Set**	WORK.AU
**Dependent Variable**	Y
**Covariance Structure**	Huynh-Feldt
**Subject Effect**	RP(TEN)
**Estimation Method**	REML
**Residual Variance Method**	None
**Fixed Effects SE Method**	Model-Based
**Degrees of Freedom Method**	Between-Within

Class Level Information		
**Class**	**Levels**	**Values**
**RP**	24	1 2 3 4 5 6 7 8 9 10 11 12 13 14 15 16 17 18 19 20 21 22 23 24
**TEN**	20	1 10 11 12 13 14 15 16 17 18 19 2 20 3 4 5 6 7 8 9
**ANI**	2	c s

Dimensions	
**Covariance Parameters**	3
**Columns in X**	63
**Columns in Z**	0
**Subjects**	439
**Max Obs per Subject**	1

Number of Observations	
**Number of Observations Read**	439
**Number of Observations Used**	434
**Number of Observations Not Used**	5

Iteration History			
**Iteration**	**Evaluations**	**-2 Res Log Like**	**Criterion**
**0**	1	4142.84756616	
**1**	2	4223.56640873	0.00000031
**2**	1	4151.48816137	0.00000018
**3**	3	4141.02368331	0.00000001
**4**	1	4140.67931627	0.00000000

Convergence criteria met but final Hessian is not positive definite.

The Mixed Procedure

Estimated R Matrix for RP(TEN) 1 1	
Row	Col1
1	1859.66

Estimated R Correlation Matrix for RP(TEN) 1 1	
Row	Col1
1	1.0000

Covariance Parameter Estimates		
Cov Parm	Subject	Estimate
Var(1)	RP(TEN)	1859.66
Var(2)	RP(TEN)	1503.60
HF	RP(TEN)	4695.65

Fit Statistics	
-2 Res Log Likelihood	4140.7
AIC (Smaller is Better)	4146.7
AICC (Smaller is Better)	4146.7
BIC (Smaller is Better)	4158.9

Null Model Likelihood Ratio Test		
DF	Chi-Square	Pr > ChiSq
2	2.17	0.3382

Type 3 Tests of Fixed Effects				
Effect	Num DF	Den DF	F Value	Pr > F
TEN	19	394	0.42	0.9858
ANI	1	394	31.98	<.0001
TEN*ANI	19	394	0.45	0.9789

The Mixed Procedure

Model Information	
**Data Set**	WORK.AU
**Dependent Variable**	Y
**Covariance Structure**	Variance Components
**Subject Effect**	RP(TEN)
**Estimation Method**	REML
**Residual Variance Method**	Parameter
**Fixed Effects SE Method**	Model-Based
**Degrees of Freedom Method**	Between-Within

Class Level Information		
**Class**	**Levels**	**Values**
**RP**	24	1 2 3 4 5 6 7 8 9 10 11 12 13 14 15 16 17 18 19 20 21 22 23 24
**TEN**	20	1 10 11 12 13 14 15 16 17 18 19 2 20 3 4 5 6 7 8 9
**ANI**	2	c s

Dimensions	
**Covariance Parameters**	1
**Columns in X**	63
**Columns in Z**	0
**Subjects**	439
**Max Obs per Subject**	1

Number of Observations	
**Number of Observations Read**	439
**Number of Observations Used**	434
**Number of Observations Not Used**	5

Iteration History			
**Iteration**	**Evaluations**	**-2 Res Log Like**	**Criterion**
**0**	1	4142.84756616	
**1**	1	4142.84756616	0.00000000

Convergence criteria met.

Estimated R Matrix for RP(TEN) 1 1	
**Row**	**Col1**
**1**	1694.72

The Mixed Procedure

Estimated R Correlation Matrix for RP(TEN) 1 1	
**Row**	**Col1**
**1**	1.0000

Covariance Parameter Estimates		
**Cov Parm**	**Subject**	**Estimate**
**ANI**	RP(TEN)	1694.72

Fit Statistics	
**-2 Res Log Likelihood**	4142.8
**AIC (Smaller is Better)**	4144.8
**AICC (Smaller is Better)**	4144.9
**BIC (Smaller is Better)**	4148.9

Null Model Likelihood Ratio Test		
**DF**	**Chi-Square**	**Pr > ChiSq**
0	0.00	1.0000

Type 3 Tests of Fixed Effects				
**Effect**	**Num DF**	**Den DF**	**F Value**	**Pr > F**
**TEN**	19	394	0.42	0.9867
**ANI**	1	394	31.59	<.0001
**TEN*ANI**	19	394	0.45	0.9803

The Mixed Procedure

Model Information	
**Data Set**	WORK.AU
**Dependent Variable**	Y
**Covariance Structure**	Toeplitz
**Subject Effect**	RP(TEN)
**Estimation Method**	REML
**Residual Variance Method**	Profile
**Fixed Effects SE Method**	Model-Based
**Degrees of Freedom Method**	Between-Within

Class Level Information		
**Class**	**Levels**	**Values**
**RP**	24	1 2 3 4 5 6 7 8 9 10 11 12 13 14 15 16 17 18 19 20 21 22 23 24
**TEN**	20	1 10 11 12 13 14 15 16 17 18 19 2 20 3 4 5 6 7 8 9
**ANI**	2	c s

Dimensions	
**Covariance Parameters**	2
**Columns in X**	63
**Columns in Z**	0
**Subjects**	439
**Max Obs per Subject**	1

Number of Observations	
**Number of Observations Read**	439
**Number of Observations Used**	434
**Number of Observations Not Used**	5

Iteration History			
**Iteration**	**Evaluations**	**-2 Res Log Like**	**Criterion**
**0**	1	4142.84756616	
**1**	1	4142.84756616	0.00000000

Convergence criteria met but final Hessian is not positive definite.

Estimated R Matrix for RP(TEN) 1 1	
**Row**	**Col1**
**1**	1694.72

The Mixed Procedure

Estimated R Correlation Matrix for RP(TEN) 1 1	
**Row**	**Col1**
**1**	1.0000

Covariance Parameter Estimates		
**Cov Parm**	**Subject**	**Estimate**
**TOEP(2)**	RP(TEN)	0
**Residual**		1694.72

Fit Statistics	
**-2 Res Log Likelihood**	4142.8
**AIC (Smaller is Better)**	4146.8
**AICC (Smaller is Better)**	4146.9
**BIC (Smaller is Better)**	4155.0

Null Model Likelihood Ratio Test		
**DF**	**Chi-Square**	**Pr > ChiSq**
1	0.00	1.0000

Type 3 Tests of Fixed Effects				
**Effect**	**Num DF**	**Den DF**	**F Value**	**Pr > F**
**TEN**	19	394	0.42	0.9867
**ANI**	1	394	31.59	<.0001
**TEN*ANI**	19	394	0.45	0.9803

The Mixed Procedure

Model Information	
Data Set	WORK.AU
Dependent Variable	Y
Covariance Structure	Heterogeneous Toeplitz
Subject Effect	RP(TEN)
Estimation Method	REML
Residual Variance Method	None
Fixed Effects SE Method	Model-Based
Degrees of Freedom Method	Between-Within

Class Level Information		
Class	Levels	Values
RP	24	1 2 3 4 5 6 7 8 9 10 11 12 13 14 15 16 17 18 19 20 21 22 23 24
TEN	20	1 10 11 12 13 14 15 16 17 18 19 2 20 3 4 5 6 7 8 9
ANI	2	c s

Dimensions	
Covariance Parameters	3
Columns in X	63
Columns in Z	0
Subjects	439
Max Obs per Subject	1

Number of Observations	
Number of Observations Read	439
Number of Observations Used	434
Number of Observations Not Used	5

Iteration History			
Iteration	Evaluations	-2 Res Log Like	Criterion
0	1	4142.84756616	
1	2	4140.75409301	0.00000000

Convergence criteria met but final Hessian is not positive definite.

Estimated R Matrix for RP(TEN) 1 1	
Row	Col1
1	1828.11

The Mixed Procedure

Estimated R Correlation Matrix for RP(TEN) 1 1	
**Row**	**Col1**
**1**	1.0000

Covariance Parameter Estimates		
**Cov Parm**	**Subject**	**Estimate**
**Var(1)**	RP(TEN)	1828.11
**Var(2)**	RP(TEN)	1480.89
**TOEPH(1)**	RP(TEN)	0

Fit Statistics	
**-2 Res Log Likelihood**	4140.8
**AIC (Smaller is Better)**	4146.8
**AICC (Smaller is Better)**	4146.8
**BIC (Smaller is Better)**	4159.0

Null Model Likelihood Ratio Test		
**DF**	**Chi-Square**	**Pr > ChiSq**
2	2.09	0.3511

Type 3 Tests of Fixed Effects				
**Effect**	**Num DF**	**Den DF**	**F Value**	**Pr > F**
**TEN**	19	394	0.43	0.9844
**ANI**	1	394	32.50	<.0001
**TEN*ANI**	19	394	0.46	0.9769

The Mixed Procedure

Model Information	
Data Set	WORK.AU
Dependent Variable	Y
Covariance Structure	Unstructured
Subject Effect	RP(TEN)
Estimation Method	REML
Residual Variance Method	None
Fixed Effects SE Method	Model-Based
Degrees of Freedom Method	Between-Within

Class Level Information		
Class	Levels	Values
RP	24	1 2 3 4 5 6 7 8 9 10 11 12 13 14 15 16 17 18 19 20 21 22 23 24
TEN	20	1 10 11 12 13 14 15 16 17 18 19 2 20 3 4 5 6 7 8 9
ANI	2	c s

Dimensions	
Covariance Parameters	3
Columns in X	63
Columns in Z	0
Subjects	439
Max Obs per Subject	1

Number of Observations	
Number of Observations Read	439
Number of Observations Used	434
Number of Observations Not Used	5

Iteration History			
Iteration	Evaluations	-2 Res Log Like	Criterion
0	1	4142.84756616	
1	1	4140.67663342	0.00000000

Convergence criteria met but final Hessian is not positive definite.

Estimated R Matrix for RP(TEN) 1 1	
Row	Col1
1	1864.58

The Mixed Procedure

Estimated R Correlation Matrix for RP(TEN) 1 1	
**Row**	**Col1**
**1**	1.0000

Covariance Parameter Estimates		
**Cov Parm**	**Subject**	**Estimate**
**UN(1,1)**	RP(TEN)	1864.58
**UN(2,1)**	RP(TEN)	0
**UN(2,2)**	RP(TEN)	1510.47

Fit Statistics	
**-2 Res Log Likelihood**	4140.7
**AIC (Smaller is Better)**	4146.7
**AICC (Smaller is Better)**	4146.7
**BIC (Smaller is Better)**	4158.9

Null Model Likelihood Ratio Test		
**DF**	**Chi-Square**	**Pr > ChiSq**
2	2.17	0.3377

Type 3 Tests of Fixed Effects				
**Effect**	**Num DF**	**Den DF**	**F Value**	**Pr > F**
**TEN**	19	394	0.42	0.9861
**ANI**	1	394	31.86	<.0001
**TEN*ANI**	19	394	0.45	0.9793

The Mixed Procedure

Model Information	
**Data Set**	WORK.AU
**Dependent Variable**	Y
**Covariance Structure**	Unstructured using Correlations
**Subject Effect**	RP(TEN)
**Estimation Method**	REML
**Residual Variance Method**	None
**Fixed Effects SE Method**	Model-Based
**Degrees of Freedom Method**	Between-Within

Class Level Information		
**Class**	**Levels**	**Values**
**RP**	24	1 2 3 4 5 6 7 8 9 10 11 12 13 14 15 16 17 18 19 20 21 22 23 24
**TEN**	20	1 10 11 12 13 14 15 16 17 18 19 2 20 3 4 5 6 7 8 9
**ANI**	2	c s

Dimensions	
**Covariance Parameters**	3
**Columns in X**	63
**Columns in Z**	0
**Subjects**	439
**Max Obs per Subject**	1

Number of Observations	
**Number of Observations Read**	439
**Number of Observations Used**	434
**Number of Observations Not Used**	5

Iteration History			
**Iteration**	**Evaluations**	**-2 Res Log Like**	**Criterion**
**0**	1	4142.84756616	
**1**	2	4140.75409301	0.00000000

Convergence criteria met but final Hessian is not positive definite.

Estimated R Matrix for RP(TEN) 1 1	
**Row**	**Col1**
**1**	1828.11

The Mixed Procedure

Estimated R Correlation Matrix for RP(TEN) 1 1	
**Row**	**Col1**
**1**	1.0000

Covariance Parameter Estimates		
**Cov Parm**	**Subject**	**Estimate**
**Var(1)**	RP(TEN)	1828.11
**Var(2)**	RP(TEN)	1480.89
**Corr(2,1)**	RP(TEN)	0

Fit Statistics	
**-2 Res Log Likelihood**	4140.8
**AIC (Smaller is Better)**	4146.8
**AICC (Smaller is Better)**	4146.8
**BIC (Smaller is Better)**	4159.0

Null Model Likelihood Ratio Test		
**DF**	**Chi-Square**	**Pr > ChiSq**
2	2.09	0.3511

Type 3 Tests of Fixed Effects				
**Effect**	**Num DF**	**Den DF**	**F Value**	**Pr > F**
**TEN**	19	394	0.43	0.9844
**ANI**	1	394	32.50	<.0001
**TEN*ANI**	19	394	0.46	0.9769

The Mixed Procedure

Model Information	
**Data Set**	WORK.AU
**Dependent Variable**	Y
**Covariance Structure**	Variance Components
**Subject Effect**	RP(TEN)
**Estimation Method**	REML
**Residual Variance Method**	Parameter
**Fixed Effects SE Method**	Model-Based
**Degrees of Freedom Method**	Between-Within

Class Level Information		
**Class**	**Levels**	**Values**
**RP**	24	1 2 3 4 5 6 7 8 9 10 11 12 13 14 15 16 17 18 19 20 21 22 23 24
**TEN**	20	1 10 11 12 13 14 15 16 17 18 19 2 20 3 4 5 6 7 8 9
**ANI**	2	c s

Dimensions	
**Covariance Parameters**	1
**Columns in X**	63
**Columns in Z**	0
**Subjects**	439
**Max Obs per Subject**	1

Number of Observations	
**Number of Observations Read**	439
**Number of Observations Used**	434
**Number of Observations Not Used**	5

Iteration History			
**Iteration**	**Evaluations**	**-2 Res Log Like**	**Criterion**
**0**	1	4142.84756616	
**1**	1	4142.84756616	0.00000000

Convergence criteria met.

Estimated R Matrix for RP(TEN) 1 1	
**Row**	**Col1**
**1**	1694.72

The Mixed Procedure

Estimated R Correlation Matrix for RP(TEN) 1 1	
**Row**	**Col1**
**1**	1.0000

Covariance Parameter Estimates		
**Cov Parm**	**Subject**	**Estimate**
**ANI**	RP(TEN)	1694.72

Fit Statistics	
**-2 Res Log Likelihood**	4142.8
**AIC (Smaller is Better)**	4144.8
**AICC (Smaller is Better)**	4144.9
**BIC (Smaller is Better)**	4148.9

Null Model Likelihood Ratio Test		
**DF**	**Chi-Square**	**Pr > ChiSq**
0	0.00	1.0000

Type 3 Tests of Fixed Effects				
**Effect**	**Num DF**	**Den DF**	**F Value**	**Pr > F**
**TEN**	19	394	0.42	0.9867
**ANI**	1	394	31.59	<.0001
**TEN*ANI**	19	394	0.45	0.9803

Obs	Descr	V_ANTE	V_AR	V_ARH	V_ARMA	V_CS	V_CSH	V_FA1
1	-2 Res Log Likelihood	4140.8	4142.8	4140.8	4142.8	4142.8	4140.8	4140.7
2	AIC (Smaller is Better)	4146.8	4146.8	4146.8	4148.8	4146.8	4146.8	4148.7
3	AICC (Smaller is Better)	4146.8	4146.9	4146.8	4148.9	4146.9	4146.8	4148.8
4	BIC (Smaller is Better)	4159.0	4155.0	4159.0	4161.1	4155.0	4159.0	4165.0

Obs	V_HF	V_SIMPLE	V_TOEP	V_TOEPH	V_UN	V_UNR	V_VC
1	4140.7	4142.8	4142.8	4140.8	4140.7	4140.8	4142.8
2	4146.7	4144.8	4146.8	4146.8	4146.7	4146.8	4144.8
3	4146.7	4144.9	4146.9	4146.8	4146.7	4146.8	4144.9
4	4158.9	4148.9	4155.0	4159.0	4158.9	4159.0	4148.9

Obs	ANI	TEN	RP	Y
**1**	c	1	1	70
**2**	c	2	1	95
**3**	c	3	1	95
**4**	c	4	1	90
**5**	c	5	1	95
**6**	c	6	1	95
**7**	c	7	1	95
**8**	c	8	1	95
**9**	c	9	1	45
**10**	c	10	1	95
**11**	c	11	1	95
**12**	c	12	1	95
**13**	c	13	1	95
**14**	c	14	1	95
**15**	c	15	1	95
**16**	c	16	1	95
**17**	c	17	1	95
**18**	c	18	1	85
**19**	c	19	1	0
**20**	c	20	1	95
**21**	c	1	2	60
**22**	c	2	2	90
**23**	c	3	2	40
**24**	c	4	2	60
**25**	c	5	2	95
**26**	c	6	2	80
**27**	c	7	2	95
**28**	c	8	2	95
**29**	c	9	2	95
**30**	c	10	2	95
**31**	c	11	2	95
**32**	c	12	2	85
**33**	c	13	2	95
**34**	c	14	2	95
**35**	c	15	2	90
**36**	c	16	2	85
**37**	c	17	2	70
**38**	c	18	2	95
**39**	c	19	2	50
**40**	c	20	2	95
**41**	c	1	3	0

Obs	ANI	TEN	RP	Y
42	c	2	3	95
43	c	3	3	0
44	c	4	3	95
45	c	5	3	95
46	c	6	3	0
47	c	7	3	95
48	c	8	3	0
49	c	9	3	0
50	c	10	3	50
51	c	11	3	100
52	c	12	3	100
53	c	13	3	5
54	c	14	3	30
55	c	15	3	95
56	c	16	3	40
57	c	17	3	95
58	c	18	3	90
59	c	19	3	30
60	c	20	3	20
61	c	1	4	45
62	c	2	4	60
63	c	3	4	60
64	c	4	4	0
65	c	5	4	10
66	c	6	4	0
67	c	7	4	45
68	c	8	4	95
69	c	9	4	0
70	c	10	4	40
71	c	11	4	95
72	c	12	4	90
73	c	13	4	0
74	c	14	4	95
75	c	15	4	15
76	c	16	4	100
77	c	17	4	60
78	c	18	4	50
79	c	19	4	50
80	c	20	4	10
81	c	1	5	78
82	c	2	5	95

Obs	ANI	TEN	RP	Y
**83**	c	3	5	95
**84**	c	4	5	97
**85**	c	5	5	95
**86**	c	6	5	78
**87**	c	7	5	78
**88**	c	8	5	78
**89**	c	9	5	78
**90**	c	10	5	95
**91**	c	11	5	98
**92**	c	12	5	78
**93**	c	13	5	78
**94**	c	14	5	78
**95**	c	15	5	78
**96**	c	16	5	78
**97**	c	17	5	78
**98**	c	18	5	78
**99**	c	19	5	78
**100**	c	20	5	78
**101**	c	1	6	78
**102**	c	2	6	78
**103**	c	3	6	78
**104**	c	4	6	78
**105**	c	5	6	0
**106**	c	6	6	78
**107**	c	7	6	78
**108**	c	8	6	95
**109**	c	9	6	78
**110**	c	10	6	0
**111**	c	11	6	95
**112**	c	12	6	95
**113**	c	13	6	95
**114**	c	14	6	12
**115**	c	15	6	12
**116**	c	16	6	0
**117**	c	17	6	0
**118**	c	18	6	2
**119**	c	19	6	95
**120**	c	20	6	95
**121**	c	1	7	12
**122**	c	2	7	0
**123**	c	3	7	0

Obs	ANI	TEN	RP	Y
**124**	c	4	7	0
**125**	c	5	7	0
**126**	c	6	7	95
**127**	c	7	7	95
**128**	c	8	7	12
**129**	c	9	7	0
**130**	c	10	7	12
**131**	c	11	7	0
**132**	c	12	7	0
**133**	c	13	7	0
**134**	c	14	7	95
**135**	c	15	7	0
**136**	c	16	7	95
**137**	c	17	7	95
**138**	c	18	7	12
**139**	c	19	7	0
**140**	c	20	7	12
**141**	c	1	8	0
**142**	c	2	8	0
**143**	c	3	8	4
**144**	c	4	8	12
**145**	c	5	8	95
**146**	c	6	8	12
**147**	c	7	8	0
**148**	c	8	8	0
**149**	c	9	8	10
**150**	c	10	8	10
**151**	c	11	8	0
**152**	c	12	8	0
**153**	c	13	8	0
**154**	c	14	8	0
**155**	c	15	8	95
**156**	c	16	8	95
**157**	c	17	8	0
**158**	c	18	8	0
**159**	c	19	8	95
**160**	c	20	8	95
**161**	c	1	9	95
**162**	c	2	9	12
**163**	c	3	9	95
**164**	c	4	9	95

Obs	ANI	TEN	RP	Y
**165**	c	5	9	12
**166**	c	6	9	95
**167**	c	7	9	95
**168**	c	8	9	95
**169**	c	9	9	95
**170**	c	10	9	2
**171**	c	11	9	0
**172**	c	12	9	0
**173**	c	13	9	92
**174**	c	14	9	0
**175**	c	15	9	0
**176**	c	16	9	0
**177**	c	17	9	0
**178**	c	18	9	0
**179**	c	19	9	0
**180**	c	20	9	0
**181**	c	4	10	0
**182**	c	5	10	100
**183**	c	6	10	0
**184**	c	7	10	0
**185**	c	8	10	50
**186**	c	9	10	0
**187**	c	10	10	0
**188**	c	11	10	.
**189**	c	13	10	.
**190**	c	15	10	.
**191**	c	17	10	.
**192**	c	19	10	.
**193**	c	1	11	0
**194**	c	2	11	0
**195**	c	3	11	100
**196**	c	4	11	0
**197**	c	6	11	100
**198**	c	7	11	100
**199**	c	8	11	0
**200**	c	9	11	0
**201**	c	10	11	100
**202**	c	11	11	100
**203**	c	12	11	100
**204**	c	13	11	100
**205**	c	14	11	100

Obs	ANI	TEN	RP	Y
**206**	c	15	11	100
**207**	c	16	11	50
**208**	c	17	11	100
**209**	c	18	11	80
**210**	c	19	11	100
**211**	c	20	11	100
**212**	c	1	12	100
**213**	c	2	12	40
**214**	c	3	12	70
**215**	c	4	12	90
**216**	c	5	12	40
**217**	c	6	12	100
**218**	c	7	12	100
**219**	c	8	12	100
**220**	c	9	12	100
**221**	c	10	12	100
**222**	c	11	12	0
**223**	c	12	12	0
**224**	c	13	12	50
**225**	c	15	12	0
**226**	c	16	12	100
**227**	c	17	12	100
**228**	c	18	12	40
**229**	c	19	12	0
**230**	c	20	12	0
**231**	s	1	13	0
**232**	s	2	13	100
**233**	s	3	13	100
**234**	s	5	13	100
**235**	s	6	13	100
**236**	s	7	13	0
**237**	s	10	13	95
**238**	s	12	13	95
**239**	s	13	13	95
**240**	s	14	13	95
**241**	s	15	13	100
**242**	s	16	13	95
**243**	s	17	13	100
**244**	s	18	13	100
**245**	s	19	13	95
**246**	s	20	13	100

Obs	ANI	TEN	RP	Y
247	s	1	14	100
248	s	3	14	100
249	s	4	14	100
250	s	5	14	100
251	s	8	14	100
252	s	9	14	100
253	s	10	14	0
254	s	11	14	100
255	s	12	14	100
256	s	13	14	60
257	s	14	14	100
258	s	15	14	100
259	s	16	14	95
260	s	17	14	50
261	s	18	14	100
262	s	19	14	100
263	s	20	14	0
264	s	1	15	95
265	s	2	15	0
266	s	3	15	100
267	s	4	15	100
268	s	6	15	100
269	s	7	15	100
270	s	8	15	100
271	s	9	15	100
272	s	10	15	100
273	s	11	15	100
274	s	12	15	0
275	s	13	15	0
276	s	14	15	100
277	s	16	15	100
278	s	17	15	100
279	s	18	15	100
280	s	19	15	0
281	s	20	15	30
282	s	1	16	100
283	s	2	16	100
284	s	3	16	70
285	s	4	16	100
286	s	5	16	30
287	s	6	16	100

Obs	ANI	TEN	RP	Y
288	s	7	16	100
289	s	8	16	100
290	s	9	16	50
291	s	10	16	100
292	s	12	16	0
293	s	14	16	0
294	s	15	16	50
295	s	16	16	100
296	s	17	16	100
297	s	18	16	100
298	s	19	16	100
299	s	1	17	0
300	s	2	17	0
301	s	3	17	100
302	s	4	17	100
303	s	5	17	60
304	s	6	17	0
305	s	7	17	100
306	s	9	17	100
307	s	10	17	100
308	s	11	17	100
309	s	12	17	40
310	s	13	17	95
311	s	14	17	100
312	s	15	17	0
313	s	16	17	100
314	s	17	17	95
315	s	18	17	40
316	s	19	17	100
317	s	20	17	95
318	s	1	18	100
319	s	2	18	0
320	s	3	18	0
321	s	4	18	0
322	s	5	18	100
323	s	6	18	100
324	s	7	18	0
325	s	8	18	0
326	s	9	18	100
327	s	10	18	100
328	s	11	18	0

Obs	ANI	TEN	RP	Y
**329**	s	12	18	0
**330**	s	13	18	100
**331**	s	14	18	0
**332**	s	15	18	100
**333**	s	16	18	0
**334**	s	17	18	0
**335**	s	18	18	100
**336**	s	19	18	0
**337**	s	1	19	100
**338**	s	2	19	100
**339**	s	4	19	100
**340**	s	5	19	100
**341**	s	6	19	0
**342**	s	7	19	100
**343**	s	8	19	100
**344**	s	9	19	100
**345**	s	11	19	100
**346**	s	12	19	100
**347**	s	13	19	100
**348**	s	16	19	100
**349**	s	17	19	100
**350**	s	19	19	100
**351**	s	1	20	100
**352**	s	2	20	50
**353**	s	3	20	90
**354**	s	4	20	95
**355**	s	5	20	100
**356**	s	7	20	100
**357**	s	8	20	50
**358**	s	9	20	60
**359**	s	10	20	95
**360**	s	12	20	0
**361**	s	13	20	95
**362**	s	14	20	95
**363**	s	15	20	95
**364**	s	16	20	100
**365**	s	17	20	95
**366**	s	19	20	100
**367**	s	20	20	100
**368**	s	1	21	100
**369**	s	3	21	100

Obs	ANI	TEN	RP	Y
**370**	s	4	21	100
**371**	s	5	21	0
**372**	s	7	21	100
**373**	s	8	21	100
**374**	s	9	21	100
**375**	s	10	21	100
**376**	s	11	21	100
**377**	s	12	21	100
**378**	s	13	21	95
**379**	s	14	21	100
**380**	s	15	21	100
**381**	s	16	21	30
**382**	s	17	21	30
**383**	s	19	21	100
**384**	s	20	21	100
**385**	s	1	22	100
**386**	s	2	22	60
**387**	s	3	22	100
**388**	s	4	22	100
**389**	s	5	22	100
**390**	s	6	22	95
**391**	s	7	22	5
**392**	s	8	22	100
**393**	s	9	22	0
**394**	s	10	22	0
**395**	s	11	22	95
**396**	s	12	22	100
**397**	s	13	22	100
**398**	s	14	22	0
**399**	s	15	22	95
**400**	s	17	22	100
**401**	s	18	22	100
**402**	s	19	22	95
**403**	s	20	22	0
**404**	s	1	23	0
**405**	s	2	23	100
**406**	s	4	23	95
**407**	s	5	23	100
**408**	s	6	23	100
**409**	s	7	23	100
**410**	s	8	23	50

Obs	ANI	TEN	RP	Y
**411**	s	9	23	95
**412**	s	10	23	100
**413**	s	11	23	100
**414**	s	12	23	100
**415**	s	13	23	100
**416**	s	14	23	90
**417**	s	15	23	100
**418**	s	16	23	100
**419**	s	17	23	100
**420**	s	18	23	100
**421**	s	20	23	100
**422**	s	2	24	95
**423**	s	4	24	95
**424**	s	5	24	95
**425**	s	6	24	100
**426**	s	7	24	100
**427**	s	8	24	100
**428**	s	9	24	50
**429**	s	10	24	0
**430**	s	11	24	100
**431**	s	12	24	100
**432**	s	13	24	100
**433**	s	14	24	100
**434**	s	15	24	100
**435**	s	16	24	100
**436**	s	17	24	100
**437**	s	18	24	100
**438**	s	19	24	100
**439**	s	20	24	100

The Mixed Procedure

Model Information	
**Data Set**	WORK.AU
**Dependent Variable**	Y
**Covariance Structure**	Variance Components
**Subject Effect**	RP(TEN*ANI)
**Estimation Method**	REML
**Residual Variance Method**	Parameter
**Fixed Effects SE Method**	Model-Based
**Degrees of Freedom Method**	Between-Within

Class Level Information		
**Class**	**Levels**	**Values**
**TEN**	20	1 10 11 12 13 14 15 16 17 18 19 2 20 3 4 5 6 7 8 9
**ANI**	2	c s
**RP**	24	1 2 3 4 5 6 7 8 9 10 11 12 13 14 15 16 17 18 19 20 21 22 23 24

Dimensions	
**Covariance Parameters**	1
**Columns in X**	63
**Columns in Z**	0
**Subjects**	439
**Max Obs per Subject**	1

Number of Observations	
**Number of Observations Read**	439
**Number of Observations Used**	434
**Number of Observations Not Used**	5

Iteration History			
**Iteration**	**Evaluations**	**-2 Res Log Like**	**Criterion**
**0**	1	4142.84756616	
**1**	1	4142.84756616	0.00000000

Convergence criteria met.

Estimated R Matrix for RP(TEN*ANI) 1 1 c	
**Row**	**Col1**
**1**	1694.72

The Mixed Procedure

Estimated R Correlation Matrix for RP(TEN*ANI) 1 1 c	
**Row**	**Col1**
**1**	1.0000

Covariance Parameter Estimates		
**Cov Parm**	**Subject**	**Estimate**
**ANI**	RP(TEN*ANI)	1694.72

Fit Statistics	
**-2 Res Log Likelihood**	4142.8
**AIC (Smaller is Better)**	4144.8
**AICC (Smaller is Better)**	4144.9
**BIC (Smaller is Better)**	4148.9

Null Model Likelihood Ratio Test		
**DF**	**Chi-Square**	**Pr > ChiSq**
0	0.00	1.0000

Type 3 Tests of Fixed Effects				
**Effect**	**Num DF**	**Den DF**	**F Value**	**Pr > F**
**TEN**	19	394	0.42	0.9867
**ANI**	1	394	31.59	<.0001
**TEN*ANI**	19	394	0.45	0.9803

The Mixed Procedure

Least Squares Means							
Effect	TEN	ANI	Estimate	Standard Error	DF	t Value	Pr > |t|
TEN	1		60.5909	8.7768	394	6.90	<.0001
TEN	10		60.8674	8.5920	394	7.08	<.0001
TEN	11		74.9848	9.2516	394	8.11	<.0001
TEN	12		59.8523	8.5920	394	6.97	<.0001
TEN	13		70.4545	8.7768	394	8.03	<.0001
TEN	14		65.4545	8.9936	394	7.28	<.0001
TEN	15		68.3636	8.9936	394	7.60	<.0001
TEN	16		75.3636	8.7768	394	8.59	<.0001
TEN	17		71.9167	8.5920	394	8.37	<.0001
TEN	18		70.8485	9.2516	394	7.66	<.0001
TEN	19		63.0909	8.7768	394	7.19	<.0001
TEN	2		55.9318	8.9936	394	6.22	<.0001
TEN	20		61.9949	9.2516	394	6.70	<.0001
TEN	3		71.1768	9.2516	394	7.69	<.0001
TEN	4		70.4811	8.5920	394	8.20	<.0001
TEN	5		69.1818	8.7768	394	7.88	<.0001
TEN	6		69.1528	9.0765	394	7.62	<.0001
TEN	7		73.0909	8.5920	394	8.51	<.0001
TEN	8		69.7917	8.8133	394	7.92	<.0001
TEN	9		59.7386	8.5920	394	6.95	<.0001
ANI		c	55.9693	2.7476	394	20.37	<.0001
ANI		s	78.2635	2.8605	394	27.36	<.0001
TEN*ANI	1	c	48.9091	12.4123	394	3.94	<.0001
TEN*ANI	1	s	72.2727	12.4123	394	5.82	<.0001
TEN*ANI	10	c	49.9167	11.8839	394	4.20	<.0001
TEN*ANI	10	s	71.8182	12.4123	394	5.79	<.0001
TEN*ANI	11	c	61.6364	12.4123	394	4.97	<.0001
TEN*ANI	11	s	88.3333	13.7223	394	6.44	<.0001
TEN*ANI	12	c	58.4545	12.4123	394	4.71	<.0001
TEN*ANI	12	s	61.2500	11.8839	394	5.15	<.0001
TEN*ANI	13	c	55.4545	12.4123	394	4.47	<.0001
TEN*ANI	13	s	85.4545	12.4123	394	6.88	<.0001
TEN*ANI	14	c	60.0000	13.0181	394	4.61	<.0001
TEN*ANI	14	s	70.9091	12.4123	394	5.71	<.0001
TEN*ANI	15	c	52.7273	12.4123	394	4.25	<.0001
TEN*ANI	15	s	84.0000	13.0181	394	6.45	<.0001
TEN*ANI	16	c	67.0909	12.4123	394	5.41	<.0001
TEN*ANI	16	s	83.6364	12.4123	394	6.74	<.0001

The Mixed Procedure

Least Squares Means							
**Effect**	**TEN**	**ANI**	**Estimate**	**Standard Error**	**DF**	**t Value**	**Pr > |t|**
**TEN*ANI**	17	c	63.0000	12.4123	394	5.08	<.0001
**TEN*ANI**	17	s	80.8333	11.8839	394	6.80	<.0001
**TEN*ANI**	18	c	48.3636	12.4123	394	3.90	0.0001
**TEN*ANI**	18	s	93.3333	13.7223	394	6.80	<.0001
**TEN*ANI**	19	c	45.2727	12.4123	394	3.65	0.0003
**TEN*ANI**	19	s	80.9091	12.4123	394	6.52	<.0001
**TEN*ANI**	2	c	51.3636	12.4123	394	4.14	<.0001
**TEN*ANI**	2	s	60.5000	13.0181	394	4.65	<.0001
**TEN*ANI**	20	c	54.5455	12.4123	394	4.39	<.0001
**TEN*ANI**	20	s	69.4444	13.7223	394	5.06	<.0001
**TEN*ANI**	3	c	57.9091	12.4123	394	4.67	<.0001
**TEN*ANI**	3	s	84.4444	13.7223	394	6.15	<.0001
**TEN*ANI**	4	c	51.4167	11.8839	394	4.33	<.0001
**TEN*ANI**	4	s	89.5455	12.4123	394	7.21	<.0001
**TEN*ANI**	5	c	57.9091	12.4123	394	4.67	<.0001
**TEN*ANI**	5	s	80.4545	12.4123	394	6.48	<.0001
**TEN*ANI**	6	c	61.0833	11.8839	394	5.14	<.0001
**TEN*ANI**	6	s	77.2222	13.7223	394	5.63	<.0001
**TEN*ANI**	7	c	73.0000	11.8839	394	6.14	<.0001
**TEN*ANI**	7	s	73.1818	12.4123	394	5.90	<.0001
**TEN*ANI**	8	c	59.5833	11.8839	394	5.01	<.0001
**TEN*ANI**	8	s	80.0000	13.0181	394	6.15	<.0001
**TEN*ANI**	9	c	41.7500	11.8839	394	3.51	0.0005
**TEN*ANI**	9	s	77.7273	12.4123	394	6.26	<.0001
**TEN**	1		60.5909	8.7768	394	6.90	<.0001
**TEN**	10		60.8674	8.5920	394	7.08	<.0001
**TEN**	11		74.9848	9.2516	394	8.11	<.0001
**TEN**	12		59.8523	8.5920	394	6.97	<.0001
**TEN**	13		70.4545	8.7768	394	8.03	<.0001
**TEN**	14		65.4545	8.9936	394	7.28	<.0001
**TEN**	15		68.3636	8.9936	394	7.60	<.0001
**TEN**	16		75.3636	8.7768	394	8.59	<.0001
**TEN**	17		71.9167	8.5920	394	8.37	<.0001
**TEN**	18		70.8485	9.2516	394	7.66	<.0001
**TEN**	19		63.0909	8.7768	394	7.19	<.0001
**TEN**	2		55.9318	8.9936	394	6.22	<.0001
**TEN**	20		61.9949	9.2516	394	6.70	<.0001
**TEN**	3		71.1768	9.2516	394	7.69	<.0001

The Mixed Procedure

Least Squares Means							
Effect	TEN	ANI	Estimate	Standard Error	DF	t Value	Pr > |t|
TEN	4		70.4811	8.5920	394	8.20	<.0001
TEN	5		69.1818	8.7768	394	7.88	<.0001
TEN	6		69.1528	9.0765	394	7.62	<.0001
TEN	7		73.0909	8.5920	394	8.51	<.0001
TEN	8		69.7917	8.8133	394	7.92	<.0001
TEN	9		59.7386	8.5920	394	6.95	<.0001
ANI		c	55.9693	2.7476	394	20.37	<.0001
ANI		s	78.2635	2.8605	394	27.36	<.0001
TEN*ANI	1	c	48.9091	12.4123	394	3.94	<.0001
TEN*ANI	1	s	72.2727	12.4123	394	5.82	<.0001
TEN*ANI	10	c	49.9167	11.8839	394	4.20	<.0001
TEN*ANI	10	s	71.8182	12.4123	394	5.79	<.0001
TEN*ANI	11	c	61.6364	12.4123	394	4.97	<.0001
TEN*ANI	11	s	88.3333	13.7223	394	6.44	<.0001
TEN*ANI	12	c	58.4545	12.4123	394	4.71	<.0001
TEN*ANI	12	s	61.2500	11.8839	394	5.15	<.0001
TEN*ANI	13	c	55.4545	12.4123	394	4.47	<.0001
TEN*ANI	13	s	85.4545	12.4123	394	6.88	<.0001
TEN*ANI	14	c	60.0000	13.0181	394	4.61	<.0001
TEN*ANI	14	s	70.9091	12.4123	394	5.71	<.0001
TEN*ANI	15	c	52.7273	12.4123	394	4.25	<.0001
TEN*ANI	15	s	84.0000	13.0181	394	6.45	<.0001
TEN*ANI	16	c	67.0909	12.4123	394	5.41	<.0001
TEN*ANI	16	s	83.6364	12.4123	394	6.74	<.0001
TEN*ANI	17	c	63.0000	12.4123	394	5.08	<.0001
TEN*ANI	17	s	80.8333	11.8839	394	6.80	<.0001
TEN*ANI	18	c	48.3636	12.4123	394	3.90	0.0001
TEN*ANI	18	s	93.3333	13.7223	394	6.80	<.0001
TEN*ANI	19	c	45.2727	12.4123	394	3.65	0.0003
TEN*ANI	19	s	80.9091	12.4123	394	6.52	<.0001
TEN*ANI	2	c	51.3636	12.4123	394	4.14	<.0001
TEN*ANI	2	s	60.5000	13.0181	394	4.65	<.0001
TEN*ANI	20	c	54.5455	12.4123	394	4.39	<.0001
TEN*ANI	20	s	69.4444	13.7223	394	5.06	<.0001
TEN*ANI	3	c	57.9091	12.4123	394	4.67	<.0001
TEN*ANI	3	s	84.4444	13.7223	394	6.15	<.0001
TEN*ANI	4	c	51.4167	11.8839	394	4.33	<.0001
TEN*ANI	4	s	89.5455	12.4123	394	7.21	<.0001

The Mixed Procedure

Least Squares Means							
**Effect**	**TEN**	**ANI**	**Estimate**	**Standard Error**	**DF**	**t Value**	**Pr > |t|**
**TEN*ANI**	5	c	57.9091	12.4123	394	4.67	<.0001
**TEN*ANI**	5	s	80.4545	12.4123	394	6.48	<.0001
**TEN*ANI**	6	c	61.0833	11.8839	394	5.14	<.0001
**TEN*ANI**	6	s	77.2222	13.7223	394	5.63	<.0001
**TEN*ANI**	7	c	73.0000	11.8839	394	6.14	<.0001
**TEN*ANI**	7	s	73.1818	12.4123	394	5.90	<.0001
**TEN*ANI**	8	c	59.5833	11.8839	394	5.01	<.0001
**TEN*ANI**	8	s	80.0000	13.0181	394	6.15	<.0001
**TEN*ANI**	9	c	41.7500	11.8839	394	3.51	0.0005
**TEN*ANI**	9	s	77.7273	12.4123	394	6.26	<.0001
**TEN*ANI**	1	c	48.9091	12.4123	394	3.94	<.0001
**TEN*ANI**	1	s	72.2727	12.4123	394	5.82	<.0001
**TEN*ANI**	10	c	49.9167	11.8839	394	4.20	<.0001
**TEN*ANI**	10	s	71.8182	12.4123	394	5.79	<.0001
**TEN*ANI**	11	c	61.6364	12.4123	394	4.97	<.0001
**TEN*ANI**	11	s	88.3333	13.7223	394	6.44	<.0001
**TEN*ANI**	12	c	58.4545	12.4123	394	4.71	<.0001
**TEN*ANI**	12	s	61.2500	11.8839	394	5.15	<.0001
**TEN*ANI**	13	c	55.4545	12.4123	394	4.47	<.0001
**TEN*ANI**	13	s	85.4545	12.4123	394	6.88	<.0001
**TEN*ANI**	14	c	60.0000	13.0181	394	4.61	<.0001
**TEN*ANI**	14	s	70.9091	12.4123	394	5.71	<.0001
**TEN*ANI**	15	c	52.7273	12.4123	394	4.25	<.0001
**TEN*ANI**	15	s	84.0000	13.0181	394	6.45	<.0001
**TEN*ANI**	16	c	67.0909	12.4123	394	5.41	<.0001
**TEN*ANI**	16	s	83.6364	12.4123	394	6.74	<.0001
**TEN*ANI**	17	c	63.0000	12.4123	394	5.08	<.0001
**TEN*ANI**	17	s	80.8333	11.8839	394	6.80	<.0001
**TEN*ANI**	18	c	48.3636	12.4123	394	3.90	0.0001
**TEN*ANI**	18	s	93.3333	13.7223	394	6.80	<.0001
**TEN*ANI**	19	c	45.2727	12.4123	394	3.65	0.0003
**TEN*ANI**	19	s	80.9091	12.4123	394	6.52	<.0001
**TEN*ANI**	2	c	51.3636	12.4123	394	4.14	<.0001
**TEN*ANI**	2	s	60.5000	13.0181	394	4.65	<.0001
**TEN*ANI**	20	c	54.5455	12.4123	394	4.39	<.0001
**TEN*ANI**	20	s	69.4444	13.7223	394	5.06	<.0001
**TEN*ANI**	3	c	57.9091	12.4123	394	4.67	<.0001
**TEN*ANI**	3	s	84.4444	13.7223	394	6.15	<.0001

The Mixed Procedure

Least Squares Means							
Effect	TEN	ANI	Estimate	Standard Error	DF	t Value	Pr > |t|
**TEN*ANI**	4	c	51.4167	11.8839	394	4.33	<.0001
**TEN*ANI**	4	s	89.5455	12.4123	394	7.21	<.0001
**TEN*ANI**	5	c	57.9091	12.4123	394	4.67	<.0001
**TEN*ANI**	5	s	80.4545	12.4123	394	6.48	<.0001
**TEN*ANI**	6	c	61.0833	11.8839	394	5.14	<.0001
**TEN*ANI**	6	s	77.2222	13.7223	394	5.63	<.0001
**TEN*ANI**	7	c	73.0000	11.8839	394	6.14	<.0001
**TEN*ANI**	7	s	73.1818	12.4123	394	5.90	<.0001
**TEN*ANI**	8	c	59.5833	11.8839	394	5.01	<.0001
**TEN*ANI**	8	s	80.0000	13.0181	394	6.15	<.0001
**TEN*ANI**	9	c	41.7500	11.8839	394	3.51	0.0005
**TEN*ANI**	9	s	77.7273	12.4123	394	6.26	<.0001

Tests of Effect Slices						
Effect	TEN	ANI	Num DF	Den DF	F Value	Pr > F
**TEN*ANI**	1		1	394	1.77	0.1840
**TEN*ANI**	10		1	394	1.62	0.2032
**TEN*ANI**	11		1	394	2.08	0.1499
**TEN*ANI**	12		1	394	0.03	0.8709
**TEN*ANI**	13		1	394	2.92	0.0882
**TEN*ANI**	14		1	394	0.37	0.5445
**TEN*ANI**	15		1	394	3.02	0.0829
**TEN*ANI**	16		1	394	0.89	0.3465
**TEN*ANI**	17		1	394	1.08	0.3000
**TEN*ANI**	18		1	394	5.91	0.0155
**TEN*ANI**	19		1	394	4.12	0.0430
**TEN*ANI**	2		1	394	0.26	0.6118
**TEN*ANI**	20		1	394	0.65	0.4212
**TEN*ANI**	3		1	394	2.06	0.1523
**TEN*ANI**	4		1	394	4.92	0.0271
**TEN*ANI**	5		1	394	1.65	0.1998
**TEN*ANI**	6		1	394	0.79	0.3745
**TEN*ANI**	7		1	394	0.00	0.9916
**TEN*ANI**	8		1	394	1.34	0.2474
**TEN*ANI**	9		1	394	4.38	0.0369
**TEN*ANI**		c	19	394	0.39	0.9915
**TEN*ANI**		s	19	394	0.47	0.9723
