# Supplementary material for: Learning curve for the laparoscopy-guided kidney biopsy procedure in small corpses of dogs and pigs
Source: PLoS One. 2021 Sep 27;16(9):e0257653. doi: 10.1371/journal.pone.0257653 (PMC8475998; doi:10.1371/journal.pone.0257653)
Supplement: S2 Raw data — (PDF) [file pone.0257653.s002.pdf]

Obs	ANI	TEN	RP	Y
1	c	1	1	17
2	c	2	1	31
3	c	3	1	46
4	c	4	1	46
5	c	5	1	35
6	c	6	1	10
7	c	7	1	43
8	c	8	1	26
9	c	9	1	32
10	c	10	1	149
11	c	11	1	28
12	c	12	1	28
13	c	13	1	16
14	c	14	1	40
15	c	15	1	24
16	c	16	1	14
17	c	17	1	12
18	c	18	1	32
19	c	19	1	0
20	c	20	1	42
21	c	1	2	24
22	c	2	2	44
23	c	3	2	14
24	c	4	2	19
25	c	5	2	69
26	c	6	2	46
27	c	7	2	48
28	c	8	2	10
29	c	9	2	5
30	c	10	2	132
31	c	11	2	37
32	c	12	2	30
33	c	13	2	26
34	c	14	2	59
35	c	15	2	52
36	c	16	2	24
37	c	17	2	26
38	c	18	2	25
39	c	19	2	16
40	c	20	2	48
41	c	1	3	0

Obs	ANI	TEN	RP	Y
42	c	2	3	16
43	c	3	3	0
44	c	4	3	12
45	c	5	3	38
46	c	6	3	0
47	c	7	3	4
48	c	8	3	0
49	c	9	3	0
50	c	10	3	36
51	c	11	3	10
52	c	12	3	2
53	c	13	3	4
54	c	14	3	6
55	c	15	3	32
56	c	16	3	8
57	c	17	3	14
58	c	18	3	18
59	c	19	3	8
60	c	20	3	18
61	c	1	4	14
62	c	2	4	6
63	c	3	4	26
64	c	4	4	0
65	c	5	4	2
66	c	6	4	0
67	c	7	4	16
68	c	8	4	15
69	c	9	4	0
70	c	10	4	12
71	c	11	4	38
72	c	12	4	12
73	c	13	4	0
74	c	14	4	56
75	c	15	4	4
76	c	16	4	19
77	c	17	4	12
78	c	18	4	14
79	c	19	4	10
80	c	20	4	30
81	c	1	5	11
82	c	2	5	28

Obs	ANI	TEN	RP	Y
83	c	3	5	28
84	c	4	5	30
85	c	5	5	20
86	c	6	5	8
87	c	7	5	15
88	c	8	5	12
89	c	9	5	18
90	c	10	5	22
91	c	11	5	31
92	c	12	5	5
93	c	13	5	7
94	c	14	5	12
95	c	15	5	10
96	c	16	5	3
97	c	17	5	6
98	c	18	5	3
99	c	19	5	4
100	c	20	5	19
101	c	1	6	7
102	c	2	6	2
103	c	3	6	2
104	c	4	6	8
105	c	5	6	0
106	c	6	6	4
107	c	7	6	6
108	c	8	6	21
109	c	9	6	11
110	c	10	6	0
111	c	11	6	24
112	c	12	6	15
113	c	13	6	10
114	c	14	6	3
115	c	15	6	3
116	c	16	6	0
117	c	17	6	0
118	c	18	6	1
119	c	19	6	15
120	c	20	6	13
121	c	1	7	5
122	c	2	7	0
123	c	3	7	0

Obs	ANI	TEN	RP	Y
124	c	4	7	0
125	c	5	7	0
126	c	6	7	11
127	c	7	7	15
128	c	8	7	5
129	c	9	7	0
130	c	10	7	3
131	c	11	7	0
132	c	12	7	0
133	c	13	7	0
134	c	14	7	20
135	c	15	7	0
136	c	16	7	11
137	c	17	7	15
138	c	18	7	5
139	c	19	7	0
140	c	20	7	3
141	c	1	8	0
142	c	2	8	0
143	c	3	8	1
144	c	4	8	8
145	c	5	8	18
146	c	6	8	6
147	c	7	8	0
148	c	8	8	0
149	c	9	8	0
150	c	10	8	0
151	c	11	8	0
152	c	12	8	0
153	c	13	8	0
154	c	14	8	0
155	c	15	8	15
156	c	16	8	14
157	c	17	8	0
158	c	18	8	0
159	c	19	8	36
160	c	20	8	24
161	c	1	9	32
162	c	2	9	4
163	c	3	9	35
164	c	4	9	54

Obs	ANI	TEN	RP	Y
**165**	c	5	9	6
**166**	c	6	9	11
**167**	c	7	9	37
**168**	c	8	9	12
**169**	c	9	9	24
**170**	c	10	9	2
**171**	c	11	9	0
**172**	c	12	9	0
**173**	c	13	9	12
**174**	c	14	9	0
**175**	c	15	9	0
**176**	c	16	9	0
**177**	c	17	9	0
**178**	c	18	9	0
**179**	c	19	9	0
**180**	c	20	9	0
**181**	c	4	10	0
**182**	c	5	10	11
**183**	c	6	10	0
**184**	c	7	10	0
**185**	c	8	10	7
**186**	c	9	10	0
**187**	c	10	10	0
**188**	c	1	11	0
**189**	c	2	11	0
**190**	c	3	11	17
**191**	c	4	11	0
**192**	c	6	11	11
**193**	c	7	11	8
**194**	c	8	11	0
**195**	c	9	11	0
**196**	c	10	11	3
**197**	c	11	11	21
**198**	c	12	11	47
**199**	c	13	11	26
**200**	c	14	11	13
**201**	c	15	11	29
**202**	c	16	11	20
**203**	c	17	11	21
**204**	c	18	11	24
**205**	c	19	11	28

Obs	ANI	TEN	RP	Y
**206**	c	20	11	7
**207**	c	1	12	13
**208**	c	2	12	16
**209**	c	3	12	18
**210**	c	4	12	26
**211**	c	5	12	20
**212**	c	6	12	40
**213**	c	7	12	49
**214**	c	8	12	21
**215**	c	9	12	27
**216**	c	10	12	9
**217**	c	11	12	0
**218**	c	12	12	0
**219**	c	13	12	16
**220**	c	15	12	0
**221**	c	16	12	21
**222**	c	17	12	3
**223**	c	18	12	15
**224**	c	19	12	0
**225**	c	20	12	0
**226**	s	1	13	0
**227**	s	2	13	18
**228**	s	3	13	13
**229**	s	5	13	29
**230**	s	6	13	20
**231**	s	7	13	0
**232**	s	10	13	20
**233**	s	12	13	18
**234**	s	13	13	32
**235**	s	14	13	31
**236**	s	15	13	22
**237**	s	16	13	17
**238**	s	17	13	24
**239**	s	18	13	18
**240**	s	19	13	29
**241**	s	20	13	24
**242**	s	1	14	36
**243**	s	3	14	10
**244**	s	4	14	45
**245**	s	5	14	0
**246**	s	8	14	36

Obs	ANI	TEN	RP	Y
247	s	9	14	14
248	s	10	14	0
249	s	11	14	39
250	s	12	14	35
251	s	13	14	8
252	s	14	14	15
253	s	15	14	4
254	s	16	14	13
255	s	17	14	7
256	s	18	14	6
257	s	19	14	8
258	s	20	14	0
259	s	1	15	13
260	s	2	15	0
261	s	3	15	6
262	s	4	15	9
263	s	6	15	14
264	s	7	15	1
265	s	8	15	21
266	s	9	15	11
267	s	10	15	1
268	s	11	15	10
269	s	12	15	0
270	s	13	15	0
271	s	14	15	22
272	s	16	15	14
273	s	17	15	13
274	s	18	15	9
275	s	19	15	0
276	s	20	15	3
277	s	1	16	26
278	s	2	16	14
279	s	3	16	14
280	s	4	16	4
281	s	5	16	3
282	s	6	16	16
283	s	7	16	17
284	s	8	16	16
285	s	9	16	25
286	s	10	16	16
287	s	12	16	0

Obs	ANI	TEN	RP	Y
**288**	s	14	16	0
**289**	s	15	16	7
**290**	s	16	16	3
**291**	s	17	16	6
**292**	s	18	16	27
**293**	s	19	16	6
**294**	s	1	17	0
**295**	s	2	17	0
**296**	s	3	17	4
**297**	s	4	17	22
**298**	s	5	17	24
**299**	s	6	17	0
**300**	s	7	17	4
**301**	s	9	17	3
**302**	s	10	17	5
**303**	s	11	17	39
**304**	s	12	17	4
**305**	s	13	17	34
**306**	s	14	17	59
**307**	s	15	17	0
**308**	s	16	17	14
**309**	s	17	17	26
**310**	s	18	17	12
**311**	s	19	17	47
**312**	s	20	17	18
**313**	s	1	18	4
**314**	s	2	18	0
**315**	s	3	18	0
**316**	s	4	18	0
**317**	s	5	18	17
**318**	s	6	18	26
**319**	s	7	18	0
**320**	s	8	18	0
**321**	s	9	18	17
**322**	s	10	18	31
**323**	s	11	18	0
**324**	s	12	18	0
**325**	s	13	18	9
**326**	s	14	18	0
**327**	s	15	18	4
**328**	s	16	18	0

Obs	ANI	TEN	RP	Y
**329**	s	17	18	0
**330**	s	18	18	15
**331**	s	19	18	0
**332**	s	1	19	21
**333**	s	2	19	10
**334**	s	4	19	25
**335**	s	5	19	20
**336**	s	6	19	0
**337**	s	7	19	11
**338**	s	8	19	10
**339**	s	9	19	6
**340**	s	11	19	15
**341**	s	12	19	7
**342**	s	13	19	16
**343**	s	15	19	0
**344**	s	16	19	10
**345**	s	17	19	15
**346**	s	19	19	1
**347**	s	20	19	0
**348**	s	1	20	0
**349**	s	2	20	9
**350**	s	3	20	9
**351**	s	4	20	8
**352**	s	5	20	20
**353**	s	7	20	22
**354**	s	8	20	4
**355**	s	9	20	10
**356**	s	10	20	13
**357**	s	12	20	0
**358**	s	13	20	9
**359**	s	14	20	3
**360**	s	15	20	15
**361**	s	16	20	13
**362**	s	17	20	8
**363**	s	19	20	7
**364**	s	20	20	25
**365**	s	1	21	15
**366**	s	3	21	13
**367**	s	4	21	4
**368**	s	5	21	0
**369**	s	7	21	14

Obs	ANI	TEN	RP	Y
**370**	s	8	21	4
**371**	s	9	21	20
**372**	s	10	21	4
**373**	s	11	21	8
**374**	s	12	21	21
**375**	s	13	21	12
**376**	s	14	21	22
**377**	s	15	21	6
**378**	s	16	21	0
**379**	s	17	21	4
**380**	s	19	21	16
**381**	s	20	21	8
**382**	s	1	22	9
**383**	s	2	22	10
**384**	s	3	22	6
**385**	s	4	22	6
**386**	s	5	22	2
**387**	s	6	22	11
**388**	s	7	22	2
**389**	s	8	22	5
**390**	s	9	22	0
**391**	s	10	22	0
**392**	s	11	22	9
**393**	s	12	22	4
**394**	s	13	22	15
**395**	s	14	22	0
**396**	s	15	22	10
**397**	s	17	22	20
**398**	s	18	22	9
**399**	s	19	22	11
**400**	s	20	22	0
**401**	s	1	23	0
**402**	s	2	23	9
**403**	s	4	23	39
**404**	s	5	23	4
**405**	s	6	23	9
**406**	s	7	23	18
**407**	s	8	23	2
**408**	s	9	23	16
**409**	s	10	23	8
**410**	s	11	23	21

Obs	ANI	TEN	RP	Y
411	s	12	23	19
412	s	13	23	16
413	s	14	23	7
414	s	15	23	12
415	s	16	23	12
416	s	17	23	4
417	s	18	23	26
418	s	20	23	11
419	s	2	24	11
420	s	4	24	16
421	s	5	24	5
422	s	6	24	4
423	s	7	24	3
424	s	8	24	18
425	s	9	24	1
426	s	10	24	0
427	s	11	24	10
428	s	12	24	14
429	s	13	24	18
430	s	14	24	15
431	s	15	24	21
432	s	16	24	30
433	s	17	24	18
434	s	18	24	12
435	s	19	24	7
436	s	20	24	30

Obs	ANI	TEN	RP	Y
1	c	1	1	17
2	c	2	1	31
3	c	3	1	46
4	c	4	1	46
5	c	5	1	35
6	c	6	1	10
7	c	7	1	43
8	c	8	1	26
9	c	9	1	32
10	c	10	1	149
11	c	11	1	28
12	c	12	1	28
13	c	13	1	16
14	c	14	1	40
15	c	15	1	24
16	c	16	1	14
17	c	17	1	12
18	c	18	1	32
19	c	19	1	0
20	c	20	1	42
21	c	1	2	24
22	c	2	2	44
23	c	3	2	14
24	c	4	2	19
25	c	5	2	69
26	c	6	2	46
27	c	7	2	48
28	c	8	2	10
29	c	9	2	5
30	c	10	2	132
31	c	11	2	37
32	c	12	2	30
33	c	13	2	26
34	c	14	2	59
35	c	15	2	52
36	c	16	2	24
37	c	17	2	26
38	c	18	2	25
39	c	19	2	16
40	c	20	2	48
41	c	1	3	0

Obs	ANI	TEN	RP	Y
42	c	2	3	16
43	c	3	3	0
44	c	4	3	12
45	c	5	3	38
46	c	6	3	0
47	c	7	3	4
48	c	8	3	0
49	c	9	3	0
50	c	10	3	36
51	c	11	3	10
52	c	12	3	2
53	c	13	3	4
54	c	14	3	6
55	c	15	3	32
56	c	16	3	8
57	c	17	3	14
58	c	18	3	18
59	c	19	3	8
60	c	20	3	18
61	c	1	4	14
62	c	2	4	6
63	c	3	4	26
64	c	4	4	0
65	c	5	4	2
66	c	6	4	0
67	c	7	4	16
68	c	8	4	15
69	c	9	4	0
70	c	10	4	12
71	c	11	4	38
72	c	12	4	12
73	c	13	4	0
74	c	14	4	56
75	c	15	4	4
76	c	16	4	19
77	c	17	4	12
78	c	18	4	14
79	c	19	4	10
80	c	20	4	30
81	c	1	5	11
82	c	2	5	28

Obs	ANI	TEN	RP	Y
83	c	3	5	28
84	c	4	5	30
85	c	5	5	20
86	c	6	5	8
87	c	7	5	15
88	c	8	5	12
89	c	9	5	18
90	c	10	5	22
91	c	11	5	31
92	c	12	5	5
93	c	13	5	7
94	c	14	5	12
95	c	15	5	10
96	c	16	5	3
97	c	17	5	6
98	c	18	5	3
99	c	19	5	4
100	c	20	5	19
101	c	1	6	7
102	c	2	6	2
103	c	3	6	2
104	c	4	6	8
105	c	5	6	0
106	c	6	6	4
107	c	7	6	6
108	c	8	6	21
109	c	9	6	11
110	c	10	6	0
111	c	11	6	24
112	c	12	6	15
113	c	13	6	10
114	c	14	6	3
115	c	15	6	3
116	c	16	6	0
117	c	17	6	0
118	c	18	6	1
119	c	19	6	15
120	c	20	6	13
121	c	1	7	5
122	c	2	7	0
123	c	3	7	0

Obs	ANI	TEN	RP	Y
**124**	c	4	7	0
**125**	c	5	7	0
**126**	c	6	7	11
**127**	c	7	7	15
**128**	c	8	7	5
**129**	c	9	7	0
**130**	c	10	7	3
**131**	c	11	7	0
**132**	c	12	7	0
**133**	c	13	7	0
**134**	c	14	7	20
**135**	c	15	7	0
**136**	c	16	7	11
**137**	c	17	7	15
**138**	c	18	7	5
**139**	c	19	7	0
**140**	c	20	7	3
**141**	c	1	8	0
**142**	c	2	8	0
**143**	c	3	8	1
**144**	c	4	8	8
**145**	c	5	8	18
**146**	c	6	8	6
**147**	c	7	8	0
**148**	c	8	8	0
**149**	c	9	8	0
**150**	c	10	8	0
**151**	c	11	8	0
**152**	c	12	8	0
**153**	c	13	8	0
**154**	c	14	8	0
**155**	c	15	8	15
**156**	c	16	8	14
**157**	c	17	8	0
**158**	c	18	8	0
**159**	c	19	8	36
**160**	c	20	8	24
**161**	c	1	9	32
**162**	c	2	9	4
**163**	c	3	9	35
**164**	c	4	9	54

Obs	ANI	TEN	RP	Y
165	c	5	9	6
166	c	6	9	11
167	c	7	9	37
168	c	8	9	12
169	c	9	9	24
170	c	10	9	2
171	c	11	9	0
172	c	12	9	0
173	c	13	9	12
174	c	14	9	0
175	c	15	9	0
176	c	16	9	0
177	c	17	9	0
178	c	18	9	0
179	c	19	9	0
180	c	20	9	0
181	c	4	10	0
182	c	5	10	11
183	c	6	10	0
184	c	7	10	0
185	c	8	10	7
186	c	9	10	0
187	c	10	10	0
188	c	1	11	0
189	c	2	11	0
190	c	3	11	17
191	c	4	11	0
192	c	6	11	11
193	c	7	11	8
194	c	8	11	0
195	c	9	11	0
196	c	10	11	3
197	c	11	11	21
198	c	12	11	47
199	c	13	11	26
200	c	14	11	13
201	c	15	11	29
202	c	16	11	20
203	c	17	11	21
204	c	18	11	24
205	c	19	11	28

Obs	ANI	TEN	RP	Y
206	c	20	11	7
207	c	1	12	13
208	c	2	12	16
209	c	3	12	18
210	c	4	12	26
211	c	5	12	20
212	c	6	12	40
213	c	7	12	49
214	c	8	12	21
215	c	9	12	27
216	c	10	12	9
217	c	11	12	0
218	c	12	12	0
219	c	13	12	16
220	c	15	12	0
221	c	16	12	21
222	c	17	12	3
223	c	18	12	15
224	c	19	12	0
225	c	20	12	0
226	s	1	13	0
227	s	2	13	18
228	s	3	13	13
229	s	5	13	29
230	s	6	13	20
231	s	7	13	0
232	s	10	13	20
233	s	12	13	18
234	s	13	13	32
235	s	14	13	31
236	s	15	13	22
237	s	16	13	17
238	s	17	13	24
239	s	18	13	18
240	s	19	13	29
241	s	20	13	24
242	s	1	14	36
243	s	3	14	10
244	s	4	14	45
245	s	5	14	0
246	s	8	14	36

Obs	ANI	TEN	RP	Y
247	s	9	14	14
248	s	10	14	0
249	s	11	14	39
250	s	12	14	35
251	s	13	14	8
252	s	14	14	15
253	s	15	14	4
254	s	16	14	13
255	s	17	14	7
256	s	18	14	6
257	s	19	14	8
258	s	20	14	0
259	s	1	15	13
260	s	2	15	0
261	s	3	15	6
262	s	4	15	9
263	s	6	15	14
264	s	7	15	1
265	s	8	15	21
266	s	9	15	11
267	s	10	15	1
268	s	11	15	10
269	s	12	15	0
270	s	13	15	0
271	s	14	15	22
272	s	16	15	14
273	s	17	15	13
274	s	18	15	9
275	s	19	15	0
276	s	20	15	3
277	s	1	16	26
278	s	2	16	14
279	s	3	16	14
280	s	4	16	4
281	s	5	16	3
282	s	6	16	16
283	s	7	16	17
284	s	8	16	16
285	s	9	16	25
286	s	10	16	16
287	s	12	16	0

Obs	ANI	TEN	RP	Y
288	s	14	16	0
289	s	15	16	7
290	s	16	16	3
291	s	17	16	6
292	s	18	16	27
293	s	19	16	6
294	s	1	17	0
295	s	2	17	0
296	s	3	17	4
297	s	4	17	22
298	s	5	17	24
299	s	6	17	0
300	s	7	17	4
301	s	9	17	3
302	s	10	17	5
303	s	11	17	39
304	s	12	17	4
305	s	13	17	34
306	s	14	17	59
307	s	15	17	0
308	s	16	17	14
309	s	17	17	26
310	s	18	17	12
311	s	19	17	47
312	s	20	17	18
313	s	1	18	4
314	s	2	18	0
315	s	3	18	0
316	s	4	18	0
317	s	5	18	17
318	s	6	18	26
319	s	7	18	0
320	s	8	18	0
321	s	9	18	17
322	s	10	18	31
323	s	11	18	0
324	s	12	18	0
325	s	13	18	9
326	s	14	18	0
327	s	15	18	4
328	s	16	18	0

Obs	ANI	TEN	RP	Y
**329**	s	17	18	0
**330**	s	18	18	15
**331**	s	19	18	0
**332**	s	1	19	21
**333**	s	2	19	10
**334**	s	4	19	25
**335**	s	5	19	20
**336**	s	6	19	0
**337**	s	7	19	11
**338**	s	8	19	10
**339**	s	9	19	6
**340**	s	11	19	15
**341**	s	12	19	7
**342**	s	13	19	16
**343**	s	15	19	0
**344**	s	16	19	10
**345**	s	17	19	15
**346**	s	19	19	1
**347**	s	20	19	0
**348**	s	1	20	0
**349**	s	2	20	9
**350**	s	3	20	9
**351**	s	4	20	8
**352**	s	5	20	20
**353**	s	7	20	22
**354**	s	8	20	4
**355**	s	9	20	10
**356**	s	10	20	13
**357**	s	12	20	0
**358**	s	13	20	9
**359**	s	14	20	3
**360**	s	15	20	15
**361**	s	16	20	13
**362**	s	17	20	8
**363**	s	19	20	7
**364**	s	20	20	25
**365**	s	1	21	15
**366**	s	3	21	13
**367**	s	4	21	4
**368**	s	5	21	0
**369**	s	7	21	14

Obs	ANI	TEN	RP	Y
**370**	s	8	21	4
**371**	s	9	21	20
**372**	s	10	21	4
**373**	s	11	21	8
**374**	s	12	21	21
**375**	s	13	21	12
**376**	s	14	21	22
**377**	s	15	21	6
**378**	s	16	21	0
**379**	s	17	21	4
**380**	s	19	21	16
**381**	s	20	21	8
**382**	s	1	22	9
**383**	s	2	22	10
**384**	s	3	22	6
**385**	s	4	22	6
**386**	s	5	22	2
**387**	s	6	22	11
**388**	s	7	22	2
**389**	s	8	22	5
**390**	s	9	22	0
**391**	s	10	22	0
**392**	s	11	22	9
**393**	s	12	22	4
**394**	s	13	22	15
**395**	s	14	22	0
**396**	s	15	22	10
**397**	s	17	22	20
**398**	s	18	22	9
**399**	s	19	22	11
**400**	s	20	22	0
**401**	s	1	23	0
**402**	s	2	23	9
**403**	s	4	23	39
**404**	s	5	23	4
**405**	s	6	23	9
**406**	s	7	23	18
**407**	s	8	23	2
**408**	s	9	23	16
**409**	s	10	23	8
**410**	s	11	23	21

Obs	ANI	TEN	RP	Y
411	s	12	23	19
412	s	13	23	16
413	s	14	23	7
414	s	15	23	12
415	s	16	23	12
416	s	17	23	4
417	s	18	23	26
418	s	20	23	11
419	s	2	24	11
420	s	4	24	16
421	s	5	24	5
422	s	6	24	4
423	s	7	24	3
424	s	8	24	18
425	s	9	24	1
426	s	10	24	0
427	s	11	24	10
428	s	12	24	14
429	s	13	24	18
430	s	14	24	15
431	s	15	24	21
432	s	16	24	30
433	s	17	24	18
434	s	18	24	12
435	s	19	24	7
436	s	20	24	30

Obs	ANI	TEN	RP	Y
1	c	1	1	17
2	c	2	1	31
3	c	3	1	46
4	c	4	1	46
5	c	5	1	35
6	c	6	1	10
7	c	7	1	43
8	c	8	1	26
9	c	9	1	32
10	c	10	1	149
11	c	11	1	28
12	c	12	1	28
13	c	13	1	16
14	c	14	1	40
15	c	15	1	24
16	c	16	1	14
17	c	17	1	12
18	c	18	1	32
19	c	19	1	0
20	c	20	1	42
21	c	1	2	24
22	c	2	2	44
23	c	3	2	14
24	c	4	2	19
25	c	5	2	69
26	c	6	2	46
27	c	7	2	48
28	c	8	2	10
29	c	9	2	5
30	c	10	2	132
31	c	11	2	37
32	c	12	2	30
33	c	13	2	26
34	c	14	2	59
35	c	15	2	52
36	c	16	2	24
37	c	17	2	26
38	c	18	2	25
39	c	19	2	16
40	c	20	2	48
41	c	1	3	0

Obs	ANI	TEN	RP	Y
42	c	2	3	16
43	c	3	3	0
44	c	4	3	12
45	c	5	3	38
46	c	6	3	0
47	c	7	3	4
48	c	8	3	0
49	c	9	3	0
50	c	10	3	36
51	c	11	3	10
52	c	12	3	2
53	c	13	3	4
54	c	14	3	6
55	c	15	3	32
56	c	16	3	8
57	c	17	3	14
58	c	18	3	18
59	c	19	3	8
60	c	20	3	18
61	c	1	4	14
62	c	2	4	6
63	c	3	4	26
64	c	4	4	0
65	c	5	4	2
66	c	6	4	0
67	c	7	4	16
68	c	8	4	15
69	c	9	4	0
70	c	10	4	12
71	c	11	4	38
72	c	12	4	12
73	c	13	4	0
74	c	14	4	56
75	c	15	4	4
76	c	16	4	19
77	c	17	4	12
78	c	18	4	14
79	c	19	4	10
80	c	20	4	30
81	c	1	5	11
82	c	2	5	28

Obs	ANI	TEN	RP	Y
**83**	c	3	5	28
**84**	c	4	5	30
**85**	c	5	5	20
**86**	c	6	5	8
**87**	c	7	5	15
**88**	c	8	5	12
**89**	c	9	5	18
**90**	c	10	5	22
**91**	c	11	5	31
**92**	c	12	5	5
**93**	c	13	5	7
**94**	c	14	5	12
**95**	c	15	5	10
**96**	c	16	5	3
**97**	c	17	5	6
**98**	c	18	5	3
**99**	c	19	5	4
**100**	c	20	5	19
**101**	c	1	6	7
**102**	c	2	6	2
**103**	c	3	6	2
**104**	c	4	6	8
**105**	c	5	6	0
**106**	c	6	6	4
**107**	c	7	6	6
**108**	c	8	6	21
**109**	c	9	6	11
**110**	c	10	6	0
**111**	c	11	6	24
**112**	c	12	6	15
**113**	c	13	6	10
**114**	c	14	6	3
**115**	c	15	6	3
**116**	c	16	6	0
**117**	c	17	6	0
**118**	c	18	6	1
**119**	c	19	6	15
**120**	c	20	6	13
**121**	c	1	7	5
**122**	c	2	7	0
**123**	c	3	7	0

Obs	ANI	TEN	RP	Y
124	c	4	7	0
125	c	5	7	0
126	c	6	7	11
127	c	7	7	15
128	c	8	7	5
129	c	9	7	0
130	c	10	7	3
131	c	11	7	0
132	c	12	7	0
133	c	13	7	0
134	c	14	7	20
135	c	15	7	0
136	c	16	7	11
137	c	17	7	15
138	c	18	7	5
139	c	19	7	0
140	c	20	7	3
141	c	1	8	0
142	c	2	8	0
143	c	3	8	1
144	c	4	8	8
145	c	5	8	18
146	c	6	8	6
147	c	7	8	0
148	c	8	8	0
149	c	9	8	0
150	c	10	8	0
151	c	11	8	0
152	c	12	8	0
153	c	13	8	0
154	c	14	8	0
155	c	15	8	15
156	c	16	8	14
157	c	17	8	0
158	c	18	8	0
159	c	19	8	36
160	c	20	8	24
161	c	1	9	32
162	c	2	9	4
163	c	3	9	35
164	c	4	9	54

Obs	ANI	TEN	RP	Y
**165**	c	5	9	6
**166**	c	6	9	11
**167**	c	7	9	37
**168**	c	8	9	12
**169**	c	9	9	24
**170**	c	10	9	2
**171**	c	11	9	0
**172**	c	12	9	0
**173**	c	13	9	12
**174**	c	14	9	0
**175**	c	15	9	0
**176**	c	16	9	0
**177**	c	17	9	0
**178**	c	18	9	0
**179**	c	19	9	0
**180**	c	20	9	0
**181**	c	4	10	0
**182**	c	5	10	11
**183**	c	6	10	0
**184**	c	7	10	0
**185**	c	8	10	7
**186**	c	9	10	0
**187**	c	10	10	0
**188**	c	1	11	0
**189**	c	2	11	0
**190**	c	3	11	17
**191**	c	4	11	0
**192**	c	6	11	11
**193**	c	7	11	8
**194**	c	8	11	0
**195**	c	9	11	0
**196**	c	10	11	3
**197**	c	11	11	21
**198**	c	12	11	47
**199**	c	13	11	26
**200**	c	14	11	13
**201**	c	15	11	29
**202**	c	16	11	20
**203**	c	17	11	21
**204**	c	18	11	24
**205**	c	19	11	28

Obs	ANI	TEN	RP	Y
206	c	20	11	7
207	c	1	12	13
208	c	2	12	16
209	c	3	12	18
210	c	4	12	26
211	c	5	12	20
212	c	6	12	40
213	c	7	12	49
214	c	8	12	21
215	c	9	12	27
216	c	10	12	9
217	c	11	12	0
218	c	12	12	0
219	c	13	12	16
220	c	15	12	0
221	c	16	12	21
222	c	17	12	3
223	c	18	12	15
224	c	19	12	0
225	c	20	12	0
226	s	1	13	0
227	s	2	13	18
228	s	3	13	13
229	s	5	13	29
230	s	6	13	20
231	s	7	13	0
232	s	10	13	20
233	s	12	13	18
234	s	13	13	32
235	s	14	13	31
236	s	15	13	22
237	s	16	13	17
238	s	17	13	24
239	s	18	13	18
240	s	19	13	29
241	s	20	13	24
242	s	1	14	36
243	s	3	14	10
244	s	4	14	45
245	s	5	14	0
246	s	8	14	36

Obs	ANI	TEN	RP	Y
**247**	s	9	14	14
**248**	s	10	14	0
**249**	s	11	14	39
**250**	s	12	14	35
**251**	s	13	14	8
**252**	s	14	14	15
**253**	s	15	14	4
**254**	s	16	14	13
**255**	s	17	14	7
**256**	s	18	14	6
**257**	s	19	14	8
**258**	s	20	14	0
**259**	s	1	15	13
**260**	s	2	15	0
**261**	s	3	15	6
**262**	s	4	15	9
**263**	s	6	15	14
**264**	s	7	15	1
**265**	s	8	15	21
**266**	s	9	15	11
**267**	s	10	15	1
**268**	s	11	15	10
**269**	s	12	15	0
**270**	s	13	15	0
**271**	s	14	15	22
**272**	s	16	15	14
**273**	s	17	15	13
**274**	s	18	15	9
**275**	s	19	15	0
**276**	s	20	15	3
**277**	s	1	16	26
**278**	s	2	16	14
**279**	s	3	16	14
**280**	s	4	16	4
**281**	s	5	16	3
**282**	s	6	16	16
**283**	s	7	16	17
**284**	s	8	16	16
**285**	s	9	16	25
**286**	s	10	16	16
**287**	s	12	16	0

Obs	ANI	TEN	RP	Y
**288**	s	14	16	0
**289**	s	15	16	7
**290**	s	16	16	3
**291**	s	17	16	6
**292**	s	18	16	27
**293**	s	19	16	6
**294**	s	1	17	0
**295**	s	2	17	0
**296**	s	3	17	4
**297**	s	4	17	22
**298**	s	5	17	24
**299**	s	6	17	0
**300**	s	7	17	4
**301**	s	9	17	3
**302**	s	10	17	5
**303**	s	11	17	39
**304**	s	12	17	4
**305**	s	13	17	34
**306**	s	14	17	59
**307**	s	15	17	0
**308**	s	16	17	14
**309**	s	17	17	26
**310**	s	18	17	12
**311**	s	19	17	47
**312**	s	20	17	18
**313**	s	1	18	4
**314**	s	2	18	0
**315**	s	3	18	0
**316**	s	4	18	0
**317**	s	5	18	17
**318**	s	6	18	26
**319**	s	7	18	0
**320**	s	8	18	0
**321**	s	9	18	17
**322**	s	10	18	31
**323**	s	11	18	0
**324**	s	12	18	0
**325**	s	13	18	9
**326**	s	14	18	0
**327**	s	15	18	4
**328**	s	16	18	0

Obs	ANI	TEN	RP	Y
**329**	s	17	18	0
**330**	s	18	18	15
**331**	s	19	18	0
**332**	s	1	19	21
**333**	s	2	19	10
**334**	s	4	19	25
**335**	s	5	19	20
**336**	s	6	19	0
**337**	s	7	19	11
**338**	s	8	19	10
**339**	s	9	19	6
**340**	s	11	19	15
**341**	s	12	19	7
**342**	s	13	19	16
**343**	s	15	19	0
**344**	s	16	19	10
**345**	s	17	19	15
**346**	s	19	19	1
**347**	s	20	19	0
**348**	s	1	20	0
**349**	s	2	20	9
**350**	s	3	20	9
**351**	s	4	20	8
**352**	s	5	20	20
**353**	s	7	20	22
**354**	s	8	20	4
**355**	s	9	20	10
**356**	s	10	20	13
**357**	s	12	20	0
**358**	s	13	20	9
**359**	s	14	20	3
**360**	s	15	20	15
**361**	s	16	20	13
**362**	s	17	20	8
**363**	s	19	20	7
**364**	s	20	20	25
**365**	s	1	21	15
**366**	s	3	21	13
**367**	s	4	21	4
**368**	s	5	21	0
**369**	s	7	21	14

Obs	ANI	TEN	RP	Y
370	s	8	21	4
371	s	9	21	20
372	s	10	21	4
373	s	11	21	8
374	s	12	21	21
375	s	13	21	12
376	s	14	21	22
377	s	15	21	6
378	s	16	21	0
379	s	17	21	4
380	s	19	21	16
381	s	20	21	8
382	s	1	22	9
383	s	2	22	10
384	s	3	22	6
385	s	4	22	6
386	s	5	22	2
387	s	6	22	11
388	s	7	22	2
389	s	8	22	5
390	s	9	22	0
391	s	10	22	0
392	s	11	22	9
393	s	12	22	4
394	s	13	22	15
395	s	14	22	0
396	s	15	22	10
397	s	17	22	20
398	s	18	22	9
399	s	19	22	11
400	s	20	22	0
401	s	1	23	0
402	s	2	23	9
403	s	4	23	39
404	s	5	23	4
405	s	6	23	9
406	s	7	23	18
407	s	8	23	2
408	s	9	23	16
409	s	10	23	8
410	s	11	23	21

Obs	ANI	TEN	RP	Y
**411**	s	12	23	19
**412**	s	13	23	16
**413**	s	14	23	7
**414**	s	15	23	12
**415**	s	16	23	12
**416**	s	17	23	4
**417**	s	18	23	26
**418**	s	20	23	11
**419**	s	2	24	11
**420**	s	4	24	16
**421**	s	5	24	5
**422**	s	6	24	4
**423**	s	7	24	3
**424**	s	8	24	18
**425**	s	9	24	1
**426**	s	10	24	0
**427**	s	11	24	10
**428**	s	12	24	14
**429**	s	13	24	18
**430**	s	14	24	15
**431**	s	15	24	21
**432**	s	16	24	30
**433**	s	17	24	18
**434**	s	18	24	12
**435**	s	19	24	7
**436**	s	20	24	30

Obs	ANI	TEN	RP	Y
1	c	1	1	17
2	c	2	1	31
3	c	3	1	46
4	c	4	1	46
5	c	5	1	35
6	c	6	1	10
7	c	7	1	43
8	c	8	1	26
9	c	9	1	32
10	c	10	1	149
11	c	11	1	28
12	c	12	1	28
13	c	13	1	16
14	c	14	1	40
15	c	15	1	24
16	c	16	1	14
17	c	17	1	12
18	c	18	1	32
19	c	19	1	0
20	c	20	1	42
21	c	1	2	24
22	c	2	2	44
23	c	3	2	14
24	c	4	2	19
25	c	5	2	69
26	c	6	2	46
27	c	7	2	48
28	c	8	2	10
29	c	9	2	5
30	c	10	2	132
31	c	11	2	37
32	c	12	2	30
33	c	13	2	26
34	c	14	2	59
35	c	15	2	52
36	c	16	2	24
37	c	17	2	26
38	c	18	2	25
39	c	19	2	16
40	c	20	2	48
41	c	1	3	0

Obs	ANI	TEN	RP	Y
**42**	c	2	3	16
**43**	c	3	3	0
**44**	c	4	3	12
**45**	c	5	3	38
**46**	c	6	3	0
**47**	c	7	3	4
**48**	c	8	3	0
**49**	c	9	3	0
**50**	c	10	3	36
**51**	c	11	3	10
**52**	c	12	3	2
**53**	c	13	3	4
**54**	c	14	3	6
**55**	c	15	3	32
**56**	c	16	3	8
**57**	c	17	3	14
**58**	c	18	3	18
**59**	c	19	3	8
**60**	c	20	3	18
**61**	c	1	4	14
**62**	c	2	4	6
**63**	c	3	4	26
**64**	c	4	4	0
**65**	c	5	4	2
**66**	c	6	4	0
**67**	c	7	4	16
**68**	c	8	4	15
**69**	c	9	4	0
**70**	c	10	4	12
**71**	c	11	4	38
**72**	c	12	4	12
**73**	c	13	4	0
**74**	c	14	4	56
**75**	c	15	4	4
**76**	c	16	4	19
**77**	c	17	4	12
**78**	c	18	4	14
**79**	c	19	4	10
**80**	c	20	4	30
**81**	c	1	5	11
**82**	c	2	5	28

Obs	ANI	TEN	RP	Y
83	c	3	5	28
84	c	4	5	30
85	c	5	5	20
86	c	6	5	8
87	c	7	5	15
88	c	8	5	12
89	c	9	5	18
90	c	10	5	22
91	c	11	5	31
92	c	12	5	5
93	c	13	5	7
94	c	14	5	12
95	c	15	5	10
96	c	16	5	3
97	c	17	5	6
98	c	18	5	3
99	c	19	5	4
100	c	20	5	19
101	c	1	6	7
102	c	2	6	2
103	c	3	6	2
104	c	4	6	8
105	c	5	6	0
106	c	6	6	4
107	c	7	6	6
108	c	8	6	21
109	c	9	6	11
110	c	10	6	0
111	c	11	6	24
112	c	12	6	15
113	c	13	6	10
114	c	14	6	3
115	c	15	6	3
116	c	16	6	0
117	c	17	6	0
118	c	18	6	1
119	c	19	6	15
120	c	20	6	13
121	c	1	7	5
122	c	2	7	0
123	c	3	7	0

Obs	ANI	TEN	RP	Y
124	c	4	7	0
125	c	5	7	0
126	c	6	7	11
127	c	7	7	15
128	c	8	7	5
129	c	9	7	0
130	c	10	7	3
131	c	11	7	0
132	c	12	7	0
133	c	13	7	0
134	c	14	7	20
135	c	15	7	0
136	c	16	7	11
137	c	17	7	15
138	c	18	7	5
139	c	19	7	0
140	c	20	7	3
141	c	1	8	0
142	c	2	8	0
143	c	3	8	1
144	c	4	8	8
145	c	5	8	18
146	c	6	8	6
147	c	7	8	0
148	c	8	8	0
149	c	9	8	0
150	c	10	8	0
151	c	11	8	0
152	c	12	8	0
153	c	13	8	0
154	c	14	8	0
155	c	15	8	15
156	c	16	8	14
157	c	17	8	0
158	c	18	8	0
159	c	19	8	36
160	c	20	8	24
161	c	1	9	32
162	c	2	9	4
163	c	3	9	35
164	c	4	9	54

Obs	ANI	TEN	RP	Y
**165**	c	5	9	6
**166**	c	6	9	11
**167**	c	7	9	37
**168**	c	8	9	12
**169**	c	9	9	24
**170**	c	10	9	2
**171**	c	11	9	0
**172**	c	12	9	0
**173**	c	13	9	12
**174**	c	14	9	0
**175**	c	15	9	0
**176**	c	16	9	0
**177**	c	17	9	0
**178**	c	18	9	0
**179**	c	19	9	0
**180**	c	20	9	0
**181**	c	4	10	0
**182**	c	5	10	11
**183**	c	6	10	0
**184**	c	7	10	0
**185**	c	8	10	7
**186**	c	9	10	0
**187**	c	10	10	0
**188**	c	1	11	0
**189**	c	2	11	0
**190**	c	3	11	17
**191**	c	4	11	0
**192**	c	6	11	11
**193**	c	7	11	8
**194**	c	8	11	0
**195**	c	9	11	0
**196**	c	10	11	3
**197**	c	11	11	21
**198**	c	12	11	47
**199**	c	13	11	26
**200**	c	14	11	13
**201**	c	15	11	29
**202**	c	16	11	20
**203**	c	17	11	21
**204**	c	18	11	24
**205**	c	19	11	28

Obs	ANI	TEN	RP	Y
206	c	20	11	7
207	c	1	12	13
208	c	2	12	16
209	c	3	12	18
210	c	4	12	26
211	c	5	12	20
212	c	6	12	40
213	c	7	12	49
214	c	8	12	21
215	c	9	12	27
216	c	10	12	9
217	c	11	12	0
218	c	12	12	0
219	c	13	12	16
220	c	15	12	0
221	c	16	12	21
222	c	17	12	3
223	c	18	12	15
224	c	19	12	0
225	c	20	12	0
226	s	1	13	0
227	s	2	13	18
228	s	3	13	13
229	s	5	13	29
230	s	6	13	20
231	s	7	13	0
232	s	10	13	20
233	s	12	13	18
234	s	13	13	32
235	s	14	13	31
236	s	15	13	22
237	s	16	13	17
238	s	17	13	24
239	s	18	13	18
240	s	19	13	29
241	s	20	13	24
242	s	1	14	36
243	s	3	14	10
244	s	4	14	45
245	s	5	14	0
246	s	8	14	36

Obs	ANI	TEN	RP	Y
**247**	s	9	14	14
**248**	s	10	14	0
**249**	s	11	14	39
**250**	s	12	14	35
**251**	s	13	14	8
**252**	s	14	14	15
**253**	s	15	14	4
**254**	s	16	14	13
**255**	s	17	14	7
**256**	s	18	14	6
**257**	s	19	14	8
**258**	s	20	14	0
**259**	s	1	15	13
**260**	s	2	15	0
**261**	s	3	15	6
**262**	s	4	15	9
**263**	s	6	15	14
**264**	s	7	15	1
**265**	s	8	15	21
**266**	s	9	15	11
**267**	s	10	15	1
**268**	s	11	15	10
**269**	s	12	15	0
**270**	s	13	15	0
**271**	s	14	15	22
**272**	s	16	15	14
**273**	s	17	15	13
**274**	s	18	15	9
**275**	s	19	15	0
**276**	s	20	15	3
**277**	s	1	16	26
**278**	s	2	16	14
**279**	s	3	16	14
**280**	s	4	16	4
**281**	s	5	16	3
**282**	s	6	16	16
**283**	s	7	16	17
**284**	s	8	16	16
**285**	s	9	16	25
**286**	s	10	16	16
**287**	s	12	16	0

Obs	ANI	TEN	RP	Y
**288**	s	14	16	0
**289**	s	15	16	7
**290**	s	16	16	3
**291**	s	17	16	6
**292**	s	18	16	27
**293**	s	19	16	6
**294**	s	1	17	0
**295**	s	2	17	0
**296**	s	3	17	4
**297**	s	4	17	22
**298**	s	5	17	24
**299**	s	6	17	0
**300**	s	7	17	4
**301**	s	9	17	3
**302**	s	10	17	5
**303**	s	11	17	39
**304**	s	12	17	4
**305**	s	13	17	34
**306**	s	14	17	59
**307**	s	15	17	0
**308**	s	16	17	14
**309**	s	17	17	26
**310**	s	18	17	12
**311**	s	19	17	47
**312**	s	20	17	18
**313**	s	1	18	4
**314**	s	2	18	0
**315**	s	3	18	0
**316**	s	4	18	0
**317**	s	5	18	17
**318**	s	6	18	26
**319**	s	7	18	0
**320**	s	8	18	0
**321**	s	9	18	17
**322**	s	10	18	31
**323**	s	11	18	0
**324**	s	12	18	0
**325**	s	13	18	9
**326**	s	14	18	0
**327**	s	15	18	4
**328**	s	16	18	0

Obs	ANI	TEN	RP	Y
**329**	s	17	18	0
**330**	s	18	18	15
**331**	s	19	18	0
**332**	s	1	19	21
**333**	s	2	19	10
**334**	s	4	19	25
**335**	s	5	19	20
**336**	s	6	19	0
**337**	s	7	19	11
**338**	s	8	19	10
**339**	s	9	19	6
**340**	s	11	19	15
**341**	s	12	19	7
**342**	s	13	19	16
**343**	s	15	19	0
**344**	s	16	19	10
**345**	s	17	19	15
**346**	s	19	19	1
**347**	s	20	19	0
**348**	s	1	20	0
**349**	s	2	20	9
**350**	s	3	20	9
**351**	s	4	20	8
**352**	s	5	20	20
**353**	s	7	20	22
**354**	s	8	20	4
**355**	s	9	20	10
**356**	s	10	20	13
**357**	s	12	20	0
**358**	s	13	20	9
**359**	s	14	20	3
**360**	s	15	20	15
**361**	s	16	20	13
**362**	s	17	20	8
**363**	s	19	20	7
**364**	s	20	20	25
**365**	s	1	21	15
**366**	s	3	21	13
**367**	s	4	21	4
**368**	s	5	21	0
**369**	s	7	21	14

Obs	ANI	TEN	RP	Y
**370**	s	8	21	4
**371**	s	9	21	20
**372**	s	10	21	4
**373**	s	11	21	8
**374**	s	12	21	21
**375**	s	13	21	12
**376**	s	14	21	22
**377**	s	15	21	6
**378**	s	16	21	0
**379**	s	17	21	4
**380**	s	19	21	16
**381**	s	20	21	8
**382**	s	1	22	9
**383**	s	2	22	10
**384**	s	3	22	6
**385**	s	4	22	6
**386**	s	5	22	2
**387**	s	6	22	11
**388**	s	7	22	2
**389**	s	8	22	5
**390**	s	9	22	0
**391**	s	10	22	0
**392**	s	11	22	9
**393**	s	12	22	4
**394**	s	13	22	15
**395**	s	14	22	0
**396**	s	15	22	10
**397**	s	17	22	20
**398**	s	18	22	9
**399**	s	19	22	11
**400**	s	20	22	0
**401**	s	1	23	0
**402**	s	2	23	9
**403**	s	4	23	39
**404**	s	5	23	4
**405**	s	6	23	9
**406**	s	7	23	18
**407**	s	8	23	2
**408**	s	9	23	16
**409**	s	10	23	8
**410**	s	11	23	21

Obs	ANI	TEN	RP	Y
**411**	s	12	23	19
**412**	s	13	23	16
**413**	s	14	23	7
**414**	s	15	23	12
**415**	s	16	23	12
**416**	s	17	23	4
**417**	s	18	23	26
**418**	s	20	23	11
**419**	s	2	24	11
**420**	s	4	24	16
**421**	s	5	24	5
**422**	s	6	24	4
**423**	s	7	24	3
**424**	s	8	24	18
**425**	s	9	24	1
**426**	s	10	24	0
**427**	s	11	24	10
**428**	s	12	24	14
**429**	s	13	24	18
**430**	s	14	24	15
**431**	s	15	24	21
**432**	s	16	24	30
**433**	s	17	24	18
**434**	s	18	24	12
**435**	s	19	24	7
**436**	s	20	24	30

Obs	ANI	TEN	RP	Y
**1**	c	1	1	17
**2**	c	2	1	31
**3**	c	3	1	46
**4**	c	4	1	46
**5**	c	5	1	35
**6**	c	6	1	10
**7**	c	7	1	43
**8**	c	8	1	26
**9**	c	9	1	32
**10**	c	10	1	149
**11**	c	11	1	28
**12**	c	12	1	28
**13**	c	13	1	16
**14**	c	14	1	40
**15**	c	15	1	24
**16**	c	16	1	14
**17**	c	17	1	12
**18**	c	18	1	32
**19**	c	19	1	0
**20**	c	20	1	42
**21**	c	1	2	24
**22**	c	2	2	44
**23**	c	3	2	14
**24**	c	4	2	19
**25**	c	5	2	69
**26**	c	6	2	46
**27**	c	7	2	48
**28**	c	8	2	10
**29**	c	9	2	5
**30**	c	10	2	132
**31**	c	11	2	37
**32**	c	12	2	30
**33**	c	13	2	26
**34**	c	14	2	59
**35**	c	15	2	52
**36**	c	16	2	24
**37**	c	17	2	26
**38**	c	18	2	25
**39**	c	19	2	16
**40**	c	20	2	48
**41**	c	1	3	0

Obs	ANI	TEN	RP	Y
**42**	c	2	3	16
**43**	c	3	3	0
**44**	c	4	3	12
**45**	c	5	3	38
**46**	c	6	3	0
**47**	c	7	3	4
**48**	c	8	3	0
**49**	c	9	3	0
**50**	c	10	3	36
**51**	c	11	3	10
**52**	c	12	3	2
**53**	c	13	3	4
**54**	c	14	3	6
**55**	c	15	3	32
**56**	c	16	3	8
**57**	c	17	3	14
**58**	c	18	3	18
**59**	c	19	3	8
**60**	c	20	3	18
**61**	c	1	4	14
**62**	c	2	4	6
**63**	c	3	4	26
**64**	c	4	4	0
**65**	c	5	4	2
**66**	c	6	4	0
**67**	c	7	4	16
**68**	c	8	4	15
**69**	c	9	4	0
**70**	c	10	4	12
**71**	c	11	4	38
**72**	c	12	4	12
**73**	c	13	4	0
**74**	c	14	4	56
**75**	c	15	4	4
**76**	c	16	4	19
**77**	c	17	4	12
**78**	c	18	4	14
**79**	c	19	4	10
**80**	c	20	4	30
**81**	c	1	5	11
**82**	c	2	5	28

Obs	ANI	TEN	RP	Y
83	c	3	5	28
84	c	4	5	30
85	c	5	5	20
86	c	6	5	8
87	c	7	5	15
88	c	8	5	12
89	c	9	5	18
90	c	10	5	22
91	c	11	5	31
92	c	12	5	5
93	c	13	5	7
94	c	14	5	12
95	c	15	5	10
96	c	16	5	3
97	c	17	5	6
98	c	18	5	3
99	c	19	5	4
100	c	20	5	19
101	c	1	6	7
102	c	2	6	2
103	c	3	6	2
104	c	4	6	8
105	c	5	6	0
106	c	6	6	4
107	c	7	6	6
108	c	8	6	21
109	c	9	6	11
110	c	10	6	0
111	c	11	6	24
112	c	12	6	15
113	c	13	6	10
114	c	14	6	3
115	c	15	6	3
116	c	16	6	0
117	c	17	6	0
118	c	18	6	1
119	c	19	6	15
120	c	20	6	13
121	c	1	7	5
122	c	2	7	0
123	c	3	7	0

Obs	ANI	TEN	RP	Y
**124**	c	4	7	0
**125**	c	5	7	0
**126**	c	6	7	11
**127**	c	7	7	15
**128**	c	8	7	5
**129**	c	9	7	0
**130**	c	10	7	3
**131**	c	11	7	0
**132**	c	12	7	0
**133**	c	13	7	0
**134**	c	14	7	20
**135**	c	15	7	0
**136**	c	16	7	11
**137**	c	17	7	15
**138**	c	18	7	5
**139**	c	19	7	0
**140**	c	20	7	3
**141**	c	1	8	0
**142**	c	2	8	0
**143**	c	3	8	1
**144**	c	4	8	8
**145**	c	5	8	18
**146**	c	6	8	6
**147**	c	7	8	0
**148**	c	8	8	0
**149**	c	9	8	0
**150**	c	10	8	0
**151**	c	11	8	0
**152**	c	12	8	0
**153**	c	13	8	0
**154**	c	14	8	0
**155**	c	15	8	15
**156**	c	16	8	14
**157**	c	17	8	0
**158**	c	18	8	0
**159**	c	19	8	36
**160**	c	20	8	24
**161**	c	1	9	32
**162**	c	2	9	4
**163**	c	3	9	35
**164**	c	4	9	54

Obs	ANI	TEN	RP	Y
**165**	c	5	9	6
**166**	c	6	9	11
**167**	c	7	9	37
**168**	c	8	9	12
**169**	c	9	9	24
**170**	c	10	9	2
**171**	c	11	9	0
**172**	c	12	9	0
**173**	c	13	9	12
**174**	c	14	9	0
**175**	c	15	9	0
**176**	c	16	9	0
**177**	c	17	9	0
**178**	c	18	9	0
**179**	c	19	9	0
**180**	c	20	9	0
**181**	c	4	10	0
**182**	c	5	10	11
**183**	c	6	10	0
**184**	c	7	10	0
**185**	c	8	10	7
**186**	c	9	10	0
**187**	c	10	10	0
**188**	c	1	11	0
**189**	c	2	11	0
**190**	c	3	11	17
**191**	c	4	11	0
**192**	c	6	11	11
**193**	c	7	11	8
**194**	c	8	11	0
**195**	c	9	11	0
**196**	c	10	11	3
**197**	c	11	11	21
**198**	c	12	11	47
**199**	c	13	11	26
**200**	c	14	11	13
**201**	c	15	11	29
**202**	c	16	11	20
**203**	c	17	11	21
**204**	c	18	11	24
**205**	c	19	11	28

Obs	ANI	TEN	RP	Y
206	c	20	11	7
207	c	1	12	13
208	c	2	12	16
209	c	3	12	18
210	c	4	12	26
211	c	5	12	20
212	c	6	12	40
213	c	7	12	49
214	c	8	12	21
215	c	9	12	27
216	c	10	12	9
217	c	11	12	0
218	c	12	12	0
219	c	13	12	16
220	c	15	12	0
221	c	16	12	21
222	c	17	12	3
223	c	18	12	15
224	c	19	12	0
225	c	20	12	0
226	s	1	13	0
227	s	2	13	18
228	s	3	13	13
229	s	5	13	29
230	s	6	13	20
231	s	7	13	0
232	s	10	13	20
233	s	12	13	18
234	s	13	13	32
235	s	14	13	31
236	s	15	13	22
237	s	16	13	17
238	s	17	13	24
239	s	18	13	18
240	s	19	13	29
241	s	20	13	24
242	s	1	14	36
243	s	3	14	10
244	s	4	14	45
245	s	5	14	0
246	s	8	14	36

Obs	ANI	TEN	RP	Y
247	s	9	14	14
248	s	10	14	0
249	s	11	14	39
250	s	12	14	35
251	s	13	14	8
252	s	14	14	15
253	s	15	14	4
254	s	16	14	13
255	s	17	14	7
256	s	18	14	6
257	s	19	14	8
258	s	20	14	0
259	s	1	15	13
260	s	2	15	0
261	s	3	15	6
262	s	4	15	9
263	s	6	15	14
264	s	7	15	1
265	s	8	15	21
266	s	9	15	11
267	s	10	15	1
268	s	11	15	10
269	s	12	15	0
270	s	13	15	0
271	s	14	15	22
272	s	16	15	14
273	s	17	15	13
274	s	18	15	9
275	s	19	15	0
276	s	20	15	3
277	s	1	16	26
278	s	2	16	14
279	s	3	16	14
280	s	4	16	4
281	s	5	16	3
282	s	6	16	16
283	s	7	16	17
284	s	8	16	16
285	s	9	16	25
286	s	10	16	16
287	s	12	16	0

Obs	ANI	TEN	RP	Y
**288**	s	14	16	0
**289**	s	15	16	7
**290**	s	16	16	3
**291**	s	17	16	6
**292**	s	18	16	27
**293**	s	19	16	6
**294**	s	1	17	0
**295**	s	2	17	0
**296**	s	3	17	4
**297**	s	4	17	22
**298**	s	5	17	24
**299**	s	6	17	0
**300**	s	7	17	4
**301**	s	9	17	3
**302**	s	10	17	5
**303**	s	11	17	39
**304**	s	12	17	4
**305**	s	13	17	34
**306**	s	14	17	59
**307**	s	15	17	0
**308**	s	16	17	14
**309**	s	17	17	26
**310**	s	18	17	12
**311**	s	19	17	47
**312**	s	20	17	18
**313**	s	1	18	4
**314**	s	2	18	0
**315**	s	3	18	0
**316**	s	4	18	0
**317**	s	5	18	17
**318**	s	6	18	26
**319**	s	7	18	0
**320**	s	8	18	0
**321**	s	9	18	17
**322**	s	10	18	31
**323**	s	11	18	0
**324**	s	12	18	0
**325**	s	13	18	9
**326**	s	14	18	0
**327**	s	15	18	4
**328**	s	16	18	0

Obs	ANI	TEN	RP	Y
**329**	s	17	18	0
**330**	s	18	18	15
**331**	s	19	18	0
**332**	s	1	19	21
**333**	s	2	19	10
**334**	s	4	19	25
**335**	s	5	19	20
**336**	s	6	19	0
**337**	s	7	19	11
**338**	s	8	19	10
**339**	s	9	19	6
**340**	s	11	19	15
**341**	s	12	19	7
**342**	s	13	19	16
**343**	s	15	19	0
**344**	s	16	19	10
**345**	s	17	19	15
**346**	s	19	19	1
**347**	s	20	19	0
**348**	s	1	20	0
**349**	s	2	20	9
**350**	s	3	20	9
**351**	s	4	20	8
**352**	s	5	20	20
**353**	s	7	20	22
**354**	s	8	20	4
**355**	s	9	20	10
**356**	s	10	20	13
**357**	s	12	20	0
**358**	s	13	20	9
**359**	s	14	20	3
**360**	s	15	20	15
**361**	s	16	20	13
**362**	s	17	20	8
**363**	s	19	20	7
**364**	s	20	20	25
**365**	s	1	21	15
**366**	s	3	21	13
**367**	s	4	21	4
**368**	s	5	21	0
**369**	s	7	21	14

Obs	ANI	TEN	RP	Y
**370**	s	8	21	4
**371**	s	9	21	20
**372**	s	10	21	4
**373**	s	11	21	8
**374**	s	12	21	21
**375**	s	13	21	12
**376**	s	14	21	22
**377**	s	15	21	6
**378**	s	16	21	0
**379**	s	17	21	4
**380**	s	19	21	16
**381**	s	20	21	8
**382**	s	1	22	9
**383**	s	2	22	10
**384**	s	3	22	6
**385**	s	4	22	6
**386**	s	5	22	2
**387**	s	6	22	11
**388**	s	7	22	2
**389**	s	8	22	5
**390**	s	9	22	0
**391**	s	10	22	0
**392**	s	11	22	9
**393**	s	12	22	4
**394**	s	13	22	15
**395**	s	14	22	0
**396**	s	15	22	10
**397**	s	17	22	20
**398**	s	18	22	9
**399**	s	19	22	11
**400**	s	20	22	0
**401**	s	1	23	0
**402**	s	2	23	9
**403**	s	4	23	39
**404**	s	5	23	4
**405**	s	6	23	9
**406**	s	7	23	18
**407**	s	8	23	2
**408**	s	9	23	16
**409**	s	10	23	8
**410**	s	11	23	21

Obs	ANI	TEN	RP	Y
**411**	s	12	23	19
**412**	s	13	23	16
**413**	s	14	23	7
**414**	s	15	23	12
**415**	s	16	23	12
**416**	s	17	23	4
**417**	s	18	23	26
**418**	s	20	23	11
**419**	s	2	24	11
**420**	s	4	24	16
**421**	s	5	24	5
**422**	s	6	24	4
**423**	s	7	24	3
**424**	s	8	24	18
**425**	s	9	24	1
**426**	s	10	24	0
**427**	s	11	24	10
**428**	s	12	24	14
**429**	s	13	24	18
**430**	s	14	24	15
**431**	s	15	24	21
**432**	s	16	24	30
**433**	s	17	24	18
**434**	s	18	24	12
**435**	s	19	24	7
**436**	s	20	24	30

The Mixed Procedure

Model Information	
Data Set	WORK.AU
Dependent Variable	Y
Covariance Structure	Ante-dependence
Subject Effect	RP(TEN)
Estimation Method	REML
Residual Variance Method	None
Fixed Effects SE Method	Model-Based
Degrees of Freedom Method	Between-Within

Class Level Information

Class	Levels	Values
RP	24	1 2 3 4 5 6 7 8 9 10 11 12 13 14 15 16 17 18 19 20 21 22 23 24
TEN	20	1 10 11 12 13 14 15 16 17 18 19 2 20 3 4 5 6 7 8 9
ANI	2	c s

Dimensions	
Covariance Parameters	3
Columns in X	63
Columns in Z	0
Subjects	436
Max Obs per Subject	1

Number of Observations	
Number of Observations Read	436
Number of Observations Used	436
Number of Observations Not Used	0

Iteration History

Iteration	Evaluations	-2 Res Log Like	Criterion
0	1	3393.82399168	
1	2	3352.26600629	0.00005523
2	1	3338.68149981	0.00000545
3	1	3335.96327818	0.00000023
4	1	3335.79344292	0.00000000

Convergence criteria met but final Hessian is not positive definite.

The Mixed Procedure

Estimated R Matrix for RP(TEN) 1 1	
Row	Col1
1	358.05

Estimated R Correlation Matrix for RP(TEN) 1 1	
Row	Col1
1	1.0000

Covariance Parameter Estimates		
Cov Parm	Subject	Estimate
Var(1)	RP(TEN)	358.05
Var(2)	RP(TEN)	117.51
Rho(1)	RP(TEN)	0

Fit Statistics	
-2 Res Log Likelihood	3335.8
AIC (Smaller is Better)	3341.8
AICC (Smaller is Better)	3341.9
BIC (Smaller is Better)	3354.0

Null Model Likelihood Ratio Test		
DF	Chi-Square	Pr > ChiSq
2	58.03	<.0001

Type 3 Tests of Fixed Effects				
Effect	Num DF	Den DF	F Value	Pr > F
TEN	19	396	0.71	0.8101
ANI	1	396	5.10	0.0244
TEN*ANI	19	396	0.91	0.5683

The Mixed Procedure

Model Information	
**Data Set**	WORK.AU
**Dependent Variable**	Y
**Covariance Structure**	Autoregressive
**Subject Effect**	RP(TEN)
**Estimation Method**	REML
**Residual Variance Method**	Profile
**Fixed Effects SE Method**	Model-Based
**Degrees of Freedom Method**	Between-Within

Class Level Information		
**Class**	**Levels**	**Values**
**RP**	24	1 2 3 4 5 6 7 8 9 10 11 12 13 14 15 16 17 18 19 20 21 22 23 24
**TEN**	20	1 10 11 12 13 14 15 16 17 18 19 2 20 3 4 5 6 7 8 9
**ANI**	2	c s

Dimensions	
**Covariance Parameters**	2
**Columns in X**	63
**Columns in Z**	0
**Subjects**	436
**Max Obs per Subject**	1

Number of Observations	
**Number of Observations Read**	436
**Number of Observations Used**	436
**Number of Observations Not Used**	0

Iteration History			
**Iteration**	**Evaluations**	**-2 Res Log Like**	**Criterion**
**0**	1	3393.82399168	
**1**	1	3393.82399168	0.00000000

Convergence criteria met but final Hessian is not positive definite.

Estimated R Matrix for RP(TEN) 1 1	
**Row**	**Col1**
**1**	242.60

The Mixed Procedure

Estimated R Correlation Matrix for RP(TEN) 1 1	
**Row**	**Col1**
**1**	1.0000

Covariance Parameter Estimates		
**Cov Parm**	**Subject**	**Estimate**
**AR(1)**	RP(TEN)	0
**Residual**		242.60

Fit Statistics	
**-2 Res Log Likelihood**	3393.8
**AIC (Smaller is Better)**	3397.8
**AICC (Smaller is Better)**	3397.9
**BIC (Smaller is Better)**	3406.0

Null Model Likelihood Ratio Test		
**DF**	**Chi-Square**	**Pr > ChiSq**
1	0.00	1.0000

Type 3 Tests of Fixed Effects				
**Effect**	**Num DF**	**Den DF**	**F Value**	**Pr > F**
**TEN**	19	396	0.69	0.8337
**ANI**	1	396	4.91	0.0272
**TEN*ANI**	19	396	0.88	0.6129

The Mixed Procedure

Model Information	
Data Set	WORK.AU
Dependent Variable	Y
Covariance Structure	Heterogeneous Autoregressive
Subject Effect	RP(TEN)
Estimation Method	REML
Residual Variance Method	None
Fixed Effects SE Method	Model-Based
Degrees of Freedom Method	Between-Within

Class Level Information		
Class	Levels	Values
RP	24	1 2 3 4 5 6 7 8 9 10 11 12 13 14 15 16 17 18 19 20 21 22 23 24
TEN	20	1 10 11 12 13 14 15 16 17 18 19 2 20 3 4 5 6 7 8 9
ANI	2	c s

Dimensions	
Covariance Parameters	3
Columns in X	63
Columns in Z	0
Subjects	436
Max Obs per Subject	1

Number of Observations	
Number of Observations Read	436
Number of Observations Used	436
Number of Observations Not Used	0

Iteration History			
Iteration	Evaluations	-2 Res Log Like	Criterion
0	1	3393.82399168	
1	2	3352.26600629	0.00005523
2	1	3338.68149981	0.00000545
3	1	3335.96327818	0.00000023
4	1	3335.79344292	0.00000000

Convergence criteria met but final Hessian is not positive definite.

The Mixed Procedure

Estimated R Matrix for RP(TEN) 1 1	
**Row**	**Col1**
**1**	358.05

Estimated R Correlation Matrix for RP(TEN) 1 1	
**Row**	**Col1**
**1**	1.0000

Covariance Parameter Estimates		
**Cov Parm**	**Subject**	**Estimate**
**Var(1)**	RP(TEN)	358.05
**Var(2)**	RP(TEN)	117.51
**ARH(1)**	RP(TEN)	0

Fit Statistics	
**-2 Res Log Likelihood**	3335.8
**AIC (Smaller is Better)**	3341.8
**AICC (Smaller is Better)**	3341.9
**BIC (Smaller is Better)**	3354.0

Null Model Likelihood Ratio Test		
**DF**	**Chi-Square**	**Pr > ChiSq**
2	58.03	<.0001

Type 3 Tests of Fixed Effects				
**Effect**	**Num DF**	**Den DF**	**F Value**	**Pr > F**
**TEN**	19	396	0.71	0.8101
**ANI**	1	396	5.10	0.0244
**TEN*ANI**	19	396	0.91	0.5683

The Mixed Procedure

Model Information	
**Data Set**	WORK.AU
**Dependent Variable**	Y
**Covariance Structure**	Autoregressive Moving Average
**Subject Effect**	RP(TEN)
**Estimation Method**	REML
**Residual Variance Method**	Profile
**Fixed Effects SE Method**	Model-Based
**Degrees of Freedom Method**	Between-Within

Class Level Information		
**Class**	**Levels**	**Values**
**RP**	24	1 2 3 4 5 6 7 8 9 10 11 12 13 14 15 16 17 18 19 20 21 22 23 24
**TEN**	20	1 10 11 12 13 14 15 16 17 18 19 2 20 3 4 5 6 7 8 9
**ANI**	2	c s

Dimensions	
**Covariance Parameters**	3
**Columns in X**	63
**Columns in Z**	0
**Subjects**	436
**Max Obs per Subject**	1

Number of Observations	
**Number of Observations Read**	436
**Number of Observations Used**	436
**Number of Observations Not Used**	0

Iteration History			
**Iteration**	**Evaluations**	**-2 Res Log Like**	**Criterion**
**0**	1	3393.82399168	
**1**	1	3393.82399168	0.00000000

Convergence criteria met but final Hessian is not positive definite.

Estimated R Matrix for RP(TEN) 1 1	
**Row**	**Col1**
**1**	242.60

The Mixed Procedure

Estimated R Correlation Matrix for RP(TEN) 1 1	
**Row**	**Col1**
**1**	1.0000

Covariance Parameter Estimates		
**Cov Parm**	**Subject**	**Estimate**
**Rho**	RP(TEN)	0
**Gamma**	RP(TEN)	0
**Residual**		242.60

Fit Statistics	
**-2 Res Log Likelihood**	3393.8
**AIC (Smaller is Better)**	3399.8
**AICC (Smaller is Better)**	3399.9
**BIC (Smaller is Better)**	3412.1

Null Model Likelihood Ratio Test		
**DF**	**Chi-Square**	**Pr > ChiSq**
2	0.00	1.0000

Type 3 Tests of Fixed Effects				
**Effect**	**Num DF**	**Den DF**	**F Value**	**Pr > F**
**TEN**	19	396	0.69	0.8337
**ANI**	1	396	4.91	0.0272
**TEN*ANI**	19	396	0.88	0.6129

The Mixed Procedure

Model Information	
Data Set	WORK.AU
Dependent Variable	Y
Covariance Structure	Compound Symmetry
Subject Effect	RP(TEN)
Estimation Method	REML
Residual Variance Method	Profile
Fixed Effects SE Method	Model-Based
Degrees of Freedom Method	Between-Within

Class Level Information		
Class	Levels	Values
RP	24	1 2 3 4 5 6 7 8 9 10 11 12 13 14 15 16 17 18 19 20 21 22 23 24
TEN	20	1 10 11 12 13 14 15 16 17 18 19 2 20 3 4 5 6 7 8 9
ANI	2	c s

Dimensions	
Covariance Parameters	2
Columns in X	63
Columns in Z	0
Subjects	436
Max Obs per Subject	1

Number of Observations	
Number of Observations Read	436
Number of Observations Used	436
Number of Observations Not Used	0

Iteration History			
Iteration	Evaluations	-2 Res Log Like	Criterion
0	1	3393.82399168	
1	1	3393.82399168	0.00000000

Convergence criteria met but final Hessian is not positive definite.

Estimated R Matrix for RP(TEN) 1 1	
Row	Col1
1	242.60

The Mixed Procedure

Estimated R Correlation Matrix for RP(TEN) 1 1	
**Row**	**Col1**
**1**	1.0000

Covariance Parameter Estimates		
**Cov Parm**	**Subject**	**Estimate**
**CS**	RP(TEN)	241.60
**Residual**		0.9959

Fit Statistics	
**-2 Res Log Likelihood**	3393.8
**AIC (Smaller is Better)**	3397.8
**AICC (Smaller is Better)**	3397.9
**BIC (Smaller is Better)**	3406.0

Null Model Likelihood Ratio Test		
**DF**	**Chi-Square**	**Pr > ChiSq**
1	0.00	1.0000

Type 3 Tests of Fixed Effects				
**Effect**	**Num DF**	**Den DF**	**F Value**	**Pr > F**
**TEN**	19	396	0.69	0.8337
**ANI**	1	396	4.91	0.0272
**TEN*ANI**	19	396	0.88	0.6129

The Mixed Procedure

Model Information	
Data Set	WORK.AU
Dependent Variable	Y
Covariance Structure	Heterogeneous Compound Symmetry
Subject Effect	RP(TEN)
Estimation Method	REML
Residual Variance Method	None
Fixed Effects SE Method	Model-Based
Degrees of Freedom Method	Between-Within

Class Level Information		
Class	Levels	Values
RP	24	1 2 3 4 5 6 7 8 9 10 11 12 13 14 15 16 17 18 19 20 21 22 23 24
TEN	20	1 10 11 12 13 14 15 16 17 18 19 2 20 3 4 5 6 7 8 9
ANI	2	c s

Dimensions	
Covariance Parameters	3
Columns in X	63
Columns in Z	0
Subjects	436
Max Obs per Subject	1

Number of Observations	
Number of Observations Read	436
Number of Observations Used	436
Number of Observations Not Used	0

Iteration History			
Iteration	Evaluations	-2 Res Log Like	Criterion
0	1	3393.82399168	
1	2	3352.26600629	0.00005523
2	1	3338.68149981	0.00000545
3	1	3335.96327818	0.00000023
4	1	3335.79344292	0.00000000

Convergence criteria met but final Hessian is not positive definite.

The Mixed Procedure

Estimated R Matrix for RP(TEN) 1 1	
**Row**	**Col1**
**1**	358.05

Estimated R Correlation Matrix for RP(TEN) 1 1	
**Row**	**Col1**
**1**	1.0000

Covariance Parameter Estimates		
**Cov Parm**	**Subject**	**Estimate**
**Var(1)**	RP(TEN)	358.05
**Var(2)**	RP(TEN)	117.51
**CSH**	RP(TEN)	0

Fit Statistics	
**-2 Res Log Likelihood**	3335.8
**AIC (Smaller is Better)**	3341.8
**AICC (Smaller is Better)**	3341.9
**BIC (Smaller is Better)**	3354.0

Null Model Likelihood Ratio Test		
**DF**	**Chi-Square**	**Pr > ChiSq**
2	58.03	<.0001

Type 3 Tests of Fixed Effects				
**Effect**	**Num DF**	**Den DF**	**F Value**	**Pr > F**
**TEN**	19	396	0.71	0.8101
**ANI**	1	396	5.10	0.0244
**TEN*ANI**	19	396	0.91	0.5683

The Mixed Procedure

Model Information	
**Data Set**	WORK.AU
**Dependent Variable**	Y
**Covariance Structure**	Factor Analytic
**Subject Effect**	RP(TEN)
**Estimation Method**	REML
**Residual Variance Method**	None
**Fixed Effects SE Method**	Model-Based
**Degrees of Freedom Method**	Between-Within

Class Level Information		
**Class**	**Levels**	**Values**
**RP**	24	1 2 3 4 5 6 7 8 9 10 11 12 13 14 15 16 17 18 19 20 21 22 23 24
**TEN**	20	1 10 11 12 13 14 15 16 17 18 19 2 20 3 4 5 6 7 8 9
**ANI**	2	c s

Dimensions	
**Covariance Parameters**	2
**Columns in X**	63
**Columns in Z**	0
**Subjects**	436
**Max Obs per Subject**	1

Number of Observations	
**Number of Observations Read**	436
**Number of Observations Used**	436
**Number of Observations Not Used**	0

The Mixed Procedure

Model Information	
**Data Set**	WORK.AU
**Dependent Variable**	Y
**Covariance Structure**	Factor Analytic
**Subject Effect**	RP(TEN)
**Estimation Method**	REML
**Residual Variance Method**	None
**Fixed Effects SE Method**	Model-Based
**Degrees of Freedom Method**	Between-Within

Class Level Information		
**Class**	**Levels**	**Values**
**RP**	24	1 2 3 4 5 6 7 8 9 10 11 12 13 14 15 16 17 18 19 20 21 22 23 24
**TEN**	20	1 10 11 12 13 14 15 16 17 18 19 2 20 3 4 5 6 7 8 9
**ANI**	2	c s

Dimensions	
**Covariance Parameters**	4
**Columns in X**	63
**Columns in Z**	0
**Subjects**	436
**Max Obs per Subject**	1

Number of Observations	
**Number of Observations Read**	436
**Number of Observations Used**	436
**Number of Observations Not Used**	0

Iteration History			
**Iteration**	**Evaluations**	**-2 Res Log Like**	**Criterion**
**0**	1	3393.82399168	
**1**	2	3341.33603370	0.03330299
**2**	2	3335.97490799	0.00093090
**3**	2	3335.79280457	0.00000154
**4**	1	3335.79248483	0.00000000

Convergence criteria met but final Hessian is not positive definite.

The Mixed Procedure

Estimated R Matrix for RP(TEN) 1 1	
**Row**	**Col1**
**1**	359.15

Estimated R Correlation Matrix for RP(TEN) 1 1	
**Row**	**Col1**
**1**	1.0000

Covariance Parameter Estimates		
**Cov Parm**	**Subject**	**Estimate**
**FA(1)**	RP(TEN)	242.53
**FA(2)**	RP(TEN)	4.7151
**FA(1,1)**	RP(TEN)	10.7990
**FA(2,1)**	RP(TEN)	10.6203

Fit Statistics	
**-2 Res Log Likelihood**	3335.8
**AIC (Smaller is Better)**	3343.8
**AICC (Smaller is Better)**	3343.9
**BIC (Smaller is Better)**	3360.1

Null Model Likelihood Ratio Test		
**DF**	**Chi-Square**	**Pr > ChiSq**
3	58.03	<.0001

Type 3 Tests of Fixed Effects				
**Effect**	**Num DF**	**Den DF**	**F Value**	**Pr > F**
**TEN**	19	396	0.71	0.8117
**ANI**	1	396	5.09	0.0246
**TEN*ANI**	19	396	0.91	0.5709

The Mixed Procedure

Model Information	
**Data Set**	WORK.AU
**Dependent Variable**	Y
**Covariance Structure**	Huynh-Feldt
**Subject Effect**	RP(TEN)
**Estimation Method**	REML
**Residual Variance Method**	None
**Fixed Effects SE Method**	Model-Based
**Degrees of Freedom Method**	Between-Within

Class Level Information		
**Class**	**Levels**	**Values**
**RP**	24	1 2 3 4 5 6 7 8 9 10 11 12 13 14 15 16 17 18 19 20 21 22 23 24
**TEN**	20	1 10 11 12 13 14 15 16 17 18 19 2 20 3 4 5 6 7 8 9
**ANI**	2	c s

Dimensions	
**Covariance Parameters**	3
**Columns in X**	63
**Columns in Z**	0
**Subjects**	436
**Max Obs per Subject**	1

Number of Observations	
**Number of Observations Read**	436
**Number of Observations Used**	436
**Number of Observations Not Used**	0

Iteration History			
**Iteration**	**Evaluations**	**-2 Res Log Like**	**Criterion**
**0**	1	3393.82399168	
**1**	2	3343.92669713	0.00000500
**2**	1	3336.19156618	0.00000041
**3**	1	3335.79500723	0.00000000

Convergence criteria met but final Hessian is not positive definite.

The Mixed Procedure

Estimated R Matrix for RP(TEN) 1 1	
**Row**	**Col1**
**1**	357.37

Estimated R Correlation Matrix for RP(TEN) 1 1	
**Row**	**Col1**
**1**	1.0000

Covariance Parameter Estimates		
**Cov Parm**	**Subject**	**Estimate**
**Var(1)**	RP(TEN)	357.37
**Var(2)**	RP(TEN)	117.51
**HF**	RP(TEN)	361.82

Fit Statistics	
**-2 Res Log Likelihood**	3335.8
**AIC (Smaller is Better)**	3341.8
**AICC (Smaller is Better)**	3341.9
**BIC (Smaller is Better)**	3354.0

Null Model Likelihood Ratio Test		
**DF**	**Chi-Square**	**Pr > ChiSq**
2	58.03	<.0001

Type 3 Tests of Fixed Effects				
**Effect**	**Num DF**	**Den DF**	**F Value**	**Pr > F**
**TEN**	19	396	0.71	0.8090
**ANI**	1	396	5.11	0.0243
**TEN*ANI**	19	396	0.91	0.5667

The Mixed Procedure

Model Information	
**Data Set**	WORK.AU
**Dependent Variable**	Y
**Covariance Structure**	Variance Components
**Subject Effect**	RP(TEN)
**Estimation Method**	REML
**Residual Variance Method**	Parameter
**Fixed Effects SE Method**	Model-Based
**Degrees of Freedom Method**	Between-Within

Class Level Information		
**Class**	**Levels**	**Values**
**RP**	24	1 2 3 4 5 6 7 8 9 10 11 12 13 14 15 16 17 18 19 20 21 22 23 24
**TEN**	20	1 10 11 12 13 14 15 16 17 18 19 2 20 3 4 5 6 7 8 9
**ANI**	2	c s

Dimensions	
**Covariance Parameters**	1
**Columns in X**	63
**Columns in Z**	0
**Subjects**	436
**Max Obs per Subject**	1

Number of Observations	
**Number of Observations Read**	436
**Number of Observations Used**	436
**Number of Observations Not Used**	0

Iteration History			
**Iteration**	**Evaluations**	**-2 Res Log Like**	**Criterion**
**0**	1	3393.82399168	
**1**	1	3393.82399168	0.00000000

Convergence criteria met.

Estimated R Matrix for RP(TEN) 1 1	
**Row**	**Col1**
**1**	242.60

The Mixed Procedure

Estimated R Correlation Matrix for RP(TEN) 1 1	
**Row**	**Col1**
**1**	1.0000

Covariance Parameter Estimates		
**Cov Parm**	**Subject**	**Estimate**
**ANI**	RP(TEN)	242.60

Fit Statistics	
**-2 Res Log Likelihood**	3393.8
**AIC (Smaller is Better)**	3395.8
**AICC (Smaller is Better)**	3395.8
**BIC (Smaller is Better)**	3399.9

Null Model Likelihood Ratio Test		
**DF**	**Chi-Square**	**Pr > ChiSq**
0	0.00	1.0000

Type 3 Tests of Fixed Effects				
**Effect**	**Num DF**	**Den DF**	**F Value**	**Pr > F**
**TEN**	19	396	0.69	0.8337
**ANI**	1	396	4.91	0.0272
**TEN*ANI**	19	396	0.88	0.6129

The Mixed Procedure

Model Information	
**Data Set**	WORK.AU
**Dependent Variable**	Y
**Covariance Structure**	Toeplitz
**Subject Effect**	RP(TEN)
**Estimation Method**	REML
**Residual Variance Method**	Profile
**Fixed Effects SE Method**	Model-Based
**Degrees of Freedom Method**	Between-Within

Class Level Information		
**Class**	**Levels**	**Values**
**RP**	24	1 2 3 4 5 6 7 8 9 10 11 12 13 14 15 16 17 18 19 20 21 22 23 24
**TEN**	20	1 10 11 12 13 14 15 16 17 18 19 2 20 3 4 5 6 7 8 9
**ANI**	2	c s

Dimensions	
**Covariance Parameters**	2
**Columns in X**	63
**Columns in Z**	0
**Subjects**	436
**Max Obs per Subject**	1

Number of Observations	
**Number of Observations Read**	436
**Number of Observations Used**	436
**Number of Observations Not Used**	0

Iteration History			
**Iteration**	**Evaluations**	**-2 Res Log Like**	**Criterion**
**0**	1	3393.82399168	
**1**	1	3393.82399168	0.00000000

Convergence criteria met but final Hessian is not positive definite.

Estimated R Matrix for RP(TEN) 1 1	
**Row**	**Col1**
**1**	242.60

The Mixed Procedure

Estimated R Correlation Matrix for RP(TEN) 1 1	
Row	Col1
1	1.0000

Covariance Parameter Estimates		
Cov Parm	Subject	Estimate
TOEP(2)	RP(TEN)	0
Residual		242.60

Fit Statistics	
-2 Res Log Likelihood	3393.8
AIC (Smaller is Better)	3397.8
AICC (Smaller is Better)	3397.9
BIC (Smaller is Better)	3406.0

Null Model Likelihood Ratio Test		
DF	Chi-Square	Pr > ChiSq
1	0.00	1.0000

Type 3 Tests of Fixed Effects				
Effect	Num DF	Den DF	F Value	Pr > F
TEN	19	396	0.69	0.8337
ANI	1	396	4.91	0.0272
TEN*ANI	19	396	0.88	0.6129

The Mixed Procedure

Model Information	
**Data Set**	WORK.AU
**Dependent Variable**	Y
**Covariance Structure**	Heterogeneous Toeplitz
**Subject Effect**	RP(TEN)
**Estimation Method**	REML
**Residual Variance Method**	None
**Fixed Effects SE Method**	Model-Based
**Degrees of Freedom Method**	Between-Within

Class Level Information		
**Class**	**Levels**	**Values**
**RP**	24	1 2 3 4 5 6 7 8 9 10 11 12 13 14 15 16 17 18 19 20 21 22 23 24
**TEN**	20	1 10 11 12 13 14 15 16 17 18 19 2 20 3 4 5 6 7 8 9
**ANI**	2	c s

Dimensions	
**Covariance Parameters**	3
**Columns in X**	63
**Columns in Z**	0
**Subjects**	436
**Max Obs per Subject**	1

Number of Observations	
**Number of Observations Read**	436
**Number of Observations Used**	436
**Number of Observations Not Used**	0

Iteration History			
**Iteration**	**Evaluations**	**-2 Res Log Like**	**Criterion**
**0**	1	3393.82399168	
**1**	2	3352.26600629	0.00005523
**2**	1	3338.68149981	0.00000545
**3**	1	3335.96327818	0.00000023
**4**	1	3335.79344292	0.00000000

Convergence criteria met but final Hessian is not positive definite.

The Mixed Procedure

Estimated R Matrix for RP(TEN) 1 1	
**Row**	**Col1**
**1**	358.05

Estimated R Correlation Matrix for RP(TEN) 1 1	
**Row**	**Col1**
**1**	1.0000

Covariance Parameter Estimates		
**Cov Parm**	**Subject**	**Estimate**
**Var(1)**	RP(TEN)	358.05
**Var(2)**	RP(TEN)	117.51
**TOEPH(1)**	RP(TEN)	0

Fit Statistics	
**-2 Res Log Likelihood**	3335.8
**AIC (Smaller is Better)**	3341.8
**AICC (Smaller is Better)**	3341.9
**BIC (Smaller is Better)**	3354.0

Null Model Likelihood Ratio Test		
**DF**	**Chi-Square**	**Pr > ChiSq**
2	58.03	<.0001

Type 3 Tests of Fixed Effects				
**Effect**	**Num DF**	**Den DF**	**F Value**	**Pr > F**
**TEN**	19	396	0.71	0.8101
**ANI**	1	396	5.10	0.0244
**TEN*ANI**	19	396	0.91	0.5683

The Mixed Procedure

Model Information	
Data Set	WORK.AU
Dependent Variable	Y
Covariance Structure	Unstructured
Subject Effect	RP(TEN)
Estimation Method	REML
Residual Variance Method	None
Fixed Effects SE Method	Model-Based
Degrees of Freedom Method	Between-Within

Class Level Information		
Class	Levels	Values
RP	24	1 2 3 4 5 6 7 8 9 10 11 12 13 14 15 16 17 18 19 20 21 22 23 24
TEN	20	1 10 11 12 13 14 15 16 17 18 19 2 20 3 4 5 6 7 8 9
ANI	2	c s

Dimensions	
Covariance Parameters	3
Columns in X	63
Columns in Z	0
Subjects	436
Max Obs per Subject	1

Number of Observations	
Number of Observations Read	436
Number of Observations Used	436
Number of Observations Not Used	0

Iteration History			
Iteration	Evaluations	-2 Res Log Like	Criterion
0	1	3393.82399168	
1	1	3335.79248483	0.00000000

Convergence criteria met but final Hessian is not positive definite.

Estimated R Matrix for RP(TEN) 1 1	
Row	Col1
1	359.15

The Mixed Procedure

Estimated R Correlation Matrix for RP(TEN) 1 1	
Row	Col1
1	1.0000

Covariance Parameter Estimates		
Cov Parm	Subject	Estimate
UN(1,1)	RP(TEN)	359.15
UN(2,1)	RP(TEN)	0
UN(2,2)	RP(TEN)	117.51

Fit Statistics	
-2 Res Log Likelihood	3335.8
AIC (Smaller is Better)	3341.8
AICC (Smaller is Better)	3341.9
BIC (Smaller is Better)	3354.0

Null Model Likelihood Ratio Test		
DF	Chi-Square	Pr > ChiSq
2	58.03	<.0001

Type 3 Tests of Fixed Effects				
Effect	Num DF	Den DF	F Value	Pr > F
TEN	19	396	0.71	0.8117
ANI	1	396	5.09	0.0246
TEN*ANI	19	396	0.91	0.5709

The Mixed Procedure

Model Information	
**Data Set**	WORK.AU
**Dependent Variable**	Y
**Covariance Structure**	Unstructured using Correlations
**Subject Effect**	RP(TEN)
**Estimation Method**	REML
**Residual Variance Method**	None
**Fixed Effects SE Method**	Model-Based
**Degrees of Freedom Method**	Between-Within

Class Level Information		
**Class**	**Levels**	**Values**
**RP**	24	1 2 3 4 5 6 7 8 9 10 11 12 13 14 15 16 17 18 19 20 21 22 23 24
**TEN**	20	1 10 11 12 13 14 15 16 17 18 19 2 20 3 4 5 6 7 8 9
**ANI**	2	c s

Dimensions	
**Covariance Parameters**	3
**Columns in X**	63
**Columns in Z**	0
**Subjects**	436
**Max Obs per Subject**	1

Number of Observations	
**Number of Observations Read**	436
**Number of Observations Used**	436
**Number of Observations Not Used**	0

Iteration History			
**Iteration**	**Evaluations**	**-2 Res Log Like**	**Criterion**
**0**	1	3393.82399168	
**1**	2	3352.26600629	0.00005523
**2**	1	3338.68149981	0.00000545
**3**	1	3335.96327818	0.00000023
**4**	1	3335.79344292	0.00000000

Convergence criteria met but final Hessian is not positive definite.

The Mixed Procedure

Estimated R Matrix for RP(TEN) 1 1	
**Row**	**Col1**
**1**	358.05

Estimated R Correlation Matrix for RP(TEN) 1 1	
**Row**	**Col1**
**1**	1.0000

Covariance Parameter Estimates		
**Cov Parm**	**Subject**	**Estimate**
**Var(1)**	RP(TEN)	358.05
**Var(2)**	RP(TEN)	117.51
**Corr(2,1)**	RP(TEN)	0

Fit Statistics	
**-2 Res Log Likelihood**	3335.8
**AIC (Smaller is Better)**	3341.8
**AICC (Smaller is Better)**	3341.9
**BIC (Smaller is Better)**	3354.0

Null Model Likelihood Ratio Test		
**DF**	**Chi-Square**	**Pr > ChiSq**
2	58.03	<.0001

Type 3 Tests of Fixed Effects				
**Effect**	**Num DF**	**Den DF**	**F Value**	**Pr > F**
**TEN**	19	396	0.71	0.8101
**ANI**	1	396	5.10	0.0244
**TEN*ANI**	19	396	0.91	0.5683

The Mixed Procedure

Model Information	
**Data Set**	WORK.AU
**Dependent Variable**	Y
**Covariance Structure**	Variance Components
**Subject Effect**	RP(TEN)
**Estimation Method**	REML
**Residual Variance Method**	Parameter
**Fixed Effects SE Method**	Model-Based
**Degrees of Freedom Method**	Between-Within

Class Level Information		
**Class**	**Levels**	**Values**
**RP**	24	1 2 3 4 5 6 7 8 9 10 11 12 13 14 15 16 17 18 19 20 21 22 23 24
**TEN**	20	1 10 11 12 13 14 15 16 17 18 19 2 20 3 4 5 6 7 8 9
**ANI**	2	c s

Dimensions	
**Covariance Parameters**	1
**Columns in X**	63
**Columns in Z**	0
**Subjects**	436
**Max Obs per Subject**	1

Number of Observations	
**Number of Observations Read**	436
**Number of Observations Used**	436
**Number of Observations Not Used**	0

Iteration History			
**Iteration**	**Evaluations**	**-2 Res Log Like**	**Criterion**
**0**	1	3393.82399168	
**1**	1	3393.82399168	0.00000000

Convergence criteria met.

Estimated R Matrix for RP(TEN) 1 1	
**Row**	**Col1**
**1**	242.60

The Mixed Procedure

Estimated R Correlation Matrix for RP(TEN) 1 1	
Row	Col1
1	1.0000

Covariance Parameter Estimates		
Cov Parm	Subject	Estimate
ANI	RP(TEN)	242.60

Fit Statistics	
-2 Res Log Likelihood	3393.8
AIC (Smaller is Better)	3395.8
AICC (Smaller is Better)	3395.8
BIC (Smaller is Better)	3399.9

Null Model Likelihood Ratio Test		
DF	Chi-Square	Pr > ChiSq
0	0.00	1.0000

Type 3 Tests of Fixed Effects				
Effect	Num DF	Den DF	F Value	Pr > F
TEN	19	396	0.69	0.8337
ANI	1	396	4.91	0.0272
TEN*ANI	19	396	0.88	0.6129

Obs	Descr	V_ANTE	V_AR	V_ARH	V_ARMA	V_CS	V_CSH	V_FA1
1	-2 Res Log Likelihood	3335.8	3393.8	3335.8	3393.8	3393.8	3335.8	3335.8
2	AIC (Smaller is Better)	3341.8	3397.8	3341.8	3399.8	3397.8	3341.8	3343.8
3	AICC (Smaller is Better)	3341.9	3397.9	3341.9	3399.9	3397.9	3341.9	3343.9
4	BIC (Smaller is Better)	3354.0	3406.0	3354.0	3412.1	3406.0	3354.0	3360.1

Obs	V_HF	V_SIMPLE	V_TOEP	V_TOEPH	V_UN	V_UNR	V_VC
1	3335.8	3393.8	3393.8	3335.8	3335.8	3335.8	3393.8
2	3341.8	3395.8	3397.8	3341.8	3341.8	3341.8	3395.8
3	3341.9	3395.8	3397.9	3341.9	3341.9	3341.9	3395.8
4	3354.0	3399.9	3406.0	3354.0	3354.0	3354.0	3399.9

Obs	ANI	TEN	RP	Y
1	c	1	1	17
2	c	2	1	31
3	c	3	1	46
4	c	4	1	46
5	c	5	1	35
6	c	6	1	10
7	c	7	1	43
8	c	8	1	26
9	c	9	1	32
10	c	10	1	149
11	c	11	1	28
12	c	12	1	28
13	c	13	1	16
14	c	14	1	40
15	c	15	1	24
16	c	16	1	14
17	c	17	1	12
18	c	18	1	32
19	c	19	1	0
20	c	20	1	42
21	c	1	2	24
22	c	2	2	44
23	c	3	2	14
24	c	4	2	19
25	c	5	2	69
26	c	6	2	46
27	c	7	2	48
28	c	8	2	10
29	c	9	2	5
30	c	10	2	132
31	c	11	2	37
32	c	12	2	30
33	c	13	2	26
34	c	14	2	59
35	c	15	2	52
36	c	16	2	24
37	c	17	2	26
38	c	18	2	25
39	c	19	2	16
40	c	20	2	48
41	c	1	3	0

Obs	ANI	TEN	RP	Y
**42**	c	2	3	16
**43**	c	3	3	0
**44**	c	4	3	12
**45**	c	5	3	38
**46**	c	6	3	0
**47**	c	7	3	4
**48**	c	8	3	0
**49**	c	9	3	0
**50**	c	10	3	36
**51**	c	11	3	10
**52**	c	12	3	2
**53**	c	13	3	4
**54**	c	14	3	6
**55**	c	15	3	32
**56**	c	16	3	8
**57**	c	17	3	14
**58**	c	18	3	18
**59**	c	19	3	8
**60**	c	20	3	18
**61**	c	1	4	14
**62**	c	2	4	6
**63**	c	3	4	26
**64**	c	4	4	0
**65**	c	5	4	2
**66**	c	6	4	0
**67**	c	7	4	16
**68**	c	8	4	15
**69**	c	9	4	0
**70**	c	10	4	12
**71**	c	11	4	38
**72**	c	12	4	12
**73**	c	13	4	0
**74**	c	14	4	56
**75**	c	15	4	4
**76**	c	16	4	19
**77**	c	17	4	12
**78**	c	18	4	14
**79**	c	19	4	10
**80**	c	20	4	30
**81**	c	1	5	11
**82**	c	2	5	28

Obs	ANI	TEN	RP	Y
**83**	c	3	5	28
**84**	c	4	5	30
**85**	c	5	5	20
**86**	c	6	5	8
**87**	c	7	5	15
**88**	c	8	5	12
**89**	c	9	5	18
**90**	c	10	5	22
**91**	c	11	5	31
**92**	c	12	5	5
**93**	c	13	5	7
**94**	c	14	5	12
**95**	c	15	5	10
**96**	c	16	5	3
**97**	c	17	5	6
**98**	c	18	5	3
**99**	c	19	5	4
**100**	c	20	5	19
**101**	c	1	6	7
**102**	c	2	6	2
**103**	c	3	6	2
**104**	c	4	6	8
**105**	c	5	6	0
**106**	c	6	6	4
**107**	c	7	6	6
**108**	c	8	6	21
**109**	c	9	6	11
**110**	c	10	6	0
**111**	c	11	6	24
**112**	c	12	6	15
**113**	c	13	6	10
**114**	c	14	6	3
**115**	c	15	6	3
**116**	c	16	6	0
**117**	c	17	6	0
**118**	c	18	6	1
**119**	c	19	6	15
**120**	c	20	6	13
**121**	c	1	7	5
**122**	c	2	7	0
**123**	c	3	7	0

Obs	ANI	TEN	RP	Y
**124**	c	4	7	0
**125**	c	5	7	0
**126**	c	6	7	11
**127**	c	7	7	15
**128**	c	8	7	5
**129**	c	9	7	0
**130**	c	10	7	3
**131**	c	11	7	0
**132**	c	12	7	0
**133**	c	13	7	0
**134**	c	14	7	20
**135**	c	15	7	0
**136**	c	16	7	11
**137**	c	17	7	15
**138**	c	18	7	5
**139**	c	19	7	0
**140**	c	20	7	3
**141**	c	1	8	0
**142**	c	2	8	0
**143**	c	3	8	1
**144**	c	4	8	8
**145**	c	5	8	18
**146**	c	6	8	6
**147**	c	7	8	0
**148**	c	8	8	0
**149**	c	9	8	0
**150**	c	10	8	0
**151**	c	11	8	0
**152**	c	12	8	0
**153**	c	13	8	0
**154**	c	14	8	0
**155**	c	15	8	15
**156**	c	16	8	14
**157**	c	17	8	0
**158**	c	18	8	0
**159**	c	19	8	36
**160**	c	20	8	24
**161**	c	1	9	32
**162**	c	2	9	4
**163**	c	3	9	35
**164**	c	4	9	54

Obs	ANI	TEN	RP	Y
**165**	c	5	9	6
**166**	c	6	9	11
**167**	c	7	9	37
**168**	c	8	9	12
**169**	c	9	9	24
**170**	c	10	9	2
**171**	c	11	9	0
**172**	c	12	9	0
**173**	c	13	9	12
**174**	c	14	9	0
**175**	c	15	9	0
**176**	c	16	9	0
**177**	c	17	9	0
**178**	c	18	9	0
**179**	c	19	9	0
**180**	c	20	9	0
**181**	c	4	10	0
**182**	c	5	10	11
**183**	c	6	10	0
**184**	c	7	10	0
**185**	c	8	10	7
**186**	c	9	10	0
**187**	c	10	10	0
**188**	c	1	11	0
**189**	c	2	11	0
**190**	c	3	11	17
**191**	c	4	11	0
**192**	c	6	11	11
**193**	c	7	11	8
**194**	c	8	11	0
**195**	c	9	11	0
**196**	c	10	11	3
**197**	c	11	11	21
**198**	c	12	11	47
**199**	c	13	11	26
**200**	c	14	11	13
**201**	c	15	11	29
**202**	c	16	11	20
**203**	c	17	11	21
**204**	c	18	11	24
**205**	c	19	11	28

Obs	ANI	TEN	RP	Y
206	c	20	11	7
207	c	1	12	13
208	c	2	12	16
209	c	3	12	18
210	c	4	12	26
211	c	5	12	20
212	c	6	12	40
213	c	7	12	49
214	c	8	12	21
215	c	9	12	27
216	c	10	12	9
217	c	11	12	0
218	c	12	12	0
219	c	13	12	16
220	c	15	12	0
221	c	16	12	21
222	c	17	12	3
223	c	18	12	15
224	c	19	12	0
225	c	20	12	0
226	s	1	13	0
227	s	2	13	18
228	s	3	13	13
229	s	5	13	29
230	s	6	13	20
231	s	7	13	0
232	s	10	13	20
233	s	12	13	18
234	s	13	13	32
235	s	14	13	31
236	s	15	13	22
237	s	16	13	17
238	s	17	13	24
239	s	18	13	18
240	s	19	13	29
241	s	20	13	24
242	s	1	14	36
243	s	3	14	10
244	s	4	14	45
245	s	5	14	0
246	s	8	14	36

Obs	ANI	TEN	RP	Y
247	s	9	14	14
248	s	10	14	0
249	s	11	14	39
250	s	12	14	35
251	s	13	14	8
252	s	14	14	15
253	s	15	14	4
254	s	16	14	13
255	s	17	14	7
256	s	18	14	6
257	s	19	14	8
258	s	20	14	0
259	s	1	15	13
260	s	2	15	0
261	s	3	15	6
262	s	4	15	9
263	s	6	15	14
264	s	7	15	1
265	s	8	15	21
266	s	9	15	11
267	s	10	15	1
268	s	11	15	10
269	s	12	15	0
270	s	13	15	0
271	s	14	15	22
272	s	16	15	14
273	s	17	15	13
274	s	18	15	9
275	s	19	15	0
276	s	20	15	3
277	s	1	16	26
278	s	2	16	14
279	s	3	16	14
280	s	4	16	4
281	s	5	16	3
282	s	6	16	16
283	s	7	16	17
284	s	8	16	16
285	s	9	16	25
286	s	10	16	16
287	s	12	16	0

Obs	ANI	TEN	RP	Y
**288**	s	14	16	0
**289**	s	15	16	7
**290**	s	16	16	3
**291**	s	17	16	6
**292**	s	18	16	27
**293**	s	19	16	6
**294**	s	1	17	0
**295**	s	2	17	0
**296**	s	3	17	4
**297**	s	4	17	22
**298**	s	5	17	24
**299**	s	6	17	0
**300**	s	7	17	4
**301**	s	9	17	3
**302**	s	10	17	5
**303**	s	11	17	39
**304**	s	12	17	4
**305**	s	13	17	34
**306**	s	14	17	59
**307**	s	15	17	0
**308**	s	16	17	14
**309**	s	17	17	26
**310**	s	18	17	12
**311**	s	19	17	47
**312**	s	20	17	18
**313**	s	1	18	4
**314**	s	2	18	0
**315**	s	3	18	0
**316**	s	4	18	0
**317**	s	5	18	17
**318**	s	6	18	26
**319**	s	7	18	0
**320**	s	8	18	0
**321**	s	9	18	17
**322**	s	10	18	31
**323**	s	11	18	0
**324**	s	12	18	0
**325**	s	13	18	9
**326**	s	14	18	0
**327**	s	15	18	4
**328**	s	16	18	0

Obs	ANI	TEN	RP	Y
**329**	s	17	18	0
**330**	s	18	18	15
**331**	s	19	18	0
**332**	s	1	19	21
**333**	s	2	19	10
**334**	s	4	19	25
**335**	s	5	19	20
**336**	s	6	19	0
**337**	s	7	19	11
**338**	s	8	19	10
**339**	s	9	19	6
**340**	s	11	19	15
**341**	s	12	19	7
**342**	s	13	19	16
**343**	s	15	19	0
**344**	s	16	19	10
**345**	s	17	19	15
**346**	s	19	19	1
**347**	s	20	19	0
**348**	s	1	20	0
**349**	s	2	20	9
**350**	s	3	20	9
**351**	s	4	20	8
**352**	s	5	20	20
**353**	s	7	20	22
**354**	s	8	20	4
**355**	s	9	20	10
**356**	s	10	20	13
**357**	s	12	20	0
**358**	s	13	20	9
**359**	s	14	20	3
**360**	s	15	20	15
**361**	s	16	20	13
**362**	s	17	20	8
**363**	s	19	20	7
**364**	s	20	20	25
**365**	s	1	21	15
**366**	s	3	21	13
**367**	s	4	21	4
**368**	s	5	21	0
**369**	s	7	21	14

Obs	ANI	TEN	RP	Y
370	s	8	21	4
371	s	9	21	20
372	s	10	21	4
373	s	11	21	8
374	s	12	21	21
375	s	13	21	12
376	s	14	21	22
377	s	15	21	6
378	s	16	21	0
379	s	17	21	4
380	s	19	21	16
381	s	20	21	8
382	s	1	22	9
383	s	2	22	10
384	s	3	22	6
385	s	4	22	6
386	s	5	22	2
387	s	6	22	11
388	s	7	22	2
389	s	8	22	5
390	s	9	22	0
391	s	10	22	0
392	s	11	22	9
393	s	12	22	4
394	s	13	22	15
395	s	14	22	0
396	s	15	22	10
397	s	17	22	20
398	s	18	22	9
399	s	19	22	11
400	s	20	22	0
401	s	1	23	0
402	s	2	23	9
403	s	4	23	39
404	s	5	23	4
405	s	6	23	9
406	s	7	23	18
407	s	8	23	2
408	s	9	23	16
409	s	10	23	8
410	s	11	23	21

Obs	ANI	TEN	RP	Y
**411**	s	12	23	19
**412**	s	13	23	16
**413**	s	14	23	7
**414**	s	15	23	12
**415**	s	16	23	12
**416**	s	17	23	4
**417**	s	18	23	26
**418**	s	20	23	11
**419**	s	2	24	11
**420**	s	4	24	16
**421**	s	5	24	5
**422**	s	6	24	4
**423**	s	7	24	3
**424**	s	8	24	18
**425**	s	9	24	1
**426**	s	10	24	0
**427**	s	11	24	10
**428**	s	12	24	14
**429**	s	13	24	18
**430**	s	14	24	15
**431**	s	15	24	21
**432**	s	16	24	30
**433**	s	17	24	18
**434**	s	18	24	12
**435**	s	19	24	7
**436**	s	20	24	30

The Mixed Procedure

Model Information	
**Data Set**	WORK.AU
**Dependent Variable**	Y
**Covariance Structure**	Variance Components
**Subject Effect**	RP(TEN*ANI)
**Estimation Method**	REML
**Residual Variance Method**	Parameter
**Fixed Effects SE Method**	Model-Based
**Degrees of Freedom Method**	Between-Within

Class Level Information		
**Class**	**Levels**	**Values**
**TEN**	20	1 10 11 12 13 14 15 16 17 18 19 2 20 3 4 5 6 7 8 9
**ANI**	2	c s
**RP**	24	1 2 3 4 5 6 7 8 9 10 11 12 13 14 15 16 17 18 19 20 21 22 23 24

Dimensions	
**Covariance Parameters**	1
**Columns in X**	63
**Columns in Z**	0
**Subjects**	436
**Max Obs per Subject**	1

Number of Observations	
**Number of Observations Read**	436
**Number of Observations Used**	436
**Number of Observations Not Used**	0

Iteration History			
**Iteration**	**Evaluations**	**-2 Res Log Like**	**Criterion**
**0**	1	3393.82399168	
**1**	1	3393.82399168	0.00000000

Convergence criteria met.

Estimated R Matrix for RP(TEN*ANI) 1 1 c	
**Row**	**Col1**
**1**	242.60

The Mixed Procedure

Estimated R Correlation Matrix for RP(TEN*ANI) 1 1 c	
Row	Col1
1	1.0000

Covariance Parameter Estimates		
Cov Parm	Subject	Estimate
ANI	RP(TEN*ANI)	242.60

Fit Statistics	
-2 Res Log Likelihood	3393.8
AIC (Smaller is Better)	3395.8
AICC (Smaller is Better)	3395.8
BIC (Smaller is Better)	3399.9

Null Model Likelihood Ratio Test		
DF	Chi-Square	Pr > ChiSq
0	0.00	1.0000

Type 3 Tests of Fixed Effects				
Effect	Num DF	Den DF	F Value	Pr > F
TEN	19	396	0.69	0.8337
ANI	1	396	4.91	0.0272
TEN*ANI	19	396	0.88	0.6129

The Mixed Procedure

Least Squares Means							
Effect	TEN	ANI	Estimate	Standard Error	DF	t Value	Pr > |t|
TEN	1		11.2273	3.3207	396	3.38	0.0008
TEN	10		19.7879	3.2508	396	6.09	<.0001
TEN	11		16.9798	3.5003	396	4.85	<.0001
TEN	12		11.4015	3.2508	396	3.51	0.0005
TEN	13		13.0000	3.3207	396	3.91	0.0001
TEN	14		18.3591	3.4027	396	5.40	<.0001
TEN	15		12.2727	3.3207	396	3.70	0.0003
TEN	16		11.8182	3.3207	396	3.56	0.0004
TEN	17		10.9962	3.2508	396	3.38	0.0008
TEN	18		13.6717	3.5003	396	3.91	0.0001
TEN	19		11.3182	3.3207	396	3.41	0.0007
TEN	2		10.7318	3.4027	396	3.15	0.0017
TEN	20		15.2227	3.4027	396	4.47	<.0001
TEN	3		12.6667	3.5003	396	3.62	0.0003
TEN	4		16.5492	3.2508	396	5.09	<.0001
TEN	5		15.5909	3.3207	396	4.70	<.0001
TEN	6		11.6806	3.4341	396	3.40	0.0007
TEN	7		14.2235	3.2508	396	4.38	<.0001
TEN	8		11.1750	3.3345	396	3.35	0.0009
TEN	9		10.4659	3.2508	396	3.22	0.0014
ANI		c	15.1158	1.0395	396	14.54	<.0001
ANI		s	11.7981	1.0766	396	10.96	<.0001
TEN*ANI	1	c	11.1818	4.6962	396	2.38	0.0177
TEN*ANI	1	s	11.2727	4.6962	396	2.40	0.0168
TEN*ANI	10	c	30.6667	4.4963	396	6.82	<.0001
TEN*ANI	10	s	8.9091	4.6962	396	1.90	0.0585
TEN*ANI	11	c	17.1818	4.6962	396	3.66	0.0003
TEN*ANI	11	s	16.7778	5.1919	396	3.23	0.0013
TEN*ANI	12	c	12.6364	4.6962	396	2.69	0.0074
TEN*ANI	12	s	10.1667	4.4963	396	2.26	0.0243
TEN*ANI	13	c	10.6364	4.6962	396	2.26	0.0241
TEN*ANI	13	s	15.3636	4.6962	396	3.27	0.0012
TEN*ANI	14	c	20.9000	4.9254	396	4.24	<.0001
TEN*ANI	14	s	15.8182	4.6962	396	3.37	0.0008
TEN*ANI	15	c	15.3636	4.6962	396	3.27	0.0012
TEN*ANI	15	s	9.1818	4.6962	396	1.96	0.0513
TEN*ANI	16	c	12.1818	4.6962	396	2.59	0.0098
TEN*ANI	16	s	11.4545	4.6962	396	2.44	0.0152

The Mixed Procedure

Least Squares Means							
**Effect**	**TEN**	**ANI**	**Estimate**	**Standard Error**	**DF**	**t Value**	**Pr > |t|**
**TEN*ANI**	17	c	9.9091	4.6962	396	2.11	0.0355
**TEN*ANI**	17	s	12.0833	4.4963	396	2.69	0.0075
**TEN*ANI**	18	c	12.4545	4.6962	396	2.65	0.0083
**TEN*ANI**	18	s	14.8889	5.1919	396	2.87	0.0044
**TEN*ANI**	19	c	10.6364	4.6962	396	2.26	0.0241
**TEN*ANI**	19	s	12.0000	4.6962	396	2.56	0.0110
**TEN*ANI**	2	c	13.3636	4.6962	396	2.85	0.0047
**TEN*ANI**	2	s	8.1000	4.9254	396	1.64	0.1009
**TEN*ANI**	20	c	18.5455	4.6962	396	3.95	<.0001
**TEN*ANI**	20	s	11.9000	4.9254	396	2.42	0.0161
**TEN*ANI**	3	c	17.0000	4.6962	396	3.62	0.0003
**TEN*ANI**	3	s	8.3333	5.1919	396	1.61	0.1093
**TEN*ANI**	4	c	16.9167	4.4963	396	3.76	0.0002
**TEN*ANI**	4	s	16.1818	4.6962	396	3.45	0.0006
**TEN*ANI**	5	c	19.9091	4.6962	396	4.24	<.0001
**TEN*ANI**	5	s	11.2727	4.6962	396	2.40	0.0168
**TEN*ANI**	6	c	12.2500	4.4963	396	2.72	0.0067
**TEN*ANI**	6	s	11.1111	5.1919	396	2.14	0.0330
**TEN*ANI**	7	c	20.0833	4.4963	396	4.47	<.0001
**TEN*ANI**	7	s	8.3636	4.6962	396	1.78	0.0757
**TEN*ANI**	8	c	10.7500	4.4963	396	2.39	0.0173
**TEN*ANI**	8	s	11.6000	4.9254	396	2.36	0.0190
**TEN*ANI**	9	c	9.7500	4.4963	396	2.17	0.0307
**TEN*ANI**	9	s	11.1818	4.6962	396	2.38	0.0177
**TEN**	1		11.2273	3.3207	396	3.38	0.0008
**TEN**	10		19.7879	3.2508	396	6.09	<.0001
**TEN**	11		16.9798	3.5003	396	4.85	<.0001
**TEN**	12		11.4015	3.2508	396	3.51	0.0005
**TEN**	13		13.0000	3.3207	396	3.91	0.0001
**TEN**	14		18.3591	3.4027	396	5.40	<.0001
**TEN**	15		12.2727	3.3207	396	3.70	0.0003
**TEN**	16		11.8182	3.3207	396	3.56	0.0004
**TEN**	17		10.9962	3.2508	396	3.38	0.0008
**TEN**	18		13.6717	3.5003	396	3.91	0.0001
**TEN**	19		11.3182	3.3207	396	3.41	0.0007
**TEN**	2		10.7318	3.4027	396	3.15	0.0017
**TEN**	20		15.2227	3.4027	396	4.47	<.0001
**TEN**	3		12.6667	3.5003	396	3.62	0.0003

The Mixed Procedure

Least Squares Means							
Effect	TEN	ANI	Estimate	Standard Error	DF	t Value	Pr > |t|
TEN	4		16.5492	3.2508	396	5.09	<.0001
TEN	5		15.5909	3.3207	396	4.70	<.0001
TEN	6		11.6806	3.4341	396	3.40	0.0007
TEN	7		14.2235	3.2508	396	4.38	<.0001
TEN	8		11.1750	3.3345	396	3.35	0.0009
TEN	9		10.4659	3.2508	396	3.22	0.0014
ANI		c	15.1158	1.0395	396	14.54	<.0001
ANI		s	11.7981	1.0766	396	10.96	<.0001
TEN*ANI	1	c	11.1818	4.6962	396	2.38	0.0177
TEN*ANI	1	s	11.2727	4.6962	396	2.40	0.0168
TEN*ANI	10	c	30.6667	4.4963	396	6.82	<.0001
TEN*ANI	10	s	8.9091	4.6962	396	1.90	0.0585
TEN*ANI	11	c	17.1818	4.6962	396	3.66	0.0003
TEN*ANI	11	s	16.7778	5.1919	396	3.23	0.0013
TEN*ANI	12	c	12.6364	4.6962	396	2.69	0.0074
TEN*ANI	12	s	10.1667	4.4963	396	2.26	0.0243
TEN*ANI	13	c	10.6364	4.6962	396	2.26	0.0241
TEN*ANI	13	s	15.3636	4.6962	396	3.27	0.0012
TEN*ANI	14	c	20.9000	4.9254	396	4.24	<.0001
TEN*ANI	14	s	15.8182	4.6962	396	3.37	0.0008
TEN*ANI	15	c	15.3636	4.6962	396	3.27	0.0012
TEN*ANI	15	s	9.1818	4.6962	396	1.96	0.0513
TEN*ANI	16	c	12.1818	4.6962	396	2.59	0.0098
TEN*ANI	16	s	11.4545	4.6962	396	2.44	0.0152
TEN*ANI	17	c	9.9091	4.6962	396	2.11	0.0355
TEN*ANI	17	s	12.0833	4.4963	396	2.69	0.0075
TEN*ANI	18	c	12.4545	4.6962	396	2.65	0.0083
TEN*ANI	18	s	14.8889	5.1919	396	2.87	0.0044
TEN*ANI	19	c	10.6364	4.6962	396	2.26	0.0241
TEN*ANI	19	s	12.0000	4.6962	396	2.56	0.0110
TEN*ANI	2	c	13.3636	4.6962	396	2.85	0.0047
TEN*ANI	2	s	8.1000	4.9254	396	1.64	0.1009
TEN*ANI	20	c	18.5455	4.6962	396	3.95	<.0001
TEN*ANI	20	s	11.9000	4.9254	396	2.42	0.0161
TEN*ANI	3	c	17.0000	4.6962	396	3.62	0.0003
TEN*ANI	3	s	8.3333	5.1919	396	1.61	0.1093
TEN*ANI	4	c	16.9167	4.4963	396	3.76	0.0002
TEN*ANI	4	s	16.1818	4.6962	396	3.45	0.0006

The Mixed Procedure

Least Squares Means							
Effect	TEN	ANI	Estimate	Standard Error	DF	t Value	Pr > |t|
TEN*ANI	5	c	19.9091	4.6962	396	4.24	<.0001
TEN*ANI	5	s	11.2727	4.6962	396	2.40	0.0168
TEN*ANI	6	c	12.2500	4.4963	396	2.72	0.0067
TEN*ANI	6	s	11.1111	5.1919	396	2.14	0.0330
TEN*ANI	7	c	20.0833	4.4963	396	4.47	<.0001
TEN*ANI	7	s	8.3636	4.6962	396	1.78	0.0757
TEN*ANI	8	c	10.7500	4.4963	396	2.39	0.0173
TEN*ANI	8	s	11.6000	4.9254	396	2.36	0.0190
TEN*ANI	9	c	9.7500	4.4963	396	2.17	0.0307
TEN*ANI	9	s	11.1818	4.6962	396	2.38	0.0177
TEN*ANI	1	c	11.1818	4.6962	396	2.38	0.0177
TEN*ANI	1	s	11.2727	4.6962	396	2.40	0.0168
TEN*ANI	10	c	30.6667	4.4963	396	6.82	<.0001
TEN*ANI	10	s	8.9091	4.6962	396	1.90	0.0585
TEN*ANI	11	c	17.1818	4.6962	396	3.66	0.0003
TEN*ANI	11	s	16.7778	5.1919	396	3.23	0.0013
TEN*ANI	12	c	12.6364	4.6962	396	2.69	0.0074
TEN*ANI	12	s	10.1667	4.4963	396	2.26	0.0243
TEN*ANI	13	c	10.6364	4.6962	396	2.26	0.0241
TEN*ANI	13	s	15.3636	4.6962	396	3.27	0.0012
TEN*ANI	14	c	20.9000	4.9254	396	4.24	<.0001
TEN*ANI	14	s	15.8182	4.6962	396	3.37	0.0008
TEN*ANI	15	c	15.3636	4.6962	396	3.27	0.0012
TEN*ANI	15	s	9.1818	4.6962	396	1.96	0.0513
TEN*ANI	16	c	12.1818	4.6962	396	2.59	0.0098
TEN*ANI	16	s	11.4545	4.6962	396	2.44	0.0152
TEN*ANI	17	c	9.9091	4.6962	396	2.11	0.0355
TEN*ANI	17	s	12.0833	4.4963	396	2.69	0.0075
TEN*ANI	18	c	12.4545	4.6962	396	2.65	0.0083
TEN*ANI	18	s	14.8889	5.1919	396	2.87	0.0044
TEN*ANI	19	c	10.6364	4.6962	396	2.26	0.0241
TEN*ANI	19	s	12.0000	4.6962	396	2.56	0.0110
TEN*ANI	2	c	13.3636	4.6962	396	2.85	0.0047
TEN*ANI	2	s	8.1000	4.9254	396	1.64	0.1009
TEN*ANI	20	c	18.5455	4.6962	396	3.95	<.0001
TEN*ANI	20	s	11.9000	4.9254	396	2.42	0.0161
TEN*ANI	3	c	17.0000	4.6962	396	3.62	0.0003
TEN*ANI	3	s	8.3333	5.1919	396	1.61	0.1093

The Mixed Procedure

Least Squares Means							
Effect	TEN	ANI	Estimate	Standard Error	DF	t Value	Pr > |t|
TEN*ANI	4	c	16.9167	4.4963	396	3.76	0.0002
TEN*ANI	4	s	16.1818	4.6962	396	3.45	0.0006
TEN*ANI	5	c	19.9091	4.6962	396	4.24	<.0001
TEN*ANI	5	s	11.2727	4.6962	396	2.40	0.0168
TEN*ANI	6	c	12.2500	4.4963	396	2.72	0.0067
TEN*ANI	6	s	11.1111	5.1919	396	2.14	0.0330
TEN*ANI	7	c	20.0833	4.4963	396	4.47	<.0001
TEN*ANI	7	s	8.3636	4.6962	396	1.78	0.0757
TEN*ANI	8	c	10.7500	4.4963	396	2.39	0.0173
TEN*ANI	8	s	11.6000	4.9254	396	2.36	0.0190
TEN*ANI	9	c	9.7500	4.4963	396	2.17	0.0307
TEN*ANI	9	s	11.1818	4.6962	396	2.38	0.0177

Tests of Effect Slices						
Effect	TEN	ANI	Num DF	Den DF	F Value	Pr > F
TEN*ANI	1		1	396	0.00	0.9891
TEN*ANI	10		1	396	11.20	0.0009
TEN*ANI	11		1	396	0.00	0.9540
TEN*ANI	12		1	396	0.14	0.7043
TEN*ANI	13		1	396	0.51	0.4770
TEN*ANI	14		1	396	0.56	0.4557
TEN*ANI	15		1	396	0.87	0.3525
TEN*ANI	16		1	396	0.01	0.9129
TEN*ANI	17		1	396	0.11	0.7382
TEN*ANI	18		1	396	0.12	0.7282
TEN*ANI	19		1	396	0.04	0.8374
TEN*ANI	2		1	396	0.60	0.4397
TEN*ANI	20		1	396	0.95	0.3294
TEN*ANI	3		1	396	1.53	0.2165
TEN*ANI	4		1	396	0.01	0.9101
TEN*ANI	5		1	396	1.69	0.1942
TEN*ANI	6		1	396	0.03	0.8684
TEN*ANI	7		1	396	3.25	0.0722
TEN*ANI	8		1	396	0.02	0.8986
TEN*ANI	9		1	396	0.05	0.8258
TEN*ANI		c	19	396	1.28	0.1926
TEN*ANI		s	19	396	0.31	0.9980
