# Supplementary material for: Learning curve for the laparoscopy-guided kidney biopsy procedure in small corpses of dogs and pigs
Source: PLoS One. 2021 Sep 27;16(9):e0257653. doi: 10.1371/journal.pone.0257653 (PMC8475998; doi:10.1371/journal.pone.0257653)
Supplement: S3 Raw data — (PDF) [file pone.0257653.s003.pdf]

Obs	ANI	TEN	RP	Y
1	c	1	1	178
2	c	2	1	83
3	c	3	1	76
4	c	4	1	63
5	c	5	1	84
6	c	6	1	79
7	c	7	1	51
8	c	8	1	35
9	c	9	1	84
10	c	10	1	59
11	c	11	1	57
12	c	12	1	55
13	c	13	1	45
14	c	14	1	55
15	c	15	1	50
16	c	16	1	82
17	c	17	1	45
18	c	18	1	50
19	c	19	1	58
20	c	20	1	62
21	c	1	2	77
22	c	2	2	40
23	c	3	2	32
24	c	4	2	30
25	c	5	2	32
26	c	6	2	27
27	c	7	2	29
28	c	8	2	28
29	c	9	2	30
30	c	10	2	27
31	c	11	2	38
32	c	12	2	46
33	c	13	2	29
34	c	14	2	44
35	c	15	2	40
36	c	16	2	46
37	c	17	2	42
38	c	18	2	37
39	c	19	2	30
40	c	20	2	33
41	c	1	3	63

Obs	ANI	TEN	RP	Y
42	c	2	3	48
43	c	3	3	48
44	c	4	3	73
45	c	5	3	36
46	c	6	3	22
47	c	7	3	31
48	c	8	3	45
49	c	9	3	35
50	c	10	3	27
51	c	11	3	25
52	c	12	3	33
53	c	13	3	21
54	c	14	3	16
55	c	15	3	12
56	c	16	3	29
57	c	17	3	20
58	c	18	3	34
59	c	19	3	32
60	c	20	3	37
61	c	1	4	99
62	c	2	4	35
63	c	3	4	46
64	c	4	4	35
65	c	5	4	29
66	c	6	4	46
67	c	7	4	25
68	c	8	4	29
69	c	9	4	22
70	c	10	4	44
71	c	11	4	24
72	c	12	4	19
73	c	13	4	21
74	c	14	4	61
75	c	15	4	42
76	c	16	4	36
77	c	17	4	29
78	c	18	4	19
79	c	19	4	18
80	c	20	4	23
81	c	1	5	19
82	c	2	5	16

Obs	ANI	TEN	RP	Y
83	c	3	5	27
84	c	4	5	27
85	c	5	5	27
86	c	6	5	12
87	c	7	5	9
88	c	8	5	17
89	c	9	5	14
90	c	10	5	20
91	c	11	5	17
92	c	12	5	8
93	c	13	5	11
94	c	14	5	172
95	c	15	5	12
96	c	16	5	9
97	c	17	5	12
98	c	18	5	14
99	c	19	5	20
100	c	20	5	13
101	c	1	6	18
102	c	2	6	28
103	c	3	6	4
104	c	4	6	6
105	c	5	6	34
106	c	6	6	8
107	c	7	6	8
108	c	8	6	13
109	c	9	6	2
110	c	10	6	10
111	c	11	6	13
112	c	12	6	19
113	c	13	6	20
114	c	14	6	16
115	c	15	6	20
116	c	16	6	18
117	c	17	6	17
118	c	18	6	9
119	c	19	6	18
120	c	20	6	13
121	c	1	7	15
122	c	2	7	16
123	c	3	7	17

Obs	ANI	TEN	RP	Y
124	c	4	7	26
125	c	5	7	13
126	c	6	7	14
127	c	7	7	11
128	c	8	7	7
129	c	9	7	16
130	c	10	7	19
131	c	11	7	53
132	c	12	7	25
133	c	13	7	44
134	c	14	7	12
135	c	15	7	19
136	c	16	7	16
137	c	17	7	22
138	c	18	7	19
139	c	19	7	20
140	c	20	7	19
141	c	1	8	65
142	c	2	8	68
143	c	3	8	26
144	c	4	8	32
145	c	5	8	27
146	c	6	8	28
147	c	7	8	33
148	c	8	8	29
149	c	9	8	26
150	c	10	8	15
151	c	11	8	21
152	c	12	8	23
153	c	13	8	22
154	c	14	8	33
155	c	15	8	25
156	c	16	8	22
157	c	17	8	20
158	c	18	8	33
159	c	19	8	9
160	c	20	8	23
161	c	1	9	32
162	c	2	9	25
163	c	3	9	16
164	c	4	9	12

Obs	ANI	TEN	RP	Y
165	c	5	9	24
166	c	6	9	16
167	c	7	9	17
168	c	8	9	10
169	c	9	9	12
170	c	10	9	10
171	c	11	9	11
172	c	12	9	11
173	c	13	9	15
174	c	14	9	24
175	c	15	9	15
176	c	16	9	16
177	c	17	9	13
178	c	18	9	11
179	c	19	9	17
180	c	20	9	31
181	c	1	10	12
182	c	2	10	18
183	c	3	10	6
184	c	4	10	11
185	c	5	10	16
186	c	6	10	11
187	c	7	10	5
188	c	8	10	5
189	c	9	10	7
190	c	10	10	14
191	c	11	10	.
192	c	13	10	.
193	c	15	10	.
194	c	17	10	.
195	c	19	10	.
196	c	1	11	30
197	c	2	11	21
198	c	3	11	29
199	c	4	11	20
200	c	5	11	14
201	c	6	11	13
202	c	7	11	13
203	c	8	11	18
204	c	9	11	11
205	c	10	11	17

Obs	ANI	TEN	RP	Y
**206**	c	11	11	62
**207**	c	12	11	47
**208**	c	13	11	25
**209**	c	14	11	11
**210**	c	15	11	20
**211**	c	16	11	19
**212**	c	17	11	95
**213**	c	18	11	21
**214**	c	19	11	14
**215**	c	20	11	33
**216**	c	1	12	28
**217**	c	2	12	24
**218**	c	3	12	28
**219**	c	4	12	23
**220**	c	5	12	24
**221**	c	6	12	33
**222**	c	7	12	12
**223**	c	8	12	18
**224**	c	9	12	14
**225**	c	10	12	32
**226**	c	11	12	30
**227**	c	12	12	35
**228**	c	13	12	30
**229**	c	15	12	31
**230**	c	16	12	9
**231**	c	17	12	34
**232**	c	18	12	26
**233**	c	19	12	15
**234**	c	20	12	20
**235**	s	1	13	10
**236**	s	2	13	24
**237**	s	3	13	21
**238**	s	4	13	12
**239**	s	5	13	16
**240**	s	6	13	12
**241**	s	7	13	21
**242**	s	8	13	14
**243**	s	9	13	13
**244**	s	10	13	7
**245**	s	12	13	13
**246**	s	13	13	14

Obs	ANI	TEN	RP	Y
**247**	s	14	13	13
**248**	s	15	13	16
**249**	s	16	13	6
**250**	s	17	13	12
**251**	s	18	13	15
**252**	s	19	13	22
**253**	s	20	13	13
**254**	s	1	14	19
**255**	s	2	14	16
**256**	s	3	14	27
**257**	s	4	14	27
**258**	s	5	14	12
**259**	s	6	14	9
**260**	s	8	14	14
**261**	s	9	14	20
**262**	s	10	14	13
**263**	s	11	14	17
**264**	s	12	14	8
**265**	s	13	14	10
**266**	s	14	14	2
**267**	s	15	14	11
**268**	s	16	14	15
**269**	s	17	14	16
**270**	s	18	14	5
**271**	s	19	14	10
**272**	s	20	14	7
**273**	s	1	15	18
**274**	s	2	15	18
**275**	s	3	15	4
**276**	s	4	15	6
**277**	s	6	15	8
**278**	s	7	15	8
**279**	s	8	15	13
**280**	s	9	15	1
**281**	s	10	15	10
**282**	s	11	15	13
**283**	s	12	15	19
**284**	s	13	15	20
**285**	s	14	15	19
**286**	s	15	15	20
**287**	s	16	15	18

Obs	ANI	TEN	RP	Y
**288**	s	17	15	17
**289**	s	18	15	9
**290**	s	19	15	18
**291**	s	20	15	13
**292**	s	1	16	15
**293**	s	2	16	16
**294**	s	3	16	17
**295**	s	4	16	26
**296**	s	5	16	13
**297**	s	6	16	14
**298**	s	7	16	11
**299**	s	8	16	7
**300**	s	9	16	16
**301**	s	10	16	19
**302**	s	11	16	53
**303**	s	12	16	25
**304**	s	13	16	44
**305**	s	14	16	12
**306**	s	15	16	17
**307**	s	16	16	16
**308**	s	17	16	22
**309**	s	18	16	19
**310**	s	19	16	20
**311**	s	20	16	19
**312**	s	1	17	60
**313**	s	2	17	68
**314**	s	3	17	26
**315**	s	4	17	32
**316**	s	5	17	27
**317**	s	6	17	28
**318**	s	7	17	33
**319**	s	9	17	26
**320**	s	10	17	15
**321**	s	11	17	21
**322**	s	12	17	23
**323**	s	13	17	22
**324**	s	14	17	25
**325**	s	15	17	22
**326**	s	16	17	20
**327**	s	17	17	33
**328**	s	18	17	9

Obs	ANI	TEN	RP	Y
**329**	s	19	17	27
**330**	s	20	17	23
**331**	s	1	18	32
**332**	s	2	18	25
**333**	s	3	18	16
**334**	s	4	18	12
**335**	s	5	18	12
**336**	s	6	18	16
**337**	s	7	18	17
**338**	s	8	18	10
**339**	s	9	18	12
**340**	s	10	18	10
**341**	s	11	18	11
**342**	s	12	18	11
**343**	s	13	18	15
**344**	s	14	18	15
**345**	s	15	18	16
**346**	s	16	18	13
**347**	s	17	18	11
**348**	s	18	18	17
**349**	s	19	18	36
**350**	s	20	18	31
**351**	s	1	19	30
**352**	s	2	19	25
**353**	s	4	19	15
**354**	s	5	19	12
**355**	s	6	19	25
**356**	s	7	19	17
**357**	s	8	19	18
**358**	s	9	19	35
**359**	s	10	19	29
**360**	s	11	19	16
**361**	s	12	19	17
**362**	s	13	19	4
**363**	s	15	19	11
**364**	s	16	19	12
**365**	s	17	19	26
**366**	s	19	19	14
**367**	s	20	19	28
**368**	s	1	20	22
**369**	s	2	20	52

Obs	ANI	TEN	RP	Y
**370**	s	3	20	59
**371**	s	4	20	24
**372**	s	5	20	18
**373**	s	7	20	29
**374**	s	8	20	25
**375**	s	9	20	20
**376**	s	10	20	18
**377**	s	12	20	20
**378**	s	13	20	24
**379**	s	14	20	24
**380**	s	15	20	28
**381**	s	16	20	27
**382**	s	17	20	24
**383**	s	18	20	12
**384**	s	19	20	24
**385**	s	20	20	16
**386**	s	1	21	36
**387**	s	2	21	33
**388**	s	3	21	20
**389**	s	4	21	10
**390**	s	5	21	16
**391**	s	6	21	13
**392**	s	7	21	25
**393**	s	8	21	12
**394**	s	9	21	18
**395**	s	10	21	17
**396**	s	11	21	13
**397**	s	12	21	5
**398**	s	13	21	13
**399**	s	14	21	13
**400**	s	15	21	21
**401**	s	16	21	25
**402**	s	17	21	21
**403**	s	18	21	17
**404**	s	19	21	8
**405**	s	20	21	17
**406**	s	1	22	56
**407**	s	2	22	20
**408**	s	3	22	21
**409**	s	4	22	31
**410**	s	5	22	41

Obs	ANI	TEN	RP	Y
**411**	s	6	22	27
**412**	s	7	22	9
**413**	s	8	22	7
**414**	s	9	22	17
**415**	s	10	22	12
**416**	s	11	22	72
**417**	s	12	22	16
**418**	s	13	22	14
**419**	s	14	22	36
**420**	s	15	22	22
**421**	s	16	22	48
**422**	s	17	22	8
**423**	s	18	22	15
**424**	s	19	22	14
**425**	s	20	22	28
**426**	s	1	23	55
**427**	s	2	23	31
**428**	s	3	23	36
**429**	s	4	23	38
**430**	s	5	23	14
**431**	s	6	23	17
**432**	s	7	23	9
**433**	s	8	23	18
**434**	s	9	23	6
**435**	s	10	23	8
**436**	s	11	23	12
**437**	s	12	23	43
**438**	s	13	23	17
**439**	s	14	23	25
**440**	s	15	23	35
**441**	s	16	23	16
**442**	s	17	23	11
**443**	s	18	23	18
**444**	s	19	23	8
**445**	s	20	23	22
**446**	s	1	24	79
**447**	s	2	24	29
**448**	s	3	24	27
**449**	s	4	24	40
**450**	s	5	24	26
**451**	s	6	24	25

Obs	ANI	TEN	RP	Y
**452**	s	7	24	46
**453**	s	8	24	19
**454**	s	9	24	40
**455**	s	10	24	16
**456**	s	11	24	21
**457**	s	12	24	15
**458**	s	13	24	24
**459**	s	14	24	19
**460**	s	15	24	20
**461**	s	16	24	8
**462**	s	17	24	23
**463**	s	18	24	19
**464**	s	19	24	14
**465**	s	20	24	14

Obs	ANI	TEN	RP	Y
1	c	1	1	178
2	c	2	1	83
3	c	3	1	76
4	c	4	1	63
5	c	5	1	84
6	c	6	1	79
7	c	7	1	51
8	c	8	1	35
9	c	9	1	84
10	c	10	1	59
11	c	11	1	57
12	c	12	1	55
13	c	13	1	45
14	c	14	1	55
15	c	15	1	50
16	c	16	1	82
17	c	17	1	45
18	c	18	1	50
19	c	19	1	58
20	c	20	1	62
21	c	1	2	77
22	c	2	2	40
23	c	3	2	32
24	c	4	2	30
25	c	5	2	32
26	c	6	2	27
27	c	7	2	29
28	c	8	2	28
29	c	9	2	30
30	c	10	2	27
31	c	11	2	38
32	c	12	2	46
33	c	13	2	29
34	c	14	2	44
35	c	15	2	40
36	c	16	2	46
37	c	17	2	42
38	c	18	2	37
39	c	19	2	30
40	c	20	2	33
41	c	1	3	63

Obs	ANI	TEN	RP	Y
**42**	c	2	3	48
**43**	c	3	3	48
**44**	c	4	3	73
**45**	c	5	3	36
**46**	c	6	3	22
**47**	c	7	3	31
**48**	c	8	3	45
**49**	c	9	3	35
**50**	c	10	3	27
**51**	c	11	3	25
**52**	c	12	3	33
**53**	c	13	3	21
**54**	c	14	3	16
**55**	c	15	3	12
**56**	c	16	3	29
**57**	c	17	3	20
**58**	c	18	3	34
**59**	c	19	3	32
**60**	c	20	3	37
**61**	c	1	4	99
**62**	c	2	4	35
**63**	c	3	4	46
**64**	c	4	4	35
**65**	c	5	4	29
**66**	c	6	4	46
**67**	c	7	4	25
**68**	c	8	4	29
**69**	c	9	4	22
**70**	c	10	4	44
**71**	c	11	4	24
**72**	c	12	4	19
**73**	c	13	4	21
**74**	c	14	4	61
**75**	c	15	4	42
**76**	c	16	4	36
**77**	c	17	4	29
**78**	c	18	4	19
**79**	c	19	4	18
**80**	c	20	4	23
**81**	c	1	5	19
**82**	c	2	5	16

Obs	ANI	TEN	RP	Y
**83**	c	3	5	27
**84**	c	4	5	27
**85**	c	5	5	27
**86**	c	6	5	12
**87**	c	7	5	9
**88**	c	8	5	17
**89**	c	9	5	14
**90**	c	10	5	20
**91**	c	11	5	17
**92**	c	12	5	8
**93**	c	13	5	11
**94**	c	14	5	172
**95**	c	15	5	12
**96**	c	16	5	9
**97**	c	17	5	12
**98**	c	18	5	14
**99**	c	19	5	20
**100**	c	20	5	13
**101**	c	1	6	18
**102**	c	2	6	28
**103**	c	3	6	4
**104**	c	4	6	6
**105**	c	5	6	34
**106**	c	6	6	8
**107**	c	7	6	8
**108**	c	8	6	13
**109**	c	9	6	2
**110**	c	10	6	10
**111**	c	11	6	13
**112**	c	12	6	19
**113**	c	13	6	20
**114**	c	14	6	16
**115**	c	15	6	20
**116**	c	16	6	18
**117**	c	17	6	17
**118**	c	18	6	9
**119**	c	19	6	18
**120**	c	20	6	13
**121**	c	1	7	15
**122**	c	2	7	16
**123**	c	3	7	17

Obs	ANI	TEN	RP	Y
**124**	c	4	7	26
**125**	c	5	7	13
**126**	c	6	7	14
**127**	c	7	7	11
**128**	c	8	7	7
**129**	c	9	7	16
**130**	c	10	7	19
**131**	c	11	7	53
**132**	c	12	7	25
**133**	c	13	7	44
**134**	c	14	7	12
**135**	c	15	7	19
**136**	c	16	7	16
**137**	c	17	7	22
**138**	c	18	7	19
**139**	c	19	7	20
**140**	c	20	7	19
**141**	c	1	8	65
**142**	c	2	8	68
**143**	c	3	8	26
**144**	c	4	8	32
**145**	c	5	8	27
**146**	c	6	8	28
**147**	c	7	8	33
**148**	c	8	8	29
**149**	c	9	8	26
**150**	c	10	8	15
**151**	c	11	8	21
**152**	c	12	8	23
**153**	c	13	8	22
**154**	c	14	8	33
**155**	c	15	8	25
**156**	c	16	8	22
**157**	c	17	8	20
**158**	c	18	8	33
**159**	c	19	8	9
**160**	c	20	8	23
**161**	c	1	9	32
**162**	c	2	9	25
**163**	c	3	9	16
**164**	c	4	9	12

Obs	ANI	TEN	RP	Y
**165**	c	5	9	24
**166**	c	6	9	16
**167**	c	7	9	17
**168**	c	8	9	10
**169**	c	9	9	12
**170**	c	10	9	10
**171**	c	11	9	11
**172**	c	12	9	11
**173**	c	13	9	15
**174**	c	14	9	24
**175**	c	15	9	15
**176**	c	16	9	16
**177**	c	17	9	13
**178**	c	18	9	11
**179**	c	19	9	17
**180**	c	20	9	31
**181**	c	1	10	12
**182**	c	2	10	18
**183**	c	3	10	6
**184**	c	4	10	11
**185**	c	5	10	16
**186**	c	6	10	11
**187**	c	7	10	5
**188**	c	8	10	5
**189**	c	9	10	7
**190**	c	10	10	14
**191**	c	11	10	.
**192**	c	13	10	.
**193**	c	15	10	.
**194**	c	17	10	.
**195**	c	19	10	.
**196**	c	1	11	30
**197**	c	2	11	21
**198**	c	3	11	29
**199**	c	4	11	20
**200**	c	5	11	14
**201**	c	6	11	13
**202**	c	7	11	13
**203**	c	8	11	18
**204**	c	9	11	11
**205**	c	10	11	17

Obs	ANI	TEN	RP	Y
**206**	c	11	11	62
**207**	c	12	11	47
**208**	c	13	11	25
**209**	c	14	11	11
**210**	c	15	11	20
**211**	c	16	11	19
**212**	c	17	11	95
**213**	c	18	11	21
**214**	c	19	11	14
**215**	c	20	11	33
**216**	c	1	12	28
**217**	c	2	12	24
**218**	c	3	12	28
**219**	c	4	12	23
**220**	c	5	12	24
**221**	c	6	12	33
**222**	c	7	12	12
**223**	c	8	12	18
**224**	c	9	12	14
**225**	c	10	12	32
**226**	c	11	12	30
**227**	c	12	12	35
**228**	c	13	12	30
**229**	c	15	12	31
**230**	c	16	12	9
**231**	c	17	12	34
**232**	c	18	12	26
**233**	c	19	12	15
**234**	c	20	12	20
**235**	s	1	13	10
**236**	s	2	13	24
**237**	s	3	13	21
**238**	s	4	13	12
**239**	s	5	13	16
**240**	s	6	13	12
**241**	s	7	13	21
**242**	s	8	13	14
**243**	s	9	13	13
**244**	s	10	13	7
**245**	s	12	13	13
**246**	s	13	13	14

Obs	ANI	TEN	RP	Y
247	s	14	13	13
248	s	15	13	16
249	s	16	13	6
250	s	17	13	12
251	s	18	13	15
252	s	19	13	22
253	s	20	13	13
254	s	1	14	19
255	s	2	14	16
256	s	3	14	27
257	s	4	14	27
258	s	5	14	12
259	s	6	14	9
260	s	8	14	14
261	s	9	14	20
262	s	10	14	13
263	s	11	14	17
264	s	12	14	8
265	s	13	14	10
266	s	14	14	2
267	s	15	14	11
268	s	16	14	15
269	s	17	14	16
270	s	18	14	5
271	s	19	14	10
272	s	20	14	7
273	s	1	15	18
274	s	2	15	18
275	s	3	15	4
276	s	4	15	6
277	s	6	15	8
278	s	7	15	8
279	s	8	15	13
280	s	9	15	1
281	s	10	15	10
282	s	11	15	13
283	s	12	15	19
284	s	13	15	20
285	s	14	15	19
286	s	15	15	20
287	s	16	15	18

Obs	ANI	TEN	RP	Y
**288**	s	17	15	17
**289**	s	18	15	9
**290**	s	19	15	18
**291**	s	20	15	13
**292**	s	1	16	15
**293**	s	2	16	16
**294**	s	3	16	17
**295**	s	4	16	26
**296**	s	5	16	13
**297**	s	6	16	14
**298**	s	7	16	11
**299**	s	8	16	7
**300**	s	9	16	16
**301**	s	10	16	19
**302**	s	11	16	53
**303**	s	12	16	25
**304**	s	13	16	44
**305**	s	14	16	12
**306**	s	15	16	17
**307**	s	16	16	16
**308**	s	17	16	22
**309**	s	18	16	19
**310**	s	19	16	20
**311**	s	20	16	19
**312**	s	1	17	60
**313**	s	2	17	68
**314**	s	3	17	26
**315**	s	4	17	32
**316**	s	5	17	27
**317**	s	6	17	28
**318**	s	7	17	33
**319**	s	9	17	26
**320**	s	10	17	15
**321**	s	11	17	21
**322**	s	12	17	23
**323**	s	13	17	22
**324**	s	14	17	25
**325**	s	15	17	22
**326**	s	16	17	20
**327**	s	17	17	33
**328**	s	18	17	9

Obs	ANI	TEN	RP	Y
**329**	s	19	17	27
**330**	s	20	17	23
**331**	s	1	18	32
**332**	s	2	18	25
**333**	s	3	18	16
**334**	s	4	18	12
**335**	s	5	18	12
**336**	s	6	18	16
**337**	s	7	18	17
**338**	s	8	18	10
**339**	s	9	18	12
**340**	s	10	18	10
**341**	s	11	18	11
**342**	s	12	18	11
**343**	s	13	18	15
**344**	s	14	18	15
**345**	s	15	18	16
**346**	s	16	18	13
**347**	s	17	18	11
**348**	s	18	18	17
**349**	s	19	18	36
**350**	s	20	18	31
**351**	s	1	19	30
**352**	s	2	19	25
**353**	s	4	19	15
**354**	s	5	19	12
**355**	s	6	19	25
**356**	s	7	19	17
**357**	s	8	19	18
**358**	s	9	19	35
**359**	s	10	19	29
**360**	s	11	19	16
**361**	s	12	19	17
**362**	s	13	19	4
**363**	s	15	19	11
**364**	s	16	19	12
**365**	s	17	19	26
**366**	s	19	19	14
**367**	s	20	19	28
**368**	s	1	20	22
**369**	s	2	20	52

Obs	ANI	TEN	RP	Y
**370**	s	3	20	59
**371**	s	4	20	24
**372**	s	5	20	18
**373**	s	7	20	29
**374**	s	8	20	25
**375**	s	9	20	20
**376**	s	10	20	18
**377**	s	12	20	20
**378**	s	13	20	24
**379**	s	14	20	24
**380**	s	15	20	28
**381**	s	16	20	27
**382**	s	17	20	24
**383**	s	18	20	12
**384**	s	19	20	24
**385**	s	20	20	16
**386**	s	1	21	36
**387**	s	2	21	33
**388**	s	3	21	20
**389**	s	4	21	10
**390**	s	5	21	16
**391**	s	6	21	13
**392**	s	7	21	25
**393**	s	8	21	12
**394**	s	9	21	18
**395**	s	10	21	17
**396**	s	11	21	13
**397**	s	12	21	5
**398**	s	13	21	13
**399**	s	14	21	13
**400**	s	15	21	21
**401**	s	16	21	25
**402**	s	17	21	21
**403**	s	18	21	17
**404**	s	19	21	8
**405**	s	20	21	17
**406**	s	1	22	56
**407**	s	2	22	20
**408**	s	3	22	21
**409**	s	4	22	31
**410**	s	5	22	41

Obs	ANI	TEN	RP	Y
**411**	s	6	22	27
**412**	s	7	22	9
**413**	s	8	22	7
**414**	s	9	22	17
**415**	s	10	22	12
**416**	s	11	22	72
**417**	s	12	22	16
**418**	s	13	22	14
**419**	s	14	22	36
**420**	s	15	22	22
**421**	s	16	22	48
**422**	s	17	22	8
**423**	s	18	22	15
**424**	s	19	22	14
**425**	s	20	22	28
**426**	s	1	23	55
**427**	s	2	23	31
**428**	s	3	23	36
**429**	s	4	23	38
**430**	s	5	23	14
**431**	s	6	23	17
**432**	s	7	23	9
**433**	s	8	23	18
**434**	s	9	23	6
**435**	s	10	23	8
**436**	s	11	23	12
**437**	s	12	23	43
**438**	s	13	23	17
**439**	s	14	23	25
**440**	s	15	23	35
**441**	s	16	23	16
**442**	s	17	23	11
**443**	s	18	23	18
**444**	s	19	23	8
**445**	s	20	23	22
**446**	s	1	24	79
**447**	s	2	24	29
**448**	s	3	24	27
**449**	s	4	24	40
**450**	s	5	24	26
**451**	s	6	24	25

Obs	ANI	TEN	RP	Y
**452**	s	7	24	46
**453**	s	8	24	19
**454**	s	9	24	40
**455**	s	10	24	16
**456**	s	11	24	21
**457**	s	12	24	15
**458**	s	13	24	24
**459**	s	14	24	19
**460**	s	15	24	20
**461**	s	16	24	8
**462**	s	17	24	23
**463**	s	18	24	19
**464**	s	19	24	14
**465**	s	20	24	14

Obs	ANI	TEN	RP	Y
**1**	c	1	1	178
**2**	c	2	1	83
**3**	c	3	1	76
**4**	c	4	1	63
**5**	c	5	1	84
**6**	c	6	1	79
**7**	c	7	1	51
**8**	c	8	1	35
**9**	c	9	1	84
**10**	c	10	1	59
**11**	c	11	1	57
**12**	c	12	1	55
**13**	c	13	1	45
**14**	c	14	1	55
**15**	c	15	1	50
**16**	c	16	1	82
**17**	c	17	1	45
**18**	c	18	1	50
**19**	c	19	1	58
**20**	c	20	1	62
**21**	c	1	2	77
**22**	c	2	2	40
**23**	c	3	2	32
**24**	c	4	2	30
**25**	c	5	2	32
**26**	c	6	2	27
**27**	c	7	2	29
**28**	c	8	2	28
**29**	c	9	2	30
**30**	c	10	2	27
**31**	c	11	2	38
**32**	c	12	2	46
**33**	c	13	2	29
**34**	c	14	2	44
**35**	c	15	2	40
**36**	c	16	2	46
**37**	c	17	2	42
**38**	c	18	2	37
**39**	c	19	2	30
**40**	c	20	2	33
**41**	c	1	3	63

Obs	ANI	TEN	RP	Y
**42**	c	2	3	48
**43**	c	3	3	48
**44**	c	4	3	73
**45**	c	5	3	36
**46**	c	6	3	22
**47**	c	7	3	31
**48**	c	8	3	45
**49**	c	9	3	35
**50**	c	10	3	27
**51**	c	11	3	25
**52**	c	12	3	33
**53**	c	13	3	21
**54**	c	14	3	16
**55**	c	15	3	12
**56**	c	16	3	29
**57**	c	17	3	20
**58**	c	18	3	34
**59**	c	19	3	32
**60**	c	20	3	37
**61**	c	1	4	99
**62**	c	2	4	35
**63**	c	3	4	46
**64**	c	4	4	35
**65**	c	5	4	29
**66**	c	6	4	46
**67**	c	7	4	25
**68**	c	8	4	29
**69**	c	9	4	22
**70**	c	10	4	44
**71**	c	11	4	24
**72**	c	12	4	19
**73**	c	13	4	21
**74**	c	14	4	61
**75**	c	15	4	42
**76**	c	16	4	36
**77**	c	17	4	29
**78**	c	18	4	19
**79**	c	19	4	18
**80**	c	20	4	23
**81**	c	1	5	19
**82**	c	2	5	16

Obs	ANI	TEN	RP	Y
**83**	c	3	5	27
**84**	c	4	5	27
**85**	c	5	5	27
**86**	c	6	5	12
**87**	c	7	5	9
**88**	c	8	5	17
**89**	c	9	5	14
**90**	c	10	5	20
**91**	c	11	5	17
**92**	c	12	5	8
**93**	c	13	5	11
**94**	c	14	5	172
**95**	c	15	5	12
**96**	c	16	5	9
**97**	c	17	5	12
**98**	c	18	5	14
**99**	c	19	5	20
**100**	c	20	5	13
**101**	c	1	6	18
**102**	c	2	6	28
**103**	c	3	6	4
**104**	c	4	6	6
**105**	c	5	6	34
**106**	c	6	6	8
**107**	c	7	6	8
**108**	c	8	6	13
**109**	c	9	6	2
**110**	c	10	6	10
**111**	c	11	6	13
**112**	c	12	6	19
**113**	c	13	6	20
**114**	c	14	6	16
**115**	c	15	6	20
**116**	c	16	6	18
**117**	c	17	6	17
**118**	c	18	6	9
**119**	c	19	6	18
**120**	c	20	6	13
**121**	c	1	7	15
**122**	c	2	7	16
**123**	c	3	7	17

Obs	ANI	TEN	RP	Y
**124**	c	4	7	26
**125**	c	5	7	13
**126**	c	6	7	14
**127**	c	7	7	11
**128**	c	8	7	7
**129**	c	9	7	16
**130**	c	10	7	19
**131**	c	11	7	53
**132**	c	12	7	25
**133**	c	13	7	44
**134**	c	14	7	12
**135**	c	15	7	19
**136**	c	16	7	16
**137**	c	17	7	22
**138**	c	18	7	19
**139**	c	19	7	20
**140**	c	20	7	19
**141**	c	1	8	65
**142**	c	2	8	68
**143**	c	3	8	26
**144**	c	4	8	32
**145**	c	5	8	27
**146**	c	6	8	28
**147**	c	7	8	33
**148**	c	8	8	29
**149**	c	9	8	26
**150**	c	10	8	15
**151**	c	11	8	21
**152**	c	12	8	23
**153**	c	13	8	22
**154**	c	14	8	33
**155**	c	15	8	25
**156**	c	16	8	22
**157**	c	17	8	20
**158**	c	18	8	33
**159**	c	19	8	9
**160**	c	20	8	23
**161**	c	1	9	32
**162**	c	2	9	25
**163**	c	3	9	16
**164**	c	4	9	12

Obs	ANI	TEN	RP	Y
**165**	c	5	9	24
**166**	c	6	9	16
**167**	c	7	9	17
**168**	c	8	9	10
**169**	c	9	9	12
**170**	c	10	9	10
**171**	c	11	9	11
**172**	c	12	9	11
**173**	c	13	9	15
**174**	c	14	9	24
**175**	c	15	9	15
**176**	c	16	9	16
**177**	c	17	9	13
**178**	c	18	9	11
**179**	c	19	9	17
**180**	c	20	9	31
**181**	c	1	10	12
**182**	c	2	10	18
**183**	c	3	10	6
**184**	c	4	10	11
**185**	c	5	10	16
**186**	c	6	10	11
**187**	c	7	10	5
**188**	c	8	10	5
**189**	c	9	10	7
**190**	c	10	10	14
**191**	c	11	10	.
**192**	c	13	10	.
**193**	c	15	10	.
**194**	c	17	10	.
**195**	c	19	10	.
**196**	c	1	11	30
**197**	c	2	11	21
**198**	c	3	11	29
**199**	c	4	11	20
**200**	c	5	11	14
**201**	c	6	11	13
**202**	c	7	11	13
**203**	c	8	11	18
**204**	c	9	11	11
**205**	c	10	11	17

Obs	ANI	TEN	RP	Y
206	c	11	11	62
207	c	12	11	47
208	c	13	11	25
209	c	14	11	11
210	c	15	11	20
211	c	16	11	19
212	c	17	11	95
213	c	18	11	21
214	c	19	11	14
215	c	20	11	33
216	c	1	12	28
217	c	2	12	24
218	c	3	12	28
219	c	4	12	23
220	c	5	12	24
221	c	6	12	33
222	c	7	12	12
223	c	8	12	18
224	c	9	12	14
225	c	10	12	32
226	c	11	12	30
227	c	12	12	35
228	c	13	12	30
229	c	15	12	31
230	c	16	12	9
231	c	17	12	34
232	c	18	12	26
233	c	19	12	15
234	c	20	12	20
235	s	1	13	10
236	s	2	13	24
237	s	3	13	21
238	s	4	13	12
239	s	5	13	16
240	s	6	13	12
241	s	7	13	21
242	s	8	13	14
243	s	9	13	13
244	s	10	13	7
245	s	12	13	13
246	s	13	13	14

Obs	ANI	TEN	RP	Y
**247**	s	14	13	13
**248**	s	15	13	16
**249**	s	16	13	6
**250**	s	17	13	12
**251**	s	18	13	15
**252**	s	19	13	22
**253**	s	20	13	13
**254**	s	1	14	19
**255**	s	2	14	16
**256**	s	3	14	27
**257**	s	4	14	27
**258**	s	5	14	12
**259**	s	6	14	9
**260**	s	8	14	14
**261**	s	9	14	20
**262**	s	10	14	13
**263**	s	11	14	17
**264**	s	12	14	8
**265**	s	13	14	10
**266**	s	14	14	2
**267**	s	15	14	11
**268**	s	16	14	15
**269**	s	17	14	16
**270**	s	18	14	5
**271**	s	19	14	10
**272**	s	20	14	7
**273**	s	1	15	18
**274**	s	2	15	18
**275**	s	3	15	4
**276**	s	4	15	6
**277**	s	6	15	8
**278**	s	7	15	8
**279**	s	8	15	13
**280**	s	9	15	1
**281**	s	10	15	10
**282**	s	11	15	13
**283**	s	12	15	19
**284**	s	13	15	20
**285**	s	14	15	19
**286**	s	15	15	20
**287**	s	16	15	18

Obs	ANI	TEN	RP	Y
**288**	s	17	15	17
**289**	s	18	15	9
**290**	s	19	15	18
**291**	s	20	15	13
**292**	s	1	16	15
**293**	s	2	16	16
**294**	s	3	16	17
**295**	s	4	16	26
**296**	s	5	16	13
**297**	s	6	16	14
**298**	s	7	16	11
**299**	s	8	16	7
**300**	s	9	16	16
**301**	s	10	16	19
**302**	s	11	16	53
**303**	s	12	16	25
**304**	s	13	16	44
**305**	s	14	16	12
**306**	s	15	16	17
**307**	s	16	16	16
**308**	s	17	16	22
**309**	s	18	16	19
**310**	s	19	16	20
**311**	s	20	16	19
**312**	s	1	17	60
**313**	s	2	17	68
**314**	s	3	17	26
**315**	s	4	17	32
**316**	s	5	17	27
**317**	s	6	17	28
**318**	s	7	17	33
**319**	s	9	17	26
**320**	s	10	17	15
**321**	s	11	17	21
**322**	s	12	17	23
**323**	s	13	17	22
**324**	s	14	17	25
**325**	s	15	17	22
**326**	s	16	17	20
**327**	s	17	17	33
**328**	s	18	17	9

Obs	ANI	TEN	RP	Y
329	s	19	17	27
330	s	20	17	23
331	s	1	18	32
332	s	2	18	25
333	s	3	18	16
334	s	4	18	12
335	s	5	18	12
336	s	6	18	16
337	s	7	18	17
338	s	8	18	10
339	s	9	18	12
340	s	10	18	10
341	s	11	18	11
342	s	12	18	11
343	s	13	18	15
344	s	14	18	15
345	s	15	18	16
346	s	16	18	13
347	s	17	18	11
348	s	18	18	17
349	s	19	18	36
350	s	20	18	31
351	s	1	19	30
352	s	2	19	25
353	s	4	19	15
354	s	5	19	12
355	s	6	19	25
356	s	7	19	17
357	s	8	19	18
358	s	9	19	35
359	s	10	19	29
360	s	11	19	16
361	s	12	19	17
362	s	13	19	4
363	s	15	19	11
364	s	16	19	12
365	s	17	19	26
366	s	19	19	14
367	s	20	19	28
368	s	1	20	22
369	s	2	20	52

Obs	ANI	TEN	RP	Y
370	s	3	20	59
371	s	4	20	24
372	s	5	20	18
373	s	7	20	29
374	s	8	20	25
375	s	9	20	20
376	s	10	20	18
377	s	12	20	20
378	s	13	20	24
379	s	14	20	24
380	s	15	20	28
381	s	16	20	27
382	s	17	20	24
383	s	18	20	12
384	s	19	20	24
385	s	20	20	16
386	s	1	21	36
387	s	2	21	33
388	s	3	21	20
389	s	4	21	10
390	s	5	21	16
391	s	6	21	13
392	s	7	21	25
393	s	8	21	12
394	s	9	21	18
395	s	10	21	17
396	s	11	21	13
397	s	12	21	5
398	s	13	21	13
399	s	14	21	13
400	s	15	21	21
401	s	16	21	25
402	s	17	21	21
403	s	18	21	17
404	s	19	21	8
405	s	20	21	17
406	s	1	22	56
407	s	2	22	20
408	s	3	22	21
409	s	4	22	31
410	s	5	22	41

Obs	ANI	TEN	RP	Y
411	s	6	22	27
412	s	7	22	9
413	s	8	22	7
414	s	9	22	17
415	s	10	22	12
416	s	11	22	72
417	s	12	22	16
418	s	13	22	14
419	s	14	22	36
420	s	15	22	22
421	s	16	22	48
422	s	17	22	8
423	s	18	22	15
424	s	19	22	14
425	s	20	22	28
426	s	1	23	55
427	s	2	23	31
428	s	3	23	36
429	s	4	23	38
430	s	5	23	14
431	s	6	23	17
432	s	7	23	9
433	s	8	23	18
434	s	9	23	6
435	s	10	23	8
436	s	11	23	12
437	s	12	23	43
438	s	13	23	17
439	s	14	23	25
440	s	15	23	35
441	s	16	23	16
442	s	17	23	11
443	s	18	23	18
444	s	19	23	8
445	s	20	23	22
446	s	1	24	79
447	s	2	24	29
448	s	3	24	27
449	s	4	24	40
450	s	5	24	26
451	s	6	24	25

Obs	ANI	TEN	RP	Y
**452**	s	7	24	46
**453**	s	8	24	19
**454**	s	9	24	40
**455**	s	10	24	16
**456**	s	11	24	21
**457**	s	12	24	15
**458**	s	13	24	24
**459**	s	14	24	19
**460**	s	15	24	20
**461**	s	16	24	8
**462**	s	17	24	23
**463**	s	18	24	19
**464**	s	19	24	14
**465**	s	20	24	14

Obs	ANI	TEN	RP	Y
**1**	c	1	1	178
**2**	c	2	1	83
**3**	c	3	1	76
**4**	c	4	1	63
**5**	c	5	1	84
**6**	c	6	1	79
**7**	c	7	1	51
**8**	c	8	1	35
**9**	c	9	1	84
**10**	c	10	1	59
**11**	c	11	1	57
**12**	c	12	1	55
**13**	c	13	1	45
**14**	c	14	1	55
**15**	c	15	1	50
**16**	c	16	1	82
**17**	c	17	1	45
**18**	c	18	1	50
**19**	c	19	1	58
**20**	c	20	1	62
**21**	c	1	2	77
**22**	c	2	2	40
**23**	c	3	2	32
**24**	c	4	2	30
**25**	c	5	2	32
**26**	c	6	2	27
**27**	c	7	2	29
**28**	c	8	2	28
**29**	c	9	2	30
**30**	c	10	2	27
**31**	c	11	2	38
**32**	c	12	2	46
**33**	c	13	2	29
**34**	c	14	2	44
**35**	c	15	2	40
**36**	c	16	2	46
**37**	c	17	2	42
**38**	c	18	2	37
**39**	c	19	2	30
**40**	c	20	2	33
**41**	c	1	3	63

Obs	ANI	TEN	RP	Y
**42**	c	2	3	48
**43**	c	3	3	48
**44**	c	4	3	73
**45**	c	5	3	36
**46**	c	6	3	22
**47**	c	7	3	31
**48**	c	8	3	45
**49**	c	9	3	35
**50**	c	10	3	27
**51**	c	11	3	25
**52**	c	12	3	33
**53**	c	13	3	21
**54**	c	14	3	16
**55**	c	15	3	12
**56**	c	16	3	29
**57**	c	17	3	20
**58**	c	18	3	34
**59**	c	19	3	32
**60**	c	20	3	37
**61**	c	1	4	99
**62**	c	2	4	35
**63**	c	3	4	46
**64**	c	4	4	35
**65**	c	5	4	29
**66**	c	6	4	46
**67**	c	7	4	25
**68**	c	8	4	29
**69**	c	9	4	22
**70**	c	10	4	44
**71**	c	11	4	24
**72**	c	12	4	19
**73**	c	13	4	21
**74**	c	14	4	61
**75**	c	15	4	42
**76**	c	16	4	36
**77**	c	17	4	29
**78**	c	18	4	19
**79**	c	19	4	18
**80**	c	20	4	23
**81**	c	1	5	19
**82**	c	2	5	16

Obs	ANI	TEN	RP	Y
**83**	c	3	5	27
**84**	c	4	5	27
**85**	c	5	5	27
**86**	c	6	5	12
**87**	c	7	5	9
**88**	c	8	5	17
**89**	c	9	5	14
**90**	c	10	5	20
**91**	c	11	5	17
**92**	c	12	5	8
**93**	c	13	5	11
**94**	c	14	5	172
**95**	c	15	5	12
**96**	c	16	5	9
**97**	c	17	5	12
**98**	c	18	5	14
**99**	c	19	5	20
**100**	c	20	5	13
**101**	c	1	6	18
**102**	c	2	6	28
**103**	c	3	6	4
**104**	c	4	6	6
**105**	c	5	6	34
**106**	c	6	6	8
**107**	c	7	6	8
**108**	c	8	6	13
**109**	c	9	6	2
**110**	c	10	6	10
**111**	c	11	6	13
**112**	c	12	6	19
**113**	c	13	6	20
**114**	c	14	6	16
**115**	c	15	6	20
**116**	c	16	6	18
**117**	c	17	6	17
**118**	c	18	6	9
**119**	c	19	6	18
**120**	c	20	6	13
**121**	c	1	7	15
**122**	c	2	7	16
**123**	c	3	7	17

Obs	ANI	TEN	RP	Y
**124**	c	4	7	26
**125**	c	5	7	13
**126**	c	6	7	14
**127**	c	7	7	11
**128**	c	8	7	7
**129**	c	9	7	16
**130**	c	10	7	19
**131**	c	11	7	53
**132**	c	12	7	25
**133**	c	13	7	44
**134**	c	14	7	12
**135**	c	15	7	19
**136**	c	16	7	16
**137**	c	17	7	22
**138**	c	18	7	19
**139**	c	19	7	20
**140**	c	20	7	19
**141**	c	1	8	65
**142**	c	2	8	68
**143**	c	3	8	26
**144**	c	4	8	32
**145**	c	5	8	27
**146**	c	6	8	28
**147**	c	7	8	33
**148**	c	8	8	29
**149**	c	9	8	26
**150**	c	10	8	15
**151**	c	11	8	21
**152**	c	12	8	23
**153**	c	13	8	22
**154**	c	14	8	33
**155**	c	15	8	25
**156**	c	16	8	22
**157**	c	17	8	20
**158**	c	18	8	33
**159**	c	19	8	9
**160**	c	20	8	23
**161**	c	1	9	32
**162**	c	2	9	25
**163**	c	3	9	16
**164**	c	4	9	12

Obs	ANI	TEN	RP	Y
165	c	5	9	24
166	c	6	9	16
167	c	7	9	17
168	c	8	9	10
169	c	9	9	12
170	c	10	9	10
171	c	11	9	11
172	c	12	9	11
173	c	13	9	15
174	c	14	9	24
175	c	15	9	15
176	c	16	9	16
177	c	17	9	13
178	c	18	9	11
179	c	19	9	17
180	c	20	9	31
181	c	1	10	12
182	c	2	10	18
183	c	3	10	6
184	c	4	10	11
185	c	5	10	16
186	c	6	10	11
187	c	7	10	5
188	c	8	10	5
189	c	9	10	7
190	c	10	10	14
191	c	11	10	.
192	c	13	10	.
193	c	15	10	.
194	c	17	10	.
195	c	19	10	.
196	c	1	11	30
197	c	2	11	21
198	c	3	11	29
199	c	4	11	20
200	c	5	11	14
201	c	6	11	13
202	c	7	11	13
203	c	8	11	18
204	c	9	11	11
205	c	10	11	17

Obs	ANI	TEN	RP	Y
**206**	c	11	11	62
**207**	c	12	11	47
**208**	c	13	11	25
**209**	c	14	11	11
**210**	c	15	11	20
**211**	c	16	11	19
**212**	c	17	11	95
**213**	c	18	11	21
**214**	c	19	11	14
**215**	c	20	11	33
**216**	c	1	12	28
**217**	c	2	12	24
**218**	c	3	12	28
**219**	c	4	12	23
**220**	c	5	12	24
**221**	c	6	12	33
**222**	c	7	12	12
**223**	c	8	12	18
**224**	c	9	12	14
**225**	c	10	12	32
**226**	c	11	12	30
**227**	c	12	12	35
**228**	c	13	12	30
**229**	c	15	12	31
**230**	c	16	12	9
**231**	c	17	12	34
**232**	c	18	12	26
**233**	c	19	12	15
**234**	c	20	12	20
**235**	s	1	13	10
**236**	s	2	13	24
**237**	s	3	13	21
**238**	s	4	13	12
**239**	s	5	13	16
**240**	s	6	13	12
**241**	s	7	13	21
**242**	s	8	13	14
**243**	s	9	13	13
**244**	s	10	13	7
**245**	s	12	13	13
**246**	s	13	13	14

Obs	ANI	TEN	RP	Y
**247**	s	14	13	13
**248**	s	15	13	16
**249**	s	16	13	6
**250**	s	17	13	12
**251**	s	18	13	15
**252**	s	19	13	22
**253**	s	20	13	13
**254**	s	1	14	19
**255**	s	2	14	16
**256**	s	3	14	27
**257**	s	4	14	27
**258**	s	5	14	12
**259**	s	6	14	9
**260**	s	8	14	14
**261**	s	9	14	20
**262**	s	10	14	13
**263**	s	11	14	17
**264**	s	12	14	8
**265**	s	13	14	10
**266**	s	14	14	2
**267**	s	15	14	11
**268**	s	16	14	15
**269**	s	17	14	16
**270**	s	18	14	5
**271**	s	19	14	10
**272**	s	20	14	7
**273**	s	1	15	18
**274**	s	2	15	18
**275**	s	3	15	4
**276**	s	4	15	6
**277**	s	6	15	8
**278**	s	7	15	8
**279**	s	8	15	13
**280**	s	9	15	1
**281**	s	10	15	10
**282**	s	11	15	13
**283**	s	12	15	19
**284**	s	13	15	20
**285**	s	14	15	19
**286**	s	15	15	20
**287**	s	16	15	18

Obs	ANI	TEN	RP	Y
**288**	s	17	15	17
**289**	s	18	15	9
**290**	s	19	15	18
**291**	s	20	15	13
**292**	s	1	16	15
**293**	s	2	16	16
**294**	s	3	16	17
**295**	s	4	16	26
**296**	s	5	16	13
**297**	s	6	16	14
**298**	s	7	16	11
**299**	s	8	16	7
**300**	s	9	16	16
**301**	s	10	16	19
**302**	s	11	16	53
**303**	s	12	16	25
**304**	s	13	16	44
**305**	s	14	16	12
**306**	s	15	16	17
**307**	s	16	16	16
**308**	s	17	16	22
**309**	s	18	16	19
**310**	s	19	16	20
**311**	s	20	16	19
**312**	s	1	17	60
**313**	s	2	17	68
**314**	s	3	17	26
**315**	s	4	17	32
**316**	s	5	17	27
**317**	s	6	17	28
**318**	s	7	17	33
**319**	s	9	17	26
**320**	s	10	17	15
**321**	s	11	17	21
**322**	s	12	17	23
**323**	s	13	17	22
**324**	s	14	17	25
**325**	s	15	17	22
**326**	s	16	17	20
**327**	s	17	17	33
**328**	s	18	17	9

Obs	ANI	TEN	RP	Y
**329**	s	19	17	27
**330**	s	20	17	23
**331**	s	1	18	32
**332**	s	2	18	25
**333**	s	3	18	16
**334**	s	4	18	12
**335**	s	5	18	12
**336**	s	6	18	16
**337**	s	7	18	17
**338**	s	8	18	10
**339**	s	9	18	12
**340**	s	10	18	10
**341**	s	11	18	11
**342**	s	12	18	11
**343**	s	13	18	15
**344**	s	14	18	15
**345**	s	15	18	16
**346**	s	16	18	13
**347**	s	17	18	11
**348**	s	18	18	17
**349**	s	19	18	36
**350**	s	20	18	31
**351**	s	1	19	30
**352**	s	2	19	25
**353**	s	4	19	15
**354**	s	5	19	12
**355**	s	6	19	25
**356**	s	7	19	17
**357**	s	8	19	18
**358**	s	9	19	35
**359**	s	10	19	29
**360**	s	11	19	16
**361**	s	12	19	17
**362**	s	13	19	4
**363**	s	15	19	11
**364**	s	16	19	12
**365**	s	17	19	26
**366**	s	19	19	14
**367**	s	20	19	28
**368**	s	1	20	22
**369**	s	2	20	52

Obs	ANI	TEN	RP	Y
370	s	3	20	59
371	s	4	20	24
372	s	5	20	18
373	s	7	20	29
374	s	8	20	25
375	s	9	20	20
376	s	10	20	18
377	s	12	20	20
378	s	13	20	24
379	s	14	20	24
380	s	15	20	28
381	s	16	20	27
382	s	17	20	24
383	s	18	20	12
384	s	19	20	24
385	s	20	20	16
386	s	1	21	36
387	s	2	21	33
388	s	3	21	20
389	s	4	21	10
390	s	5	21	16
391	s	6	21	13
392	s	7	21	25
393	s	8	21	12
394	s	9	21	18
395	s	10	21	17
396	s	11	21	13
397	s	12	21	5
398	s	13	21	13
399	s	14	21	13
400	s	15	21	21
401	s	16	21	25
402	s	17	21	21
403	s	18	21	17
404	s	19	21	8
405	s	20	21	17
406	s	1	22	56
407	s	2	22	20
408	s	3	22	21
409	s	4	22	31
410	s	5	22	41

Obs	ANI	TEN	RP	Y
**411**	s	6	22	27
**412**	s	7	22	9
**413**	s	8	22	7
**414**	s	9	22	17
**415**	s	10	22	12
**416**	s	11	22	72
**417**	s	12	22	16
**418**	s	13	22	14
**419**	s	14	22	36
**420**	s	15	22	22
**421**	s	16	22	48
**422**	s	17	22	8
**423**	s	18	22	15
**424**	s	19	22	14
**425**	s	20	22	28
**426**	s	1	23	55
**427**	s	2	23	31
**428**	s	3	23	36
**429**	s	4	23	38
**430**	s	5	23	14
**431**	s	6	23	17
**432**	s	7	23	9
**433**	s	8	23	18
**434**	s	9	23	6
**435**	s	10	23	8
**436**	s	11	23	12
**437**	s	12	23	43
**438**	s	13	23	17
**439**	s	14	23	25
**440**	s	15	23	35
**441**	s	16	23	16
**442**	s	17	23	11
**443**	s	18	23	18
**444**	s	19	23	8
**445**	s	20	23	22
**446**	s	1	24	79
**447**	s	2	24	29
**448**	s	3	24	27
**449**	s	4	24	40
**450**	s	5	24	26
**451**	s	6	24	25

Obs	ANI	TEN	RP	Y
**452**	s	7	24	46
**453**	s	8	24	19
**454**	s	9	24	40
**455**	s	10	24	16
**456**	s	11	24	21
**457**	s	12	24	15
**458**	s	13	24	24
**459**	s	14	24	19
**460**	s	15	24	20
**461**	s	16	24	8
**462**	s	17	24	23
**463**	s	18	24	19
**464**	s	19	24	14
**465**	s	20	24	14

Obs	ANI	TEN	RP	Y
**1**	c	1	1	178
**2**	c	2	1	83
**3**	c	3	1	76
**4**	c	4	1	63
**5**	c	5	1	84
**6**	c	6	1	79
**7**	c	7	1	51
**8**	c	8	1	35
**9**	c	9	1	84
**10**	c	10	1	59
**11**	c	11	1	57
**12**	c	12	1	55
**13**	c	13	1	45
**14**	c	14	1	55
**15**	c	15	1	50
**16**	c	16	1	82
**17**	c	17	1	45
**18**	c	18	1	50
**19**	c	19	1	58
**20**	c	20	1	62
**21**	c	1	2	77
**22**	c	2	2	40
**23**	c	3	2	32
**24**	c	4	2	30
**25**	c	5	2	32
**26**	c	6	2	27
**27**	c	7	2	29
**28**	c	8	2	28
**29**	c	9	2	30
**30**	c	10	2	27
**31**	c	11	2	38
**32**	c	12	2	46
**33**	c	13	2	29
**34**	c	14	2	44
**35**	c	15	2	40
**36**	c	16	2	46
**37**	c	17	2	42
**38**	c	18	2	37
**39**	c	19	2	30
**40**	c	20	2	33
**41**	c	1	3	63

Obs	ANI	TEN	RP	Y
**42**	c	2	3	48
**43**	c	3	3	48
**44**	c	4	3	73
**45**	c	5	3	36
**46**	c	6	3	22
**47**	c	7	3	31
**48**	c	8	3	45
**49**	c	9	3	35
**50**	c	10	3	27
**51**	c	11	3	25
**52**	c	12	3	33
**53**	c	13	3	21
**54**	c	14	3	16
**55**	c	15	3	12
**56**	c	16	3	29
**57**	c	17	3	20
**58**	c	18	3	34
**59**	c	19	3	32
**60**	c	20	3	37
**61**	c	1	4	99
**62**	c	2	4	35
**63**	c	3	4	46
**64**	c	4	4	35
**65**	c	5	4	29
**66**	c	6	4	46
**67**	c	7	4	25
**68**	c	8	4	29
**69**	c	9	4	22
**70**	c	10	4	44
**71**	c	11	4	24
**72**	c	12	4	19
**73**	c	13	4	21
**74**	c	14	4	61
**75**	c	15	4	42
**76**	c	16	4	36
**77**	c	17	4	29
**78**	c	18	4	19
**79**	c	19	4	18
**80**	c	20	4	23
**81**	c	1	5	19
**82**	c	2	5	16

Obs	ANI	TEN	RP	Y
**83**	c	3	5	27
**84**	c	4	5	27
**85**	c	5	5	27
**86**	c	6	5	12
**87**	c	7	5	9
**88**	c	8	5	17
**89**	c	9	5	14
**90**	c	10	5	20
**91**	c	11	5	17
**92**	c	12	5	8
**93**	c	13	5	11
**94**	c	14	5	172
**95**	c	15	5	12
**96**	c	16	5	9
**97**	c	17	5	12
**98**	c	18	5	14
**99**	c	19	5	20
**100**	c	20	5	13
**101**	c	1	6	18
**102**	c	2	6	28
**103**	c	3	6	4
**104**	c	4	6	6
**105**	c	5	6	34
**106**	c	6	6	8
**107**	c	7	6	8
**108**	c	8	6	13
**109**	c	9	6	2
**110**	c	10	6	10
**111**	c	11	6	13
**112**	c	12	6	19
**113**	c	13	6	20
**114**	c	14	6	16
**115**	c	15	6	20
**116**	c	16	6	18
**117**	c	17	6	17
**118**	c	18	6	9
**119**	c	19	6	18
**120**	c	20	6	13
**121**	c	1	7	15
**122**	c	2	7	16
**123**	c	3	7	17

Obs	ANI	TEN	RP	Y
124	c	4	7	26
125	c	5	7	13
126	c	6	7	14
127	c	7	7	11
128	c	8	7	7
129	c	9	7	16
130	c	10	7	19
131	c	11	7	53
132	c	12	7	25
133	c	13	7	44
134	c	14	7	12
135	c	15	7	19
136	c	16	7	16
137	c	17	7	22
138	c	18	7	19
139	c	19	7	20
140	c	20	7	19
141	c	1	8	65
142	c	2	8	68
143	c	3	8	26
144	c	4	8	32
145	c	5	8	27
146	c	6	8	28
147	c	7	8	33
148	c	8	8	29
149	c	9	8	26
150	c	10	8	15
151	c	11	8	21
152	c	12	8	23
153	c	13	8	22
154	c	14	8	33
155	c	15	8	25
156	c	16	8	22
157	c	17	8	20
158	c	18	8	33
159	c	19	8	9
160	c	20	8	23
161	c	1	9	32
162	c	2	9	25
163	c	3	9	16
164	c	4	9	12

Obs	ANI	TEN	RP	Y
165	c	5	9	24
166	c	6	9	16
167	c	7	9	17
168	c	8	9	10
169	c	9	9	12
170	c	10	9	10
171	c	11	9	11
172	c	12	9	11
173	c	13	9	15
174	c	14	9	24
175	c	15	9	15
176	c	16	9	16
177	c	17	9	13
178	c	18	9	11
179	c	19	9	17
180	c	20	9	31
181	c	1	10	12
182	c	2	10	18
183	c	3	10	6
184	c	4	10	11
185	c	5	10	16
186	c	6	10	11
187	c	7	10	5
188	c	8	10	5
189	c	9	10	7
190	c	10	10	14
191	c	11	10	.
192	c	13	10	.
193	c	15	10	.
194	c	17	10	.
195	c	19	10	.
196	c	1	11	30
197	c	2	11	21
198	c	3	11	29
199	c	4	11	20
200	c	5	11	14
201	c	6	11	13
202	c	7	11	13
203	c	8	11	18
204	c	9	11	11
205	c	10	11	17

Obs	ANI	TEN	RP	Y
**206**	c	11	11	62
**207**	c	12	11	47
**208**	c	13	11	25
**209**	c	14	11	11
**210**	c	15	11	20
**211**	c	16	11	19
**212**	c	17	11	95
**213**	c	18	11	21
**214**	c	19	11	14
**215**	c	20	11	33
**216**	c	1	12	28
**217**	c	2	12	24
**218**	c	3	12	28
**219**	c	4	12	23
**220**	c	5	12	24
**221**	c	6	12	33
**222**	c	7	12	12
**223**	c	8	12	18
**224**	c	9	12	14
**225**	c	10	12	32
**226**	c	11	12	30
**227**	c	12	12	35
**228**	c	13	12	30
**229**	c	15	12	31
**230**	c	16	12	9
**231**	c	17	12	34
**232**	c	18	12	26
**233**	c	19	12	15
**234**	c	20	12	20
**235**	s	1	13	10
**236**	s	2	13	24
**237**	s	3	13	21
**238**	s	4	13	12
**239**	s	5	13	16
**240**	s	6	13	12
**241**	s	7	13	21
**242**	s	8	13	14
**243**	s	9	13	13
**244**	s	10	13	7
**245**	s	12	13	13
**246**	s	13	13	14

Obs	ANI	TEN	RP	Y
**247**	s	14	13	13
**248**	s	15	13	16
**249**	s	16	13	6
**250**	s	17	13	12
**251**	s	18	13	15
**252**	s	19	13	22
**253**	s	20	13	13
**254**	s	1	14	19
**255**	s	2	14	16
**256**	s	3	14	27
**257**	s	4	14	27
**258**	s	5	14	12
**259**	s	6	14	9
**260**	s	8	14	14
**261**	s	9	14	20
**262**	s	10	14	13
**263**	s	11	14	17
**264**	s	12	14	8
**265**	s	13	14	10
**266**	s	14	14	2
**267**	s	15	14	11
**268**	s	16	14	15
**269**	s	17	14	16
**270**	s	18	14	5
**271**	s	19	14	10
**272**	s	20	14	7
**273**	s	1	15	18
**274**	s	2	15	18
**275**	s	3	15	4
**276**	s	4	15	6
**277**	s	6	15	8
**278**	s	7	15	8
**279**	s	8	15	13
**280**	s	9	15	1
**281**	s	10	15	10
**282**	s	11	15	13
**283**	s	12	15	19
**284**	s	13	15	20
**285**	s	14	15	19
**286**	s	15	15	20
**287**	s	16	15	18

Obs	ANI	TEN	RP	Y
**288**	s	17	15	17
**289**	s	18	15	9
**290**	s	19	15	18
**291**	s	20	15	13
**292**	s	1	16	15
**293**	s	2	16	16
**294**	s	3	16	17
**295**	s	4	16	26
**296**	s	5	16	13
**297**	s	6	16	14
**298**	s	7	16	11
**299**	s	8	16	7
**300**	s	9	16	16
**301**	s	10	16	19
**302**	s	11	16	53
**303**	s	12	16	25
**304**	s	13	16	44
**305**	s	14	16	12
**306**	s	15	16	17
**307**	s	16	16	16
**308**	s	17	16	22
**309**	s	18	16	19
**310**	s	19	16	20
**311**	s	20	16	19
**312**	s	1	17	60
**313**	s	2	17	68
**314**	s	3	17	26
**315**	s	4	17	32
**316**	s	5	17	27
**317**	s	6	17	28
**318**	s	7	17	33
**319**	s	9	17	26
**320**	s	10	17	15
**321**	s	11	17	21
**322**	s	12	17	23
**323**	s	13	17	22
**324**	s	14	17	25
**325**	s	15	17	22
**326**	s	16	17	20
**327**	s	17	17	33
**328**	s	18	17	9

Obs	ANI	TEN	RP	Y
329	s	19	17	27
330	s	20	17	23
331	s	1	18	32
332	s	2	18	25
333	s	3	18	16
334	s	4	18	12
335	s	5	18	12
336	s	6	18	16
337	s	7	18	17
338	s	8	18	10
339	s	9	18	12
340	s	10	18	10
341	s	11	18	11
342	s	12	18	11
343	s	13	18	15
344	s	14	18	15
345	s	15	18	16
346	s	16	18	13
347	s	17	18	11
348	s	18	18	17
349	s	19	18	36
350	s	20	18	31
351	s	1	19	30
352	s	2	19	25
353	s	4	19	15
354	s	5	19	12
355	s	6	19	25
356	s	7	19	17
357	s	8	19	18
358	s	9	19	35
359	s	10	19	29
360	s	11	19	16
361	s	12	19	17
362	s	13	19	4
363	s	15	19	11
364	s	16	19	12
365	s	17	19	26
366	s	19	19	14
367	s	20	19	28
368	s	1	20	22
369	s	2	20	52

Obs	ANI	TEN	RP	Y
**370**	s	3	20	59
**371**	s	4	20	24
**372**	s	5	20	18
**373**	s	7	20	29
**374**	s	8	20	25
**375**	s	9	20	20
**376**	s	10	20	18
**377**	s	12	20	20
**378**	s	13	20	24
**379**	s	14	20	24
**380**	s	15	20	28
**381**	s	16	20	27
**382**	s	17	20	24
**383**	s	18	20	12
**384**	s	19	20	24
**385**	s	20	20	16
**386**	s	1	21	36
**387**	s	2	21	33
**388**	s	3	21	20
**389**	s	4	21	10
**390**	s	5	21	16
**391**	s	6	21	13
**392**	s	7	21	25
**393**	s	8	21	12
**394**	s	9	21	18
**395**	s	10	21	17
**396**	s	11	21	13
**397**	s	12	21	5
**398**	s	13	21	13
**399**	s	14	21	13
**400**	s	15	21	21
**401**	s	16	21	25
**402**	s	17	21	21
**403**	s	18	21	17
**404**	s	19	21	8
**405**	s	20	21	17
**406**	s	1	22	56
**407**	s	2	22	20
**408**	s	3	22	21
**409**	s	4	22	31
**410**	s	5	22	41

Obs	ANI	TEN	RP	Y
**411**	s	6	22	27
**412**	s	7	22	9
**413**	s	8	22	7
**414**	s	9	22	17
**415**	s	10	22	12
**416**	s	11	22	72
**417**	s	12	22	16
**418**	s	13	22	14
**419**	s	14	22	36
**420**	s	15	22	22
**421**	s	16	22	48
**422**	s	17	22	8
**423**	s	18	22	15
**424**	s	19	22	14
**425**	s	20	22	28
**426**	s	1	23	55
**427**	s	2	23	31
**428**	s	3	23	36
**429**	s	4	23	38
**430**	s	5	23	14
**431**	s	6	23	17
**432**	s	7	23	9
**433**	s	8	23	18
**434**	s	9	23	6
**435**	s	10	23	8
**436**	s	11	23	12
**437**	s	12	23	43
**438**	s	13	23	17
**439**	s	14	23	25
**440**	s	15	23	35
**441**	s	16	23	16
**442**	s	17	23	11
**443**	s	18	23	18
**444**	s	19	23	8
**445**	s	20	23	22
**446**	s	1	24	79
**447**	s	2	24	29
**448**	s	3	24	27
**449**	s	4	24	40
**450**	s	5	24	26
**451**	s	6	24	25

Obs	ANI	TEN	RP	Y
**452**	s	7	24	46
**453**	s	8	24	19
**454**	s	9	24	40
**455**	s	10	24	16
**456**	s	11	24	21
**457**	s	12	24	15
**458**	s	13	24	24
**459**	s	14	24	19
**460**	s	15	24	20
**461**	s	16	24	8
**462**	s	17	24	23
**463**	s	18	24	19
**464**	s	19	24	14
**465**	s	20	24	14

The Mixed Procedure

Model Information	
**Data Set**	WORK.AU
**Dependent Variable**	Y
**Covariance Structure**	Ante-dependence
**Subject Effect**	RP(TEN)
**Estimation Method**	REML
**Residual Variance Method**	None
**Fixed Effects SE Method**	Model-Based
**Degrees of Freedom Method**	Between-Within

Class Level Information		
**Class**	**Levels**	**Values**
**RP**	24	1 2 3 4 5 6 7 8 9 10 11 12 13 14 15 16 17 18 19 20 21 22 23 24
**TEN**	20	1 10 11 12 13 14 15 16 17 18 19 2 20 3 4 5 6 7 8 9
**ANI**	2	c s

Dimensions	
**Covariance Parameters**	3
**Columns in X**	63
**Columns in Z**	0
**Subjects**	465
**Max Obs per Subject**	1

Number of Observations	
**Number of Observations Read**	465
**Number of Observations Used**	460
**Number of Observations Not Used**	5

Iteration History			
**Iteration**	**Evaluations**	**-2 Res Log Like**	**Criterion**
**0**	1	3700.31309402	
**1**	2	3634.52174871	0.00005939
**2**	1	3615.73654895	0.00002179
**3**	1	3610.09798892	0.00000080
**4**	1	3609.56313585	0.00000001

Convergence criteria met but final Hessian is not positive definite.

The Mixed Procedure

Estimated R Matrix for RP(TEN) 1 1	
Row	Col1
1	492.31

Estimated R Correlation Matrix for RP(TEN) 1 1	
Row	Col1
1	1.0000

Covariance Parameter Estimates		
Cov Parm	Subject	Estimate
Var(1)	RP(TEN)	492.31
Var(2)	RP(TEN)	127.18
Rho(1)	RP(TEN)	0

Fit Statistics	
-2 Res Log Likelihood	3609.6
AIC (Smaller is Better)	3615.6
AICC (Smaller is Better)	3615.6
BIC (Smaller is Better)	3628.0

Null Model Likelihood Ratio Test		
DF	Chi-Square	Pr > ChiSq
2	90.75	<.0001

Type 3 Tests of Fixed Effects				
Effect	Num DF	Den DF	F Value	Pr > F
TEN	19	420	2.80	<.0001
ANI	1	420	29.28	<.0001
TEN*ANI	19	420	0.52	0.9532

The Mixed Procedure

Model Information	
**Data Set**	WORK.AU
**Dependent Variable**	Y
**Covariance Structure**	Autoregressive
**Subject Effect**	RP(TEN)
**Estimation Method**	REML
**Residual Variance Method**	Profile
**Fixed Effects SE Method**	Model-Based
**Degrees of Freedom Method**	Between-Within

Class Level Information		
**Class**	**Levels**	**Values**
**RP**	24	1 2 3 4 5 6 7 8 9 10 11 12 13 14 15 16 17 18 19 20 21 22 23 24
**TEN**	20	1 10 11 12 13 14 15 16 17 18 19 2 20 3 4 5 6 7 8 9
**ANI**	2	c s

Dimensions	
**Covariance Parameters**	2
**Columns in X**	63
**Columns in Z**	0
**Subjects**	465
**Max Obs per Subject**	1

Number of Observations	
**Number of Observations Read**	465
**Number of Observations Used**	460
**Number of Observations Not Used**	5

Iteration History			
**Iteration**	**Evaluations**	**-2 Res Log Like**	**Criterion**
**0**	1	3700.31309402	
**1**	1	3700.31309402	0.00000000

Convergence criteria met but final Hessian is not positive definite.

Estimated R Matrix for RP(TEN) 1 1	
**Row**	**Col1**
**1**	311.04

The Mixed Procedure

Estimated R Correlation Matrix for RP(TEN) 1 1	
Row	Col1
1	1.0000

Covariance Parameter Estimates		
Cov Parm	Subject	Estimate
AR(1)	RP(TEN)	0
Residual		311.04

Fit Statistics	
-2 Res Log Likelihood	3700.3
AIC (Smaller is Better)	3704.3
AICC (Smaller is Better)	3704.3
BIC (Smaller is Better)	3712.6

Null Model Likelihood Ratio Test		
DF	Chi-Square	Pr > ChiSq
1	0.00	1.0000

Type 3 Tests of Fixed Effects				
Effect	Num DF	Den DF	F Value	Pr > F
TEN	19	420	2.79	<.0001
ANI	1	420	29.23	<.0001
TEN*ANI	19	420	0.52	0.9517

The Mixed Procedure

Model Information	
**Data Set**	WORK.AU
**Dependent Variable**	Y
**Covariance Structure**	Heterogeneous Autoregressive
**Subject Effect**	RP(TEN)
**Estimation Method**	REML
**Residual Variance Method**	None
**Fixed Effects SE Method**	Model-Based
**Degrees of Freedom Method**	Between-Within

Class Level Information		
**Class**	**Levels**	**Values**
**RP**	24	1 2 3 4 5 6 7 8 9 10 11 12 13 14 15 16 17 18 19 20 21 22 23 24
**TEN**	20	1 10 11 12 13 14 15 16 17 18 19 2 20 3 4 5 6 7 8 9
**ANI**	2	c s

Dimensions	
**Covariance Parameters**	3
**Columns in X**	63
**Columns in Z**	0
**Subjects**	465
**Max Obs per Subject**	1

Number of Observations	
**Number of Observations Read**	465
**Number of Observations Used**	460
**Number of Observations Not Used**	5

Iteration History			
**Iteration**	**Evaluations**	**-2 Res Log Like**	**Criterion**
**0**	1	3700.31309402	
**1**	2	3634.52174871	0.00005939
**2**	1	3615.73654895	0.00002179
**3**	1	3610.09798892	0.00000080
**4**	1	3609.56313585	0.00000001

Convergence criteria met but final Hessian is not positive definite.

The Mixed Procedure

Estimated R Matrix for RP(TEN) 1 1	
**Row**	**Col1**
**1**	492.31

Estimated R Correlation Matrix for RP(TEN) 1 1	
**Row**	**Col1**
**1**	1.0000

Covariance Parameter Estimates		
**Cov Parm**	**Subject**	**Estimate**
**Var(1)**	RP(TEN)	492.31
**Var(2)**	RP(TEN)	127.18
**ARH(1)**	RP(TEN)	0

Fit Statistics	
**-2 Res Log Likelihood**	3609.6
**AIC (Smaller is Better)**	3615.6
**AICC (Smaller is Better)**	3615.6
**BIC (Smaller is Better)**	3628.0

Null Model Likelihood Ratio Test		
**DF**	**Chi-Square**	**Pr > ChiSq**
2	90.75	<.0001

Type 3 Tests of Fixed Effects				
**Effect**	**Num DF**	**Den DF**	**F Value**	**Pr > F**
**TEN**	19	420	2.80	<.0001
**ANI**	1	420	29.28	<.0001
**TEN*ANI**	19	420	0.52	0.9532

The Mixed Procedure

Model Information	
**Data Set**	WORK.AU
**Dependent Variable**	Y
**Covariance Structure**	Autoregressive Moving Average
**Subject Effect**	RP(TEN)
**Estimation Method**	REML
**Residual Variance Method**	Profile
**Fixed Effects SE Method**	Model-Based
**Degrees of Freedom Method**	Between-Within

Class Level Information		
**Class**	**Levels**	**Values**
**RP**	24	1 2 3 4 5 6 7 8 9 10 11 12 13 14 15 16 17 18 19 20 21 22 23 24
**TEN**	20	1 10 11 12 13 14 15 16 17 18 19 2 20 3 4 5 6 7 8 9
**ANI**	2	c s

Dimensions	
**Covariance Parameters**	3
**Columns in X**	63
**Columns in Z**	0
**Subjects**	465
**Max Obs per Subject**	1

Number of Observations	
**Number of Observations Read**	465
**Number of Observations Used**	460
**Number of Observations Not Used**	5

Iteration History			
**Iteration**	**Evaluations**	**-2 Res Log Like**	**Criterion**
**0**	1	3700.31309402	
**1**	1	3700.31309402	0.00000000

Convergence criteria met but final Hessian is not positive definite.

Estimated R Matrix for RP(TEN) 1 1	
**Row**	**Col1**
**1**	311.04

The Mixed Procedure

Estimated R Correlation Matrix for RP(TEN) 1 1	
**Row**	**Col1**
**1**	1.0000

Covariance Parameter Estimates		
**Cov Parm**	**Subject**	**Estimate**
**Rho**	RP(TEN)	0
**Gamma**	RP(TEN)	0
**Residual**		311.04

Fit Statistics	
**-2 Res Log Likelihood**	3700.3
**AIC (Smaller is Better)**	3706.3
**AICC (Smaller is Better)**	3706.4
**BIC (Smaller is Better)**	3718.7

Null Model Likelihood Ratio Test		
**DF**	**Chi-Square**	**Pr > ChiSq**
2	0.00	1.0000

Type 3 Tests of Fixed Effects				
**Effect**	**Num DF**	**Den DF**	**F Value**	**Pr > F**
**TEN**	19	420	2.79	<.0001
**ANI**	1	420	29.23	<.0001
**TEN*ANI**	19	420	0.52	0.9517

The Mixed Procedure

Model Information	
**Data Set**	WORK.AU
**Dependent Variable**	Y
**Covariance Structure**	Compound Symmetry
**Subject Effect**	RP(TEN)
**Estimation Method**	REML
**Residual Variance Method**	Profile
**Fixed Effects SE Method**	Model-Based
**Degrees of Freedom Method**	Between-Within

Class Level Information		
**Class**	**Levels**	**Values**
**RP**	24	1 2 3 4 5 6 7 8 9 10 11 12 13 14 15 16 17 18 19 20 21 22 23 24
**TEN**	20	1 10 11 12 13 14 15 16 17 18 19 2 20 3 4 5 6 7 8 9
**ANI**	2	c s

Dimensions	
**Covariance Parameters**	2
**Columns in X**	63
**Columns in Z**	0
**Subjects**	465
**Max Obs per Subject**	1

Number of Observations	
**Number of Observations Read**	465
**Number of Observations Used**	460
**Number of Observations Not Used**	5

Iteration History			
**Iteration**	**Evaluations**	**-2 Res Log Like**	**Criterion**
**0**	1	3700.31309402	
**1**	1	3700.31309402	0.00000000

Convergence criteria met.

Estimated R Matrix for RP(TEN) 1 1	
**Row**	**Col1**
**1**	311.04

The Mixed Procedure

Estimated R Correlation Matrix for RP(TEN) 1 1	
**Row**	**Col1**
**1**	1.0000

Covariance Parameter Estimates		
**Cov Parm**	**Subject**	**Estimate**
**CS**	RP(TEN)	310.04
**Residual**		0.9968

Fit Statistics	
**-2 Res Log Likelihood**	3700.3
**AIC (Smaller is Better)**	3704.3
**AICC (Smaller is Better)**	3704.3
**BIC (Smaller is Better)**	3712.6

Null Model Likelihood Ratio Test		
**DF**	**Chi-Square**	**Pr > ChiSq**
1	0.00	1.0000

Type 3 Tests of Fixed Effects				
**Effect**	**Num DF**	**Den DF**	**F Value**	**Pr > F**
**TEN**	19	420	2.79	<.0001
**ANI**	1	420	29.23	<.0001
**TEN*ANI**	19	420	0.52	0.9517

The Mixed Procedure

Model Information	
Data Set	WORK.AU
Dependent Variable	Y
Covariance Structure	Heterogeneous Compound Symmetry
Subject Effect	RP(TEN)
Estimation Method	REML
Residual Variance Method	None
Fixed Effects SE Method	Model-Based
Degrees of Freedom Method	Between-Within

Class Level Information		
Class	Levels	Values
RP	24	1 2 3 4 5 6 7 8 9 10 11 12 13 14 15 16 17 18 19 20 21 22 23 24
TEN	20	1 10 11 12 13 14 15 16 17 18 19 2 20 3 4 5 6 7 8 9
ANI	2	c s

Dimensions	
Covariance Parameters	3
Columns in X	63
Columns in Z	0
Subjects	465
Max Obs per Subject	1

Number of Observations	
Number of Observations Read	465
Number of Observations Used	460
Number of Observations Not Used	5

Iteration History			
Iteration	Evaluations	-2 Res Log Like	Criterion
0	1	3700.31309402	
1	2	3634.52174871	0.00005939
2	1	3615.73654895	0.00002179
3	1	3610.09798892	0.00000080
4	1	3609.56313585	0.00000001

Convergence criteria met but final Hessian is not positive definite.

The Mixed Procedure

Estimated R Matrix for RP(TEN) 1 1	
**Row**	**Col1**
**1**	492.31

Estimated R Correlation Matrix for RP(TEN) 1 1	
**Row**	**Col1**
**1**	1.0000

Covariance Parameter Estimates		
**Cov Parm**	**Subject**	**Estimate**
**Var(1)**	RP(TEN)	492.31
**Var(2)**	RP(TEN)	127.18
**CSH**	RP(TEN)	0

Fit Statistics	
**-2 Res Log Likelihood**	3609.6
**AIC (Smaller is Better)**	3615.6
**AICC (Smaller is Better)**	3615.6
**BIC (Smaller is Better)**	3628.0

Null Model Likelihood Ratio Test		
**DF**	**Chi-Square**	**Pr > ChiSq**
2	90.75	<.0001

Type 3 Tests of Fixed Effects				
**Effect**	**Num DF**	**Den DF**	**F Value**	**Pr > F**
**TEN**	19	420	2.80	<.0001
**ANI**	1	420	29.28	<.0001
**TEN*ANI**	19	420	0.52	0.9532

The Mixed Procedure

Model Information	
**Data Set**	WORK.AU
**Dependent Variable**	Y
**Covariance Structure**	Factor Analytic
**Subject Effect**	RP(TEN)
**Estimation Method**	REML
**Residual Variance Method**	None
**Fixed Effects SE Method**	Model-Based
**Degrees of Freedom Method**	Between-Within

Class Level Information		
**Class**	**Levels**	**Values**
**RP**	24	1 2 3 4 5 6 7 8 9 10 11 12 13 14 15 16 17 18 19 20 21 22 23 24
**TEN**	20	1 10 11 12 13 14 15 16 17 18 19 2 20 3 4 5 6 7 8 9
**ANI**	2	c s

Dimensions	
**Covariance Parameters**	2
**Columns in X**	63
**Columns in Z**	0
**Subjects**	465
**Max Obs per Subject**	1

Number of Observations	
**Number of Observations Read**	465
**Number of Observations Used**	460
**Number of Observations Not Used**	5

The Mixed Procedure

Model Information	
**Data Set**	WORK.AU
**Dependent Variable**	Y
**Covariance Structure**	Factor Analytic
**Subject Effect**	RP(TEN)
**Estimation Method**	REML
**Residual Variance Method**	None
**Fixed Effects SE Method**	Model-Based
**Degrees of Freedom Method**	Between-Within

Class Level Information		
**Class**	**Levels**	**Values**
**RP**	24	1 2 3 4 5 6 7 8 9 10 11 12 13 14 15 16 17 18 19 20 21 22 23 24
**TEN**	20	1 10 11 12 13 14 15 16 17 18 19 2 20 3 4 5 6 7 8 9
**ANI**	2	c s

Dimensions	
**Covariance Parameters**	4
**Columns in X**	63
**Columns in Z**	0
**Subjects**	465
**Max Obs per Subject**	1

Number of Observations	
**Number of Observations Read**	465
**Number of Observations Used**	460
**Number of Observations Not Used**	5

Iteration History			
**Iteration**	**Evaluations**	**-2 Res Log Like**	**Criterion**
**0**	1	3700.31309402	
**1**	2	3612.20956330	0.01008826
**2**	1	3609.58556841	0.00013566
**3**	1	3609.55538328	0.00000005
**4**	1	3609.55537242	0.00000000

Convergence criteria met but final Hessian is not positive definite.

The Mixed Procedure

Estimated R Matrix for RP(TEN) 1 1	
**Row**	**Col1**
**1**	496.55

Estimated R Correlation Matrix for RP(TEN) 1 1	
**Row**	**Col1**
**1**	1.0000

Covariance Parameter Estimates		
**Cov Parm**	**Subject**	**Estimate**
**FA(1)**	RP(TEN)	310.95
**FA(2)**	RP(TEN)	7.7814
**FA(1,1)**	RP(TEN)	13.6234
**FA(2,1)**	RP(TEN)	10.9319

Fit Statistics	
**-2 Res Log Likelihood**	3609.6
**AIC (Smaller is Better)**	3617.6
**AICC (Smaller is Better)**	3617.7
**BIC (Smaller is Better)**	3634.1

Null Model Likelihood Ratio Test		
**DF**	**Chi-Square**	**Pr > ChiSq**
3	90.76	<.0001

Type 3 Tests of Fixed Effects				
**Effect**	**Num DF**	**Den DF**	**F Value**	**Pr > F**
**TEN**	19	420	2.78	0.0001
**ANI**	1	420	29.07	<.0001
**TEN*ANI**	19	420	0.52	0.9549

The Mixed Procedure

Model Information	
**Data Set**	WORK.AU
**Dependent Variable**	Y
**Covariance Structure**	Huynh-Feldt
**Subject Effect**	RP(TEN)
**Estimation Method**	REML
**Residual Variance Method**	None
**Fixed Effects SE Method**	Model-Based
**Degrees of Freedom Method**	Between-Within

Class Level Information		
**Class**	**Levels**	**Values**
**RP**	24	1 2 3 4 5 6 7 8 9 10 11 12 13 14 15 16 17 18 19 20 21 22 23 24
**TEN**	20	1 10 11 12 13 14 15 16 17 18 19 2 20 3 4 5 6 7 8 9
**ANI**	2	c s

Dimensions	
**Covariance Parameters**	3
**Columns in X**	63
**Columns in Z**	0
**Subjects**	465
**Max Obs per Subject**	1

Number of Observations	
**Number of Observations Read**	465
**Number of Observations Used**	460
**Number of Observations Not Used**	5

Iteration History			
**Iteration**	**Evaluations**	**-2 Res Log Like**	**Criterion**
**0**	1	3700.31309402	
**1**	2	3786.50219864	0.00003775
**2**	1	3689.73652401	0.00006236
**3**	1	3636.81940724	0.00005737
**4**	1	3611.80245669	0.00002869
**5**	1	3609.65470481	0.00000099
**6**	1	3609.55569731	0.00000000

Convergence criteria met but final Hessian is not positive definite.

The Mixed Procedure

Estimated R Matrix for RP(TEN) 1 1	
Row	Col1
1	496.54

Estimated R Correlation Matrix for RP(TEN) 1 1	
Row	Col1
1	1.0000

Covariance Parameter Estimates		
Cov Parm	Subject	Estimate
Var(1)	RP(TEN)	496.54
Var(2)	RP(TEN)	127.07
HF	RP(TEN)	1117.59

Fit Statistics	
-2 Res Log Likelihood	3609.6
AIC (Smaller is Better)	3615.6
AICC (Smaller is Better)	3615.6
BIC (Smaller is Better)	3628.0

Null Model Likelihood Ratio Test		
DF	Chi-Square	Pr > ChiSq
2	90.76	<.0001

Type 3 Tests of Fixed Effects				
Effect	Num DF	Den DF	F Value	Pr > F
TEN	19	420	2.78	0.0001
ANI	1	420	29.08	<.0001
TEN*ANI	19	420	0.52	0.9548

The Mixed Procedure

Model Information	
**Data Set**	WORK.AU
**Dependent Variable**	Y
**Covariance Structure**	Variance Components
**Subject Effect**	RP(TEN)
**Estimation Method**	REML
**Residual Variance Method**	Parameter
**Fixed Effects SE Method**	Model-Based
**Degrees of Freedom Method**	Between-Within

Class Level Information		
**Class**	**Levels**	**Values**
**RP**	24	1 2 3 4 5 6 7 8 9 10 11 12 13 14 15 16 17 18 19 20 21 22 23 24
**TEN**	20	1 10 11 12 13 14 15 16 17 18 19 2 20 3 4 5 6 7 8 9
**ANI**	2	c s

Dimensions	
**Covariance Parameters**	1
**Columns in X**	63
**Columns in Z**	0
**Subjects**	465
**Max Obs per Subject**	1

Number of Observations	
**Number of Observations Read**	465
**Number of Observations Used**	460
**Number of Observations Not Used**	5

Iteration History			
**Iteration**	**Evaluations**	**-2 Res Log Like**	**Criterion**
**0**	1	3700.31309402	
**1**	1	3700.31309402	0.00000000

Convergence criteria met.

Estimated R Matrix for RP(TEN) 1 1	
**Row**	**Col1**
**1**	311.04

The Mixed Procedure

Estimated R Correlation Matrix for RP(TEN) 1 1	
Row	Col1
1	1.0000

Covariance Parameter Estimates		
Cov Parm	Subject	Estimate
ANI	RP(TEN)	311.04

Fit Statistics	
-2 Res Log Likelihood	3700.3
AIC (Smaller is Better)	3702.3
AICC (Smaller is Better)	3702.3
BIC (Smaller is Better)	3706.5

Null Model Likelihood Ratio Test		
DF	Chi-Square	Pr > ChiSq
0	0.00	1.0000

Type 3 Tests of Fixed Effects				
Effect	Num DF	Den DF	F Value	Pr > F
TEN	19	420	2.79	<.0001
ANI	1	420	29.23	<.0001
TEN*ANI	19	420	0.52	0.9517

The Mixed Procedure

Model Information	
**Data Set**	WORK.AU
**Dependent Variable**	Y
**Covariance Structure**	Toeplitz
**Subject Effect**	RP(TEN)
**Estimation Method**	REML
**Residual Variance Method**	Profile
**Fixed Effects SE Method**	Model-Based
**Degrees of Freedom Method**	Between-Within

Class Level Information		
**Class**	**Levels**	**Values**
**RP**	24	1 2 3 4 5 6 7 8 9 10 11 12 13 14 15 16 17 18 19 20 21 22 23 24
**TEN**	20	1 10 11 12 13 14 15 16 17 18 19 2 20 3 4 5 6 7 8 9
**ANI**	2	c s

Dimensions	
**Covariance Parameters**	2
**Columns in X**	63
**Columns in Z**	0
**Subjects**	465
**Max Obs per Subject**	1

Number of Observations	
**Number of Observations Read**	465
**Number of Observations Used**	460
**Number of Observations Not Used**	5

Iteration History			
**Iteration**	**Evaluations**	**-2 Res Log Like**	**Criterion**
**0**	1	3700.31309402	
**1**	1	3700.31309402	0.00000000

Convergence criteria met but final Hessian is not positive definite.

Estimated R Matrix for RP(TEN) 1 1	
**Row**	**Col1**
**1**	311.04

The Mixed Procedure

Estimated R Correlation Matrix for RP(TEN) 1 1	
**Row**	**Col1**
**1**	1.0000

Covariance Parameter Estimates		
**Cov Parm**	**Subject**	**Estimate**
**TOEP(2)**	RP(TEN)	0
**Residual**		311.04

Fit Statistics	
**-2 Res Log Likelihood**	3700.3
**AIC (Smaller is Better)**	3704.3
**AICC (Smaller is Better)**	3704.3
**BIC (Smaller is Better)**	3712.6

Null Model Likelihood Ratio Test		
**DF**	**Chi-Square**	**Pr > ChiSq**
1	0.00	1.0000

Type 3 Tests of Fixed Effects				
**Effect**	**Num DF**	**Den DF**	**F Value**	**Pr > F**
**TEN**	19	420	2.79	<.0001
**ANI**	1	420	29.23	<.0001
**TEN*ANI**	19	420	0.52	0.9517

The Mixed Procedure

Model Information	
**Data Set**	WORK.AU
**Dependent Variable**	Y
**Covariance Structure**	Heterogeneous Toeplitz
**Subject Effect**	RP(TEN)
**Estimation Method**	REML
**Residual Variance Method**	None
**Fixed Effects SE Method**	Model-Based
**Degrees of Freedom Method**	Between-Within

Class Level Information		
**Class**	**Levels**	**Values**
**RP**	24	1 2 3 4 5 6 7 8 9 10 11 12 13 14 15 16 17 18 19 20 21 22 23 24
**TEN**	20	1 10 11 12 13 14 15 16 17 18 19 2 20 3 4 5 6 7 8 9
**ANI**	2	c s

Dimensions	
**Covariance Parameters**	3
**Columns in X**	63
**Columns in Z**	0
**Subjects**	465
**Max Obs per Subject**	1

Number of Observations	
**Number of Observations Read**	465
**Number of Observations Used**	460
**Number of Observations Not Used**	5

Iteration History			
**Iteration**	**Evaluations**	**-2 Res Log Like**	**Criterion**
**0**	1	3700.31309402	
**1**	2	3634.52174871	0.00005939
**2**	1	3615.73654895	0.00002179
**3**	1	3610.09798892	0.00000080
**4**	1	3609.56313585	0.00000001

Convergence criteria met but final Hessian is not positive definite.

The Mixed Procedure

Estimated R Matrix for RP(TEN) 1 1	
**Row**	**Col1**
**1**	492.31

Estimated R Correlation Matrix for RP(TEN) 1 1	
**Row**	**Col1**
**1**	1.0000

Covariance Parameter Estimates		
**Cov Parm**	**Subject**	**Estimate**
**Var(1)**	RP(TEN)	492.31
**Var(2)**	RP(TEN)	127.18
**TOEPH(1)**	RP(TEN)	0

Fit Statistics	
**-2 Res Log Likelihood**	3609.6
**AIC (Smaller is Better)**	3615.6
**AICC (Smaller is Better)**	3615.6
**BIC (Smaller is Better)**	3628.0

Null Model Likelihood Ratio Test		
**DF**	**Chi-Square**	**Pr > ChiSq**
2	90.75	<.0001

Type 3 Tests of Fixed Effects				
**Effect**	**Num DF**	**Den DF**	**F Value**	**Pr > F**
**TEN**	19	420	2.80	<.0001
**ANI**	1	420	29.28	<.0001
**TEN*ANI**	19	420	0.52	0.9532

The Mixed Procedure

Model Information	
**Data Set**	WORK.AU
**Dependent Variable**	Y
**Covariance Structure**	Unstructured
**Subject Effect**	RP(TEN)
**Estimation Method**	REML
**Residual Variance Method**	None
**Fixed Effects SE Method**	Model-Based
**Degrees of Freedom Method**	Between-Within

Class Level Information		
**Class**	**Levels**	**Values**
**RP**	24	1 2 3 4 5 6 7 8 9 10 11 12 13 14 15 16 17 18 19 20 21 22 23 24
**TEN**	20	1 10 11 12 13 14 15 16 17 18 19 2 20 3 4 5 6 7 8 9
**ANI**	2	c s

Dimensions	
**Covariance Parameters**	3
**Columns in X**	63
**Columns in Z**	0
**Subjects**	465
**Max Obs per Subject**	1

Number of Observations	
**Number of Observations Read**	465
**Number of Observations Used**	460
**Number of Observations Not Used**	5

Iteration History			
**Iteration**	**Evaluations**	**-2 Res Log Like**	**Criterion**
**0**	1	3700.31309402	
**1**	1	3609.55537242	0.00000000

Convergence criteria met but final Hessian is not positive definite.

Estimated R Matrix for RP(TEN) 1 1	
**Row**	**Col1**
**1**	496.55

The Mixed Procedure

Estimated R Correlation Matrix for RP(TEN) 1 1	
**Row**	**Col1**
**1**	1.0000

Covariance Parameter Estimates		
**Cov Parm**	**Subject**	**Estimate**
**UN(1,1)**	RP(TEN)	496.55
**UN(2,1)**	RP(TEN)	0
**UN(2,2)**	RP(TEN)	127.29

Fit Statistics	
**-2 Res Log Likelihood**	3609.6
**AIC (Smaller is Better)**	3615.6
**AICC (Smaller is Better)**	3615.6
**BIC (Smaller is Better)**	3628.0

Null Model Likelihood Ratio Test		
**DF**	**Chi-Square**	**Pr > ChiSq**
2	90.76	<.0001

Type 3 Tests of Fixed Effects				
**Effect**	**Num DF**	**Den DF**	**F Value**	**Pr > F**
**TEN**	19	420	2.78	0.0001
**ANI**	1	420	29.07	<.0001
**TEN*ANI**	19	420	0.52	0.9549

The Mixed Procedure

Model Information	
**Data Set**	WORK.AU
**Dependent Variable**	Y
**Covariance Structure**	Unstructured using Correlations
**Subject Effect**	RP(TEN)
**Estimation Method**	REML
**Residual Variance Method**	None
**Fixed Effects SE Method**	Model-Based
**Degrees of Freedom Method**	Between-Within

Class Level Information		
**Class**	**Levels**	**Values**
**RP**	24	1 2 3 4 5 6 7 8 9 10 11 12 13 14 15 16 17 18 19 20 21 22 23 24
**TEN**	20	1 10 11 12 13 14 15 16 17 18 19 2 20 3 4 5 6 7 8 9
**ANI**	2	c s

Dimensions	
**Covariance Parameters**	3
**Columns in X**	63
**Columns in Z**	0
**Subjects**	465
**Max Obs per Subject**	1

Number of Observations	
**Number of Observations Read**	465
**Number of Observations Used**	460
**Number of Observations Not Used**	5

Iteration History			
**Iteration**	**Evaluations**	**-2 Res Log Like**	**Criterion**
**0**	1	3700.31309402	
**1**	2	3634.52174871	0.00005939
**2**	1	3615.73654895	0.00002179
**3**	1	3610.09798892	0.00000080
**4**	1	3609.56313585	0.00000001

Convergence criteria met but final Hessian is not positive definite.

The Mixed Procedure

Estimated R Matrix for RP(TEN) 1 1	
**Row**	**Col1**
**1**	492.31

Estimated R Correlation Matrix for RP(TEN) 1 1	
**Row**	**Col1**
**1**	1.0000

Covariance Parameter Estimates		
**Cov Parm**	**Subject**	**Estimate**
**Var(1)**	RP(TEN)	492.31
**Var(2)**	RP(TEN)	127.18
**Corr(2,1)**	RP(TEN)	0

Fit Statistics	
**-2 Res Log Likelihood**	3609.6
**AIC (Smaller is Better)**	3615.6
**AICC (Smaller is Better)**	3615.6
**BIC (Smaller is Better)**	3628.0

Null Model Likelihood Ratio Test		
**DF**	**Chi-Square**	**Pr > ChiSq**
2	90.75	<.0001

Type 3 Tests of Fixed Effects				
**Effect**	**Num DF**	**Den DF**	**F Value**	**Pr > F**
**TEN**	19	420	2.80	<.0001
**ANI**	1	420	29.28	<.0001
**TEN*ANI**	19	420	0.52	0.9532

The Mixed Procedure

Model Information	
Data Set	WORK.AU
Dependent Variable	Y
Covariance Structure	Variance Components
Subject Effect	RP(TEN)
Estimation Method	REML
Residual Variance Method	Parameter
Fixed Effects SE Method	Model-Based
Degrees of Freedom Method	Between-Within

Class Level Information		
Class	Levels	Values
RP	24	1 2 3 4 5 6 7 8 9 10 11 12 13 14 15 16 17 18 19 20 21 22 23 24
TEN	20	1 10 11 12 13 14 15 16 17 18 19 2 20 3 4 5 6 7 8 9
ANI	2	c s

Dimensions	
Covariance Parameters	1
Columns in X	63
Columns in Z	0
Subjects	465
Max Obs per Subject	1

Number of Observations	
Number of Observations Read	465
Number of Observations Used	460
Number of Observations Not Used	5

Iteration History			
Iteration	Evaluations	-2 Res Log Like	Criterion
0	1	3700.31309402	
1	1	3700.31309402	0.00000000

Convergence criteria met.

Estimated R Matrix for RP(TEN) 1 1	
Row	Col1
1	311.04

The Mixed Procedure

Estimated R Correlation Matrix for RP(TEN) 1 1	
Row	Col1
1	1.0000

Covariance Parameter Estimates		
Cov Parm	Subject	Estimate
ANI	RP(TEN)	311.04

Fit Statistics	
-2 Res Log Likelihood	3700.3
AIC (Smaller is Better)	3702.3
AICC (Smaller is Better)	3702.3
BIC (Smaller is Better)	3706.5

Null Model Likelihood Ratio Test		
DF	Chi-Square	Pr > ChiSq
0	0.00	1.0000

Type 3 Tests of Fixed Effects				
Effect	Num DF	Den DF	F Value	Pr > F
TEN	19	420	2.79	<.0001
ANI	1	420	29.23	<.0001
TEN*ANI	19	420	0.52	0.9517

Obs	Descr	V_ANTE	V_AR	V_ARH	V_ARMA	V_CS	V_CSH	V_FA1
1	-2 Res Log Likelihood	3609.6	3700.3	3609.6	3700.3	3700.3	3609.6	3609.6
2	AIC (Smaller is Better)	3615.6	3704.3	3615.6	3706.3	3704.3	3615.6	3617.6
3	AICC (Smaller is Better)	3615.6	3704.3	3615.6	3706.4	3704.3	3615.6	3617.7
4	BIC (Smaller is Better)	3628.0	3712.6	3628.0	3718.7	3712.6	3628.0	3634.1

Obs	V_HF	V_SIMPLE	V_TOEP	V_TOEPH	V_UN	V_UNR	V_VC
1	3609.6	3700.3	3700.3	3609.6	3609.6	3609.6	3700.3
2	3615.6	3702.3	3704.3	3615.6	3615.6	3615.6	3702.3
3	3615.6	3702.3	3704.3	3615.6	3615.6	3615.6	3702.3
4	3628.0	3706.5	3712.6	3628.0	3628.0	3628.0	3706.5

Obs	ANI	TEN	RP	Y
1	c	1	1	178
2	c	2	1	83
3	c	3	1	76
4	c	4	1	63
5	c	5	1	84
6	c	6	1	79
7	c	7	1	51
8	c	8	1	35
9	c	9	1	84
10	c	10	1	59
11	c	11	1	57
12	c	12	1	55
13	c	13	1	45
14	c	14	1	55
15	c	15	1	50
16	c	16	1	82
17	c	17	1	45
18	c	18	1	50
19	c	19	1	58
20	c	20	1	62
21	c	1	2	77
22	c	2	2	40
23	c	3	2	32
24	c	4	2	30
25	c	5	2	32
26	c	6	2	27
27	c	7	2	29
28	c	8	2	28
29	c	9	2	30
30	c	10	2	27
31	c	11	2	38
32	c	12	2	46
33	c	13	2	29
34	c	14	2	44
35	c	15	2	40
36	c	16	2	46
37	c	17	2	42
38	c	18	2	37
39	c	19	2	30
40	c	20	2	33
41	c	1	3	63

Obs	ANI	TEN	RP	Y
**42**	c	2	3	48
**43**	c	3	3	48
**44**	c	4	3	73
**45**	c	5	3	36
**46**	c	6	3	22
**47**	c	7	3	31
**48**	c	8	3	45
**49**	c	9	3	35
**50**	c	10	3	27
**51**	c	11	3	25
**52**	c	12	3	33
**53**	c	13	3	21
**54**	c	14	3	16
**55**	c	15	3	12
**56**	c	16	3	29
**57**	c	17	3	20
**58**	c	18	3	34
**59**	c	19	3	32
**60**	c	20	3	37
**61**	c	1	4	99
**62**	c	2	4	35
**63**	c	3	4	46
**64**	c	4	4	35
**65**	c	5	4	29
**66**	c	6	4	46
**67**	c	7	4	25
**68**	c	8	4	29
**69**	c	9	4	22
**70**	c	10	4	44
**71**	c	11	4	24
**72**	c	12	4	19
**73**	c	13	4	21
**74**	c	14	4	61
**75**	c	15	4	42
**76**	c	16	4	36
**77**	c	17	4	29
**78**	c	18	4	19
**79**	c	19	4	18
**80**	c	20	4	23
**81**	c	1	5	19
**82**	c	2	5	16

Obs	ANI	TEN	RP	Y
**83**	c	3	5	27
**84**	c	4	5	27
**85**	c	5	5	27
**86**	c	6	5	12
**87**	c	7	5	9
**88**	c	8	5	17
**89**	c	9	5	14
**90**	c	10	5	20
**91**	c	11	5	17
**92**	c	12	5	8
**93**	c	13	5	11
**94**	c	14	5	172
**95**	c	15	5	12
**96**	c	16	5	9
**97**	c	17	5	12
**98**	c	18	5	14
**99**	c	19	5	20
**100**	c	20	5	13
**101**	c	1	6	18
**102**	c	2	6	28
**103**	c	3	6	4
**104**	c	4	6	6
**105**	c	5	6	34
**106**	c	6	6	8
**107**	c	7	6	8
**108**	c	8	6	13
**109**	c	9	6	2
**110**	c	10	6	10
**111**	c	11	6	13
**112**	c	12	6	19
**113**	c	13	6	20
**114**	c	14	6	16
**115**	c	15	6	20
**116**	c	16	6	18
**117**	c	17	6	17
**118**	c	18	6	9
**119**	c	19	6	18
**120**	c	20	6	13
**121**	c	1	7	15
**122**	c	2	7	16
**123**	c	3	7	17

Obs	ANI	TEN	RP	Y
**124**	c	4	7	26
**125**	c	5	7	13
**126**	c	6	7	14
**127**	c	7	7	11
**128**	c	8	7	7
**129**	c	9	7	16
**130**	c	10	7	19
**131**	c	11	7	53
**132**	c	12	7	25
**133**	c	13	7	44
**134**	c	14	7	12
**135**	c	15	7	19
**136**	c	16	7	16
**137**	c	17	7	22
**138**	c	18	7	19
**139**	c	19	7	20
**140**	c	20	7	19
**141**	c	1	8	65
**142**	c	2	8	68
**143**	c	3	8	26
**144**	c	4	8	32
**145**	c	5	8	27
**146**	c	6	8	28
**147**	c	7	8	33
**148**	c	8	8	29
**149**	c	9	8	26
**150**	c	10	8	15
**151**	c	11	8	21
**152**	c	12	8	23
**153**	c	13	8	22
**154**	c	14	8	33
**155**	c	15	8	25
**156**	c	16	8	22
**157**	c	17	8	20
**158**	c	18	8	33
**159**	c	19	8	9
**160**	c	20	8	23
**161**	c	1	9	32
**162**	c	2	9	25
**163**	c	3	9	16
**164**	c	4	9	12

Obs	ANI	TEN	RP	Y
165	c	5	9	24
166	c	6	9	16
167	c	7	9	17
168	c	8	9	10
169	c	9	9	12
170	c	10	9	10
171	c	11	9	11
172	c	12	9	11
173	c	13	9	15
174	c	14	9	24
175	c	15	9	15
176	c	16	9	16
177	c	17	9	13
178	c	18	9	11
179	c	19	9	17
180	c	20	9	31
181	c	1	10	12
182	c	2	10	18
183	c	3	10	6
184	c	4	10	11
185	c	5	10	16
186	c	6	10	11
187	c	7	10	5
188	c	8	10	5
189	c	9	10	7
190	c	10	10	14
191	c	11	10	.
192	c	13	10	.
193	c	15	10	.
194	c	17	10	.
195	c	19	10	.
196	c	1	11	30
197	c	2	11	21
198	c	3	11	29
199	c	4	11	20
200	c	5	11	14
201	c	6	11	13
202	c	7	11	13
203	c	8	11	18
204	c	9	11	11
205	c	10	11	17

Obs	ANI	TEN	RP	Y
**206**	c	11	11	62
**207**	c	12	11	47
**208**	c	13	11	25
**209**	c	14	11	11
**210**	c	15	11	20
**211**	c	16	11	19
**212**	c	17	11	95
**213**	c	18	11	21
**214**	c	19	11	14
**215**	c	20	11	33
**216**	c	1	12	28
**217**	c	2	12	24
**218**	c	3	12	28
**219**	c	4	12	23
**220**	c	5	12	24
**221**	c	6	12	33
**222**	c	7	12	12
**223**	c	8	12	18
**224**	c	9	12	14
**225**	c	10	12	32
**226**	c	11	12	30
**227**	c	12	12	35
**228**	c	13	12	30
**229**	c	15	12	31
**230**	c	16	12	9
**231**	c	17	12	34
**232**	c	18	12	26
**233**	c	19	12	15
**234**	c	20	12	20
**235**	s	1	13	10
**236**	s	2	13	24
**237**	s	3	13	21
**238**	s	4	13	12
**239**	s	5	13	16
**240**	s	6	13	12
**241**	s	7	13	21
**242**	s	8	13	14
**243**	s	9	13	13
**244**	s	10	13	7
**245**	s	12	13	13
**246**	s	13	13	14

Obs	ANI	TEN	RP	Y
**247**	s	14	13	13
**248**	s	15	13	16
**249**	s	16	13	6
**250**	s	17	13	12
**251**	s	18	13	15
**252**	s	19	13	22
**253**	s	20	13	13
**254**	s	1	14	19
**255**	s	2	14	16
**256**	s	3	14	27
**257**	s	4	14	27
**258**	s	5	14	12
**259**	s	6	14	9
**260**	s	8	14	14
**261**	s	9	14	20
**262**	s	10	14	13
**263**	s	11	14	17
**264**	s	12	14	8
**265**	s	13	14	10
**266**	s	14	14	2
**267**	s	15	14	11
**268**	s	16	14	15
**269**	s	17	14	16
**270**	s	18	14	5
**271**	s	19	14	10
**272**	s	20	14	7
**273**	s	1	15	18
**274**	s	2	15	18
**275**	s	3	15	4
**276**	s	4	15	6
**277**	s	6	15	8
**278**	s	7	15	8
**279**	s	8	15	13
**280**	s	9	15	1
**281**	s	10	15	10
**282**	s	11	15	13
**283**	s	12	15	19
**284**	s	13	15	20
**285**	s	14	15	19
**286**	s	15	15	20
**287**	s	16	15	18

Obs	ANI	TEN	RP	Y
288	s	17	15	17
289	s	18	15	9
290	s	19	15	18
291	s	20	15	13
292	s	1	16	15
293	s	2	16	16
294	s	3	16	17
295	s	4	16	26
296	s	5	16	13
297	s	6	16	14
298	s	7	16	11
299	s	8	16	7
300	s	9	16	16
301	s	10	16	19
302	s	11	16	53
303	s	12	16	25
304	s	13	16	44
305	s	14	16	12
306	s	15	16	17
307	s	16	16	16
308	s	17	16	22
309	s	18	16	19
310	s	19	16	20
311	s	20	16	19
312	s	1	17	60
313	s	2	17	68
314	s	3	17	26
315	s	4	17	32
316	s	5	17	27
317	s	6	17	28
318	s	7	17	33
319	s	9	17	26
320	s	10	17	15
321	s	11	17	21
322	s	12	17	23
323	s	13	17	22
324	s	14	17	25
325	s	15	17	22
326	s	16	17	20
327	s	17	17	33
328	s	18	17	9

Obs	ANI	TEN	RP	Y
**329**	s	19	17	27
**330**	s	20	17	23
**331**	s	1	18	32
**332**	s	2	18	25
**333**	s	3	18	16
**334**	s	4	18	12
**335**	s	5	18	12
**336**	s	6	18	16
**337**	s	7	18	17
**338**	s	8	18	10
**339**	s	9	18	12
**340**	s	10	18	10
**341**	s	11	18	11
**342**	s	12	18	11
**343**	s	13	18	15
**344**	s	14	18	15
**345**	s	15	18	16
**346**	s	16	18	13
**347**	s	17	18	11
**348**	s	18	18	17
**349**	s	19	18	36
**350**	s	20	18	31
**351**	s	1	19	30
**352**	s	2	19	25
**353**	s	4	19	15
**354**	s	5	19	12
**355**	s	6	19	25
**356**	s	7	19	17
**357**	s	8	19	18
**358**	s	9	19	35
**359**	s	10	19	29
**360**	s	11	19	16
**361**	s	12	19	17
**362**	s	13	19	4
**363**	s	15	19	11
**364**	s	16	19	12
**365**	s	17	19	26
**366**	s	19	19	14
**367**	s	20	19	28
**368**	s	1	20	22
**369**	s	2	20	52

Obs	ANI	TEN	RP	Y
**370**	s	3	20	59
**371**	s	4	20	24
**372**	s	5	20	18
**373**	s	7	20	29
**374**	s	8	20	25
**375**	s	9	20	20
**376**	s	10	20	18
**377**	s	12	20	20
**378**	s	13	20	24
**379**	s	14	20	24
**380**	s	15	20	28
**381**	s	16	20	27
**382**	s	17	20	24
**383**	s	18	20	12
**384**	s	19	20	24
**385**	s	20	20	16
**386**	s	1	21	36
**387**	s	2	21	33
**388**	s	3	21	20
**389**	s	4	21	10
**390**	s	5	21	16
**391**	s	6	21	13
**392**	s	7	21	25
**393**	s	8	21	12
**394**	s	9	21	18
**395**	s	10	21	17
**396**	s	11	21	13
**397**	s	12	21	5
**398**	s	13	21	13
**399**	s	14	21	13
**400**	s	15	21	21
**401**	s	16	21	25
**402**	s	17	21	21
**403**	s	18	21	17
**404**	s	19	21	8
**405**	s	20	21	17
**406**	s	1	22	56
**407**	s	2	22	20
**408**	s	3	22	21
**409**	s	4	22	31
**410**	s	5	22	41

Obs	ANI	TEN	RP	Y
**411**	s	6	22	27
**412**	s	7	22	9
**413**	s	8	22	7
**414**	s	9	22	17
**415**	s	10	22	12
**416**	s	11	22	72
**417**	s	12	22	16
**418**	s	13	22	14
**419**	s	14	22	36
**420**	s	15	22	22
**421**	s	16	22	48
**422**	s	17	22	8
**423**	s	18	22	15
**424**	s	19	22	14
**425**	s	20	22	28
**426**	s	1	23	55
**427**	s	2	23	31
**428**	s	3	23	36
**429**	s	4	23	38
**430**	s	5	23	14
**431**	s	6	23	17
**432**	s	7	23	9
**433**	s	8	23	18
**434**	s	9	23	6
**435**	s	10	23	8
**436**	s	11	23	12
**437**	s	12	23	43
**438**	s	13	23	17
**439**	s	14	23	25
**440**	s	15	23	35
**441**	s	16	23	16
**442**	s	17	23	11
**443**	s	18	23	18
**444**	s	19	23	8
**445**	s	20	23	22
**446**	s	1	24	79
**447**	s	2	24	29
**448**	s	3	24	27
**449**	s	4	24	40
**450**	s	5	24	26
**451**	s	6	24	25

Obs	ANI	TEN	RP	Y
**452**	s	7	24	46
**453**	s	8	24	19
**454**	s	9	24	40
**455**	s	10	24	16
**456**	s	11	24	21
**457**	s	12	24	15
**458**	s	13	24	24
**459**	s	14	24	19
**460**	s	15	24	20
**461**	s	16	24	8
**462**	s	17	24	23
**463**	s	18	24	19
**464**	s	19	24	14
**465**	s	20	24	14

The Mixed Procedure

Model Information	
**Data Set**	WORK.AU
**Dependent Variable**	Y
**Covariance Structure**	Variance Components
**Subject Effect**	RP(TEN*ANI)
**Estimation Method**	REML
**Residual Variance Method**	Parameter
**Fixed Effects SE Method**	Model-Based
**Degrees of Freedom Method**	Between-Within

Class Level Information		
**Class**	**Levels**	**Values**
**TEN**	20	1 10 11 12 13 14 15 16 17 18 19 2 20 3 4 5 6 7 8 9
**ANI**	2	c s
**RP**	24	1 2 3 4 5 6 7 8 9 10 11 12 13 14 15 16 17 18 19 20 21 22 23 24

Dimensions	
**Covariance Parameters**	1
**Columns in X**	63
**Columns in Z**	0
**Subjects**	465
**Max Obs per Subject**	1

Number of Observations	
**Number of Observations Read**	465
**Number of Observations Used**	460
**Number of Observations Not Used**	5

Iteration History			
**Iteration**	**Evaluations**	**-2 Res Log Like**	**Criterion**
**0**	1	3700.31309402	
**1**	1	3700.31309402	0.00000000

Convergence criteria met.

Estimated R Matrix for RP(TEN*ANI) 1 1 c	
**Row**	**Col1**
**1**	311.04

The Mixed Procedure

Estimated R Correlation Matrix for RP(TEN*ANI) 1 1 c	
Row	Col1
**1**	1.0000

Covariance Parameter Estimates		
Cov Parm	Subject	Estimate
**ANI**	RP(TEN*ANI)	311.04

Fit Statistics	
**-2 Res Log Likelihood**	3700.3
**AIC (Smaller is Better)**	3702.3
**AICC (Smaller is Better)**	3702.3
**BIC (Smaller is Better)**	3706.5

Null Model Likelihood Ratio Test		
DF	Chi-Square	Pr > ChiSq
0	0.00	1.0000

Type 3 Tests of Fixed Effects				
Effect	Num DF	Den DF	F Value	Pr > F
**TEN**	19	420	2.79	<.0001
**ANI**	1	420	29.23	<.0001
**TEN*ANI**	19	420	0.52	0.9517

The Mixed Procedure

Least Squares Means							
Effect	TEN	ANI	Estimate	Standard Error	DF	t Value	Pr > |t|
TEN	1		44.5000	3.6000	420	12.36	<.0001
TEN	10		19.5000	3.6000	420	5.42	<.0001
TEN	11		28.4045	3.8529	420	7.37	<.0001
TEN	12		23.5492	3.6809	420	6.40	<.0001
TEN	13		22.0720	3.6809	420	6.00	<.0001
TEN	14		31.4273	3.8529	420	8.16	<.0001
TEN	15		22.9583	3.6809	420	6.24	<.0001
TEN	16		23.0606	3.6809	420	6.26	<.0001
TEN	17		25.1970	3.6809	420	6.85	<.0001
TEN	18		19.4545	3.7601	420	5.17	<.0001
TEN	19		20.3674	3.6809	420	5.53	<.0001
TEN	2		32.4583	3.6000	420	9.02	<.0001
TEN	20		23.5795	3.6809	420	6.41	<.0001
TEN	3		27.2462	3.6809	420	7.40	<.0001
TEN	4		26.2917	3.6000	420	7.30	<.0001
TEN	5		24.4091	3.6809	420	6.63	<.0001
TEN	6		21.6932	3.6809	420	5.89	<.0001
TEN	7		20.3939	3.6809	420	5.54	<.0001
TEN	8		17.7197	3.6809	420	4.81	<.0001
TEN	9		20.7083	3.6000	420	5.75	<.0001
ANI		c	29.2014	1.1670	420	25.02	<.0001
ANI		s	20.2977	1.1620	420	17.47	<.0001
TEN*ANI	1	c	53.0000	5.0912	420	10.41	<.0001
TEN*ANI	1	s	36.0000	5.0912	420	7.07	<.0001
TEN*ANI	10	c	24.5000	5.0912	420	4.81	<.0001
TEN*ANI	10	s	14.5000	5.0912	420	2.85	0.0046
TEN*ANI	11	c	31.9091	5.3175	420	6.00	<.0001
TEN*ANI	11	s	24.9000	5.5771	420	4.46	<.0001
TEN*ANI	12	c	29.1818	5.3175	420	5.49	<.0001
TEN*ANI	12	s	17.9167	5.0912	420	3.52	0.0005
TEN*ANI	13	c	25.7273	5.3175	420	4.84	<.0001
TEN*ANI	13	s	18.4167	5.0912	420	3.62	0.0003
TEN*ANI	14	c	44.4000	5.5771	420	7.96	<.0001
TEN*ANI	14	s	18.4545	5.3175	420	3.47	0.0006
TEN*ANI	15	c	26.0000	5.3175	420	4.89	<.0001
TEN*ANI	15	s	19.9167	5.0912	420	3.91	0.0001
TEN*ANI	16	c	27.4545	5.3175	420	5.16	<.0001
TEN*ANI	16	s	18.6667	5.0912	420	3.67	0.0003

The Mixed Procedure

Least Squares Means							
Effect	TEN	ANI	Estimate	Standard Error	DF	t Value	Pr > |t|
TEN*ANI	17	c	31.7273	5.3175	420	5.97	<.0001
TEN*ANI	17	s	18.6667	5.0912	420	3.67	0.0003
TEN*ANI	18	c	24.8182	5.3175	420	4.67	<.0001
TEN*ANI	18	s	14.0909	5.3175	420	2.65	0.0084
TEN*ANI	19	c	22.8182	5.3175	420	4.29	<.0001
TEN*ANI	19	s	17.9167	5.0912	420	3.52	0.0005
TEN*ANI	2	c	35.1667	5.0912	420	6.91	<.0001
TEN*ANI	2	s	29.7500	5.0912	420	5.84	<.0001
TEN*ANI	20	c	27.9091	5.3175	420	5.25	<.0001
TEN*ANI	20	s	19.2500	5.0912	420	3.78	0.0002
TEN*ANI	3	c	29.5833	5.0912	420	5.81	<.0001
TEN*ANI	3	s	24.9091	5.3175	420	4.68	<.0001
TEN*ANI	4	c	29.8333	5.0912	420	5.86	<.0001
TEN*ANI	4	s	22.7500	5.0912	420	4.47	<.0001
TEN*ANI	5	c	30.0000	5.0912	420	5.89	<.0001
TEN*ANI	5	s	18.8182	5.3175	420	3.54	0.0004
TEN*ANI	6	c	25.7500	5.0912	420	5.06	<.0001
TEN*ANI	6	s	17.6364	5.3175	420	3.32	0.0010
TEN*ANI	7	c	20.3333	5.0912	420	3.99	<.0001
TEN*ANI	7	s	20.4545	5.3175	420	3.85	0.0001
TEN*ANI	8	c	21.1667	5.0912	420	4.16	<.0001
TEN*ANI	8	s	14.2727	5.3175	420	2.68	0.0076
TEN*ANI	9	c	22.7500	5.0912	420	4.47	<.0001
TEN*ANI	9	s	18.6667	5.0912	420	3.67	0.0003
TEN	1		44.5000	3.6000	420	12.36	<.0001
TEN	10		19.5000	3.6000	420	5.42	<.0001
TEN	11		28.4045	3.8529	420	7.37	<.0001
TEN	12		23.5492	3.6809	420	6.40	<.0001
TEN	13		22.0720	3.6809	420	6.00	<.0001
TEN	14		31.4273	3.8529	420	8.16	<.0001
TEN	15		22.9583	3.6809	420	6.24	<.0001
TEN	16		23.0606	3.6809	420	6.26	<.0001
TEN	17		25.1970	3.6809	420	6.85	<.0001
TEN	18		19.4545	3.7601	420	5.17	<.0001
TEN	19		20.3674	3.6809	420	5.53	<.0001
TEN	2		32.4583	3.6000	420	9.02	<.0001
TEN	20		23.5795	3.6809	420	6.41	<.0001
TEN	3		27.2462	3.6809	420	7.40	<.0001

The Mixed Procedure

Least Squares Means							
Effect	TEN	ANI	Estimate	Standard Error	DF	t Value	Pr > |t|
TEN	4		26.2917	3.6000	420	7.30	<.0001
TEN	5		24.4091	3.6809	420	6.63	<.0001
TEN	6		21.6932	3.6809	420	5.89	<.0001
TEN	7		20.3939	3.6809	420	5.54	<.0001
TEN	8		17.7197	3.6809	420	4.81	<.0001
TEN	9		20.7083	3.6000	420	5.75	<.0001
ANI		c	29.2014	1.1670	420	25.02	<.0001
ANI		s	20.2977	1.1620	420	17.47	<.0001
TEN*ANI	1	c	53.0000	5.0912	420	10.41	<.0001
TEN*ANI	1	s	36.0000	5.0912	420	7.07	<.0001
TEN*ANI	10	c	24.5000	5.0912	420	4.81	<.0001
TEN*ANI	10	s	14.5000	5.0912	420	2.85	0.0046
TEN*ANI	11	c	31.9091	5.3175	420	6.00	<.0001
TEN*ANI	11	s	24.9000	5.5771	420	4.46	<.0001
TEN*ANI	12	c	29.1818	5.3175	420	5.49	<.0001
TEN*ANI	12	s	17.9167	5.0912	420	3.52	0.0005
TEN*ANI	13	c	25.7273	5.3175	420	4.84	<.0001
TEN*ANI	13	s	18.4167	5.0912	420	3.62	0.0003
TEN*ANI	14	c	44.4000	5.5771	420	7.96	<.0001
TEN*ANI	14	s	18.4545	5.3175	420	3.47	0.0006
TEN*ANI	15	c	26.0000	5.3175	420	4.89	<.0001
TEN*ANI	15	s	19.9167	5.0912	420	3.91	0.0001
TEN*ANI	16	c	27.4545	5.3175	420	5.16	<.0001
TEN*ANI	16	s	18.6667	5.0912	420	3.67	0.0003
TEN*ANI	17	c	31.7273	5.3175	420	5.97	<.0001
TEN*ANI	17	s	18.6667	5.0912	420	3.67	0.0003
TEN*ANI	18	c	24.8182	5.3175	420	4.67	<.0001
TEN*ANI	18	s	14.0909	5.3175	420	2.65	0.0084
TEN*ANI	19	c	22.8182	5.3175	420	4.29	<.0001
TEN*ANI	19	s	17.9167	5.0912	420	3.52	0.0005
TEN*ANI	2	c	35.1667	5.0912	420	6.91	<.0001
TEN*ANI	2	s	29.7500	5.0912	420	5.84	<.0001
TEN*ANI	20	c	27.9091	5.3175	420	5.25	<.0001
TEN*ANI	20	s	19.2500	5.0912	420	3.78	0.0002
TEN*ANI	3	c	29.5833	5.0912	420	5.81	<.0001
TEN*ANI	3	s	24.9091	5.3175	420	4.68	<.0001
TEN*ANI	4	c	29.8333	5.0912	420	5.86	<.0001
TEN*ANI	4	s	22.7500	5.0912	420	4.47	<.0001

The Mixed Procedure

Least Squares Means							
Effect	TEN	ANI	Estimate	Standard Error	DF	t Value	Pr > |t|
TEN*ANI	5	c	30.0000	5.0912	420	5.89	<.0001
TEN*ANI	5	s	18.8182	5.3175	420	3.54	0.0004
TEN*ANI	6	c	25.7500	5.0912	420	5.06	<.0001
TEN*ANI	6	s	17.6364	5.3175	420	3.32	0.0010
TEN*ANI	7	c	20.3333	5.0912	420	3.99	<.0001
TEN*ANI	7	s	20.4545	5.3175	420	3.85	0.0001
TEN*ANI	8	c	21.1667	5.0912	420	4.16	<.0001
TEN*ANI	8	s	14.2727	5.3175	420	2.68	0.0076
TEN*ANI	9	c	22.7500	5.0912	420	4.47	<.0001
TEN*ANI	9	s	18.6667	5.0912	420	3.67	0.0003
TEN*ANI	1	c	53.0000	5.0912	420	10.41	<.0001
TEN*ANI	1	s	36.0000	5.0912	420	7.07	<.0001
TEN*ANI	10	c	24.5000	5.0912	420	4.81	<.0001
TEN*ANI	10	s	14.5000	5.0912	420	2.85	0.0046
TEN*ANI	11	c	31.9091	5.3175	420	6.00	<.0001
TEN*ANI	11	s	24.9000	5.5771	420	4.46	<.0001
TEN*ANI	12	c	29.1818	5.3175	420	5.49	<.0001
TEN*ANI	12	s	17.9167	5.0912	420	3.52	0.0005
TEN*ANI	13	c	25.7273	5.3175	420	4.84	<.0001
TEN*ANI	13	s	18.4167	5.0912	420	3.62	0.0003
TEN*ANI	14	c	44.4000	5.5771	420	7.96	<.0001
TEN*ANI	14	s	18.4545	5.3175	420	3.47	0.0006
TEN*ANI	15	c	26.0000	5.3175	420	4.89	<.0001
TEN*ANI	15	s	19.9167	5.0912	420	3.91	0.0001
TEN*ANI	16	c	27.4545	5.3175	420	5.16	<.0001
TEN*ANI	16	s	18.6667	5.0912	420	3.67	0.0003
TEN*ANI	17	c	31.7273	5.3175	420	5.97	<.0001
TEN*ANI	17	s	18.6667	5.0912	420	3.67	0.0003
TEN*ANI	18	c	24.8182	5.3175	420	4.67	<.0001
TEN*ANI	18	s	14.0909	5.3175	420	2.65	0.0084
TEN*ANI	19	c	22.8182	5.3175	420	4.29	<.0001
TEN*ANI	19	s	17.9167	5.0912	420	3.52	0.0005
TEN*ANI	2	c	35.1667	5.0912	420	6.91	<.0001
TEN*ANI	2	s	29.7500	5.0912	420	5.84	<.0001
TEN*ANI	20	c	27.9091	5.3175	420	5.25	<.0001
TEN*ANI	20	s	19.2500	5.0912	420	3.78	0.0002
TEN*ANI	3	c	29.5833	5.0912	420	5.81	<.0001
TEN*ANI	3	s	24.9091	5.3175	420	4.68	<.0001

The Mixed Procedure

Least Squares Means							
Effect	TEN	ANI	Estimate	Standard Error	DF	t Value	Pr > |t|
TEN*ANI	4	c	29.8333	5.0912	420	5.86	<.0001
TEN*ANI	4	s	22.7500	5.0912	420	4.47	<.0001
TEN*ANI	5	c	30.0000	5.0912	420	5.89	<.0001
TEN*ANI	5	s	18.8182	5.3175	420	3.54	0.0004
TEN*ANI	6	c	25.7500	5.0912	420	5.06	<.0001
TEN*ANI	6	s	17.6364	5.3175	420	3.32	0.0010
TEN*ANI	7	c	20.3333	5.0912	420	3.99	<.0001
TEN*ANI	7	s	20.4545	5.3175	420	3.85	0.0001
TEN*ANI	8	c	21.1667	5.0912	420	4.16	<.0001
TEN*ANI	8	s	14.2727	5.3175	420	2.68	0.0076
TEN*ANI	9	c	22.7500	5.0912	420	4.47	<.0001
TEN*ANI	9	s	18.6667	5.0912	420	3.67	0.0003

Tests of Effect Slices						
Effect	TEN	ANI	Num DF	Den DF	F Value	Pr > F
TEN*ANI	1		1	420	5.57	0.0187
TEN*ANI	10		1	420	1.93	0.1656
TEN*ANI	11		1	420	0.83	0.3636
TEN*ANI	12		1	420	2.34	0.1267
TEN*ANI	13		1	420	0.99	0.3213
TEN*ANI	14		1	420	11.34	0.0008
TEN*ANI	15		1	420	0.68	0.4091
TEN*ANI	16		1	420	1.42	0.2333
TEN*ANI	17		1	420	3.15	0.0768
TEN*ANI	18		1	420	2.03	0.1545
TEN*ANI	19		1	420	0.44	0.5059
TEN*ANI	2		1	420	0.57	0.4523
TEN*ANI	20		1	420	1.38	0.2402
TEN*ANI	3		1	420	0.40	0.5258
TEN*ANI	4		1	420	0.97	0.3258
TEN*ANI	5		1	420	2.31	0.1295
TEN*ANI	6		1	420	1.21	0.2710
TEN*ANI	7		1	420	0.00	0.9869
TEN*ANI	8		1	420	0.88	0.3496
TEN*ANI	9		1	420	0.32	0.5709
TEN*ANI		c	19	420	2.25	0.0021
TEN*ANI		s	19	420	1.05	0.4019
